# Risk factors associated with aggressive tumor phenotypes in papillary thyroid microcarcinoma: a systematic review and meta-analysis

**DOI:** 10.3389/fendo.2026.1876912

**Published:** 2026-07-02

**Authors:** Máté Orgoványi, Anett Rancz, Anca Dolhascu, Gergely Agócs, Boglárka Lilla Szentes, Emese Sipter, Péter Hegyi, Gábor László Kovács

**Affiliations:** 1 Centre for Translational Medicine, Semmelweis University, Budapest, Hungary; 2 Department of Internal Medicine, Uzsoki Hospital, Budapest, Hungary; 3 Department of Rheumatology and Immunology, Semmelweis University, Budapest, Hungary; 4 Department of Pneumology, Institute of Pneumology “Marius Nasta”, Bucharest, Romania; 5 Department of Biophysics and Radiation Biology, Semmelweis University, Budapest, Hungary; 6 Department of Internal Medicine and Haematology, Semmelweis University, Budapest, Hungary; 7 Institute for Translational Medicine, Medical School, University of Pécs, Pécs, Hungary; 8 Institute of Pancreatic Diseases, Semmelweis University, Budapest, Hungary; 9 Department of Internal Medicine, North-Pest Central Hospital – Military Hospital, Budapest, Hungary

**Keywords:** papillary thyroid microcarcinoma, subcentimeter papillary thyroid microcarcinoma, lymph node metastasis, extrathyroidal extension, risk factor, active surveillance, BRAFV600E mutation, meta-analysis

## Abstract

**Introduction:**

In line with current guidelines, risk stratification in papillary thyroid microcarcinoma (PTMC) has shifted from simple diameter-based assessments to evaluation of individual tumor biology. Therefore, this study aimed to investigate how baseline clinical and molecular risk factors influence aggressive PTMC phenotypes.

**Methods:**

Registered in PROSPERO (CRD42024603662), we searched PubMed, Embase, and Cochrane through February 14, 2025. We analyzed risk factors for lymph node metastasis (LNM) and extrathyroidal extension (ETE) utilizing data from surgical cohorts of patients with PTMC, calculating pooled odds ratios (OR) and 95% confidence intervals (CI).

**Results:**

A total of 274 articles we found increased odds of central lymph node metastasis (CLNM) associated with male sex (OR = 1.86; CI:1.73–2.00), age (<45 and <55 years old OR = 1.61; CI:1.42–1.83 and OR = 1.89; CI:1.71–2.09), tumor size (>5 and >7 mm OR = 2.26; CI:2.00–2.56 and OR = 1.81; CI:1.11–2.96), multifocality (OR = 1.96; CI:1.78–2.16), bilaterality (OR = 1.70; CI:1.45–2.00) and BRAFV600E mutation (OR = 1.51; CI:1.25–1.82). Lateral lymph node metastasis (LLNM) was associated with male sex (OR = 1.69; CI:1.51–1.90), age (<45 and <55 years old OR = 1.5; CI:1.28–1.76 and CI:1.31–1.72), tumor size (>5 mm and >7 mm, OR = 2.34; CI:1.68–3.25 and OR = 3.71; CI:3.15–4.36), multifocality (OR = 1.98; CI:1.46–2.70) and bilaterality (OR = 1.91; CI:1.22–3.00). For ETE, risk factors were as follows: tumor size (>5 mm OR = 3.00; CI:2.03–4.45), multifocality (OR = 2.41; CI:1.99–2.91), bilaterality (OR = 2.58; CI:1.26–5.27), and BRAFV600E mutation (OR = 2.19; CI:1.61–2.97).

**Conclusion:**

Male sex, age (<45 and <55 years), tumor size (>5 mm), multifocality, bilaterality, and BRAF^V600E^ mutation are clinically relevant risk factors for aggressive PTMC phenotypes.

**Systematic review registration:**

https://www.crd.york.ac.uk/PROSPERO/view/CRD42024603662, identifier CRD42024603662.

## 1 Introduction

Papillary thyroid microcarcinoma (PTMC) affects one in ten individuals; however, it is a very indolent disease in most cases (1). Autopsy studies have reported an average prevalence of PTMC of 11.5% (2). In the Netherlands, more than 1,000 people were diagnosed with PTMC between 2005 and 2015 (3). A report from the Surveillance, Epidemiology, and End Results (SEER) database from 1975 to 2018 found a rising incidence of papillary thyroid cancers (4). This phenomenon is due to improvements in ultrasound (US) imaging, which enable the detection of tumors at an earlier stage (5).

In line with the 2025 American Thyroid Association (ATA) guidelines (6), risk stratification for differentiated thyroid cancer has fundamentally transitioned toward a more dynamic assessment of individual tumor biology. In the current clinical landscape, where management strategies are becoming increasingly nuanced, relying solely on historical postoperative recurrence scales or tumor diameter is no longer sufficient. Rather than viewing PTMC as a uniform entity, modern oncology should focuses on the multi-parametric characterization of individual tumor phenotypes.

These aggressive phenotypes typically manifest as central lymph node metastasis (CLNM), lateral lymph node metastasis (LLNM), or extrathyroidal extension (ETE) at the time of diagnosis. While their presence is closely linked to baseline clinicopathological traits, such as sex, age, tumor size, and genetic mutations, these variables do not exist in isolation in a real-world setting; instead, they interact within the same patient. Therefore, an objective, baseline assessment of the clinical presentation is critical.

The aim of this study was to evaluate the clinical and molecular risk factors associated with aggressive phenotypes in PTMC, establishing a clear clinicopathological baseline for individual patient profiles.

## 2 Methods

This work was conducted out as part of the Systems Education Program (7) at Semmelweis University and within the Translational Medicine (TM) Cycle Framework of the Academia Europaea (8).

We conducted our systematic review and meta-analysis in accordance with the PRISMA 2020 guidelines (9) (**Supplementary Table 1**) and followed the Cochrane Handbook (10). The protocol of the study was registered in PROSPERO (registration number CRD42024603662), and we fully adhered to it.

### 2.1 Eligibility criteria

We used the PFO framework (population, factor(s), outcome) for our study. The population (P) comprised patients with histologically confirmed PTMC with modifiable and non-modifiable risk factors (F), and the outcome (O) was defined as presence or developement lymph node metastasis (LNM) and/or ETE and/or tumor size increase (SI) of at least >3 mm.

Cohorts (mainly surgical cohorts evaluating post-operative histopathology), case-control, and cross-sectional studies were included if they satisfied the elements of our framework.

### 2.2 Information sources

Our systematic search was conducted on February 14, 2025 in three databases: MEDLINE (via PubMed), Embase, and Cochrane Central Register of Controlled Trials (CENTRAL). Furthermore, the systematic search was performed using backward and forward reference searches of eligible articles on July 7, 2025.

### 2.3 Search strategy

For our search protocol, we used a search key focusing on one domain that describes the population (**Supplementary Material 2a**).

### 2.4 Selection process

The search results were imported into a reference manager (EndNote X9, Clarivate Analytics, Philadelphia, PA, USA). The selection was performed independently by two review authors (MO and AD). After automatic and manual removal of duplicates, records were screened by title and abstract, followed by full-text assessment. For articles with unavailable full texts, data requests were sent to the corresponding authors. Cohen’s kappa coefficient (*κ*) was calculated at every main step to check the inter-rater reliability. Any potential disagreement was resolved by a third independent investigator (AR).

### 2.5 Data collection process

Data from eligible articles were independently extracted by two authors (MO and AD) using a predesigned Excel sheet (Office 365, Microsoft, Redmond, WA, USA). Any disagreements were resolved by a third independent investigator (AR). The extracted data were organized into 2 x 2 contingency tables, detailing the number of the PTMC patients with and without each risk factor, stratified by the different outcomes (**Supplementary Material 2b**).

### 2.6 Data items

We extracted the following data from each study: first author, year of publication, digital object identifier (DOI), study design, study period, country of origin, study centers, sample size and the percentage of female participants, population age (mean +/- SD or median +/- IQR or range), observed factors (yes/no or mean +/- SD) and the number of the patients by the outcomes – LNM (CLNM/LLNM/undefined (udLNM) - yes/no), ETE (yes/no – microscopic or gross ETE), tumor SI (yes/no).

### 2.7 Study risk of bias assessment

Two authors (MO and AD) independently performed the risk of bias (RoB) assessment with the help of the Quality in Prognosis Studies (QUIPS) tool. The RoB was assessed for each study, and in cases of disagreement, a third reviewer (AR) was consulted.

### 2.8 Reporting bias assessment

To assess the RoB due to missing results, we visually inspected funnel plots. For outcomes involving more than 10 studies, Egger’s test was performed to statistically evaluate the asymmetry of the plots. A p-value < 0.05 was considered indicative of significant publication bias.

### 2.9 Synthesis methods

This analysis focuses on both modifiable (body mass index, pregnancy) and non-modifiable risk factors (age, sex, tumor size, tumor focality, bilaterality, tumor localization in the lobe, microcalcification, invasion of capsule, thyreoglobulin antibody (anti-Tg), thyroid-stimulating hormone (TSH), Graves’ disease, goiter, Hashimoto’s thyroiditis (HT), and various genetic markers) in the progression of CLNM, LLNM, udLNM, and ETE. The pooled effect size was crude or adjusted odds ratios (OR), which were extracted or calculated from each manuscript. As studies used different cut-offs for the same risk factor (age, tumor size), we conducted analyses for all feasible cut-offs. When clear cut-off was not reported, the mean value of the variable was used, and a rma.mv model was fitted (11).

Pooled results were calculated when more than two studies were available for a given outcome. To estimate the heterogeneity variance measure (τ2), we used the restricted maximum-likelihood estimator with the Q profile method for confidence intervals (12).

All statistical analyses were performed using R (13), with the meta package for basic meta-analysis calculations and plots (14), and the dmetar (15) package for additional influential analysis calculations and plots within the MetaBoostR framework (16). Analyses were based on the recommendations of Harrer et al. (17).

## 3 Results

### 3.1 Search and selection

**Figure 1:**
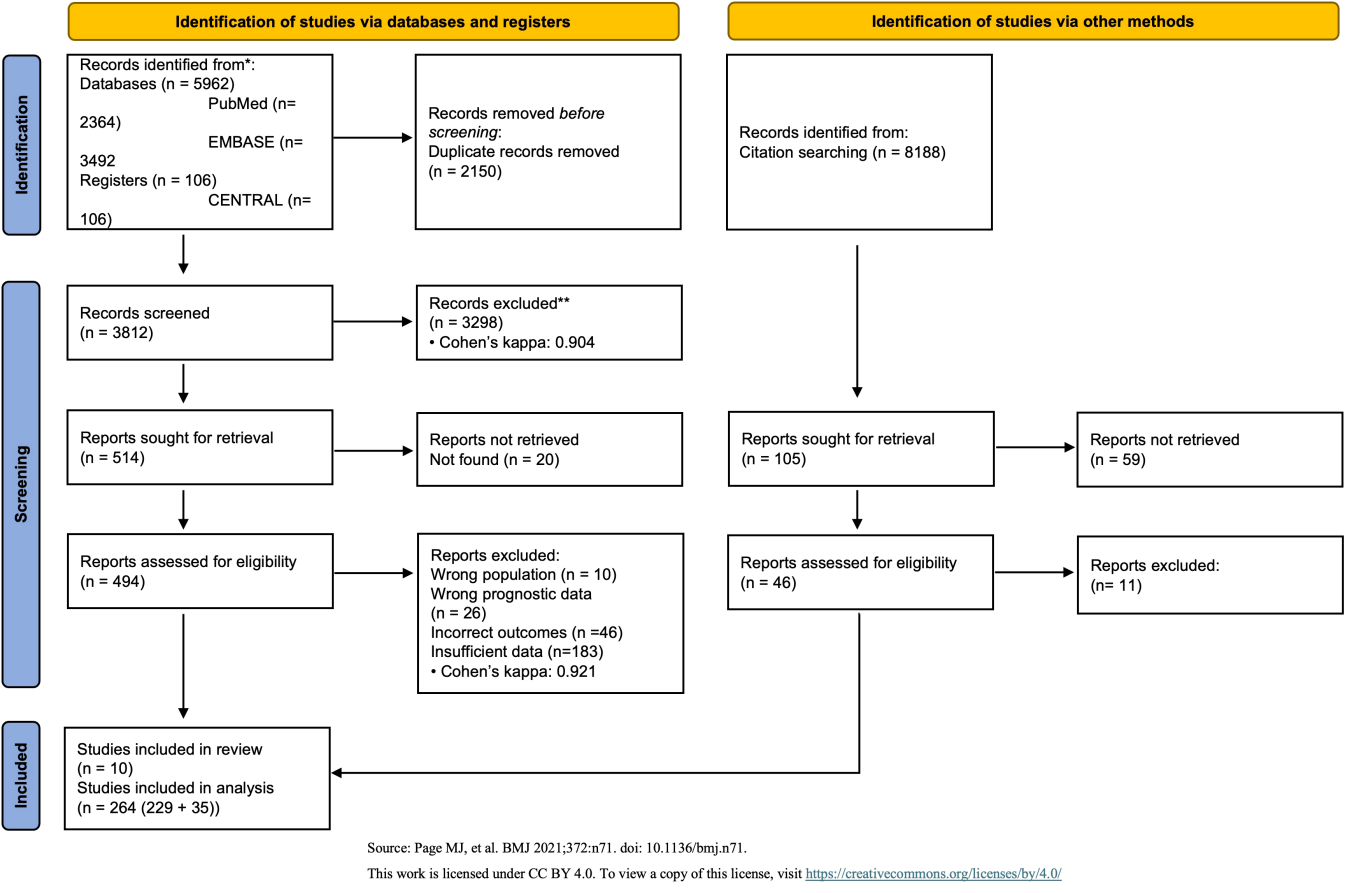
PRISMA 2020 flowchart representing the study selection process. The flowchart illustrates the systematic identification, screening, and inclusion of studies for the meta-analysis. Initially, 5962 records were identified through database searching (PubMed, Embase, and Cochrane Library) and 8188 via other methods. After the removal of duplicates, titles and abstracts were screened, resulting in 494 + 46 records for full-text assessment. A total of 274 studies (10 prospective and 264 retrospective) met the final inclusion criteria for the systematic review and meta-analysis. Exclusion at the full-text stage was primarily due to ineligible study populations and lack of relevant outcome data.

Altogether, 3,812 studies were screened for our systematic search, supplemented by 261 hits identified through backward and forward reference searching. Of these, 274 articles were deemed eligible for our final analysis. From this pool, 10 articles were included in the systematic review, and 264 surgical cohort studies were suitable for meta-analysis (**Figure 1**).

### 3.2 Basic characteristics of included studies

A summary of baseline characteristics of the included articles is presented in **Table 1**.

**Table 1:** Basic characteristics of included studies.

Study number	Country	No. of patients	Quality of study	MC/SC	Smallest and largest population	QUIPS – low risk
274	23	225,307	264 retrospective cohort	14/257 1 database and 2 n.a.	Ghirri et al., 5 patients (21) Ding et al., 35118 patients (22)	43 (1,57%)

MC/SC: *multi-center/single-center*; n.a.: *not applicable*; No.: *number of*; QUIPS: *Quality In Prognosis Studies*.

**Supplementary Table 2** provides more detailed information on each study, and **Supplementary Table 3** summarizes the main predictors by outcome.

We analyzed more than 220,000 patients across 264 retrospective studies. Most studies were single-center studies, and a majority of the articles originated from Asian populations (80.1%). The smallest study, by Ghirri et al. (18), included 20 patients, while Ding et al. (19) included more than 35,000 PTMC patients.

### 3.3 Risk factors of lymph node metastasis

#### 3.3.1 Sex

The relationship between male sex and risk of LNM in univariate analysis was as follows: the OR for CLNM was 1.86 (CI: 1.73–2.00; I^2^ = 49%, CI: 36–59), for LLNM, it was 1.69 (CI: 1.51–1.90; I^2^ = 57%, CI: 40–69), and finally, for udLNM, the OR was 1.91 (CI: 1.79–2.05; I^2^ = 9%, CI: 0–36). Regarding the pooled adjusted ORs, the results were as follows: the OR for CLNM was 1.77 (CI: 1.33–2.36; I^2^ = 80%, CI: 71–86), for LLNM, it was 1.39 (CI: 0.96–2.01; I^2^ = 50%, CI: 3–74), and finally, for udLNM, the OR was 1.73 (CI: 1.26–2.38; I^2^ = 53%, CI: 11–76).

#### 3.3.2 Age

The relationship between age (<45 years and <55 years) and risk of LNM in univariate analysis was as follows: for CLNM, the OR was 1.61 (CI: 1.42–1.83; I^2^ = 81%, CI: 76–85) and 1.89 (CI: 1.71–2.09; I^2^ = 25%, CI: 0–58), for LLNM, it was 1.5 equally (CI: 1.28–1.76; I^2^ = 11%, CI: 0–47 and CI: 1.31–1.72; I^2^ = 15%, CI: 0–53), finally, for udLNM, the OR was 1.72 (CI: 1.50–1.97; I^2^ = 50%, CI: 18–70) and 1.29 (CI: 0.84–1.98; I^2^ = 76%, CI: 58–86). We also performed meta-regression (**Supplementary Table 4**). Regarding the pooled adjusted ORs, the results were as follows: the OR for CLNM was 2.01 (CI: 1.16–3.49; I^2^ = 91%, CI: 85–95), and 2.14 (CI: 1.64–2.80; I^2^ = 20%, CI: 0–65, for udLNM, the OR was 0.99 (CI: 0.48–2.05; I^2^ = 81%, CI: 55–92) and 1.96 (CI: 0.21–18.72; I^2^ = 90%, CI: 75–96).

#### 3.3.3 Tumor size

The relationship between size (above 5 mm, 6 mm, 6.5 mm and 7 mm) and risk of LNM in univariate analysis was as follows: for CLNM, the OR was 2.26 (CI: 2.00–2.56; I^2^ = 75%, CI: 68–80) for 5 mm, 2.24 (CI: 0.75–6.72; I^2^ = 89%, CI: 70–96) for 6.5 mm, and 1.81 (CI: 1.11–2.96; I^2^ = 68%, CI: 25–87) for 7 mm. For LLNM (above 5 and 7 mm), the OR was 2.34 (CI: 1.68–3.25; I^2^ = 81%, CI: 72–87) and 3.71 (CI: 3.15–4.36; I^2^ = 0%, CI: 0–75). Finally, for udLNM (above 5 and 7 mm), the OR was 2.28 (CI: 1.80–2.88; I^2^ = 77%, CI: 68–83) and 2.52 (CI: 1.99–3.18; I^2^ = 0%, CI: 0–79). We also performed meta-regression (**Supplementary Table 4**). Regarding the pooled adjusted ORs, the results were as follows: the OR for CLNM (> 5 mm) was 2.07 (CI: 1.63–2.64; I^2^ = 87%, CI: 79–91), for CLNM (> 7 mm) was 2.09 (CI: 0.73–5.97; I^2^ = 60%, CI: 0–89), for LLNM (> 5 mm), it was 2.27 (CI: 0.75–6.91; I^2^ = 92%, CI: 83–96), and finally, for udLNM (> 5 mm), the OR was 1.89 (CI: 1.61–2.23; I^2^ = 0%, CI: 0–71).

#### 3.3.4 Multifocality

The relationship between multifocality and risk of LNM in univariate analysis was as follows: the OR for CLNM was 1.96 (CI: 1.78–2.16; I^2^ = 78%, CI: 74–82), for LLNM, it was 1.98 (CI: 1.46–2.70; I^2^ = 90%, CI: 87–92) and, finally, for udLNM, the OR was 2.28 (CI: 1.85–2.82; I^2^ = 73%, CI: 63–80). Regarding the pooled adjusted ORs, the results were as follows: the OR for CLNM was 1.71 (CI: 1.38–2.12; I^2^ = 74%, CI: 59–83), for LLNM, it was 2.06 (CI: 1.55–2.75; I^2^ = 34%, CI: 0–70), and finally, for udLNM, the OR was 1.89 (CI: 1.61–2.22; I^2^ = 3%, CI: 0–66).

#### 3.3.5 Bilaterality

The relationship between bilaterality and risk of LNM in univariate analysis was as follows:

For CLNM, the OR was 1.70 (CI: 1.45–2.00; I^2^ = 84%, CI: 79–87), for LLNM, it was 1.91 (CI: 1.22–3.00; I^2^ = 80%, CI: 68–87) and, finally, for udLNM, it was 2.01 (CI: 1.16–3.49; I^2^ = 81%, CI: 72–88). Regarding the pooled adjusted ORs, the results were as follows: the OR for CLNM was 1.47 (CI: 1.09–2.00; I^2^ = 46%, CI: 0–73).

#### 3.3.6 BRAF^V600E^ mutation

The relationship between the BRAF^V600E^ mutation and risk of LNM in univariate analysis was as follows: for CLNM, the OR was 1.51 (CI: 1.25–1.82; I^2^ = 59%, CI: 40–72), for LLNM, it was 1.11 (CI: 0.69–1.78; I^2^ = 61%, CI: 35–76) and, finally, for udLNM, it was 1.51 (CI: 1.14–1.98; I^2^ = 58%, CI: 33–74). Regarding the pooled adjusted ORs, the results were as follows: the OR for CLNM was 2.20 (CI: 1.15–4.18; I^2^ = 64%, CI: 5–86), and for udLNM, the OR was 1.22 (CI: 0.06–26.54; I^2^ = 83%, CI: 48–94).

#### 3.3.7 Hashimoto’s thyroiditis

The relationship between Hashimoto’s thyroiditis and risk of LNM was the following: The OR in case of CLNM was 0.91 (CI: 0.85–0.98; I^2^ = 46%, CI: 27–59), at LLNM it was 1.02 (CI: 0.82–1.27; I^2^ = 71%, CI: 58–80) and finally at udLNM the OR was 0.82 (CI: 0.7–0.96; I^2^ = 65%, CI: 58–76). Regarding the pooled adjusted ORs, the results were as follows: the OR for CLNM was 0.56 (CI: 0.15–2.14; I^2^ = 93%, CI: 87–96), for LLNM, it was 1.40 (CI: 0.88–2.20; I^2^ = 0%, CI: 0–90), and finally, for udLNM, the OR was 0.77 (CI: 0.42–1.42; I^2^ = 85%, CI: 67–93).

#### 3.3.8 Obesity, Graves’ disease, anti-Tg, goiter, increased TSH, TERT mutation, microcalcification, and capsule invasion

Detailed findings for these factors, including those identified as non-risk and those identified as predictors, are provided in **Supplementary Materials 3a, b**.

Summary forest plots are shown in **Figures 2** and **3**. Moreover, all forest plots are in **Supplementary Figures 1–88**.

**Figure 2:**
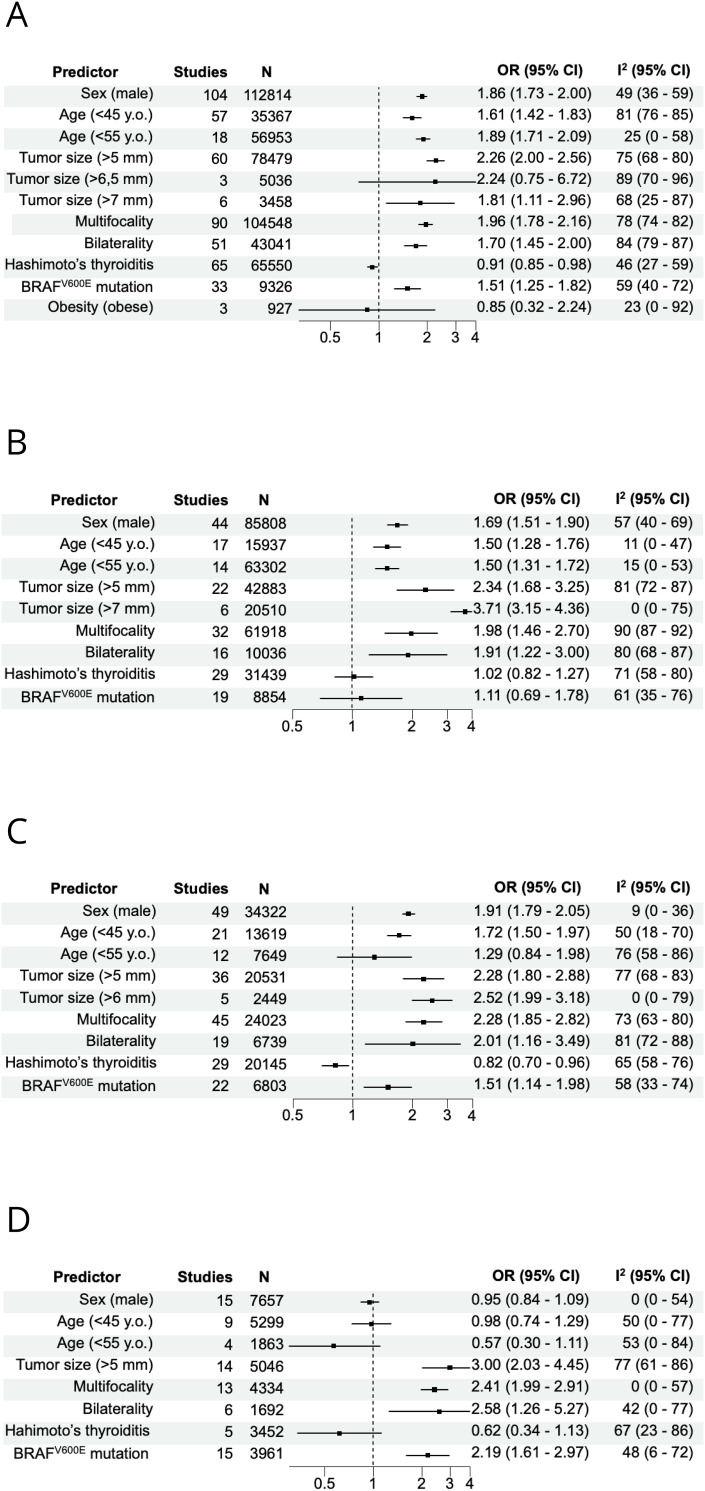
Summary forest plots representing the unadjusted odds of different risk factors in the case of **(A)** central lymph node metastasis, **(B)** lateral lymph node metastasis, **(C)** undefined lymph node metastasis and **(D)** extrathyroidal extension. The plot presents the pooled odds ratios (OR) and 95% confidence intervals (CI) for the identified risk factors. Each row represents a specific meta-analysis of the association between the given factor and the given outcome. The square dot represents the overall pooled effect size for each category. Statistical significance is reached where the CI does not cross the vertical null line (OR = 1.0). Between-study heterogeneity was assessed using the I^2^ statistic. BRAF^V600E^, v-Raf murine sarcoma viral oncogene homolog B1; CI, confidence interval; I^2^, heterogeneity; mm, millimeter; N, sample size; OR, odds ratio; y.o., years old; V600E, valine-to-glutamic acid substitution at codon 600.

**Figure 3:**
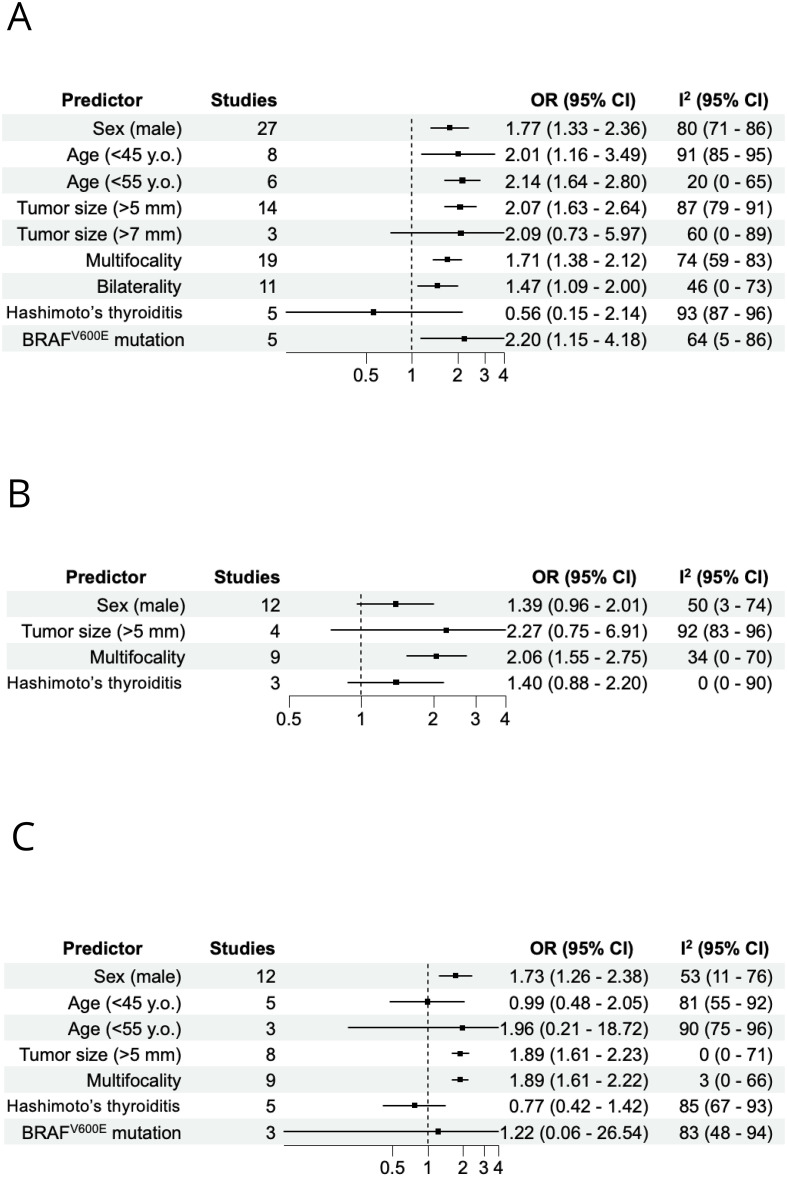
Summary forest plots representing the pooled adjusted odds of different risk factors in the case of **(A)** central lymph node metastasis, **(B)** lateral lymph node metastasis and **(C)** undefined lymph node metastasis. The plot presents the pooled odds ratios (OR) and 95% confidence intervals (CI) for the identified risk factors. Each row represents a specific meta-analysis of the association between the given factor andthe given outcome. The square dot represents the overall pooled effect size for each category. Statistical significance is reached where the CI does not cross the vertical null line (OR = 1.0). Between-study heterogeneity was assessed using the I^2^ statistic. BRAF^V600E^, v-Raf murine sarcoma viral oncogene homolog B1; CI, confidence interval; I^2^, heterogeneity; mm, millimeter; N, sample size; OR, odds ratio; y.o., years old; V600E, valine-to-glutamic acid substitution at codon 600.

### 3.4 Risk factors of extrathyroidal extension

#### 3.4.1 Sex

The relationship between the male sex and the risk of ETE in univariate analysis was as follows: OR 0.95 (CI: 0.84–1.09; I2 = 0%; CI: 0–54).

#### 3.4.2 Age

The relationship between age (<45 years and <55 years) and risk of ETE in univariate analysis was as follows: OR 0.98 (CI: 0.74–1.29; I^2^ = 50%, CI: 0–77) and OR 0.57 (CI: 0.30–1.11; I^2^ = 53%, CI: 0–84).

#### 3.4.3 Tumor size

The relationship between size (above 5 mm) and risk of ETE in univariate analysis was as follows: OR 3.00 (CI: 2.03–4.45; I^2^ = 77%, CI: 61–86).

#### 3.4.4 Multifocality

The relationship between multifocality and ETE in univariate analysis was as follows: OR 2.41 (CI: 1.99–2.91; I^2^ = 0%, CI: 0–57).

#### 3.4.5 Bilaterality

The relationship between bilaterality and ETE in univariate analysis was as follows: OR 2.58 (CI: 1.26–5.27; I^2^ = 42%, CI: 0–77).

#### 3.4.6 BRAF^V600E^ mutation

The relationship between the BRAF^V600E^ mutation and risk of ETE in univariate analysis was as follows: OR 2.19 (CI: 1.61–2.97; I^2^ = 48%, CI: 6–72).

#### 3.4.7 Hashimoto’s thyroiditis

Hashimoto’s thyroiditis is not considered a risk factor for ETE: OR 0.62 (CI: 0.34–1.13; I^2^ = 67%, CI: 23-86).

All forest plots are available in **Supplementary Figure 1–52**.

### 3.5 Risk factors of tumor size increase

Previously, only surgical treatment was recommended for differentiated thyroid cancer (DTC), including PTMC. Before surgery, a dynamic risk stratification was performed, as described in the latest ATA guidelines (6). This was mostly used to calculate the risk of persistent or recurrent disease based on various pathological characteristics. This approach achieves favorable all-cause and cancer-specific mortality, but adverse events (e.g., temporary or permanent vocal cord paralysis or hypoparathyroidism) may be a disadvantage (20–22).

Nowadays, a new noninvasive treatment option has emerged for PTMC, in which specialists follow selected patients every 6 or 12 months and recommend further intervention, such as surgery, if the tumor size increases by at least 3 mm and/or novel lymph node metastasis is detected (6). This method is called active surveillance (AS). This treatment option may be a reasonable choice in many cases; however, due to the low certainty evidence, it is not widely applied in everyday practice. AS is associated with fewer adverse effects compared with surgery; on the other hand, the psychological burden – including daily stress and anxiety – experienced by both patients and clinicians cannot be ignored (22–24). The ATA guidelines recommend AS as an acceptable management option in selected patients; however, it does not specify the ideal candidates, and no risk stratification exists for patients with PTMC.

Due to heterogeneous definitions, a meta-analysis of data was not feasible; however, several patterns were detected. Sex was reported as a risk factor in four articles (25–28), but only one performed a multivariate analysis (26), which found that male sex was associated with higher odds of progression than female. Furthermore, younger age was consistently associated with tumor enlargement, with multiple studies reporting higher progression rates in patients under 30–45 years of age (25–28, 33). Associations with initial tumor size were less consistent (25–28). While some cohorts identified initial nodules ≥5–7 mm as having a higher likelihood of growth, others found no significant predictive value. Multifocality was evaluated in only one study, with limited evidence supporting its prognostic role (28). HT and TSH levels showed conflicting associations across studies, with some suggesting potential relevance and others reporting no significant effect (25, 27, 28). Pregnancy, examined in five studies, was generally not correlated with significant tumor growth, although slight increases were observed in isolated cases (23, 29–32). However, tumor size mostly returned to baseline size or below after delivery.

Finally, prospective AS cohorts could represent the gold standard, real-world clinical setting for investigating true tumor kinetics and predictive indicators of progression and malignant behavior. Because these patients are monitored without immediate surgical intervention, tracking their changes could provide the most unconfounded clinical snapshot. However, as demonstrated by the limited, highly heterogeneous data summarized above, the current literature lacks standardized reporting and consistent multivariable analysis across AS settings. This underscores the vital importance of evaluating robust histopathological and molecular risk profiles from high-volume surgical meta-analyses to establish a reliable baseline landscape for these patients.

Studies included solely in the systematic review part are summarized in **Table 2**.

**Table 2:** Summary table of included studies in the systematic review part.

Study characteristic	Sex	Age	Size	Multifocality	Hashimoto-thyroiditis	Pregnancy	TSH
Total studies evaluating (Total number of patients with risk factor)	4 (1542)	5 (2777)	4 (1542)	1 (571)	3 (265)	5 (78)	2 (644)
Patients with risk factor	- Ge et al., (25): 21 (male) - Lee et al., (26): 177 (male) - Kwon et al., (27): 47 (male) - Nagaoka et al., (28): 76 (male)	- Ge et al., (25): 54 (< 45 years) - Lee et al., (26): 32 (< 30 years) - Ito et al., 2014 (33): 169 (< 40 years) - Kwon et al., (27): 61 (< 45 years) - Nagaoka et al., (28): 82 (< 40 years)	- Ge et al., (25): 8 (>= 7 mm) - Lee et al., (26): 382 (>= 6 mm) - Kwon et al., (27): median 5.5 (4.2–6.9) mm - Nagaoka et al., (28): 497 (>= 5 mm)	- Nagaoka et al., (28): 114	- Ge et al., (25): 18 - Kwon et al., (27): 42	- Rosario et al., (30): 5 - Ito et al., 2016 (29): 50 - Shindo et al., 2014 (31): 9 - Wen Liu et al., (35): 9 - Ghirri et al., (18): 5	- Ge et al., (25): 43 - Nagaoka et al., (28): 107 (>= 2.5 mIU/L)
Follow-up time	- Ge et al., (25): median 33 month - Lee et al., (26): mean 38.8 +/- 18.4 month - Kwon et al., (27): median 30.1 month (IQR, 21.4 - 43.7) - Nagaoka et al., (28): 7.6 +/- 5.0 years (mean)	- Ge et al., (25): median 33 month - Lee et al., (26): mean 38.8 +/- 18.4 month - Ito et al., 2014 (33): mean 68 month - Kwon et al., (27): median 30.1 month (IQR, 21.4 - 43.7) - Nagaoka et al., (28): 7.6 +/- 5.0 years (mean)	- Ge et al., (25): median 33 month - Lee et al., (26): mean 38.8 +/- 18.4 month - Kwon et al., (27): median 30.1 month (IQR, 21.4 - 43.7) - Nagaoka et al., (28): 7.6 +/- 5.0 years (mean)	- Nagaoka et al., (28): 7.6 +/- 5.0 years (mean)	- Ge et al., (25): median 33 month - Kwon et al., (27): median 30.1 month (IQR, 21.4 - 43.7)	- Rosario et al., (30): during pregnancy + 6 month after delivery - Ito et al., 2016 (29): during pregnancy (between 1993-2013) - Shindo et al., 2014 (31): during pregnancy (2005–2011) - Wen Liu et al., (35): during pregnancy - Ghirri et al., (18): 70 months (34–86 months, IQR 39.5-82.0)	- Ge et al., (25): median 33 month - Nagaoka et al., (28): 7.6 +/- 5.0 years (mean)
Definition of outcome	- Ge et al., (25): > 3 mm size increase and/or novel lymph node metastasis - Lee et al., (26): > 3 mm size increase and/or novel lymph node metastasis (or >2 mm in two dimension, or extrathyroidal extension or all four composite outcome) - Kwon et al., (27): > 3 mm size increase - Nagaoka et al., (28): > 3 mm size increase and/or novel lymph node metastasis	- Ge et al., (25): > 3 mm size increase and/or novel lymph node metastasis - Lee et al., (26): > 3 mm size increase and/or novel lymph node metastasis (or >2 mm in two dimension, or extrathyroidal extension or all four composite outcome) - Ito et al., 2014 (33): > 3 mm size increase - Kwon et al., (27): > 3 mm size increase - Nagaoka et al., (28): > 3 mm size increase and/or novel lymph node metastasis	- Ge et al., (25): > 3 mm size increase and/or novel lymph node metastasis - Lee et al., (26): > 3 mm size increase and/or novel lymph node metastasis (or >2 mm in two dimension, or extrathyroidal extension or all four composite outcome) - Kwon et al., (27): > 3 mm size increase - Nagaoka et al., (28): > 3 mm size increase and/or novel lymph node metastasis	- Nagaoka et al., (28): > 3 mm size increase	- Ge et al., (25): > 3 mm size increase and/or novel lymph node metastasis - Kwon et al., (27): > 3 mm size increase	- Rosario et al., (30): >= 3 mm size increase - Ito et al., 2016 (29): >= 3 mm size increase - Shindo et al., 2014 (31): >= 3 mm size increase - Wen Liu et al., (35): >= 3 mm size increase - Ghirri et al., (18): >=3 mm size increase	- Ge et al., (25): > 3 mm size increase and/or novel lymph node metastasis - Nagaoka et al., (28): > 3 mm size increase and/or novel lymph node metastasis
Progression during the study	- Ge et al., (25): 16/73 - Lee et al., (26): 49/706 - Kwon et al., (27): 27/192 - Nagaoka et al., (28): 59/571	- Ge et al., (25): 16/73 - Lee et al., (26): 49/706 - Ito et al., 2014 (33): 58/1235 - Kwon et al., (27): 27/192 - Nagaoka et al., (28): 59/571	- Ge et al., (25): 16/73 - Lee et al., (26): 49/706 - Kwon et al., (27): 27/192 - Nagaoka et al., (28): 59/571	- Nagaoka et al., (28): 53/571	- Ge et al., (25): 16/73 - Kwon et al., (27): 27/192	- Rosario et al., (30): 0 - Ito et al., 2016 (29): 4 - Shindo et al., 2014 (31): 7/36 - Wen Liu et al., (35): 0 - Ghirri et al., (18): 0	- Ge et al., (25): 16/73 - Nagaoka et al., (28): 59/571
Progression with the risk factor	- Ge et al., (25): 5 - Lee et al., (26): 4 - Kwon et al., (27): 5 - Nagaoka et al., (28): 2	- Ge et al., (25): 15 - Lee et al., (26): 5 - Ito et al., 2014 (33): 14 - Kwon et al., (27): 11 - Nagaoka et al., (28): 12	- Ge et al., (25): 5 - Lee et al., (26): 36 - Kwon et al., (27): median 4.5 (3.5–5.8) mm - Nagaoka et al., (28): 54	- Nagaoka et al., (28): 15	- Ge et al., (25): 8 - Kwon et al., (27): 6	- Rosario et al., (30): 0 - Ito et al., 2016 (29): 4 - Shindo et al., 2014 (31): 4 - Wen Liu et al., (35): 0 (missing data) - Ghirri et al., (18): 0	- Ge et al., (25): 7 - Nagaoka et al., (28): 11
Showed prognostic significance	- Ge et al., (25): n.a. - Lee et al., (26): yes - Kwon et al., (27): n.a. - Nagaoka et al., (28): n.a.	- Ge et al., (25): no - Lee et al., (26): yes - Ito et al., 2014 (33): yes - Kwon et al., (27): n.a. - Nagaoka et al., (28): no	- Ge et al., (25): yes - Lee et al., (26): yes - Kwon et al., (27): n.a. - Nagaoka et al., (28): n.a.	- Nagaoka et al., (28): n.a.	- Ge et al., (25): yes - Kwon et al., (27): n.a.	- Rosario et al., (30): no - Ito et al., 2016 (29): no - Shindo et al., 2014 (31): no - Wen Liu et al., (35): no - Ghirri et al., (18): no	- Ge et al., (25): n.a. - Nagaoka et al., (28): no
Multivariable analysis	- Ge et al., (25): no - Lee et al., (26): yes - Kwon et al., (27): no - Nagaoka et al., (28): no	- Ge et al., (25): yes - Lee et al., (26): yes - Ito et al., 2014 (33): yes - Kwon et al., (27): no - Nagaoka et al., (28): yes	- Ge et al., (25): yes - Lee et al., (26): yes - Kwon et al., (27): no - Nagaoka et al., (28): no	- Nagaoka et al., (28): no (just with the composite outcome)	- Ge et al., (25): yes - Kwon et al., (27): no	- Rosario et al., (30): no - Ito et al., 2016 (29): no - Shindo et al., 2014 (31): no - Wen Liu et al., (35): no - Ghirri et al., (18): no	- Ge et al., (25): no - Nagaoka et al., (28): yes
Direction of effect	- Ge et al., (25): n.a. - Lee et al., (26): male sex increase the odds - Kwon et al., (27): n.a. - Nagaoka et al., (28): n.a.	- Ge et al., (25): n.a. - Lee et al., (26): < 30 years increase the odds - Ito et al., 2014 (33): < 40 years increase the odds - Kwon et al., (27): n.a. - Nagaoka et al., (28): n.a.	- Ge et al., (25): >= 7 mm is increasing the odds - Lee et al., (26): >= 6 mm is increasing the odds - Kwon et al., (27): n.a. - Nagaoka et al., (28): n.a.	- Nagaoka et al., (28): n.a. (with the composite outcome is nonsignificant)	- Ge et al., (25): Hashimoto-thyroiditis increase the odds - Kwon et al., (27): n.a.	- Rosario et al., (30): n.a. - Ito et al., 2016 (29): n.a. - Shindo et al., 2014 (31): n.a. - Wen Liu et al., (35): n.a. - Ghirri et al., (18): n.a.	- Ge et al., (25): n.a. - Nagaoka et al., (28): nonsignificant
Effect size	- Ge et al., (25): n.a. - Lee et al., (26): OR 2.91 with 95% CI (1.59-5.33) - Kwon et al., (27): n.a. - Nagaoka et al., (28): n.a.	- Ge et al., (25): OR 2.744 with 95% CI (0.721-10.462) - Lee et al., (26): OR 3.48 with 95% CI (1.22-9.87) - Ito et al., 2014 (33): OR 2.50 with 95% CI (1.357-4.608) - Kwon et al., (27): n.a. - Nagaoka et al., (28): n.a.	- Ge et al., (25): OR 6.196 with 95% CI (1.152-33.333) - Lee et al., (26): OR 2.16 with 95% CI (1.12-4.18) - Kwon et al., (27): n.a. - Nagaoka et al., (28): n.a.	- Nagaoka et al., (28): n.a. (nonsignificant with the composite outcome)	- Ge et al., (25): OR 4.311 with 95% CI (1.154-16.110) - Kwon et al., (27): n.a.	- Rosario et al., (30): n.a. - Ito et al., 2016 (29): n.a. - Shindo et al., 2014 (31): n.a. - Wen Liu et al., (35): n.a. - Ghirri et al., (18): n.a.	- Ge et al., (25): n.a. - Nagaoka et al., (28): n.a. (nonsignificant)
Risk of bias	- Ge et al., (25): high - Lee et al., (26): high - Kwon et al., (27): high - Nagaoka et al., (28): high	- Ge et al., (25): high - Lee et al., (26): high - Ito et al., 2014 (33): moderate - Kwon et al., (27): high - Nagaoka et al., (28): high	- Ge et al., (25): high - Lee et al., (26): high - Kwon et al., (27): high - Nagaoka et al., (28): high	- Nagaoka et al., (28): high	- Ge et al., (25): high - Kwon et al., (27): high	- Rosario et al., (30): high - Ito et al., 2016 (29): high - Shindo et al., 2014 (31): high - Wen Liu et al., (35): high - Ghirri et al., (18): high	- Ge et al., (25): high - Nagaoka et al., (28): high

### 3.6 Risk of bias assessment

**Supplementary Table 5** presents the results of the risk of bias assessment.

Given the nature of the study, we applied the QUIPS (Quality in Prognostic Studies) tools in our meta-analysis. During the evaluation, studies were assessed according to our self-developed guidelines (**Supplementary Material 3d**). Many of the studies were classified as high risk (65.6%), 47 as moderate, and 43 as low risk. The study population and attrition domains were both low risk, due to the quality of the study: all patients had PTMC, and we worked with baseline raw data, making dropout negligible. Prognostic factors were independently assessed one by one following our guidelines, with those failing to meet the criteria downgraded. The study outcome domain was mostly low risk in our pool, but ratings were increased when the histology results were missing or when lymph node metastasis was reported without specifying the exact location (central or lateral). Confounding factors were evaluated; most studies did not report adjustment. In that case, the studies were downgraded. Statistical analysis and reporting domains were deemed low risk of bias across all cases.

### 3.7 Publication bias and heterogeneity

Assessments of publication bias for CLNM, LLNM, udLNM, and ETE are presented as funnel plots in **Supplementary Figure 1–52**. Based on our analysis, significant publication bias was observed for capsule invasion and CLNM, male sex and LLNM, multifocality and udLNM, and BRAF^V600E^ mutation (**Supplementary Figures 1–52**).

A leave-one-out analysis was conducted to assess the effect of the identified outliers. However, this did not result in meaningful changes to the pooled estimates; these studies were therefore retained in the final analysis.

Many articles did not report whether patients under 18 were included. As we systematically checked the baseline data, we identified different age ranges. Based on these, we performed an analysis excluding studies that may include a child population. The results received did not differ significantly from the original pool. However, we would like to mention another limitation: in cases where the mean age was provided but reporting was poor, we may have missed some of the child population (**Supplementary Figures 53–88**).

High heterogeneity was observed in approximately 90% of cases, despite most studies being conducted in Asian populations.

## 4 Discussion

Management strategies for PTMC are increasingly shifting toward more personalized care due to the indolent nature and the favorable behavior of this malignancy. However, the overall favorable prognosis of PTMC can obscure substantial biological and clinical heterogeneity, leading attending physicians to adopt a one-size-fits-all principle. While most tumors remain quiet, a small but clinically relevant subset demonstrates behavior that warrants delayed surgical intervention (34). These reports highlight that malignant tumor phenotype exists from the very beginning. Our specific goal was to identify these factors, and based on our analysis from enormous surgical cohorts, younger age (<45 and <55 years), male sex, tumor size >5 mm, multifocality, bilateral localization, and the BRAF^V600E^ mutation emerged as potential disease-modifying features.

While previous meta-analyses by Wen et al. and Luo et al. (35, 36) provided initial insights into PTMC characteristics, our analysis extends these findings by providing a more comprehensive geographical and clinical perspective. These studies were restricted to Asian cohorts and, unlike our analysis, did not include data from Western populations. Furthermore, their analysis focuses exclusively on SI or LNM; in contrast, our analysis provides more detailed outcomes. Finally, by incorporating a wider set of predictors, our study enables a more nuanced analysis. In this study, we used the Medical Subject Headings (MeSH) term papillary thyroid microcarcinoma in accordance with the current ATA guidelines (6). We acknowledge the emerging terminology of ‘subcentimeter papillary thyroid carcinoma’, but for the purpose of systematic literature retrieval and historical consistency, we employed the term PTMC. The following sections discuss these specific risk factors identified as significant indicators of an aggressive tumor phenotype.

Demographic factors such as sex and age are essential in prognostic studies. Although the incidence of PTMC is higher in women, male sex was significantly associated with increased odds of CLNM, LLNM, and udLNM; however, sex appeared neutral regarding ETE. This aligns with the observation by Nilubol et al. (37) that DTC is advanced in males, suggesting that biological sex may be a disease-modifying factor. Age is a much more complex factor. Our findings suggest a relationship between younger age and LNM. The higher prevalence of LNM in younger patients may be attributed to a more active lymphatic system compared to older populations (38). In terms of age, our research approach is also important: our inclusion criteria did not specify a minimum age. Some studies based on hospital databases or histological records did not exclude children with PTMC. Although the most common thyroid cancer in juveniles is papillary thyroid cancer, PTMC is very rare (39). Pediatric cases exhibit more aggressive disease than in adults and are more likely to develop more LNM. Based on our sensitivity analysis, their limited representation in the included studies suggests that their impact on the overall effect size is negligible. Therefore, the identified risk factors can be considered robust for adult risk stratification. Identifying a definitive age threshold is complicated by the inconsistent categorization of age intervals across studies. Despite the heterogeneity, our analysis suggests that patients <45 years have an increased risk of developing LNM, whereas those >55 years have a decreased risk. The 45-55-year range remains a clinical “gray zone”.

Pathological factors such as size, multifocality, and bilaterality are also important risk factors, as they can be identified on US. Size is a decisive characteristic for thyroid cancer, and our analysis showed an increased risk for CLNM, LLNM, udLNM, and ETE in bigger tumors in univariate analysis, but only CLNM and udLNM showed significant results in the case of pooled adjusted OR's. Different studies used various cut-off values, and the exact breakpoint remains uncertain. In our opinion, the 5-mm threshold is clinically relevant, as lesions below this size are typically not subjected to routine FNAC, reflecting their lower propensity for clinically relevant phenotypes. Smaller tumors could be more interesting in another context, particularly in histological findings after lobectomy or thyroidectomy (e.g., multinodular goiter or Graves’ disease) with the presence of an occult PTMC. Our results suggest that if the tumor is <5 mm, the probability of occult locoregional metastasis is low. On the other hand, larger tumors have elevated odds of co-existing LNM, so risk stratification and treatment based solely on size are insufficient. Both multifocality and bilaterality have a huge impact on the actual risk stratification in terms of recurrence, and their influence for advanced baseline features are also represented in our results. Hashimoto’s thyroiditis showed a significant association with CLNM and udLNM, and technically points toward a minor protective effect. We interpret these small effect sizes as clinically negligible rather than a true biological shield, as this marginal trend is largely a statistical artifact driven by our enormous sample size. In such large cohorts, the exceptionally high statistical power compresses the confidence intervals so closely to the null effect line that even a minimal 9% or 18% reduction in odds becomes statistically significant without translating into real-world clinical relevance. This view is further supported by the clear inconsistency across endpoints: HT fails to show a reliable protective signal for the other outcomes, indicating that its clinical impact is insignificant. Moreover, the pooled adjusted OR also failed to show a significant result. Based on these, our conclusion is that HT is a non-actionable autoimmune condition, whether its statistical effect is marginally protective or neutral is clinically inconsequential, as it cannot guide management or counterpoise dominant risk factors like young age or BRAF mutations without validated multiparametric scoring systems.

Molecular markers, such as the BRAF^V600E^ mutation, may represent a potential disease-modifying factor in PTMC. As outlined in the ATA 2025 guidelines, higher-risk tumors are more likely to harbor BRAF^V600E^ mutations than low-risk tumors, yet low-risk tumors still do so at a high frequency. It is possible that a higher allelic frequency of the BRAF^V600E^ mutation is associated with more aggressive tumor behavior (6). In our cohort, those with the mutation had elevated odds of developing CLNM, LNM, and ETE in univariate analysis and developing CLNM in multivariate calculations. Surprisingly, lateral compartment metastasis did not reach statistical significance. More than 50% of the observed patients had the mutation; the high prevalence may have influenced the result. Moreover, variability in the prognostic value of BRAF across different studies may also reflect differences in detection sensitivity. While older methodologies, such as Sanger sequencing, require a high tumor-to-normal cell ratio, more sensitive techniques, such as Next-Generation Sequencing or digital PCR, are capable of detecting the mutation at a much lower variant allele frequency. These, taken together, limit the role of BRAF as a standalone prognosticator. Our findings support the emerging consensus that BRAF status should be integrated into a multi-factorial risk assessment rather than used as a binary decision-making tool.

Our analysis indicates that, while microcalcification and tumor capsule invasion are potent predictors of LNM in PTMC, reflecting a more aggressive tumor biology, other clinical factors, such as obesity, multinodular goiter, Graves’ disease, and TSH levels, showed no significant association with nodal spread. Although HT has been historically debated as a risk factor, our findings confirm its neutral role regarding malignant features, potentially due to a protective immune microenvironment. Furthermore, neither anti-TG antibodies nor TERT promoter mutations reached statistical significance in relation to LNM in our cohort, suggesting that the metastatic cascade in microcarcinomas may occur independently of these traditional prognostic markers.

Pregnancy is a temporary condition that primarily affects young adults and represents a vulnerable state because the treatment options are limited during gestation. According to the included prospective studies, pregnancy does not represent an absolute indication for therapeutic failure or mandatory surgical intervention (**Table 2**). In some cases, a reduction in size was also observed, and the authors continued the noninvasive management.

The pooling of adjusted risk estimates has some limitations, such as the fact that the majority of the articles did not report any confounding factors, so an exact calculation was not possible. Consideration of these results is a must, and the clinical interpretation should be approached with balanced caution.

Although our meta-analysis identified robust predictors for aggressive PTMC phenotype, the observed heterogeneity across the included studies warrants careful interpretation. Geographical differences in iodine intake may contribute to heterogeneity between the studies, as iodine status influences thyroid function and disease behavior (40, 41). In addition, histological subtypes were not analyzed separately, as this information was inconsistently reported across studies. Although certain variants are known to exhibit more aggressive behavior, their exclusion reflects data availability rather than methodological oversight. As noted above, the inclusion of pediatric cases and molecular methodologies may also have partially influenced the results. Variability in outcome definitions, particularly regarding lymph node metastasis and extrathyroidal extension, represents another potential source of heterogeneity across studies. During the analysis, occasional outliers were identified, which did not alter the direction of the pooled estimates. In this context, heterogeneity should be considered as an expected feature of PTMC research, reflecting real-world biological behavior.

### 4.1 Strengths and limitations

We followed our registered protocol and applied a rigorous methodology. Among the main strengths of our analysis are the broad search strategy and the large sample size, with data collected from more than 200,000 patients, including those from multicenter studies. Although substantial heterogeneity was observed, this reflects the clinical heterogeneity of PTMC rather than methodological inconsistency. Capturing this variability is a strength of the present study.

Several limitations should also be acknowledged. First, the definitions of some risk factors varied across the articles, which contributed to many studies being classified as having a high RoB. Second, in retrospective studies, a unified research setting was missing. Finally, the incoherent adjusted data from multivariable analysis prevents the ability to draw complex conclusions.

### 4.2 Implications for practice and research

Translating scientific findings into practice is crucial (42); therefore, we emphasize the importance of systematic thinking and the development of a risk calculation model. Individual patients may have multiple risk factors for progression, underscoring the need for careful consideration by policymakers. Further prospective data collection, along with multivariable analysis, is necessary to more accurately assess the problem at hand. We emphasize the importance for international collaboration in the collection of detailed data.

## 5 Conclusion

In conclusion, male sex, younger age, multifocality, bilateral localization, and the BRAF^V600E^ mutation are also associated with a higher risk of LNM or ETE. Risk assessment of PTMCs based solely on size, even if it is a risk factor, is insufficient to determine subsequent clinical steps. Future management strategies must transition toward a multi-parametric baseline approach to guide personalized care.

## Data Availability

The data analyzed in this study is subject to the following licenses/restrictions: No restrictions apply to the dataset. The data used in this study are available from the corresponding author upon reasonable request. Requests to access these datasets should be directed to MO, org.mate21@gmail.com.
