# Supplementary material for: Risk factors associated with aggressive tumor phenotypes in papillary thyroid microcarcinoma: a systematic review and meta-analysis
Source: Front Endocrinol (Lausanne). 2026 Jul 2;17:1876912. doi: 10.3389/fendo.2026.1876912 (PMC13372712; doi:10.3389/fendo.2026.1876912)
Supplement: Supplementary file 1 [file DataSheet1.docx]

Supplementary Material

**SUPPLEMENTARY MATERIAL**

1. PRISMA 2020 CHECKLIST – Supplementary Table 1.

2. METHODS

a. Applied search key

b. Data collection process

3. RESULTS

a. Supplementary tables for basic characteristics – Supplementary Table 2-3.

b. Supplementary results – figures and plots – Supplementary Figure 1-88.

c. Metaregression – Supplementary Table 4.

d. Risk of bias – Supplementary Table 5.

4. REFERENC

1. PRISMA 2020 CHECKLIST

**Supplementary Table 1.** | PRISMA 2020 checklist

| **Section and Topic** | **Item #** | **Checklist item** | **Location where item is reported** |
| --- | --- | --- | --- |
| **TITLE** | | |  |
| Title | 1 | Identify the report as a systematic review. | Title |
| **ABSTRACT** | | |  |
| Abstract | 2 | See the PRISMA 2020 for Abstracts checklist. | Abstract |
| **INTRODUCTION** | | |  |
| Rationale | 3 | Describe the rationale for the review in the context of existing knowledge. | Introduction |
| Objectives | 4 | Provide an explicit statement of the objective(s) or question(s) the review addresses. | Introduction |
| **METHODS** | | |  |
| Eligibility criteria | 5 | Specify the inclusion and exclusion criteria for the review and how studies were grouped for the syntheses. | Methods – Eligibility criteria |
| Information sources | 6 | Specify all databases, registers, websites, organisations, reference lists and other sources searched or consulted to identify studies. Specify the date when each source was last searched or consulted. | Methods – Information sources |
| Search strategy | 7 | Present the full search strategies for all databases, registers and websites, including any filters and limits used. | Methods – Search strategy |
| Selection process | 8 | Specify the methods used to decide whether a study met the inclusion criteria of the review, including how many reviewers screened each record and each report retrieved, whether they worked independently, and if applicable, details of automation tools used in the process. | Methods – Selection process |
| Data collection process | 9 | Specify the methods used to collect data from reports, including how many reviewers collected data from each report, whether they worked independently, any processes for obtaining or confirming data from study investigators, and if applicable, details of automation tools used in the process. | Methods – Data collection process |
| Data items | 10a | List and define all outcomes for which data were sought. Specify whether all results that were compatible with each outcome domain in each study were sought (e.g. for all measures, time points, analyses), and if not, the methods used to decide which results to collect. | Methods – Data items |
|  | 10b | List and define all other variables for which data were sought (e.g. participant and intervention characteristics, funding sources). Describe any assumptions made about any missing or unclear information. | Methods – Data items |
| Study risk of bias assessment | 11 | Specify the methods used to assess risk of bias in the included studies, including details of the tool(s) used, how many reviewers assessed each study and whether they worked independently, and if applicable, details of automation tools used in the process. | Methods – Study risk of bias assessment |
| Effect measures | 12 | Specify for each outcome the effect measure(s) (e.g. risk ratio, mean difference) used in the synthesis or presentation of results. | Methods – Synthesis methods |
| Synthesis methods | 13a | Describe the processes used to decide which studies were eligible for each synthesis (e.g. tabulating the study intervention characteristics and comparing against the planned groups for each synthesis (item #5)). | Methods – Synthesis methods |
|  | 13b | Describe any methods required to prepare the data for presentation or synthesis, such as handling of missing summary statistics, or data conversions. | Methods – Synthesis methods |
|  | 13c | Describe any methods used to tabulate or visually display results of individual studies and syntheses. | Methods – Synthesis methods |
|  | 13d | Describe any methods used to synthesize results and provide a rationale for the choice(s). If meta-analysis was performed, describe the model(s), method(s) to identify the presence and extent of statistical heterogeneity, and software package(s) used. | Methods – Synthesis methods |
|  | 13e | Describe any methods used to explore possible causes of heterogeneity among study results (e.g. subgroup analysis, meta-regression). | Methods – Synthesis methods |
|  | 13f | Describe any sensitivity analyses conducted to assess robustness of the synthesized results. | Results – Publication bias and heterogeneity |
| Reporting bias assessment | 14 | Describe any methods used to assess risk of bias due to missing results in a synthesis (arising from reporting biases). | Results – Publication bias and heterogeneity |
| Certainty assessment | 15 | Describe any methods used to assess certainty (or confidence) in the body of evidence for an outcome. | n.a. |
| **RESULTS** | | |  |
| Study selection | 16a | Describe the results of the search and selection process, from the number of records identified in the search to the number of studies included in the review, ideally using a flow diagram. | Results – Search and selection |
|  | 16b | Cite studies that might appear to meet the inclusion criteria, but which were excluded, and explain why they were excluded. | n.a. |
| Study characteristics | 17 | Cite each included study and present its characteristics. | Supplementary material |
| Risk of bias in studies | 18 | Present assessments of risk of bias for each included study. | Supplementary material |
| Results of individual studies | 19 | For all outcomes, present, for each study: (a) summary statistics for each group (where appropriate) and (b) an effect estimate and its precision (e.g. confidence/credible interval), ideally using structured tables or plots. | Results 1-3. and Supplementary material |
| Results of syntheses | 20a | For each synthesis, briefly summarise the characteristics and risk of bias among contributing studies. | Risk of bias assessment |
|  | 20b | Present results of all statistical syntheses conducted. If meta-analysis was done, present for each the summary estimate and its precision (e.g. confidence/credible interval) and measures of statistical heterogeneity. If comparing groups, describe the direction of the effect. | Results and Risk of bias assessment and Publication bias and heterogeneity |
|  | 20c | Present results of all investigations of possible causes of heterogeneity among study results. | Publication bias and heterogeneity and Supplementary material |
|  | 20d | Present results of all sensitivity analyses conducted to assess the robustness of the synthesized results. | Publication bias and heterogeneity |
| Reporting biases | 21 | Present assessments of risk of bias due to missing results (arising from reporting biases) for each synthesis assessed. | Supplementary material |
| Certainty of evidence | 22 | Present assessments of certainty (or confidence) in the body of evidence for each outcome assessed. | n.a. |
| **DISCUSSION** | | |  |
| Discussion | 23a | Provide a general interpretation of the results in the context of other evidence. | Discussion |
|  | 23b | Discuss any limitations of the evidence included in the review. | Discussion |
|  | 23c | Discuss any limitations of the review processes used. | Discussion |
|  | 23d | Discuss implications of the results for practice, policy, and future research. | Discussion |
| **OTHER INFORMATION** | | |  |
| Registration and protocol | 24a | Provide registration information for the review, including register name and registration number, or state that the review was not registered. | Methods – Information sources |
|  | 24b | Indicate where the review protocol can be accessed, or state that a protocol was not prepared. | Methods – Information sources |
|  | 24c | Describe and explain any amendments to information provided at registration or in the protocol. | Methods – Information sources |
| Support | 25 | Describe sources of financial or non-financial support for the review, and the role of the funders or sponsors in the review. | Funding |
| Competing interests | 26 | Declare any competing interests of review authors. | Conflict of interest |
| Availability of data, code and other materials | 27 | Report which of the following are publicly available and where they can be found: template data collection forms; data extracted from included studies; data used for all analyses; analytic code; any other materials used in the review. | Methods |

*From:*  Page MJ, McKenzie JE, Bossuyt PM, Boutron I, Hoffmann TC, Mulrow CD, et al. The PRISMA 2020 statement: an updated guideline for reporting systematic reviews. BMJ 2021;372:n71. doi: 10.1136/bmj.n71. This work is licensed under CC BY 4.0. To view a copy of this license, visit <https://creativecommons.org/licenses/by/4.0/>

n.a.: *not applicable*

2. METHODS

a. Applied search key

For our search protocol, we used a search key consisting of one domain that describes the population. The search was conducted in 3 databases. Furthermore we used a citation chase method to expand our results.

**Pubmed**

((((”papillary thyroid”) **AND** ”microcarcinoma”) **OR** ”occult carcinoma”) **OR** T1aN*)

Domain 1 refers to: population

Number of results: **2364**

Notes: We used only one domain because of the few results. We used the truncation **T1aN*** to find all varieties of the word, like T1aN1 or T1aN2.

Database settings: We did not use any settings.

**Embase**

((((’papillary thyroid’) **AND** ’microcarcinoma’) **OR** ’occult carcinoma’) **OR** T1aN*)

Number of results: **3492**

Notes: Same as PubMed

Database settings: We did not use any settings.

**CENTRAL (Cochrane)**

((((”papillary thyroid”) **AND** ”microcarcinoma”) **OR** ”occult carcinoma”) **OR** T1aN*)

Domain 1 refers to: population

Number of results: **106**

Notes: Same as Pubmed

Database settings: All text

b. Data collection process

General rules:

- Sort the articles by title
- In the case of duplicates, treat them the same: either keep both or exclude both.
- If it is hard to decide based on the title whether to keep the record or not, and the abstract is not available, then first look for the abstract. If there is none of them, include it as eligible, and we will look for the abstracts online again at the next phase of the selection
- We will perform Cohen’s kappa, and after the resolution of disagreements, a new library file will be sent to you.

|  | **INCLUSION** | **EXCLUSION** |
| --- | --- | --- |
|  | **HUMAN studies** | ANIMAL , MOLECULAR studies  (eg. fish, dog, mice, rat, in vitro, genetic) |
| **Type of study** | RCT, non-randomized interventional trials, observational studies, prospective and retrospective studies. | Reviews  Systematic rewievs  Meta analysis  Guidlines  Case reports, Case series, case records  Protocols  Abstracts (any kind)  Congress/Conference books, abstract selection, reviews |
| **Population** | patients with papillary thyroid microcarcinoma AND active surveillance OR treated surgically | patients without papillary thyroid carcinoma, patients with other thyroid histology subtype/subvariant, patients who has papillary thyroid carcinoma BUT the size is > 1 cm or > 10 mm |

- Factor:
- sex: no comment
- age: during the collection several age cut-offs were registered, so based on the majority articles we conlcuded two cut-off value as 45 y.o. and 55 y.o. Based on these the patients were grouped by as mentioned.
- tumor size: also several size cut-offs were registered, so based on the majority articles we conlcuded four cut-offs.
- body mass index: we groupped the participants based on their BMI under and above 24 kg/m2
- multifocality: no comment
- bilaterality: no comment
- localization: no comment
- Hashimoto’s thyroiditis: no comment
- goiter or Graves’s disease: no comment
- genetics: no comment
- TSH: no comment
- anti-Tg: no comment
- pregnancy: no comment
- capsule: no comment
- microcalcification: no comment
- Outcome:
- Primary outcome will be tumor size increase (at least > 3 mm), novel lymph node metastasis, extrathyroidal extension
  - Note: in some cases, microscopic LNM was counted as positive LNM, and microscopic ETE was also counted as positive ETE.

3. RESULTS

a. Supplementary tables for basic characteristics

**Supplementary Table 2** | Baseline characteristics of the included studies

| **Study number** | **Study first author** | **Study type** | **Country** | **Centers** | **Population count** |
| --- | --- | --- | --- | --- | --- |
| 1 | Kim et al. 2020 | retrospective | USA, Poland, Italy, Australia, Spain, Czech Republic | 6 | 743 |
| 2 | Choi et al. 2020 | retrospective | South-Korea | 1 | 876 |
| 3 | Besic et al. 2009 | retrospective | Slovenia | 1 | 254 |
| 4 | Cai et al. 2016 | retrospective | China | 1 | 498 |
| 5 | Ding et al. 2023 | retrospective | USA | database | 35118 |
| 6 | Fu et al. 2020 | retrospective | China | 1 | 107 |
| 7 | Ping Yang et al. 2022 | retrospective | China | 1 | 564 |
| 8 | Apostol et al. 2017 | retrospective | Romania | 1 | 168 |
| 9 | Liu et al. 2014 | retrospective | China | 1 | 823 |
| 10 | Jin et al. 2023 | retrospective | China | 1 | 398 |
| 11 | Kaliszewski et al. 2020 | retrospective | Poland | 1 | 182 |
| 12 | Sohee Lee et al. 2019 | retrospective | South-Korea | 2 | 44 |
| 13 | Lin et al. 2005 | retrospective | Taiwan | 1 | 227 |
| 14 | Amendola et al. 2024 | retrospective | Italy | 3 | 136 |
| 15 | Xiang et al. 2018 | retrospective | China | 1 | 245 |
| 16 | Zhang et al. 2016 | retrospective | China | 1 | 1226 |
| 17 | Zhu et al. 2020 | retrospective | China | 1 | 1622 |
| 18 | Han et al. 2022 | retrospective | China | 1 | 172 |
| 19 | Liu et al. 2022 | retrospective | China | 1 | 5399 |
| 20 | Shi et al. 2022 | retrospective | China | 1 | 449 |
| 21 | Nam Seop Lee et al. 2010 | retrospective | South-Korea | 1 | 335 |
| 22 | Jeong et al. 2017 | retrospective | South-Korea | 1 | 575 |
| 23 | Luo et al. 2022 | retrospective | China | 1 | 546 |
| 24 | Iscan et al. 2019 | retrospective | Turkey | 1 | 306 |
| 25 | Medas et al. 2020 | retrospective | Italy | 1 | 293 |
| 26 | Ye et al. 2022 | retrospective | China | 2 | 399 |
| 27 | Wang et al. 2023 | retrospective | China | 1 | 1506 |
| 28 | Yin et al. 2021 | retrospective | China | 1 | 607 |
| 29 | Zhao et al. 2021 | retrospective | China | 1 | 179 |
| 30 | Cheng et al. 2019 | retrospective | China | 1 | 785 |
| 31 | Peng et al. 2016 | retrospective | China | 1 | 1401 |
| 32 | Zhang et al. 2018 | retrospective | China | 1 | 1304 |
| 33 | Bircan et al. 2014 | retrospective | Turkey | 2 | 172 |
| 34 | Li et al. 2017 | retrospective | China | 1 | 273 |
| 35 | Nechifor Boila et al. 2018 | retrospective | Romania | 1 | 25 |
| 36 | Liu et al. 2016 | retrospective | China | 1 | 168 |
| 37 | Zeming Liu et al. 2017 | retrospective | China | 1 | 1115 |
| 38 | Chunping Liu et al. 2017 | retrospective | China | 1 | 1106 |
| 39 | Zhang et al. 2017 | retrospective | China | 1 | 1268 |
| 40 | Zheng et al. 2020 | retrospective | China | 1 | 200 |
| 41 | Lee et al. 2014 | retrospective | South-Korea | 1 | 325 |
| 42 | Dzepina et al. 2012 | retrospective | Croatia | 1 | 318 |
| 43 | Yufei Wang et al. 2017 | retrospective | China | 1 | 169 |
| 44 | Wang et al. 2017 | retrospective | China | 1 | 150 |
| 45 | Yoon et al. 2017 | retrospective | South-Korea | 1 | 79 |
| 46 | Yan et al. 2019 | retrospective | USA | 1 | 182 |
| 47 | Dirikoc et al. 2021 | retrospective | Turkey | 1 | 1184 |
| 48 | Denghui Wang et al. 2022 | retrospective | China | 1 | 804 |
| 49 | Chang et al. 2016 | retrospective | South-Korea | 1 | 613 |
| 50 | Jun Liu et al. 2022 | retrospective | China | 1 | 171 |
| 51 | Zhao et al. 2020 | retrospective | China | 1 | 2434 |
| 52 | Yesiloglu et al. 2024 | retrospective | Turkey | 1 | 95 |
| 53 | Tam et al. 2017 | retrospective | Turkey | 1 | 166 |
| 54 | Zhao et al. 2024 | retrospective | China | 1 | 2094 |
| 55 | Yunjun Wang et al. 2018 | retrospective | China | 1 | 8668 |
| 56 | Sheng et al. 2019 | retrospective | China | 1 | 2404 |
| 57 | Xiao et al. 2021 | retrospective | China | 1 | 576 |
| 58 | Bastos et al. 2015 | retrospective | Brazil | 1 | 34 |
| 59 | Baoding Chen et al. 2019 | retrospective | China | 1 | 116 |
| 60 | Jeon et al. 2017 | retrospective | South-Korea | 1 | 395 |
| 61 | Kim et al. 2005 | retrospective | South-Korea | 1 | 60 |
| 62 | Kim et al. 2012 | retrospective | South-Korea | 1 | 490 |
| 63 | Kim et al. 2014 | retrospective | South-Korea | 1 | 109 |
| 64 | Kwak et al. 2009 | retrospective | South-Korea | 1 | 339 |
| 65 | Lee et al. 2008 | retrospective | South-Korea | 1 | 52 |
| 66 | Lim et al. 2007 | retrospective | South-Korea | 1 | 111 |
| 67 | Lim et al. 2009 | retrospective | South-Korea | 1 | 86 |
| 68 | Oh et al. 2017 | retrospective | South-Korea | 1 | 2329 |
| 69 | Park et al. 2014 | retrospective | South-Korea | 1 | 193 |
| 70 | Jin et al. 2014 | retrospective | China | 1 | 119 |
| 71 | Roti et al. 2006 | retrospective | Italy | 1 | 243 |
| 72 | Pisanu et al. 2009 | retrospective | Italy | 1 | 76 |
| 73 | Lee et al. 2011 | retrospective | South-Korea | 1 | 275 |
| 74 | Lombardi et al. 2010 | retrospective | Italy | 1 | 933 |
| 75 | Kim et al. 2013 | retrospective | South-Korea | 1 | 483 |
| 76 | Cho Rok Lee et al. 2014 | retrospective | South-Korea | 1 | 281 |
| 77 | Lee et al. 2013 | retrospective | South-Korea | 2 | 396 |
| 78 | Jeong et al. 2022 | retrospective | South-Korea | 1 | 506 |
| 79 | Cui Zhang et al. 2020 | retrospective | China | 1 | 553 |
| 80 | Hui Huang II et al. 2023 | retrospective | China | 1 | 9929 |
| 81 | Lee et al. 2009 | retrospective | China | 1 | 64 |
| 82 | Koo et al. 2010 | retrospective | South-Korea | 1 | 132 |
| 83 | Lin et al. 2010 | retrospective | China | 1 | 61 |
| 84 | Moon et al. 2011 | retrospective | South-Korea | 1 | 288 |
| 85 | Hyun et al. 2012 | retrospective | South-Korea | 1 | 152 |
| 86 | Zhao et al. 2013 | retrospective | China | 1 | 212 |
| 87 | Zheng et al. 2013 | retrospective | China | 1 | 977 |
| 88 | Kim et al. 2016 | retrospective | South-Korea | 1 | 5137 |
| 89 | Yi Ho Lee et al. 2017 | retrospective | South-Korea | 1 | 1179 |
| 90 | Back et al. 2019 | retrospective | South-Korea | 1 | 2967 |
| 91 | Shin et al. 2014 | retrospective | South-Korea | 1 | 588 |
| 92 | Choi et al. 2015 | retrospective | South-Korea | 1 | 612 |
| 93 | Pisanu et al. 2015 | retrospective | Italy | 1 | 219 |
| 94 | Kwangsoon Kim et al. 2020 | retrospective | South-Korea | 1 | 3578 |
| 95 | Zuhur et al. 2024 | retrospective | Turkey | n.a. | 1009 |
| 96 | Jingjia Cao et al. 2024 | retrospective | China | 1 | 322 |
| 97 | Kim et al. 2010 | retrospective | South-Korea | 1 | 323 |
| 98 | Zheng et al. 2012 | retrospective | China | 1 | 176 |
| 99 | Qu et al. 2016 | retrospective | China | 1 | 1250 |
| 100 | Gui et al. 2018 | retrospective | China | 1 | 541 |
| 101 | Ahn et al. 2014 | retrospective | South-Korea | 1 | 348 |
| 102 | Feng et al. 2020 | retrospective | China | 2 | 371 |
| 103 | Lee et al. 2021 | retrospective | South-Korea | 1 | 497 |
| 104 | Saaduddin et al. 2016 | retrospective | USA | 1 | 163 |
| 105 | Varshney et al. 2014 | retrospective | Canada | 1 | 170 |
| 106 | Vasileiadis et al. 2011 | retrospective | Greece | 1 | 276 |
| 107 | Wang et al. 2016 | retrospective | China | 1 | 1204 |
| 108 | Wu et al. 2018 | retrospective | China | 1 | 2129 |
| 109 | Xiang et al. 2014 | retrospective | China | 1 | 949 |
| 110 | Xu et al. 2018 | retrospective | China | 1 | 3607 |
| 111 | Yan et al. 2021 | retrospective | China | 1 | 3005 |
| 112 | Yu et al. 2017 | retrospective | China | 1 | 917 |
| 113 | Yuan et al. 2017 | retrospective | China | 1 | 295 |
| 114 | Zeng et al. 2013 | retrospective | China | 1 | 141 |
| 115 | Zhang et al. 2013 | retrospective | China | 1 | 1066 |
| 116 | Zheng et al. 2019 | retrospective | China | 1 | 246 |
| 117 | Zhao et al. 2017 | retrospective | China | 1 | 521 |
| 118 | Gao et al. 2025 | retrospective | China | 1 | 87 |
| 119 | Ozemir et al. 2023 | retrospective | Turkey | 1 | 161 |
| 120 | Goran et al. 2017 | retrospective | Serbia | 1 | 111 |
| 121 | Goran et al. 2019 | retrospective | Serbia | 1 | 257 |
| 122 | Meilinger-Dobra et al. 2018 | retrospective | Hungary | 1 | 103 |
| 123 | Zhao et al. 2019 | retrospective | China | 1 | 215 |
| 124 | Zhou et al. 2012 | retrospective | China | 1 | 122 |
| 125 | Choi et al. 2013 | retrospective | South-Korea | 1 | 101 |
| 126 | Seifert et al. 2021 | retrospective | Germany | n.a. | 216 |
| 127 | Kayhan et al. 2024 | retrospective | Turkey | 1 | 255 |
| 128 | Zhang Zhu et al. 2024 | retrospective | China | 1 | 188 |
| 129 | Zhi Zhao et al. 2018 | retrospective | China | 1 | 1007 |
| 130 | Wada et al. 2003 | retrospective | Japan | 1 | 189 |
| 131 | Chen et al. 2019 | retrospective | China | 1 | 182 |
| 132 | Lu et al. 2020 | retrospective | China | 1 | 1031 |
| 133 | Oh et al. 2013 | retrospective | South-Korea | 1 | 379 |
| 134 | Yu et al. 2022 | retrospective | China | 1 | 1121 |
| 135 | Xiaojuan Zheng et al. 2020 | retrospective | China | 1 | 97 |
| 136 | Chow et al. 2003 | retrospective | China | 1 | 203 |
| 137 | Rossi et al. 2012 | retrospective | Italy | 1 | 50 |
| 138 | Tagliabue et al. 2021 | retrospective | Italy | 1 | 195 |
| 139 | Xiangqian Zheng et al. 2019 | retrospective | China | 1 | 1587 |
| 140 | Wei et al. 2019 | retrospective | China | 1 | 710 |
| 141 | Lee et al. 2017 | retrospective | South-Korea | 1 | 332 |
| 142 | Seo et al. 2016 | retrospective | South-Korea | 1 | 74 |
| 143 | Song Yan et al. 2020 | retrospective | China | 1 | 3686 |
| 144 | Yang et al. 2014 | retrospective | China | 1 | 291 |
| 145 | Ren et al. 2018 | retrospective | China | 1 | 133 |
| 146 | Zheng et al. 2018 | retrospective | China | 1 | 3543 |
| 147 | Takahito et al. 2024 | retrospective | Japan | 1 | 51 |
| 148 | Chen et al. 2023 | retrospective | China | 1 | 103 |
| 149 | Kim et al. 2021 | retrospective | South-Korea | 1 | 27 |
| 150 | Lim et al. 2024 | retrospective | South-Korea | 2 | 783 |
| 151 | Xue et al. 2019 | retrospective | China | 1 | 252 |
| 152 | Sun et al. 2021 | retrospective | China | 1 | 552 |
| 153 | Chunwang et al. 2021 | retrospective | China | 1 | 220 |
| 154 | Ma et al. 2023 | retrospective | China | 1 | 139 |
| 155 | Li et al. 2023 | retrospective | China | 1 | 102 |
| 156 | Zhang et al. 2023 | retrospective | China | 1 | 159 |
| 157 | Huang et al. 2024 | retrospective | China | 1 | 907 |
| 158 | Lai et al. 2016 | retrospective | China | 1 | 367 |
| 159 | Kim et al. 2017 | retrospective | South-Korea | 1 | 361 |
| 160 | Wenlong Wan et al. 2020 | retrospective | China | 1 | 142 |
| 161 | Cao et al. 2022 | retrospective | China | 1 | 33 |
| 162 | Zhu et al. 2022 | retrospective | China | 1 | 102 |
| 163 | Wu et al. 2020 | retrospective | China | 1 | 115 |
| 164 | Wang et al. 2020 | retrospective | China | 1 | 114 |
| 165 | Zhao et al. 2023 | retrospective | China | 1 | 55 |
| 166 | Karatzas et al. 2013 | retrospective | Greece | 1 | 319 |
| 167 | Zeming et al. 20156 | retrospective | China | 1 | 501 |
| 168 | Dequan Xu et al. 2014 | retrospective | China | 1 | 402 |
| 169 | He et al. 2017 | retrospective | China | 4 | 374 |
| 170 | Ji et al. 2019 | retrospective | China | 1 | 89 |
| 171 | Liu Wang et al. 2021 | retrospective | China | 1 | 687 |
| 172 | Lai et al. 2023 | retrospective | China | 1 | 250 |
| 173 | Hitu et al. 2021 | retrospective | Romania | 1 | 82 |
| 174 | Parvathareddy et al. 2022 | retrospective | Saudi Arabia | 1 | 202 |
| 175 | Yang et al. 2022 | retrospective | China | 3 | 717 |
| 176 | Cho et al. 2012 | retrospective | South-Korea | 1 | 209 |
| 177 | Kemin et al. 2021 | retrospective | China | 1 | 185 |
| 178 | Sun et al. 2024 | retrospective | China | 1 | 253 |
| 179 | Jiwang et al. 2022 | retrospective | China | 1 | 599 |
| 180 | Tao et al. 2016 | retrospective | China | 1 | 66 |
| 181 | Park et al. 2015 | retrospective | South-Korea | 1 | 460 |
| 182 | Xu et al. 2016 | retrospective | China | 1 | 252 |
| 183 | Zheng Liu et al. 2017 | retrospective | China | 1 | 366 |
| 184 | Wu et al. 2019 | retrospective | China | 1 | 936 |
| 185 | Wang et al. 2018 | retrospective | China | 1 | 216 |
| 186 | Sezer et al. 2020 | retrospective | Turkey | 1 | 72 |
| 187 | Lu et al. 2017 | retrospective | China | 1 | 94 |
| 188 | Zhou et al. 2019 | retrospective | China | 1 | 162 |
| 189 | Dong et al. 2021 | retrospective | China | 1 | 313 |
| 190 | Wang et al. 2024 | retrospective | China | 1 | 611 |
| 191 | Xue et al. 2024 | retrospective | China | 1 | 106 |
| 192 | Virk et al. 2013 | retrospective | USA | 1 | 87 |
| 193 | Shen et al. 2022 | retrospective | China | 1 | 1433 |
| 194 | Jin et al. 2018 | retrospective | China | 1 | 673 |
| 195 | Zhang et al. 2015 | retrospective | China | 1 | 178 |
| 196 | Ayesha et al. 2023 | retrospective | India | 1 | 48 |
| 197 | Bradley et al. 2017 | retrospective | Canada | 1 | 132 |
| 198 | Huang et al. 2023 | retrospective | China | 1 | 4872 |
| 199 | Qiu et al. 2024 | retrospective | China | 1 | 377 |
| 200 | Gu et al. 2019 | retrospective | China | 1 | 268 |
| 201 | Xie et al. 2023 | retrospective | China | 1 | 170 |
| 202 | Yin et al. 2017 | retrospective | China | 1 | 1092 |
| 203 | Song et al. 2022 | retrospective | South-Korea | 1 | 814 |
| 204 | Cao et al. 2024 | retrospective | China | 1 | 20 |
| 205 | Hong et al. 2015 | retrospective | China | 1 | 127 |
| 206 | Bo Yeon Kim et al. 2012 | retrospective | South-Korea | 1 | 160 |
| 207 | Pan et al. 2018 | retrospective | China | 1 | 187 |
| 208 | Zhang et al. 2022 | retrospective | China | 1 | 3128 |
| 209 | Besic et al. 2008 | retrospective | Slovenia | 1 | 228 |
| 210 | Li Xiaojing et al. 2017 | retrospective | China | 1 | 1988 |
| 211 | Xu et al. 2014 | retrospective | China | 1 | 85 |
| 212 | Xia et al. 2011 | retrospective | China | 1 | 286 |
| 213 | Ma et al. 2021 | retrospective | China | 1 | 80 |
| 214 | Korkmaz et al. 2016 | retrospective | Turkey | 1 | 228 |
| 215 | Chang Liu et al. 2021 | retrospective | China | 1 | 556 |
| 216 | Pardo et al. 2020 | retrospective | Spain | 1 | 161 |
| 217 | Bian et al. 2015 | retrospective | China | 1 | 1037 |
| 218 | Chen et al. 2016 | retrospective | China | 1 | 487 |
| 219 | Jiang et al. 2018 | retrospective | China | 1 | 3132 |
| 220 | Yu et al. 2018 | retrospective | China | 1 | 186 |
| 221 | Zhang Haidong et al. 2023 | retrospective | China | 1 | 154 |
| 222 | Wang Zhaohui et al. 2019 | retrospective | China | 1 | 700 |
| 223 | Wu et al. 2017 | retrospective | China | 1 | 707 |
| 224 | Wang et al. 2022 | retrospective | China | 1 | 72 |
| 225 | Wang et al. 2019 | retrospective | China | 1 | 385 |
| 226 | Tang et al. 2024 | retrospective | China | 1 | 102 |
| 227 | Mei et al. 2023 | retrospective | China | 1 | 230 |
| 228 | Akgun et al. 2023 | retrospective | Turkey | 1 | 178 |
| 229 | Yan et al. 2022 | retrospective | China | 1 | 383 |
| 230 | Caliskan et al. 2012 | retrospective | South-Korea | 1 | 842 |
| 231 | Gweon et al. 2017 | retrospective | South-Korea | 1 | 1041 |
| 232 | Hassan et al. 2023 | retrospective | Egypt | 1 | 9744 |
| 233 | Hui Huang et al. 2023 | retrospective | China | 1 | 4872 |
| 234 | Yoon et al. 2024 | retrospective | South-Korea | 1 | 358 |
| 235 | Jinqiu Wang et al. 2022 | retrospective | China | 1 | 500 |
| 236 | Jinqiu Wang et al. 2025 | retrospective | China | 1 | 81 |
| 237 | Kaliszewski et al. 2019 | retrospective | Poland | 1 | 177 |
| 238 | Yong Wan Kim et al. 2009 | retrospective | South-Korea | 1 | 161 |
| 239 | Won Jin Kim et al. 2013 | retrospective | South-Korea | 1 | 820 |
| 240 | Ju Yeon Kim et al. 2015 | retrospective | South-Korea | 1 | 428 |
| 241 | Young Hun Kim et a 2016 | retrospective | South-Korea | 1 | 622 |
| 242 | Hyeung Kyoo Kim et al. 2025 | retrospective | South-Korea | 1 | 3004 |
| 243 | Jae Bok Lee et al. 2008 | retrospective | South-Korea | 1 | 165 |
| 244 | Sung Min Lee et al. 2019 | retrospective | South-Korea | 1 | 911 |
| 245 | Lindner et al. 2022 | retrospective | Germany | 1 | 246 |
| 246 | Yunhe Liu et al. 2025 | retrospective | China | 1 | 1352 |
| 247 | Luo et al. 2018 | retrospective | China | 1 | 803 |
| 248 | Pennestri et al. 2024 | retrospective | Italy | 1 | 203 |
| 249 | Pardo et al. 2021 | retrospective | Spain | 1 | 161 |
| 250 | Rodolico et al. 2007 | retrospective | Italy | 1 | 214 |
| 251 | Song et al. 2009 | retrospective | South-Korea | 1 | 106 |
| 252 | Su et al. 2024 | retrospective | China | 1 | 256 |
| 253 | Sun et al. 2022 | retrospective | China | 1 | 375 |
| 254 | Tacchi et al. 2025 | retrospective | Italy | 2 | 145 |
| 255 | Tallini et al. 2015 | retrospective | Italy | 6 | 298 |
| 256 | Tian et al. 2024 | retrospective | China | 1 | 493 |
| 257 | Zhou et al. 2025 | retrospective | China | 1 | 214 |
| 258 | Xiaojun Zhang et al. 2021 | retrospective | China | 1 | 345 |
| 259 | Yin Zhu Zhao et al. 2022 | retrospective | China | 1 | 330 |
| 260 | Yoo et al. 2009 | retrospective | South-Korea | 1 | 165 |
| 261 | Zahan et al. 2016 | retrospective | Romania | 1 | 237 |
| 262 | Xi Zhang et al. 2018 | retrospective | China | 1 | 171 |
| 263 | Ge et al. 2024 | prospective | China | 1 | 73 |
| 264 | Rosario et al. 2021 | prospective | Brazil | 1 | 5 |
| 265 | Nagaoka et al. 2021 | prospective | Japan | 2 | 571 |
| 266 | Kwon et al. 2017 | prospective | South-Korea | 1 | 192 |
| 267 | Shindo et al. 2014 | prospective (retrospective) | Japan | 1 | 36 |
| 268 | Ghirri et al. 2023 | retrospective | Italy | 1 | 5 |
| 269 | Ito et al. 2014 | prospective | Japan | 1 | 1235 |
| 270 | Ito et al. 2016 | prospective | Japan | 1 | 1549 |
| 271 | Lee et al. 2022 | prospective | South-Korea | 3 | 706 |
| 272 | Wen Liu et al. 2022 | prospective | China | 1 | 115 |
| 273 | Liu et al. 2017 | retrospective | China | 1 | 712 |
| 274 | Biase et al. 2015 | retrospective | Italy | 1 | 266 |

**Supplementary Table 3** | Main predictors based on outcome

| **Predictor** | **Outcome** | **No. of studies** | **Total patients** |
| --- | --- | --- | --- |
| **Sex (male)** | central lymph node metastasis | 104 | 112814 |
|  | lateral lymph node metastasis | 44 | 85808 |
|  | undetermined lymph node metastasis | 49 | 34322 |
|  | extrathyroidal extension | 15 | 7657 |
| **Age (<45 y.o.)** | central lymph node metastasis | 57 | 35367 |
|  | lateral lymph node metastasis | 17 | 15937 |
|  | undetermined lymph node metastasis | 21 | 13619 |
|  | extrathyroidal extension | 9 | 5299 |
| **Age (<55 y.o.)** | central lymph node metastasis | 18 | 56953 |
|  | lateral lymph node metastasis | 14 | 63302 |
|  | undetermined lymph node metastasis | 12 | 7649 |
|  | extrathyroidal extension | 4 | 1863 |
| **Tumor size (>5 mm)** | central lymph node metastasis | 60 | 78479 |
|  | lateral lymph node metastasis | 22 | 42883 |
|  | undetermined lymph node metastasis | 36 | 20531 |
|  | extrathyroidal extension | 14 | 5046 |
| **Tumor size (>6 mm)** | lymph node metastasis | 5 | 2449 |
| **Tumor size**  **(>6,5 mm)** | central lymph node metastasis | 3 | 5036 |
| **Tumor size (>7 mm)** | central lymph node metastasis | 6 | 3458 |
|  | lateral lymph node metastasis | 6 | 20510 |
| **Multifocality** | central lymph node metastasis | 90 | 104548 |
|  | lateral lymph node metastasis | 32 | 61918 |
|  | undetermined lymph node metastasis | 45 | 24023 |
|  | extrathyroidal extension | 13 | 4334 |
| **Bilaterality** | central lymph node metastasis | 51 | 43041 |
|  | lateral lymph node metastasis | 16 | 10036 |
|  | undetermined lymph node metastasis | 19 | 6739 |
|  | extrathyroidal extension | 13 | 4334 |
| **Hashimoto’s thyroiditis** | central lymph node metastasis | 65 | 65550 |
|  | lateral lymph node metastasis | 29 | 31439 |
|  | undetermined lymph node metastasis | 29 | 20145 |
|  | extrathyroidal extension | 5 | 3452 |
| **BRAFV600E mutation** | central lymph node metastasis | 33 | 9326 |
|  | lateral lymph node metastasis | 19 | 8854 |
|  | undetermined lymph node metastasis | 22 | 6803 |
|  | extrathyroidal extension | 15 | 3961 |
| **Obesity (obese)** | central lymph node metastasis | 3 | 927 |

b. Supplementary results – figures and plots

**Obesity**

The relationship between obesity and risk of CLNM was the following: The OR was 0.85 (CI: 0.32-2.24; I^2^ = 23%, CI: 0-92).

**Microcalcification**

The relationship between microcalcification and risk of LNM was the following: The OR in case of CLNM was 1.97 (CI: 1.61–2.41; I^2^ = 79%, CI: 70–86), at LLNM it was 2.24 (CI: 0.74–6.80; I^2^ = 78%, CI: 48–91) and finally at udLNM the OR was 2.54 (CI: 0.23–28.05; I^2^ = 98%, CI: 96–99).

**Capsule invasion**

The relationship between capsule invasion and risk of LNM was the following: The OR in case of CLNM was 2.17 (CI: 1.70–2.77; I^2^ = 80%, CI: 72–85), at LLNM it was 2.96 (CI: 1.35–6.50; I^2^ = 83%, CI: 71–90) and finally at udLNM the OR was 1.63 (CI: 1.10–2.44; I^2^ = 82%, CI: 74–88).

**Goiter**

The relationship between goiter and risk of LNM was the following: The OR in case of CLNM was 0.90 (CI: 0.76–1.05; I^2^ = 44%, CI: 0–72), at LLNM it was 1.07 (CI: 0.63–1.82; I^2^ = 37%, CI: 0–76) and finally at udLNM the OR was 0.80 (CI: 0.04–15.73; I^2^ = 95%, CI: 90–97).

**Graves’s disease**

The relationship between Graves’ disease and the risk of udLNM was as follows: OR 4.17 (CI: 0.00–8244.85; I^2^ = 80%, CI: 37–94).

**Anti-Tg**

The relationship between anti-Tg and risk of LNM was the following: The OR in case of CLNM was 0.79 (CI: 0.57–1.09; I^2^ = 0%, CI: 0–90), at udLNM the OR was 0.96 (CI: 0.59–1.54; I^2^ = 10%, CI: 0–86).

**Increased TSH**

The relationship between increased TSH and risk of udLNM was the following: The OR was 1.03 (CI: 0.80–1.33; I^2^ = 0%, CI: 0–90).

**TERT mutation**

The relationship between TERT mutation and risk of udLNM was the following: The OR was 1.48 (CI: 0.71–3.06; I^2^ = 0%, CI: 0–90).

**Supplementary Figure 1 a-b** | Forest and funnel plots of male sex and its influence in the case of central lymph node metastasis (CLNM)

a.)


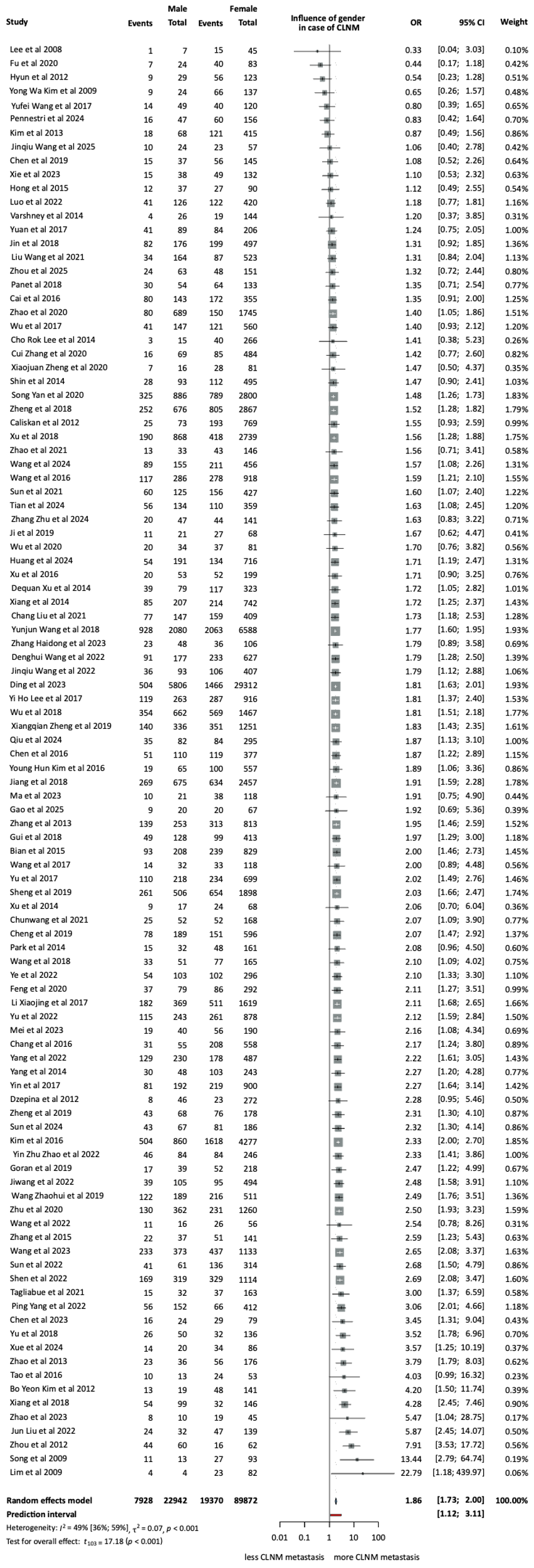


b.)


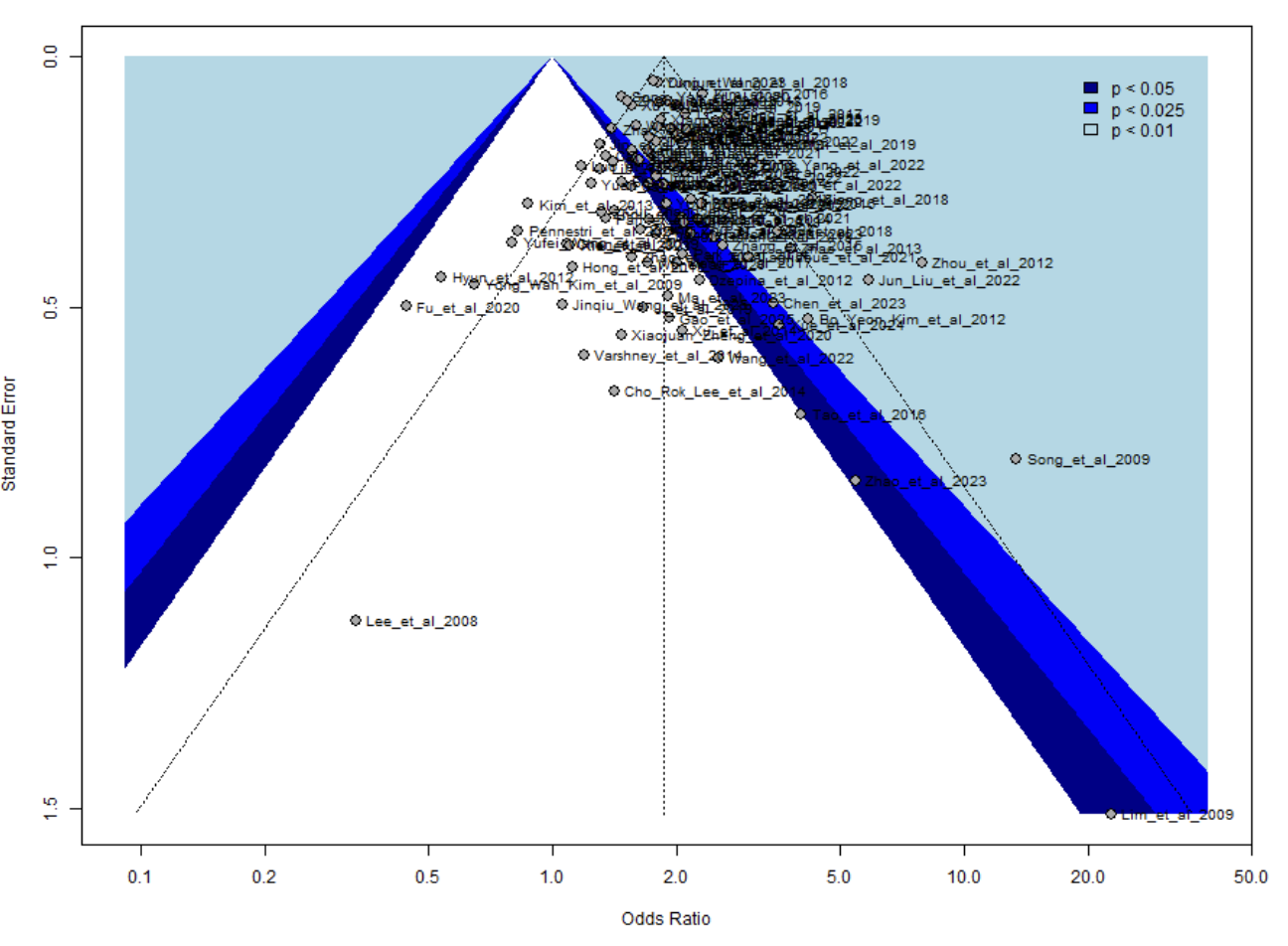


Egger’s test p= 0.3606

**Supplementary Figure 2 a-b** | Forest and funnel plots of age under 45 and its influence in the case of central lymph node metastasis (CLNM)

a.)


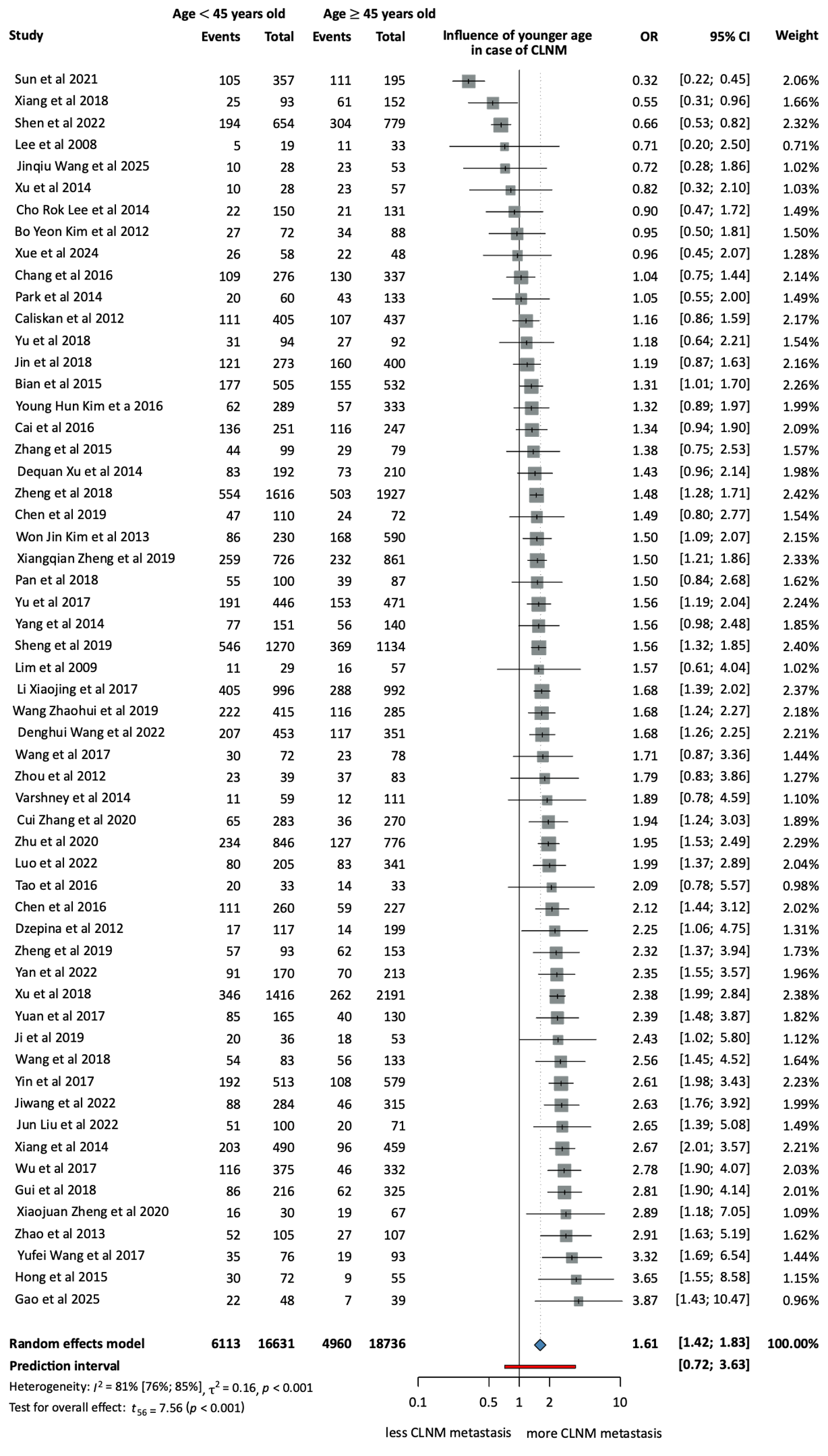


b.)


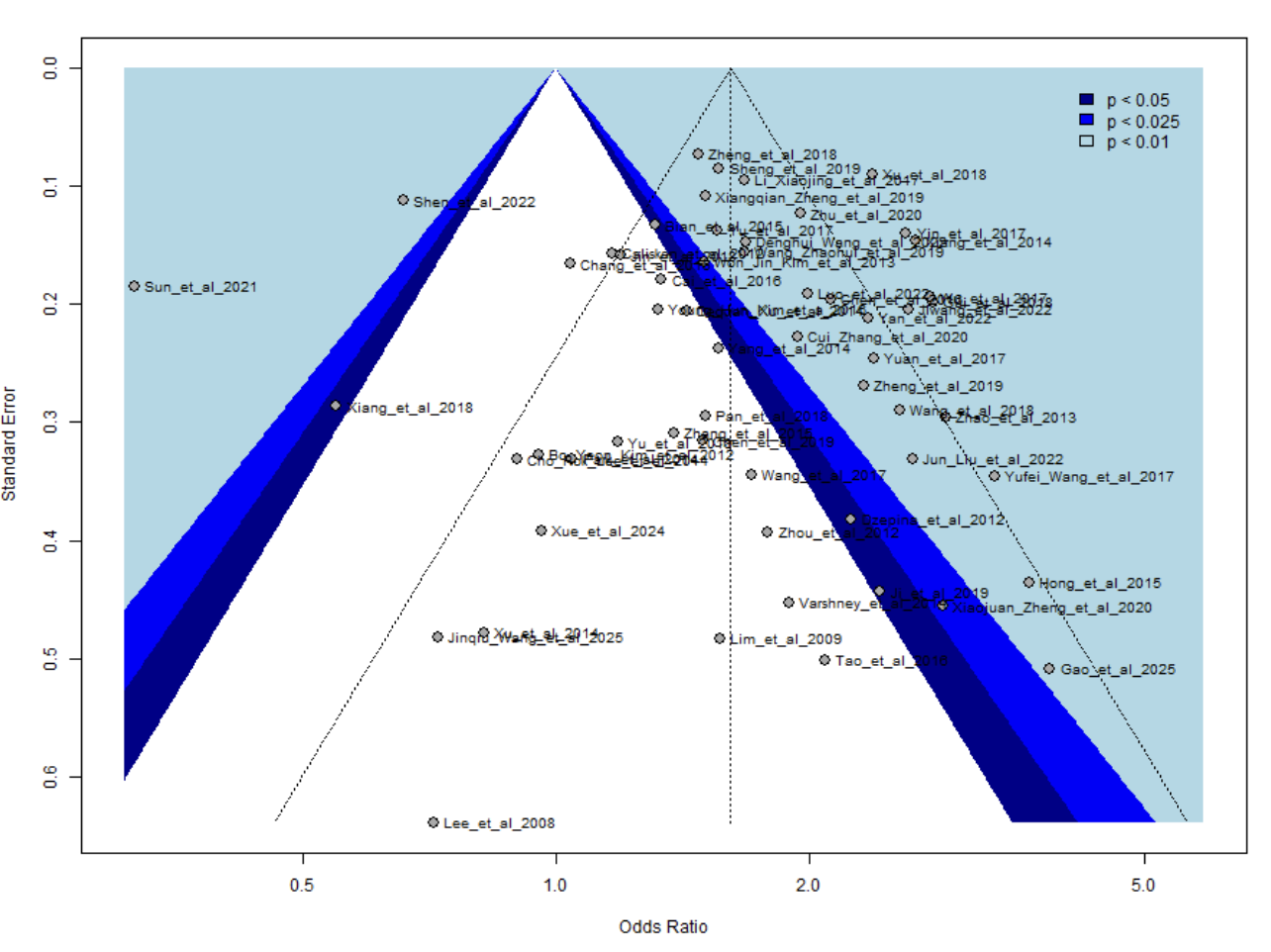


Egger’s test p= 0.9439

**Supplementary Figure 3 a-b** | Forest and funnel plots of age under 55 and its influence in the case of central lymph node metastasis (CLNM)

a.)


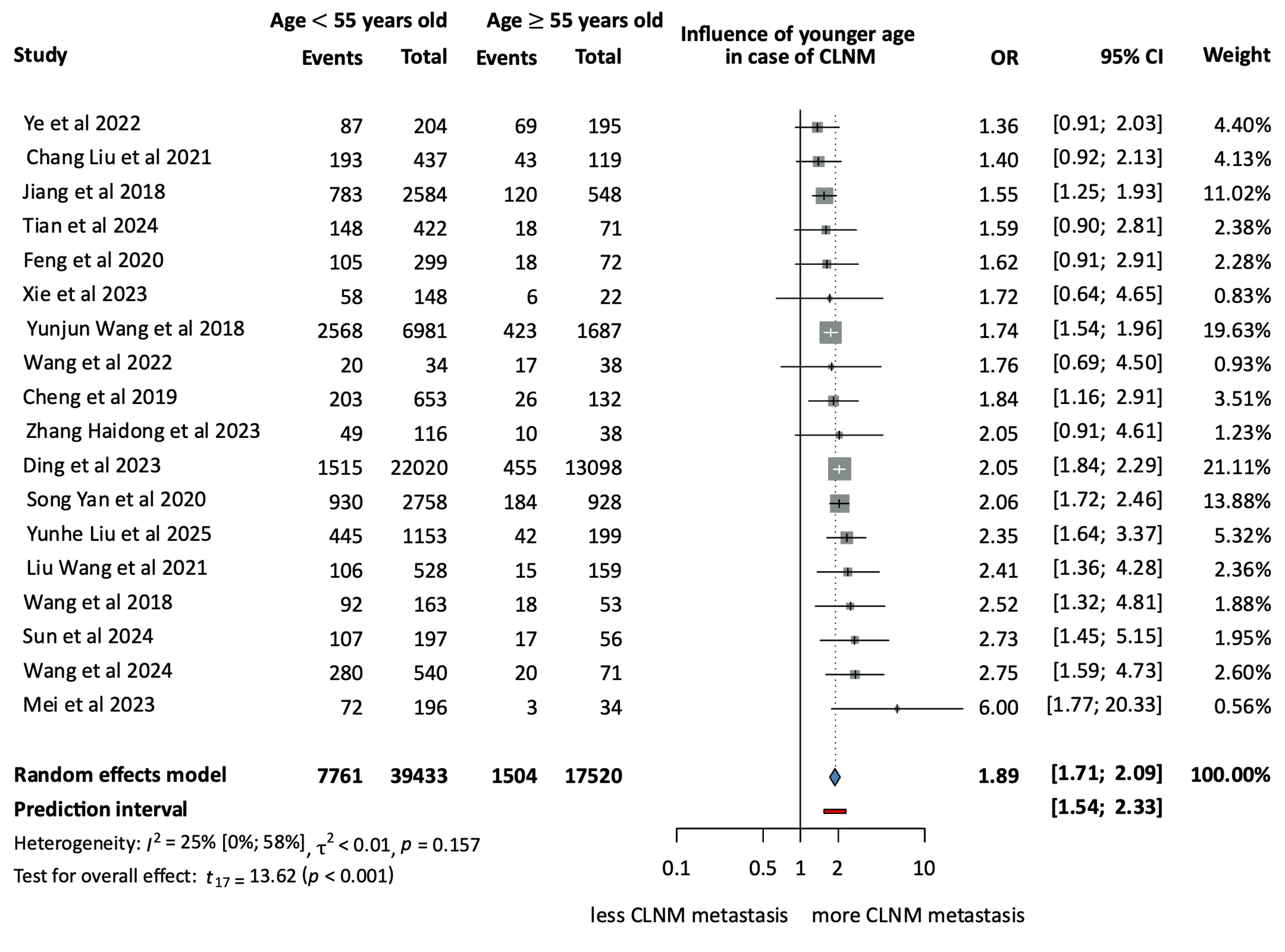


b.)


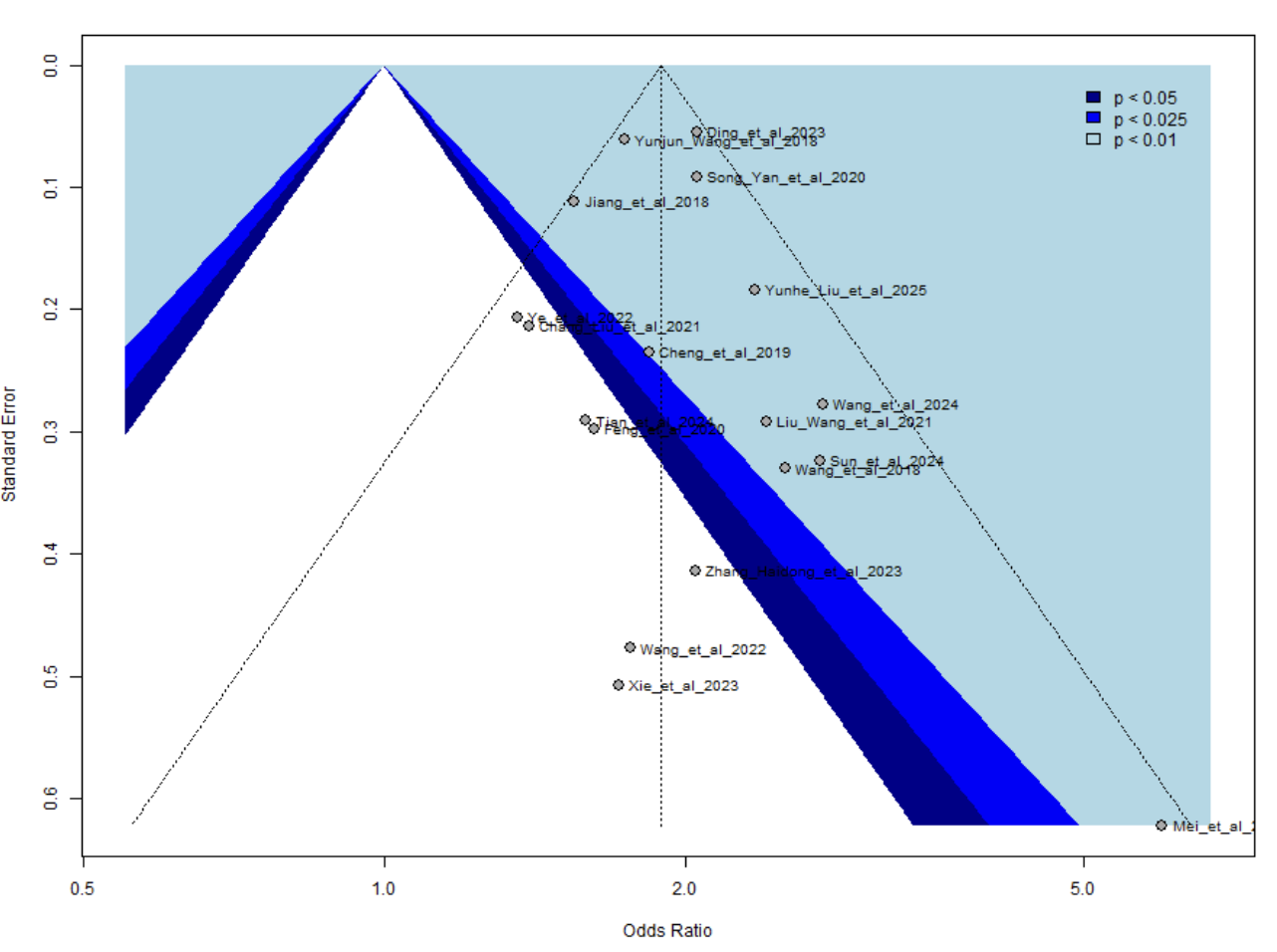


Egger’s test p= 0.4672

**Supplementary Figure 4 a-b** | Forest and funnel plots of tumor size above 5 mm and its influence in the case of central lymph node metastasis (CLNM)

a.)


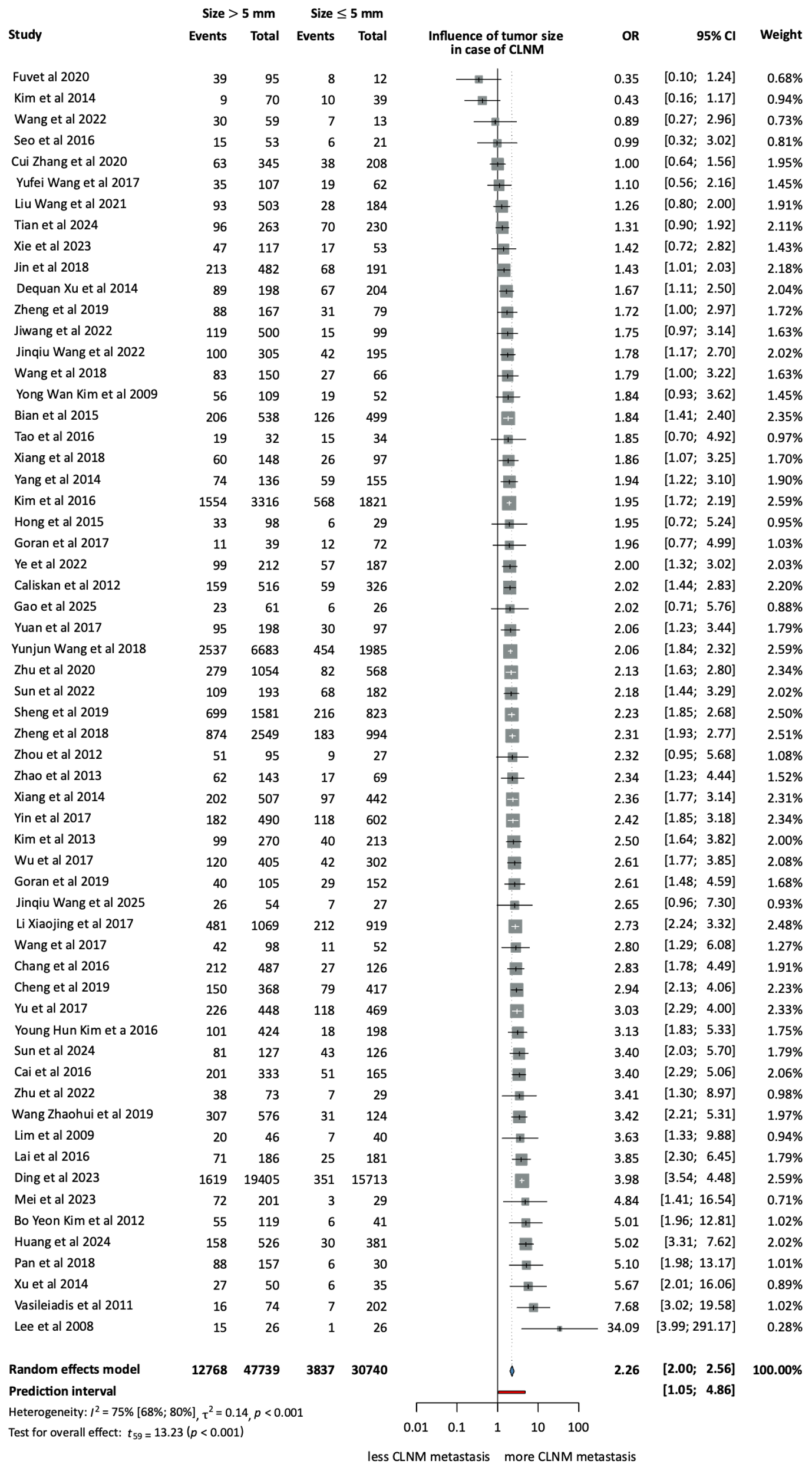


b.)


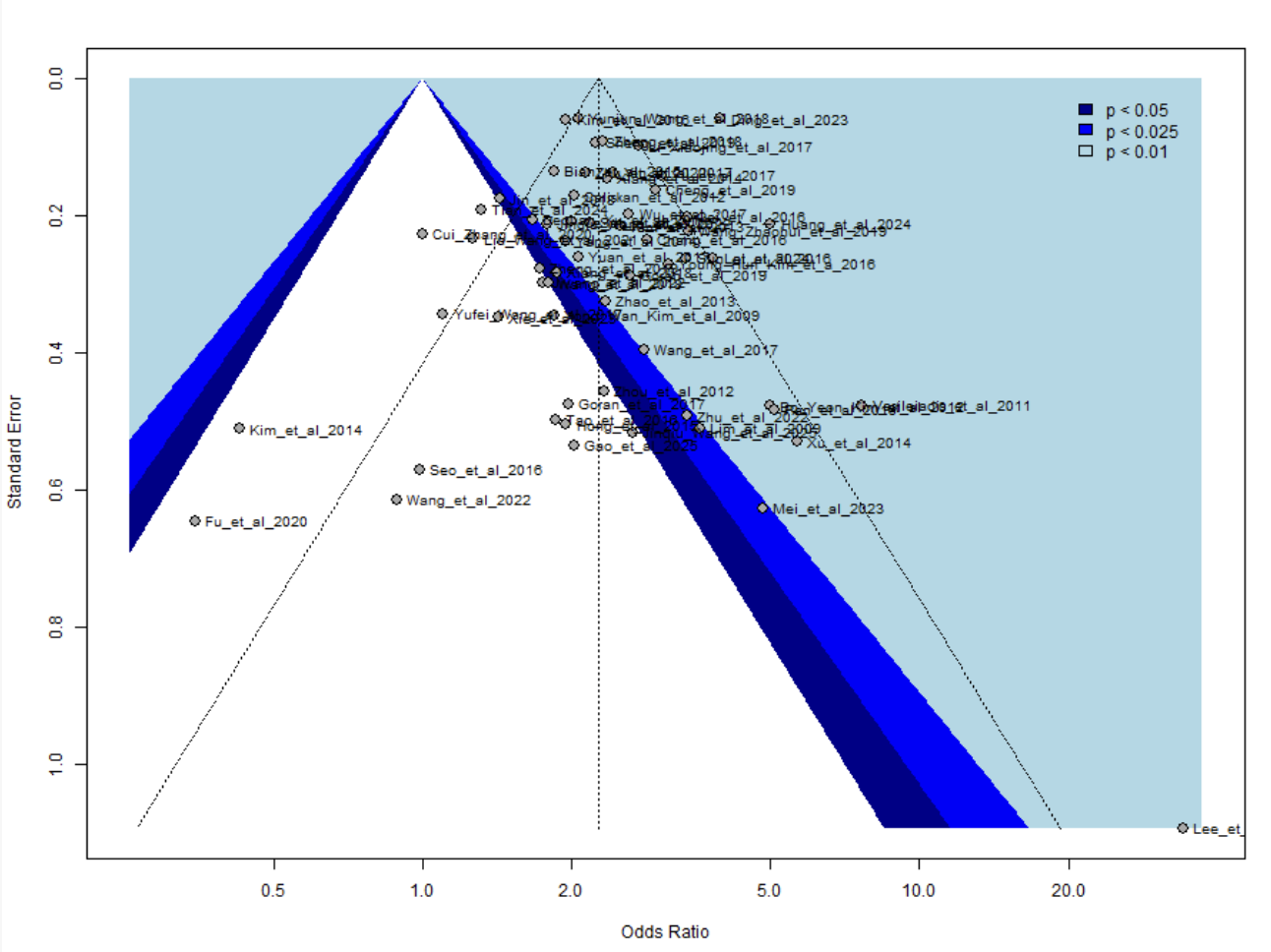


Egger’s test p= 0.4369

**Supplementary Figure 6** | Forest plot of tumor size above 6.5 mm and its influence in the case of central lymph node metastasis (CLNM)


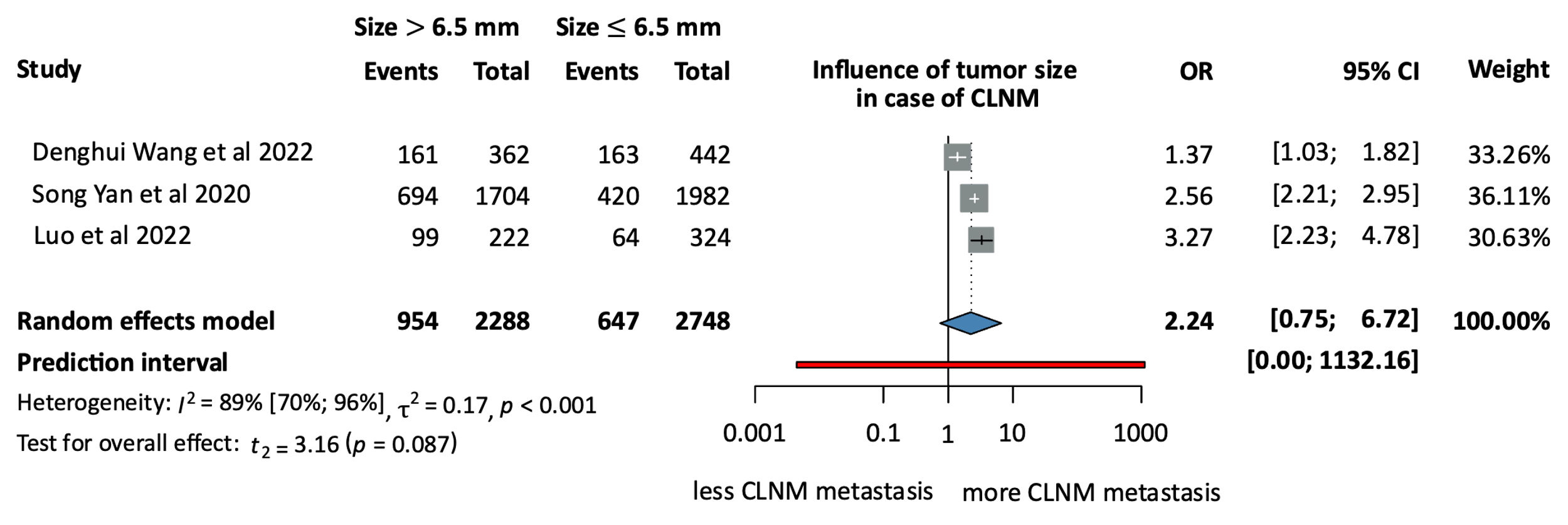


**Supplementary Figure 7** | Forest plot of tumor size above 7 mm and its influence in the case of central lymph node metastasis (CLNM)


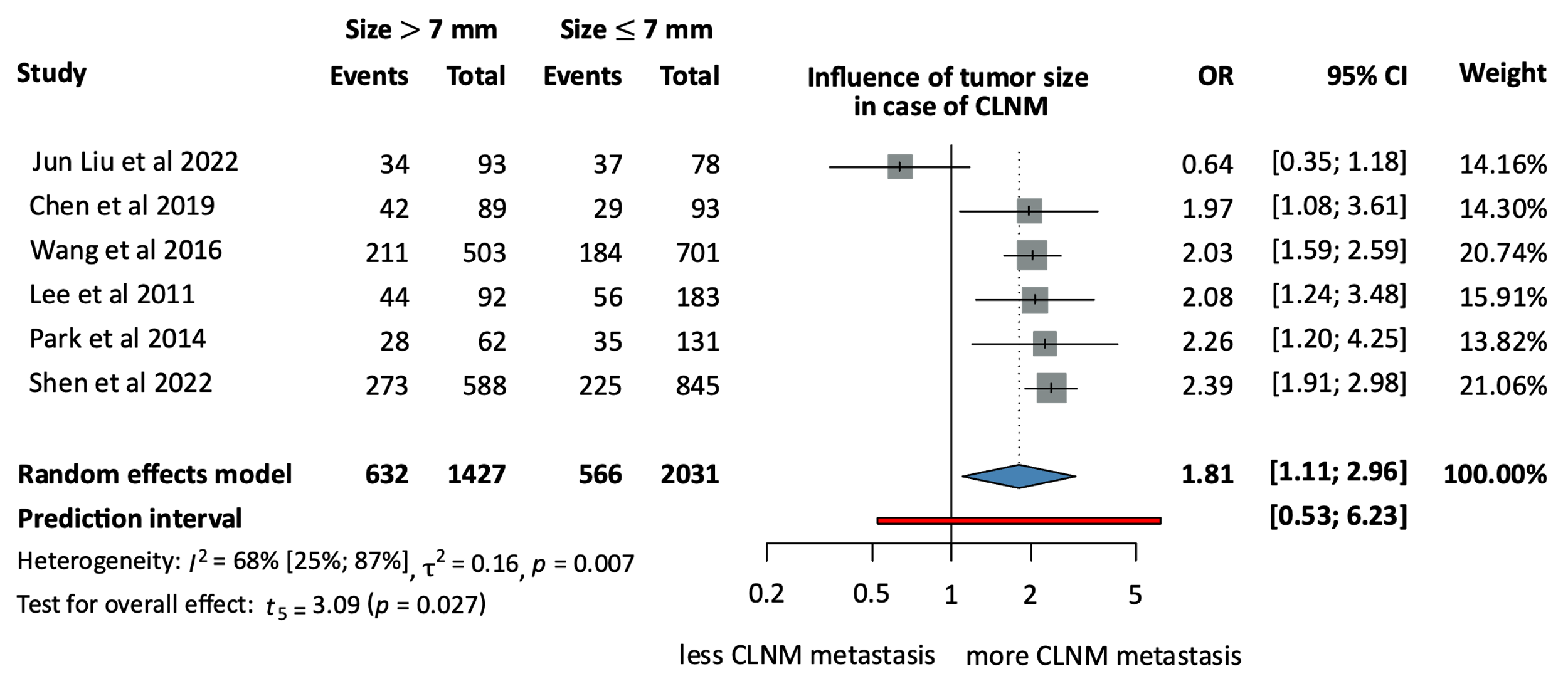


**Supplementary Figure 8 a-b** | Forest and funnel plot sof multifocality and its influence in the case of central lymph node metastasis (CLNM)

a.)


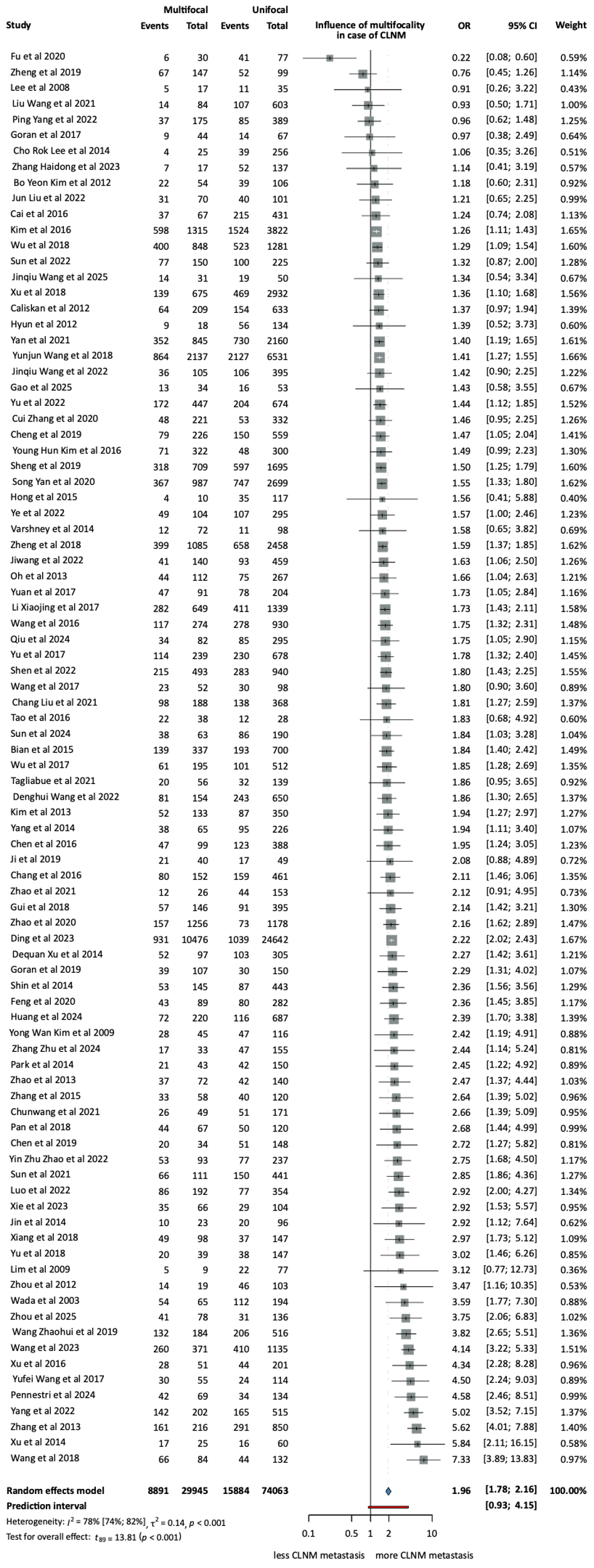


b.)


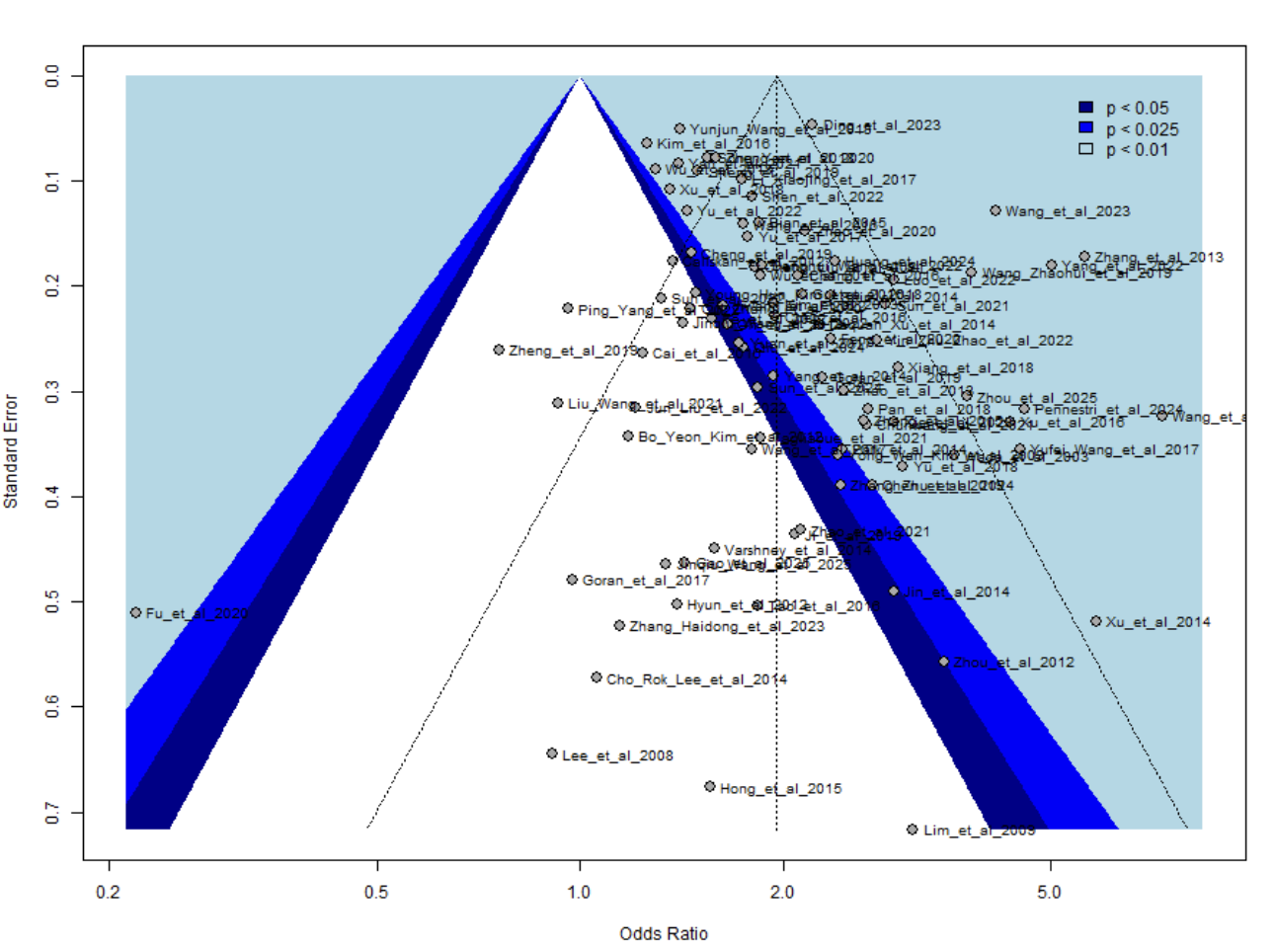


Egger’s test p= 0.0780

**Supplementary Figure 9 a-b** | Forest and funnel plots of bilaterality and its influence in the case of central lymph node metastasis (CLNM)

a.)


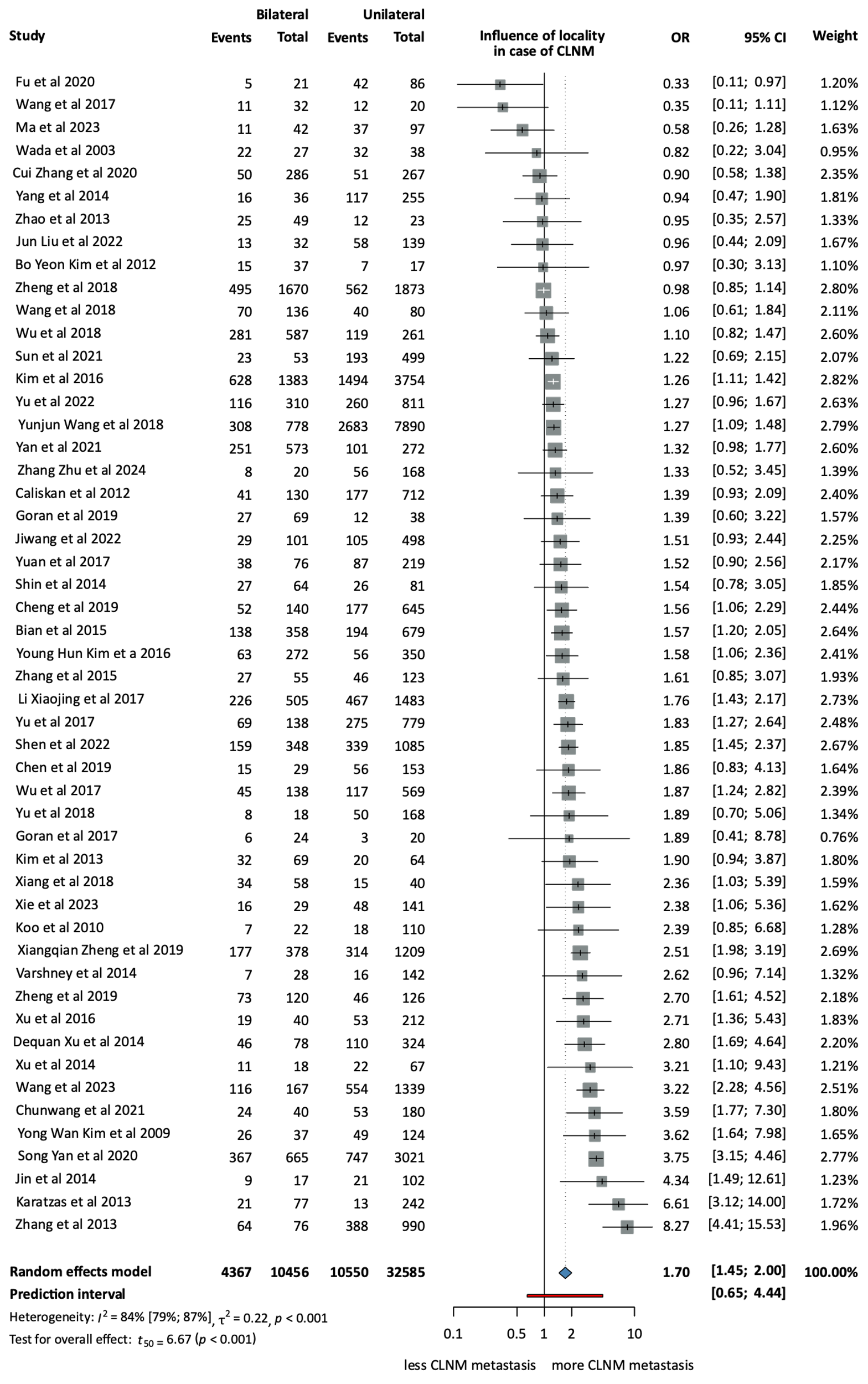


b.)


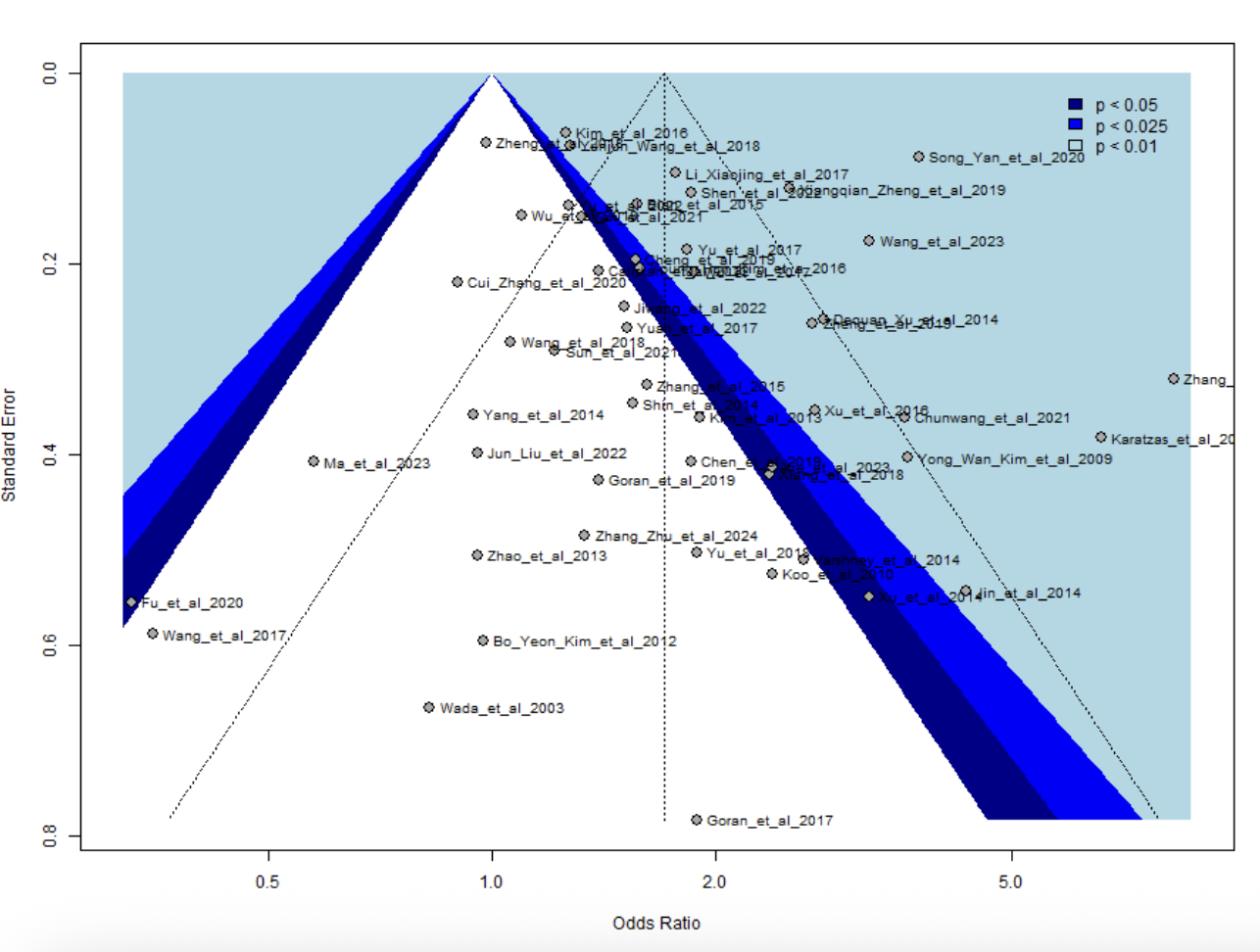


Egger’s test p= 0.9250

**Supplementary Figure 10 a-b** | Forest and funnel plots of BRAF^V600E^ mutation and its influence in the case of central lymph node metastasis (CLNM)

a.)


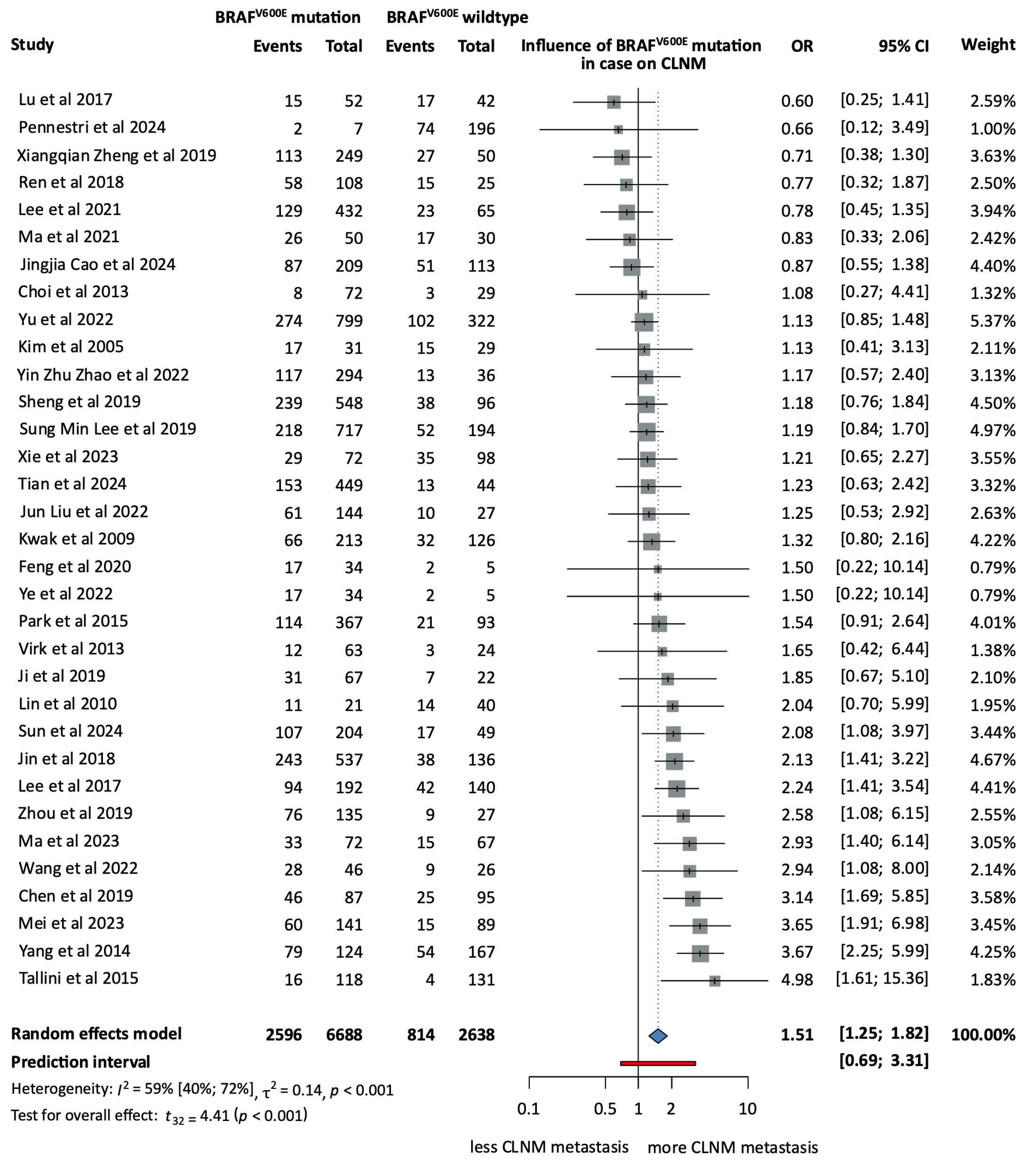


b.)


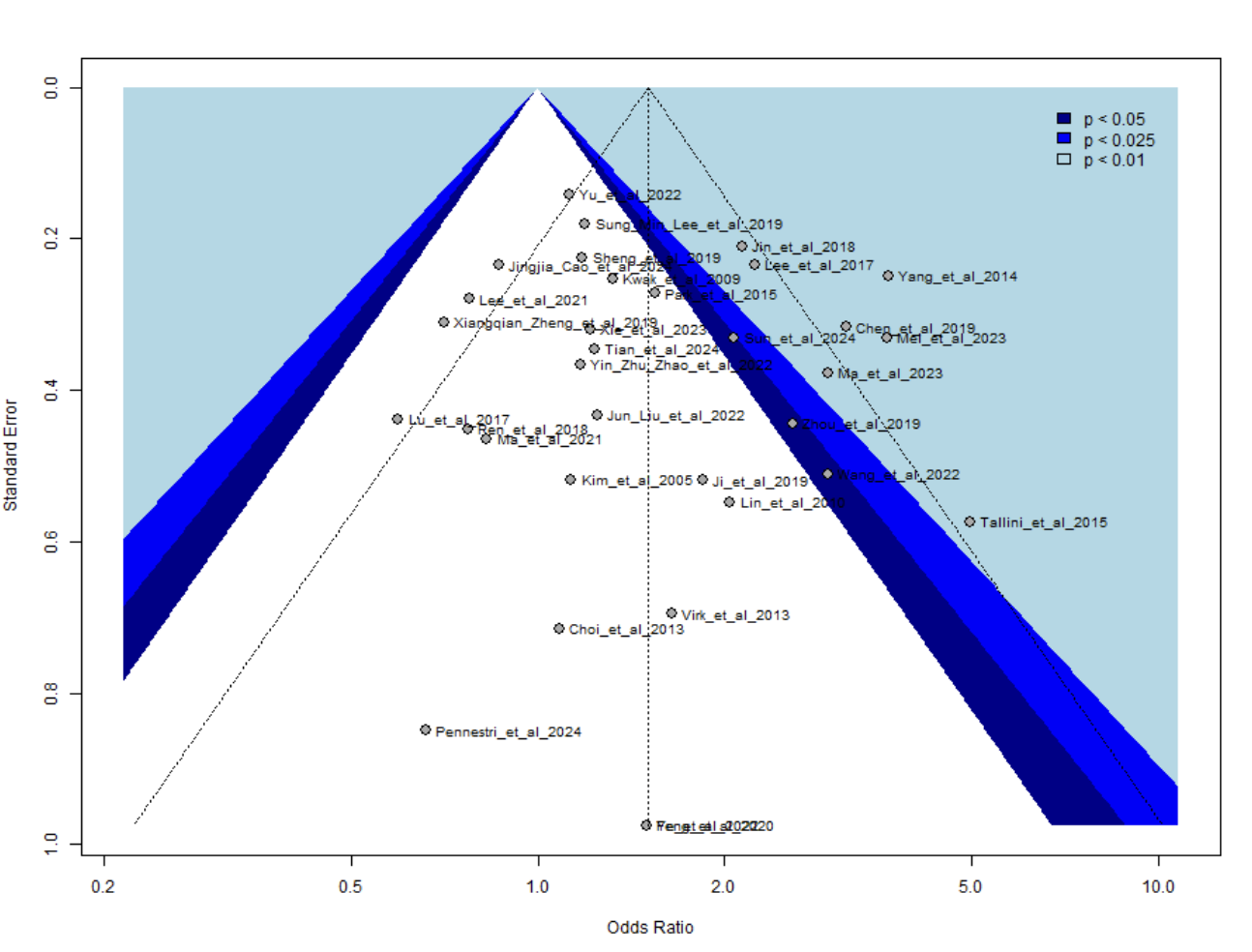


Egger’s test p= 0.5446

**Supplementary Figure 11 a-b** | Forest and funnel plots of Hashimoto’s thyroiditis and its influence in the case of central lymph node metastasis (CLNM)

a.)


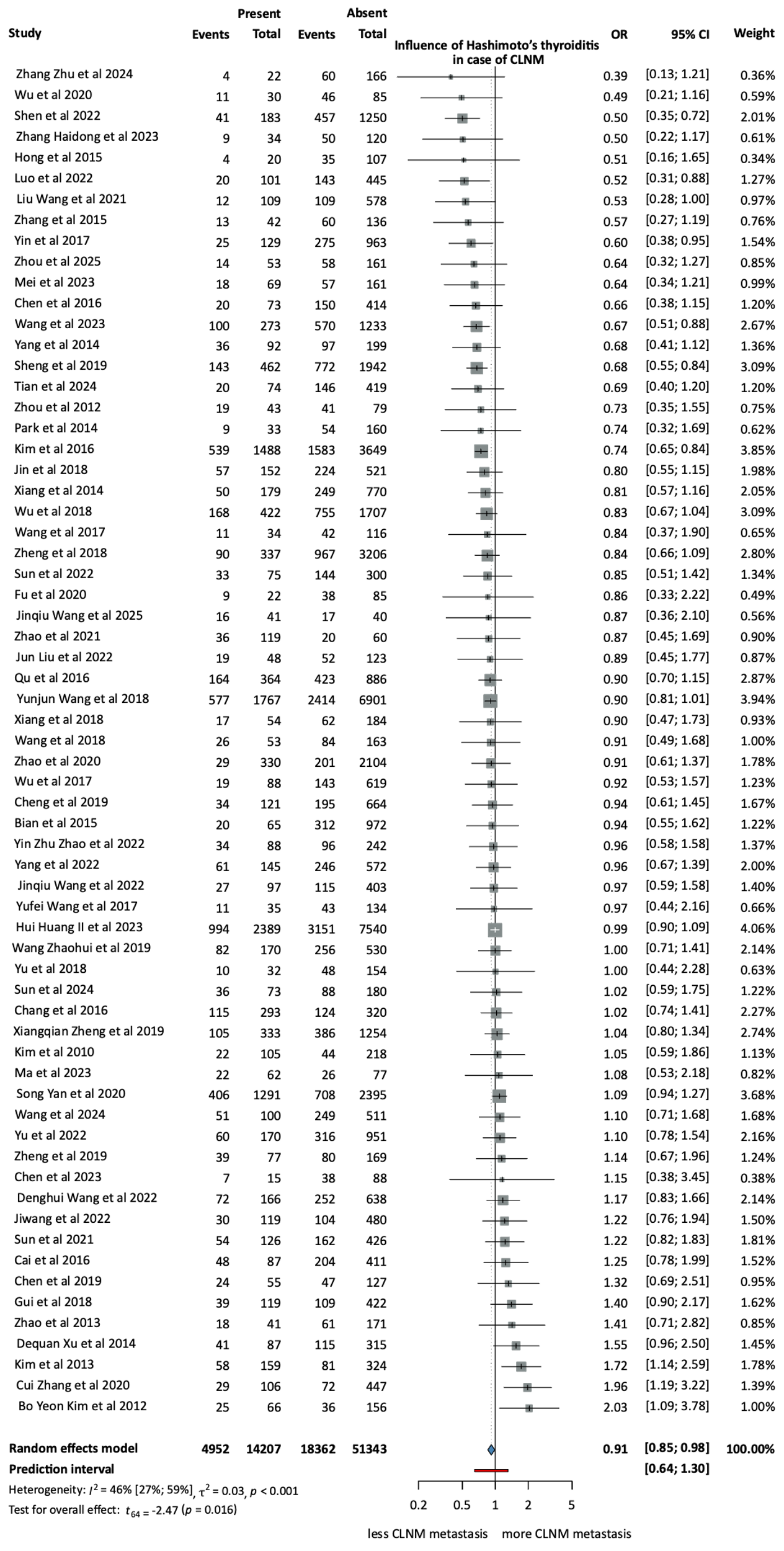


b.)


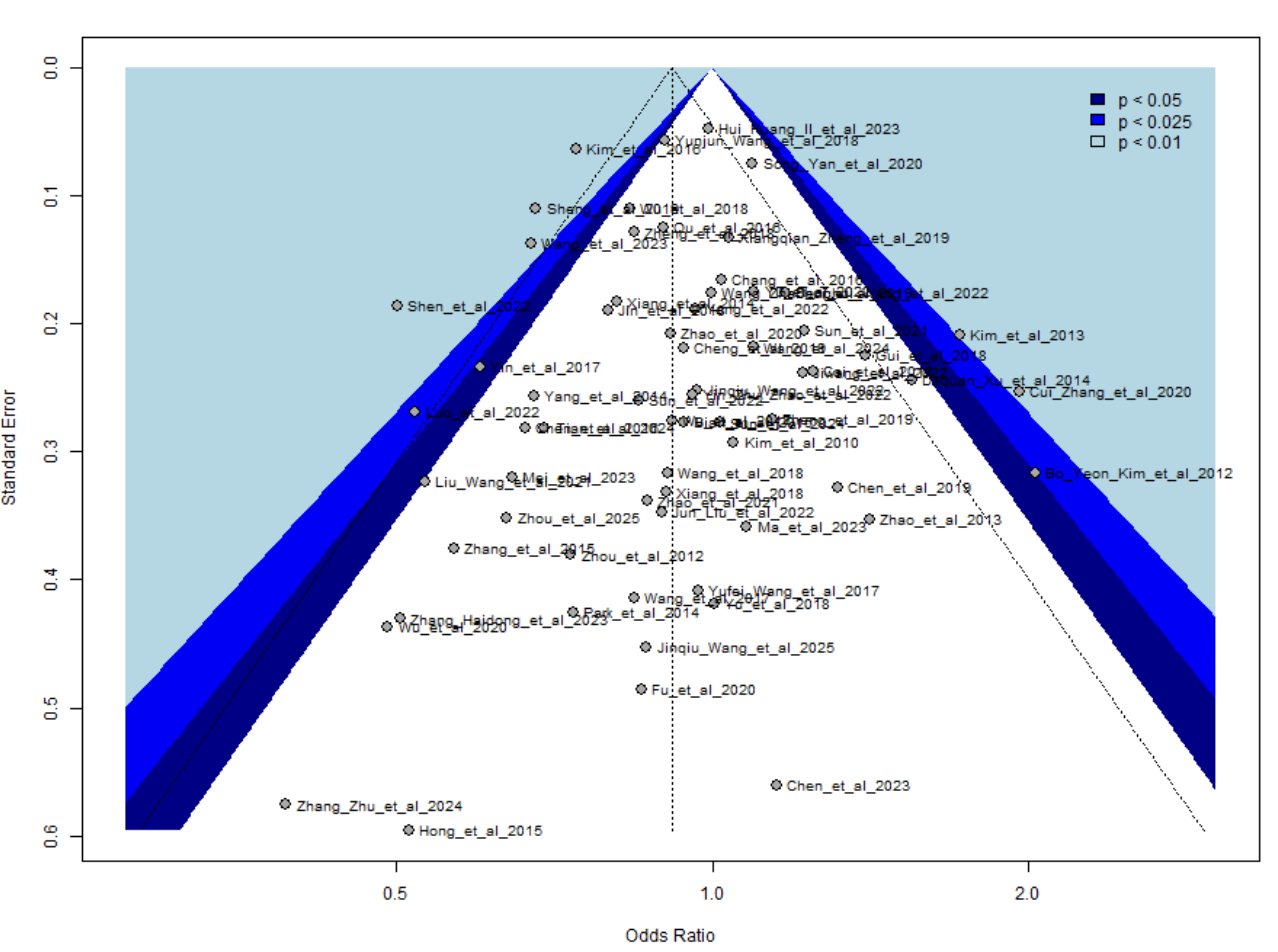


Egger’s test p= 0.9381

**Supplementary Figure 12** | Forest plot of obesity and its influence in the case of central lymph node metastasis (CLNM)


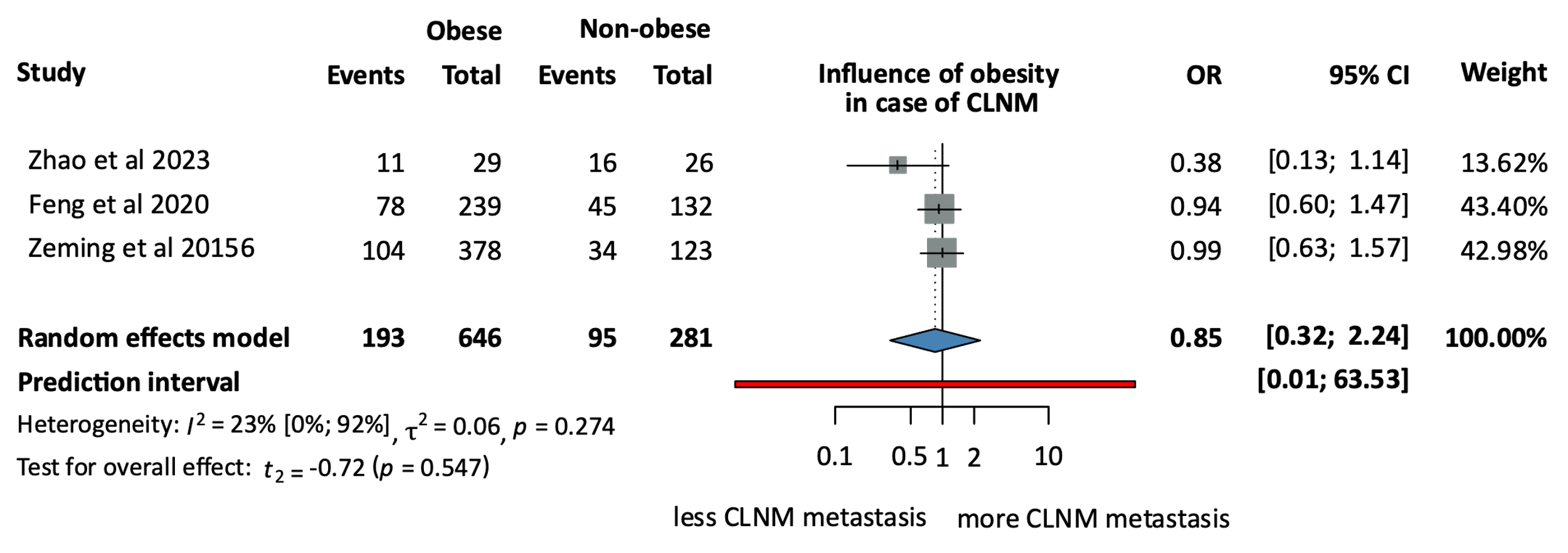


**Supplementary Figure 13** | Forest plot of thyroglobulin antibody (anti-Tg) and its influence in the case of central lymph node metastasis (CLNM)


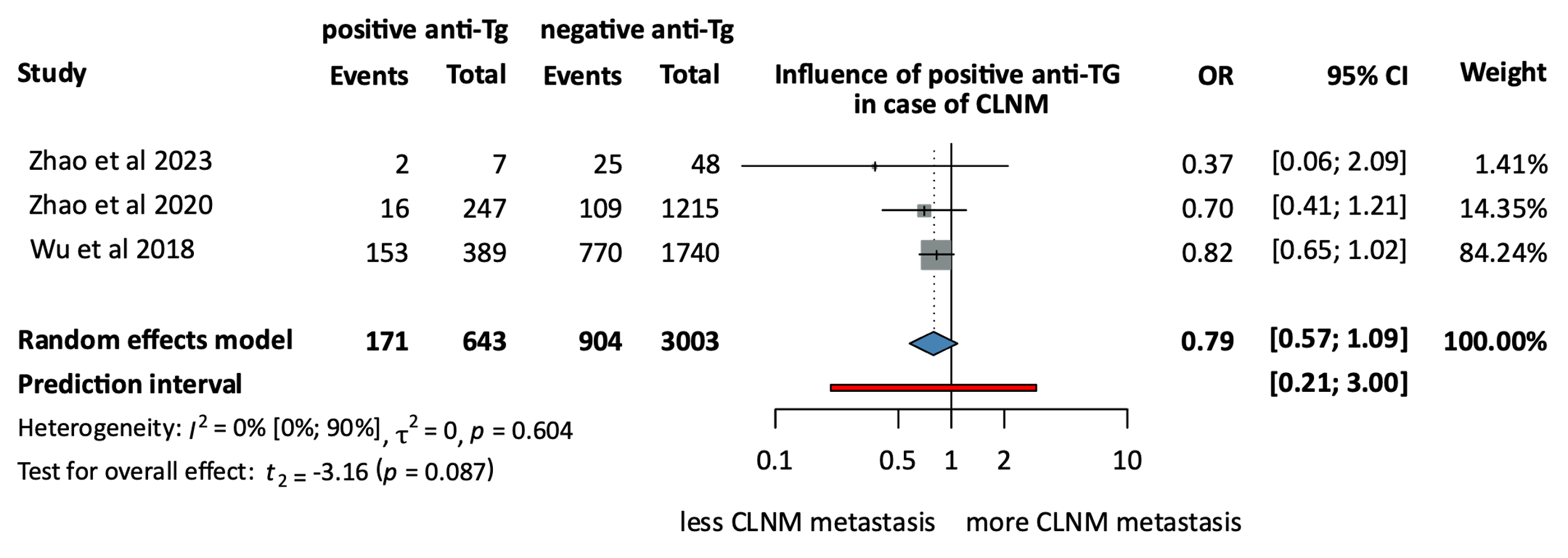


**Supplementary Figure 14** **a-b**| Forest and funnel plots of microcalcification and its influence in the case of central lymph node metastasis (CLNM)

a.)


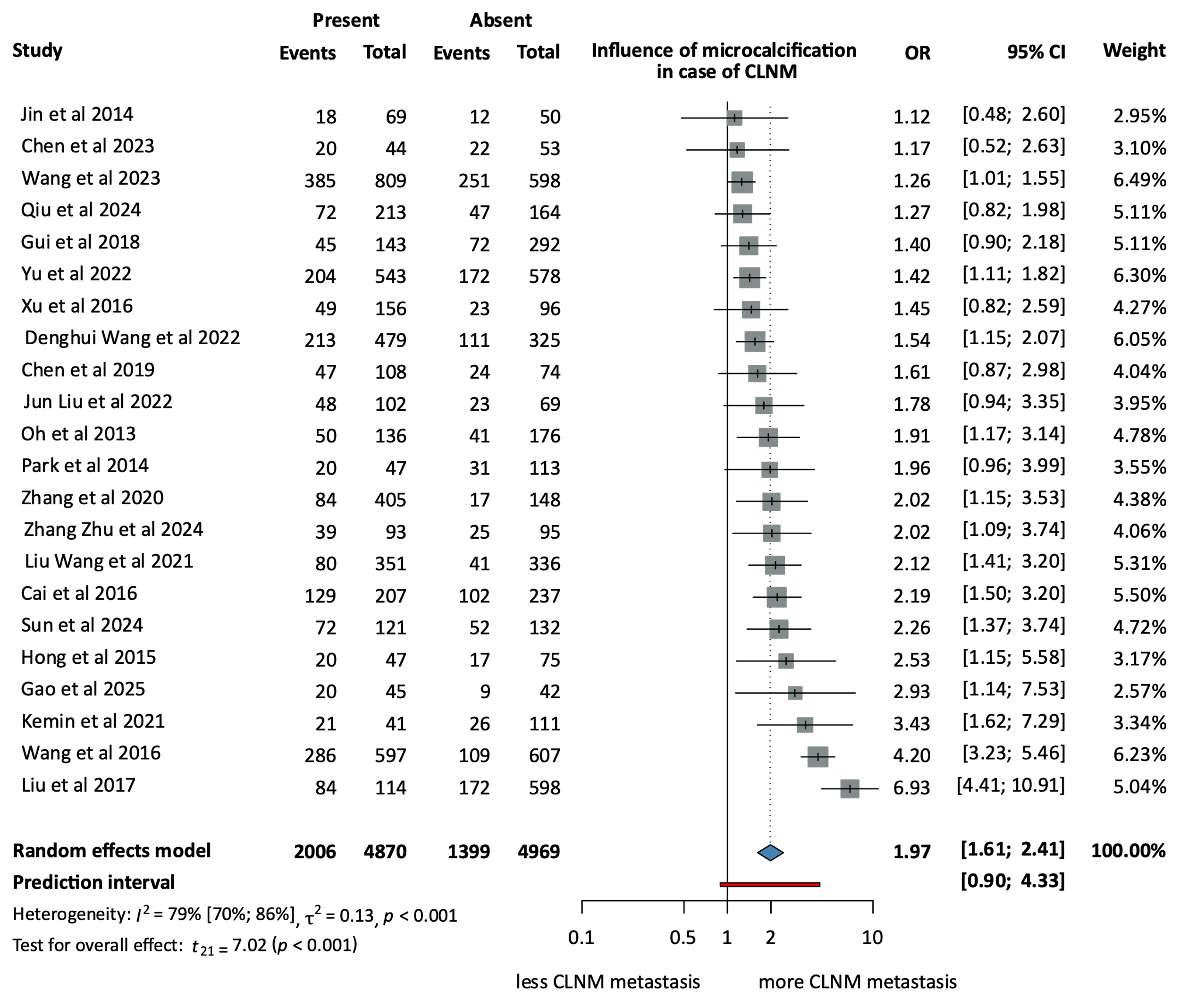


b.)


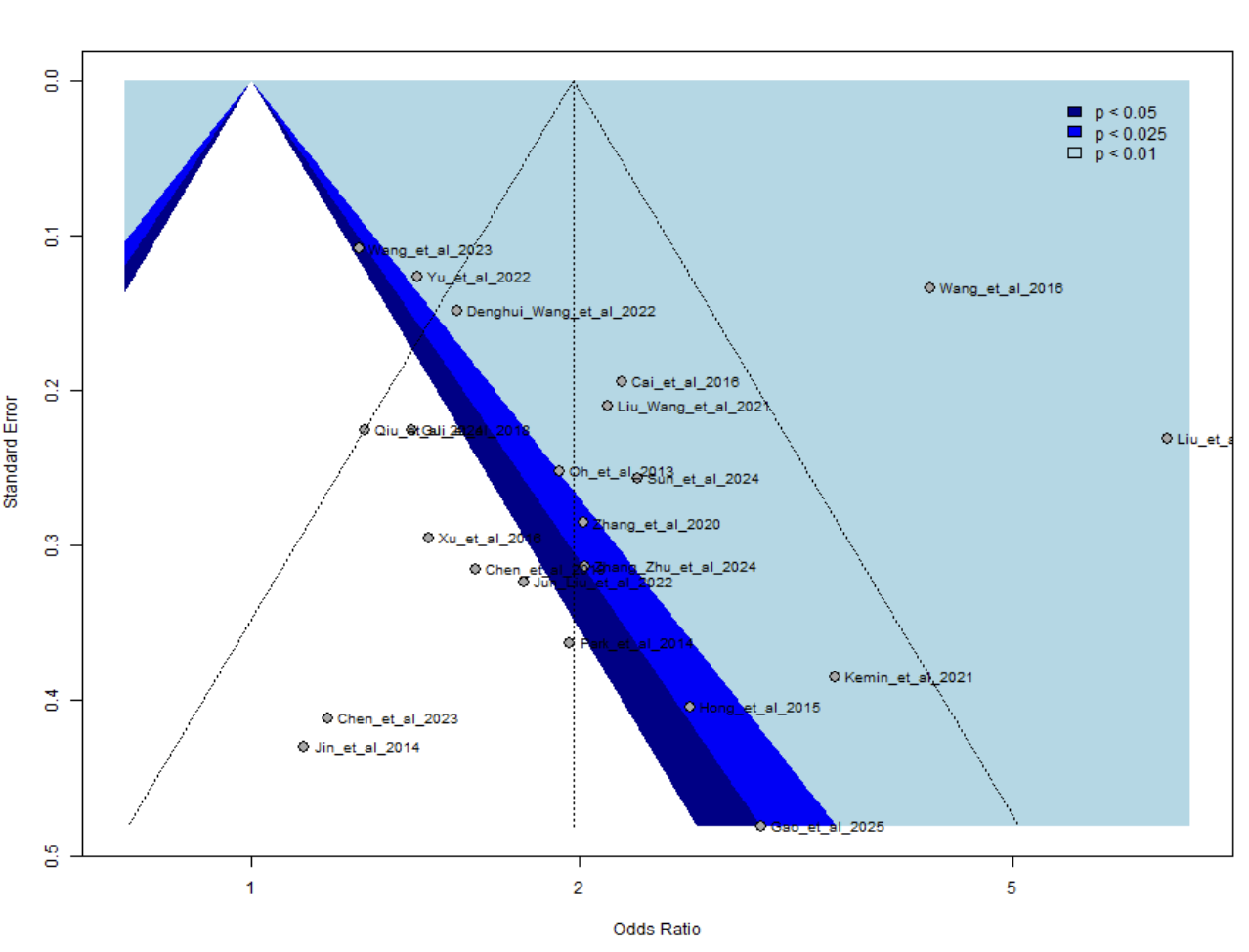


Egger’s test p= 0.8184

**Supplementary Figure 15** **a-b**| Forest and funnel plots of goiter and its influence in the case of central lymph node metastasis (CLNM)

a.)


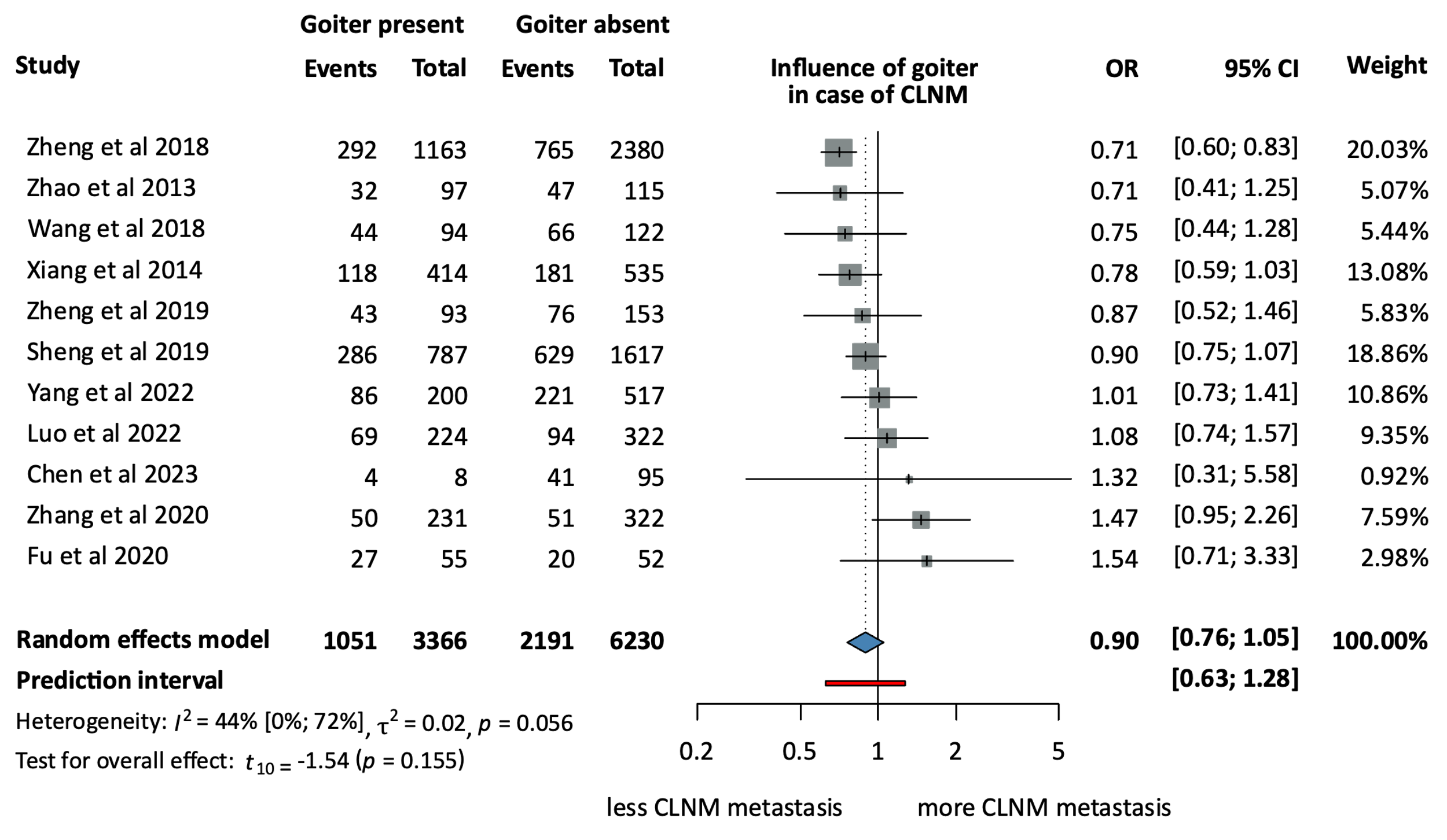


b.)


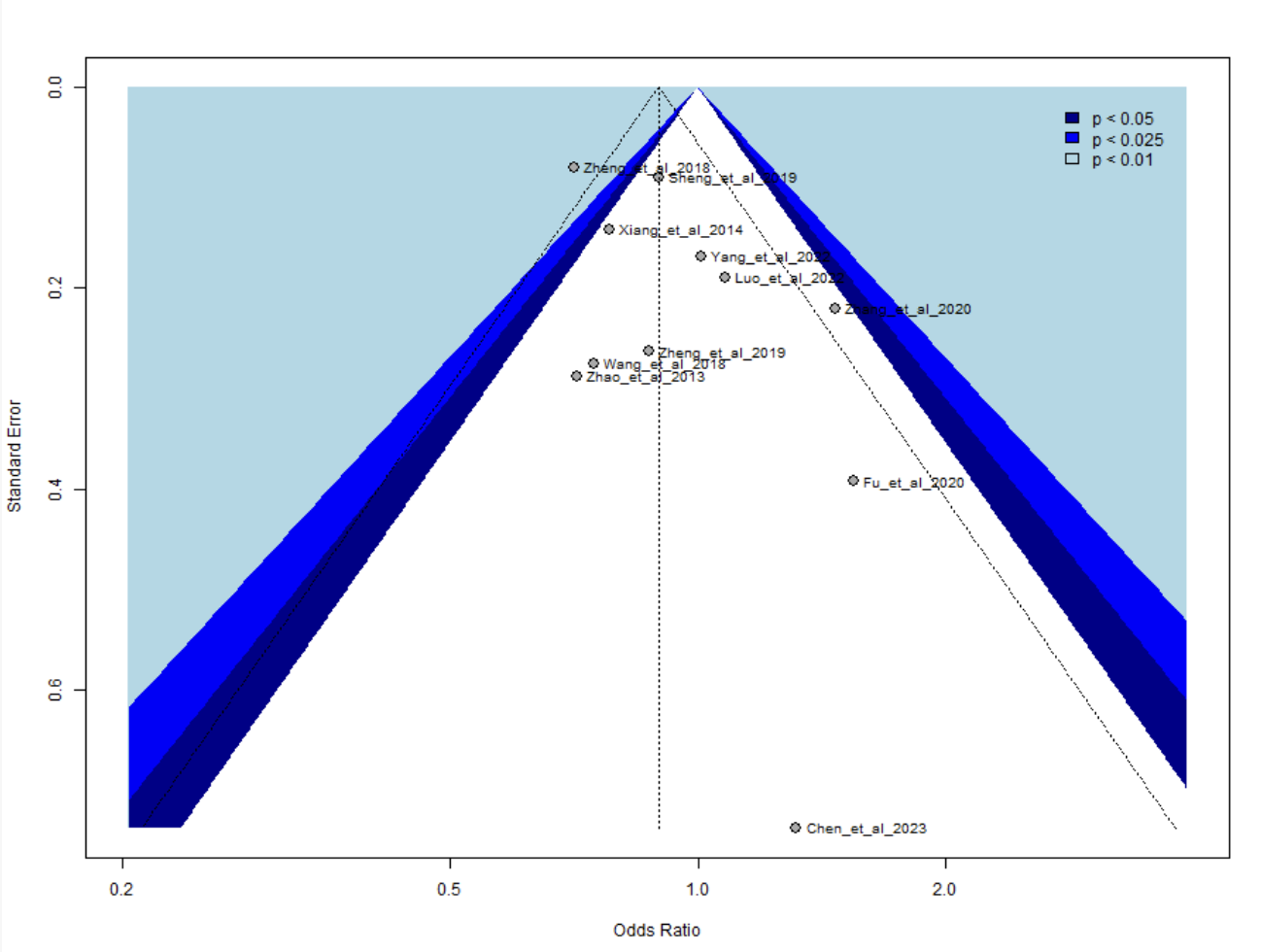


Egger’s test p= 0.1623

**Supplementary Figure 16** **a-b**| Forest and funnel plots of capsule invasion and its influence in the case of central lymph node metastasis (CLNM)

a.)


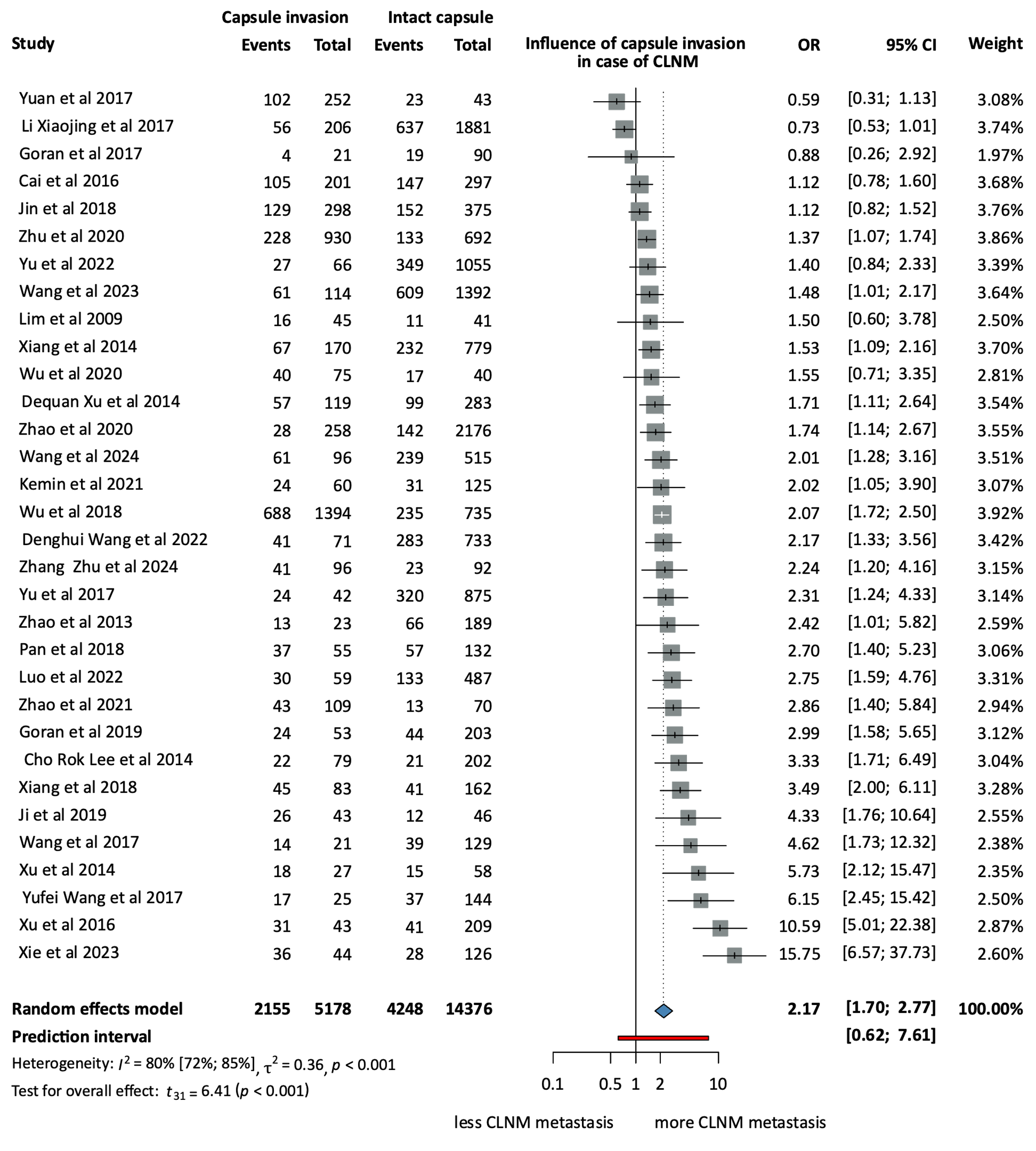


b.)


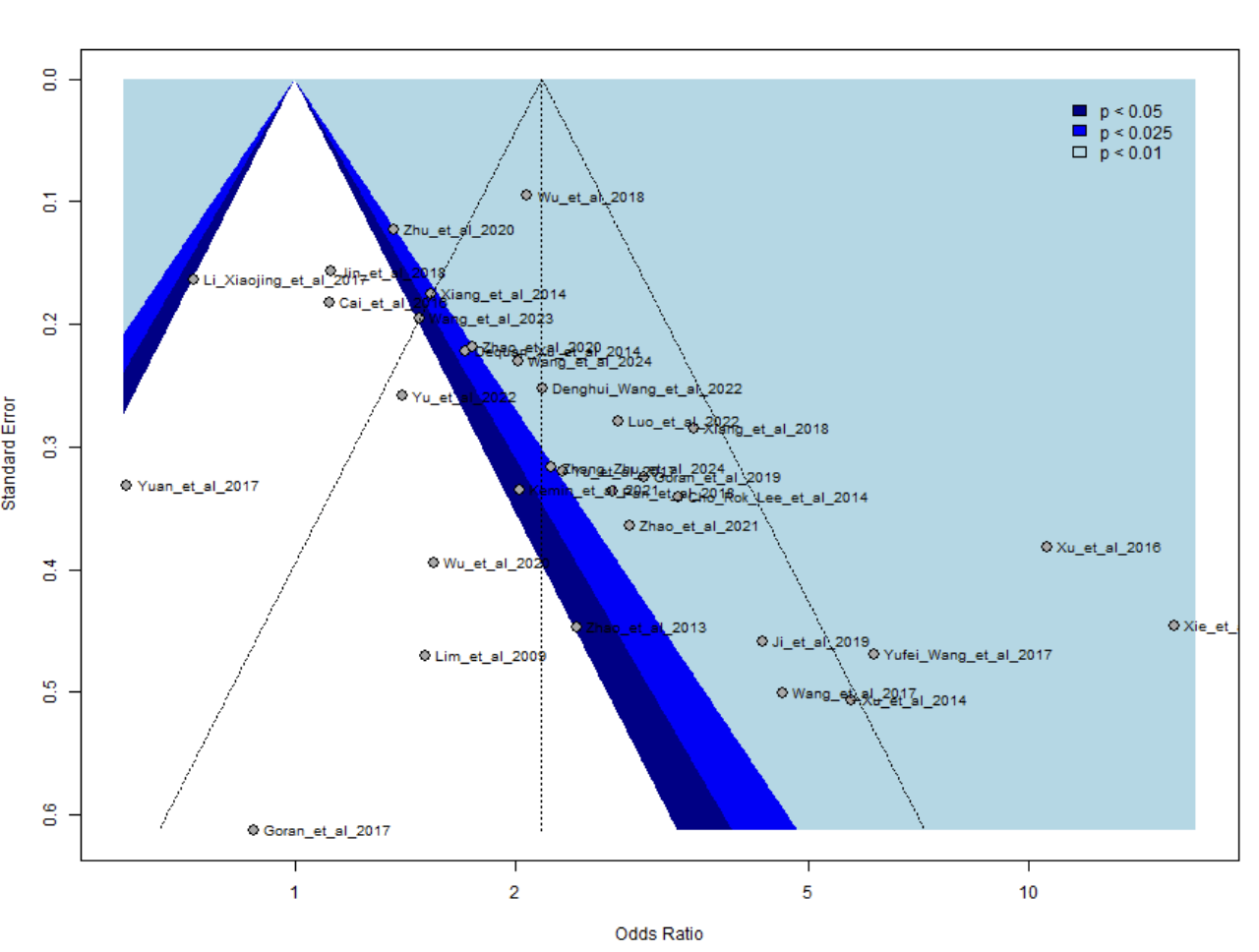


Egger’s test p= 0.0083

**Supplementary Figure 17 a-b** | Forest and funnel plots of male sex and its influence in the case of lateral lymph node metastasis (LLNM)

a.)


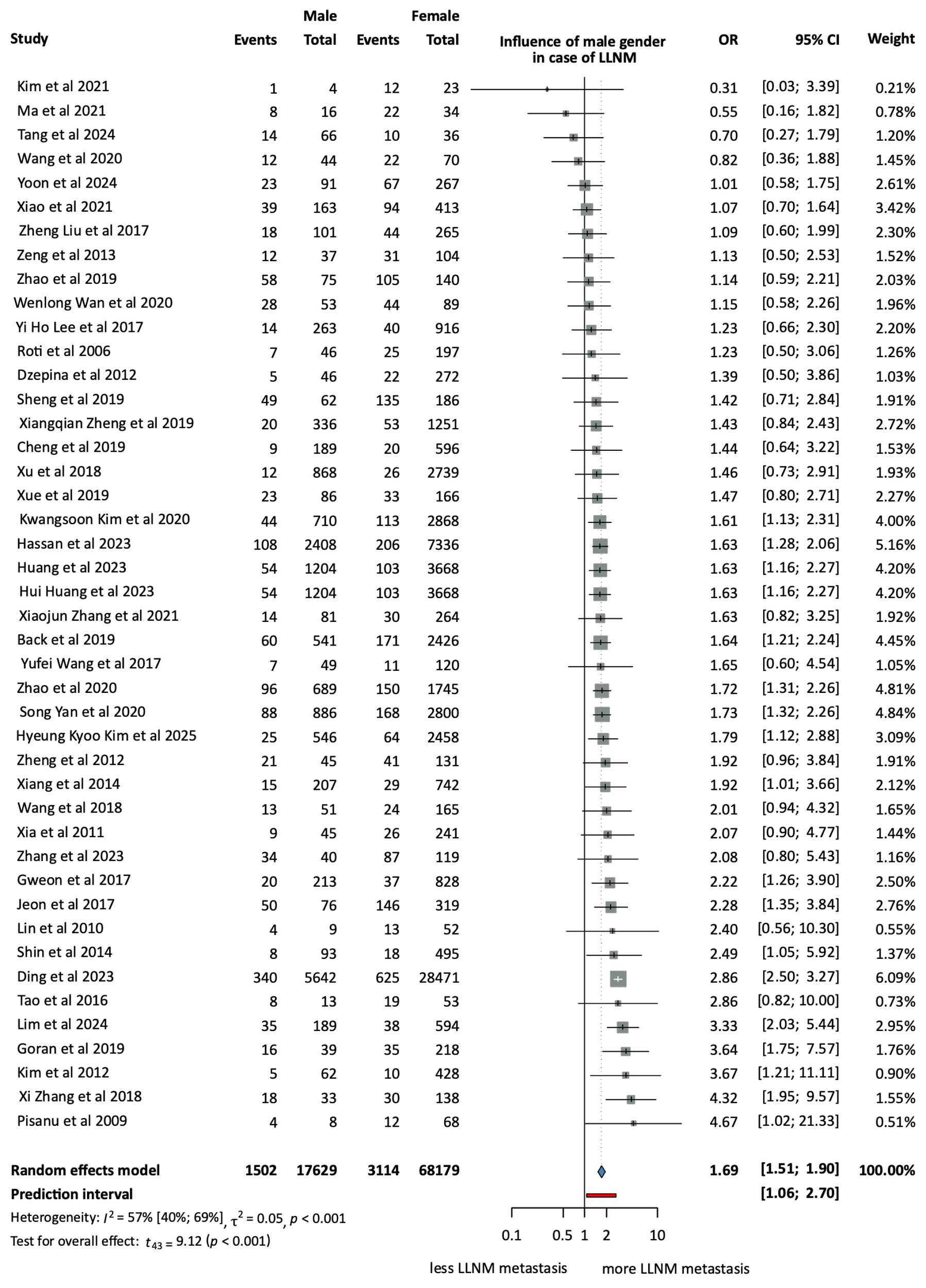


b.)


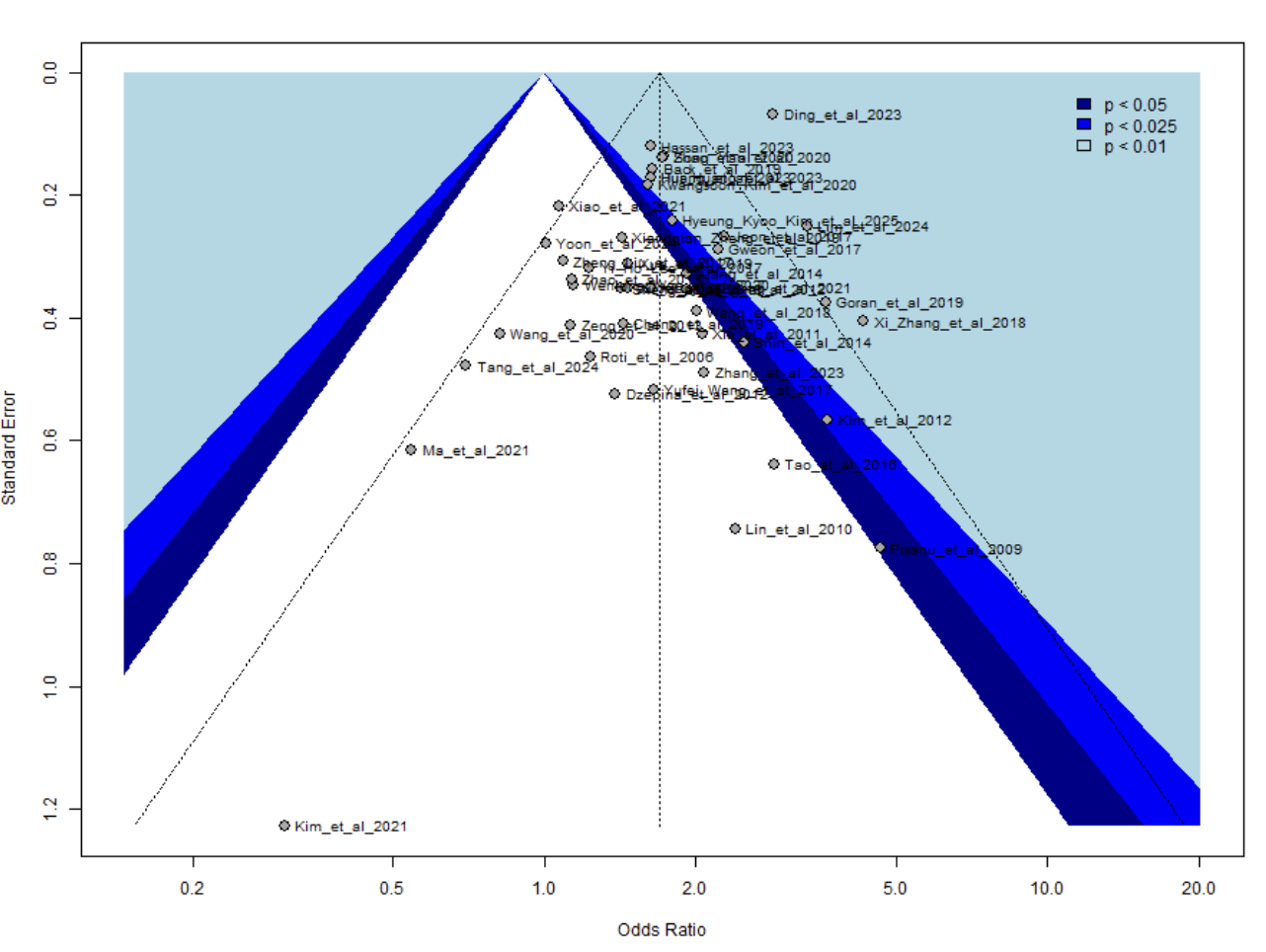


Egger’s test p= 0.0385

**Supplementary Figure 18 a-b** | Forest and funnel plots of age under 45 and its influence in case of lateral lymph node metastasis (LLNM)

a.)


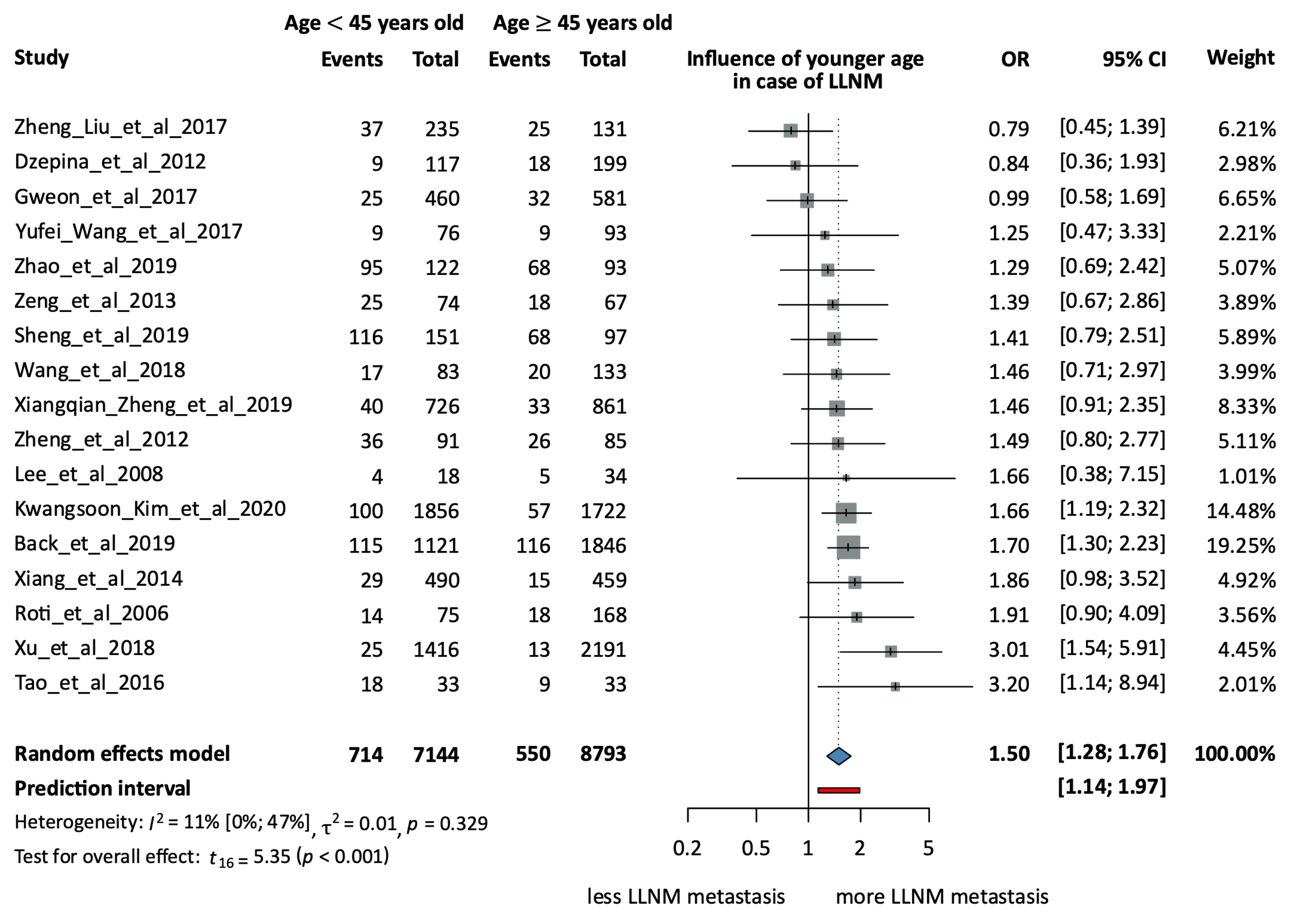


b.)


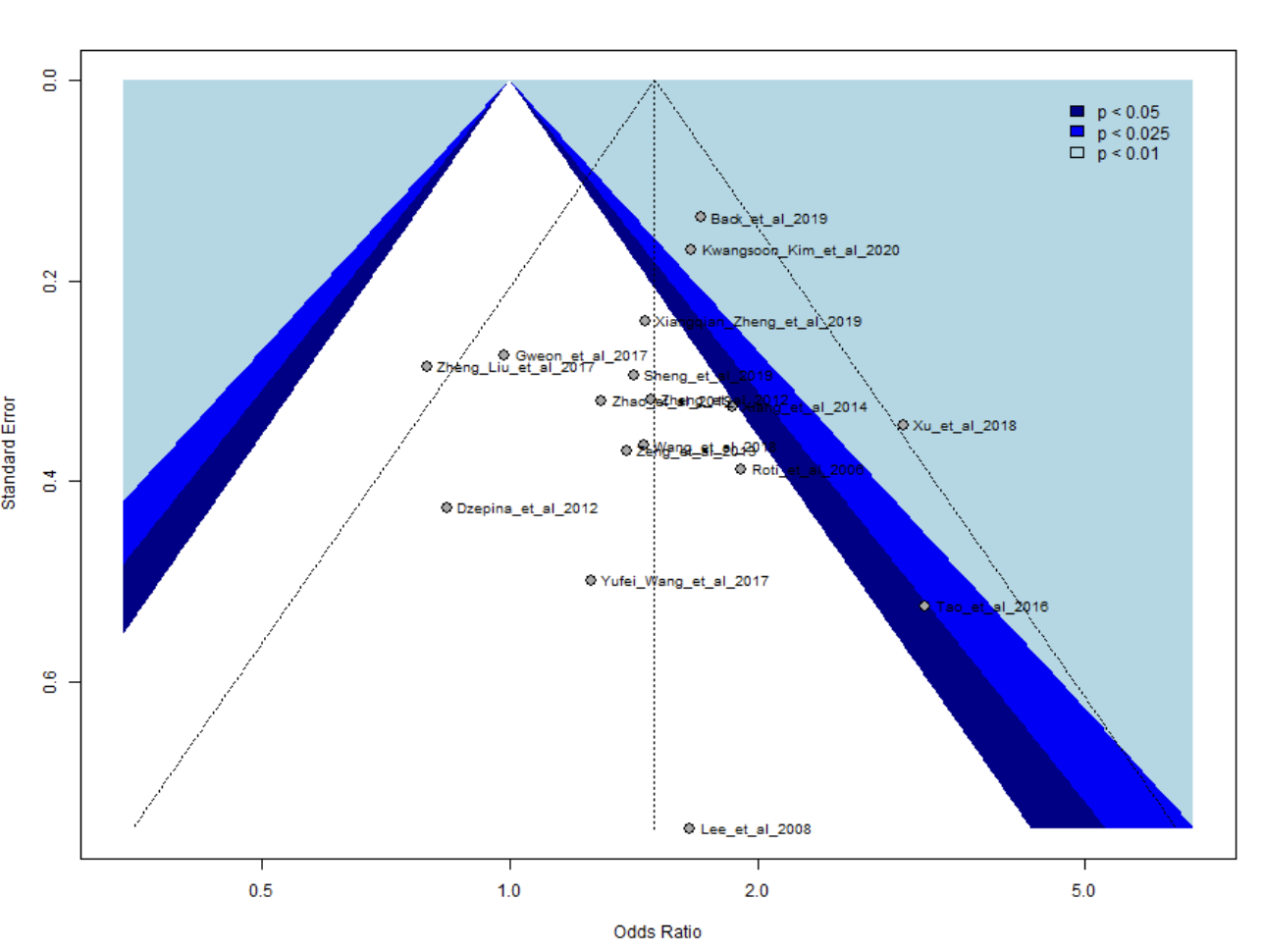


Egger’s test p= 0.9788

**Supplementary Figure 19 a-b** | Forest and funnel plots of age under 55 and its influence in the case of lateral lymph node metastasis (LLNM)

a.)


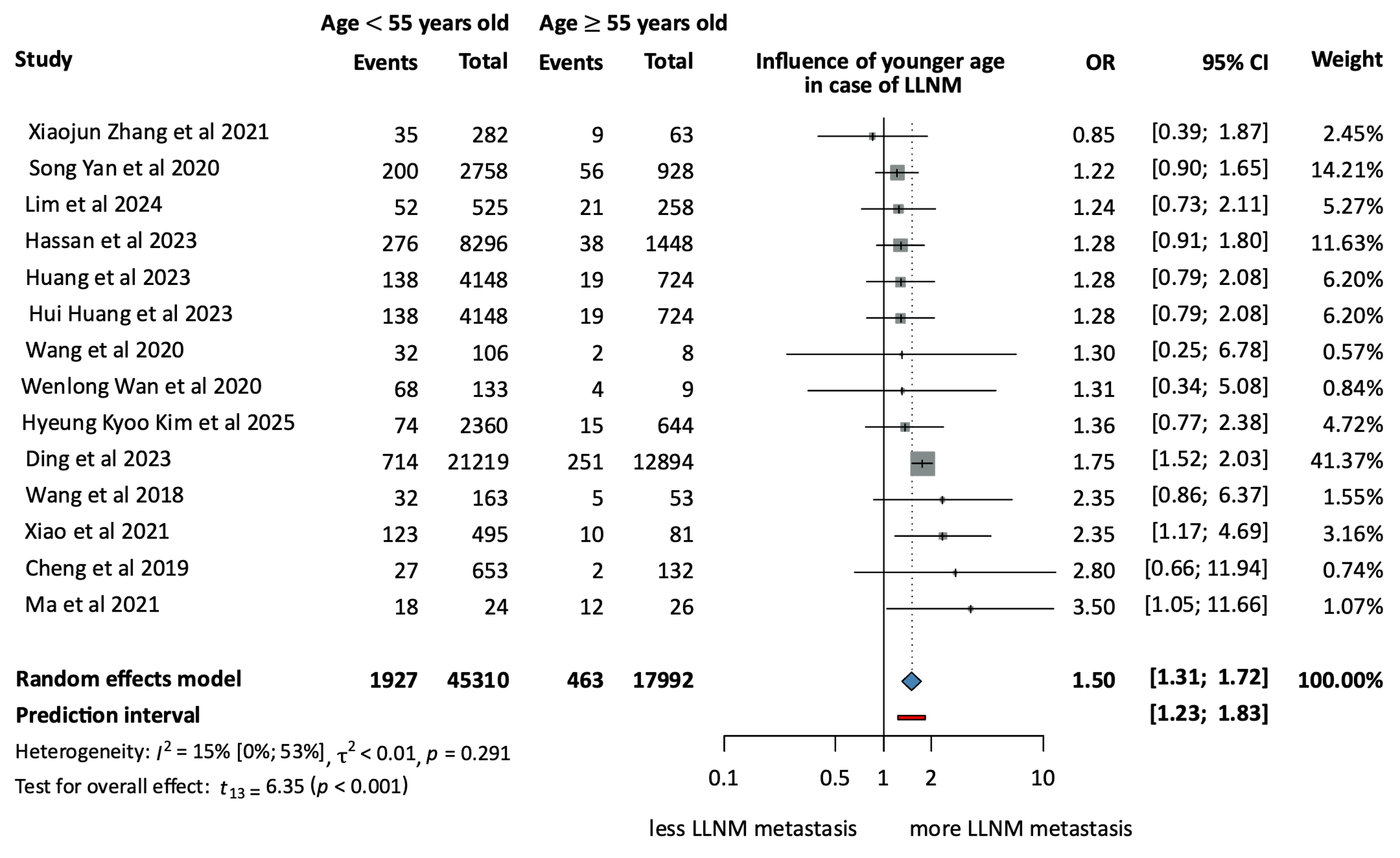


b.)


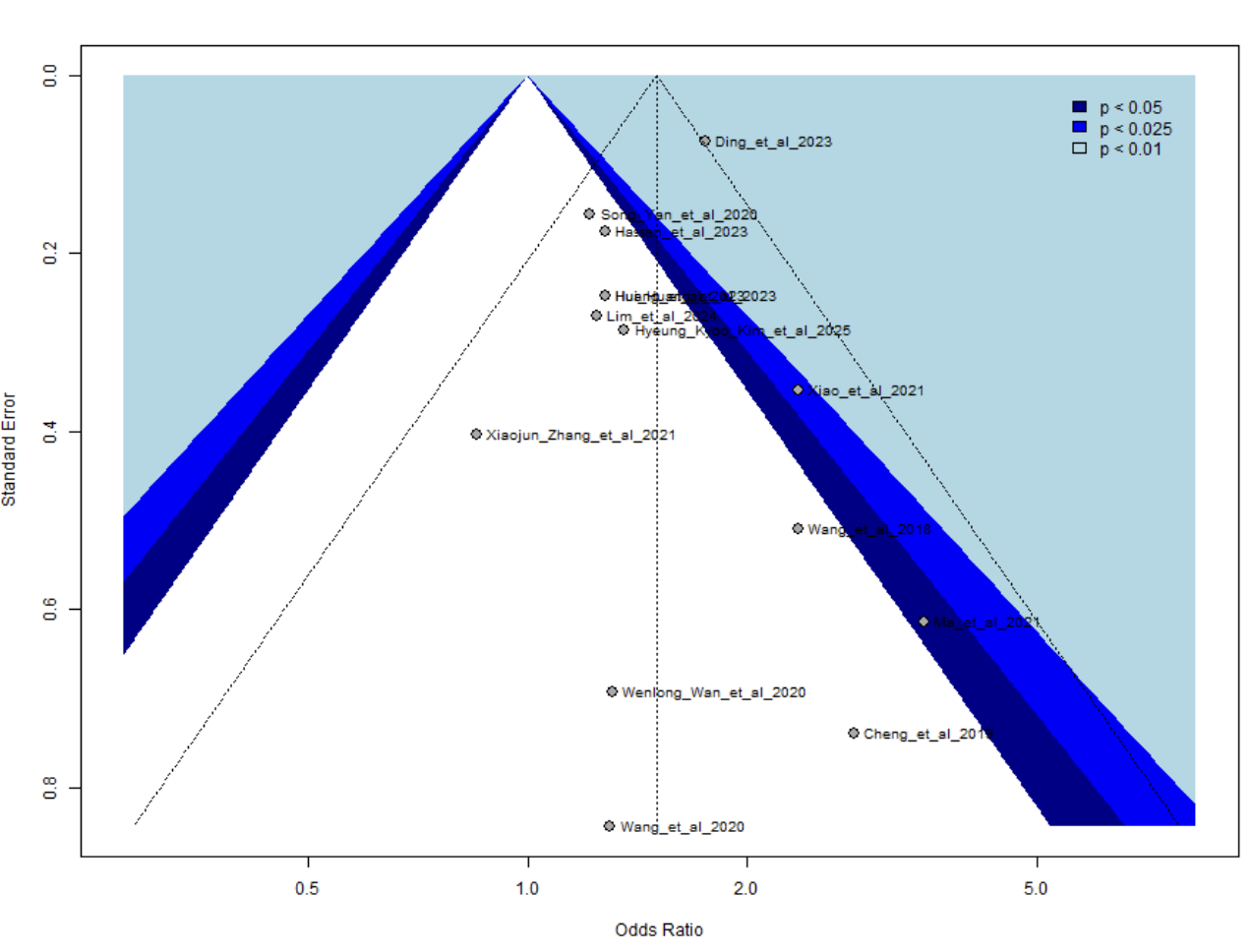


Egger’s test p= 0.5497

**Supplementary Figure 20 a-b** | Forest and funnel plots of tumor size above 5 mm and its influence in case of lateral lymph node metastasis (LLNM)

a.)


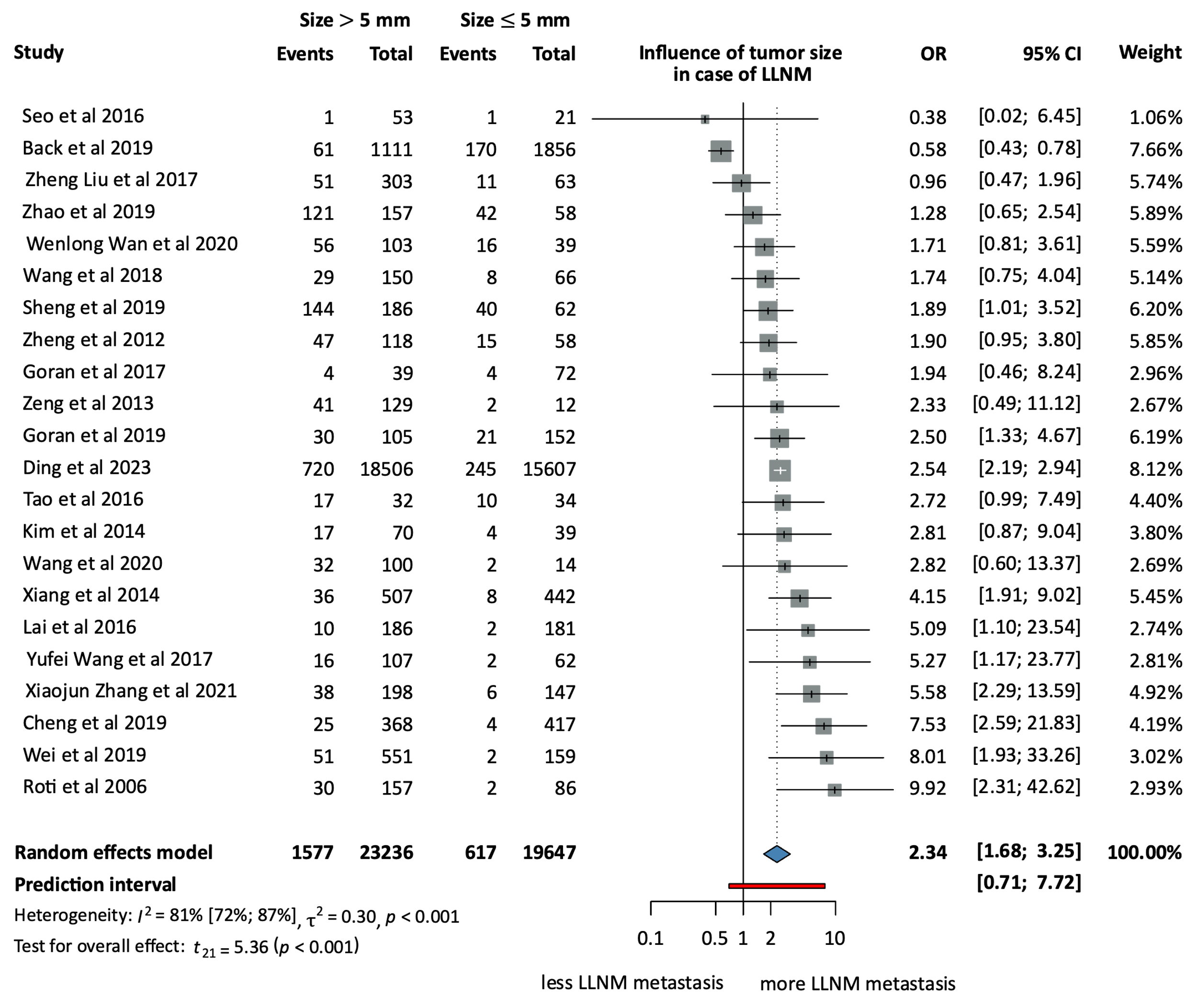


b.)


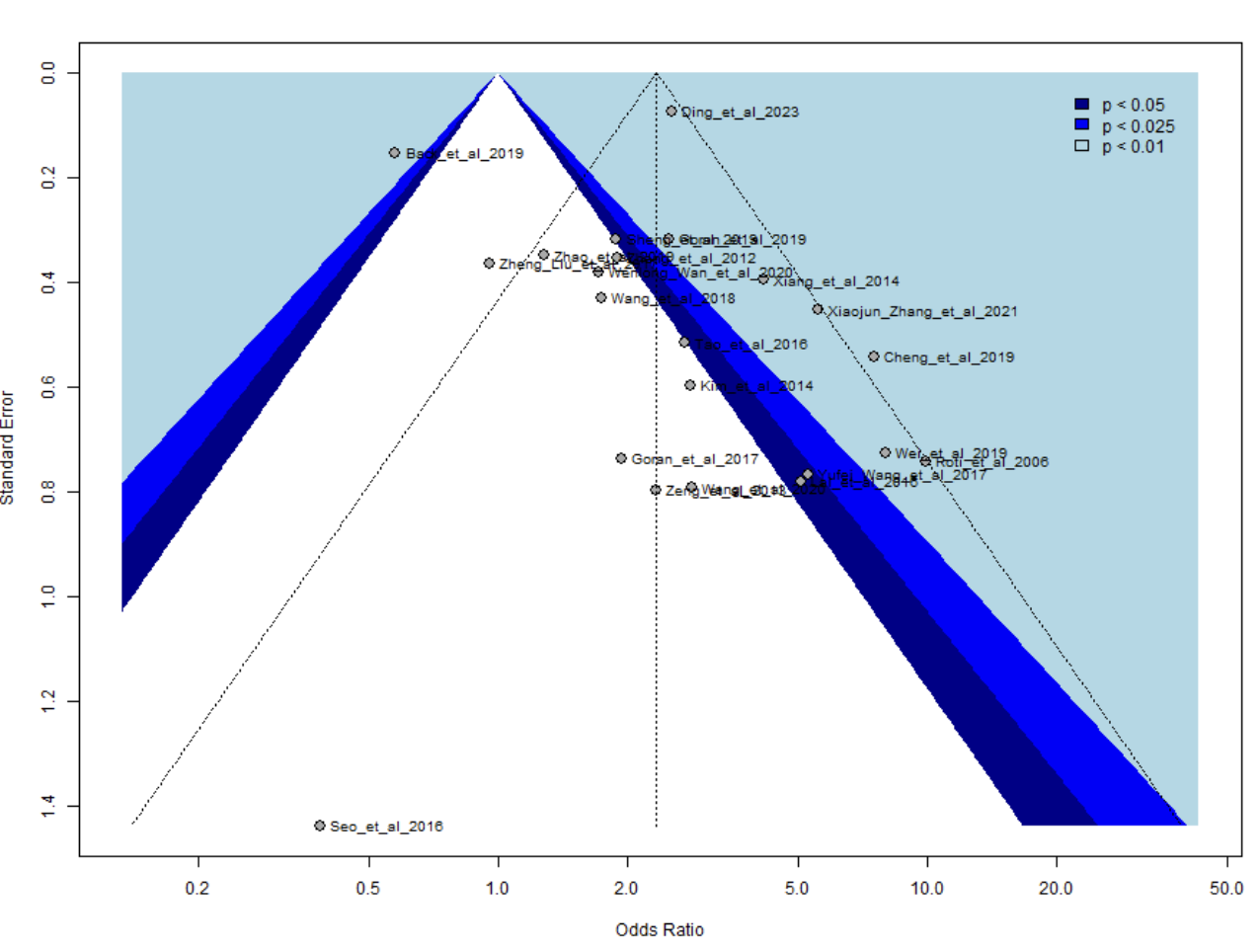


Egger’s test p= 0.7684

**Supplementary Figure 21** | Forest plot of tumor size above 7 mm and its influence in case of lateral lymph node metastasis (LLNM)


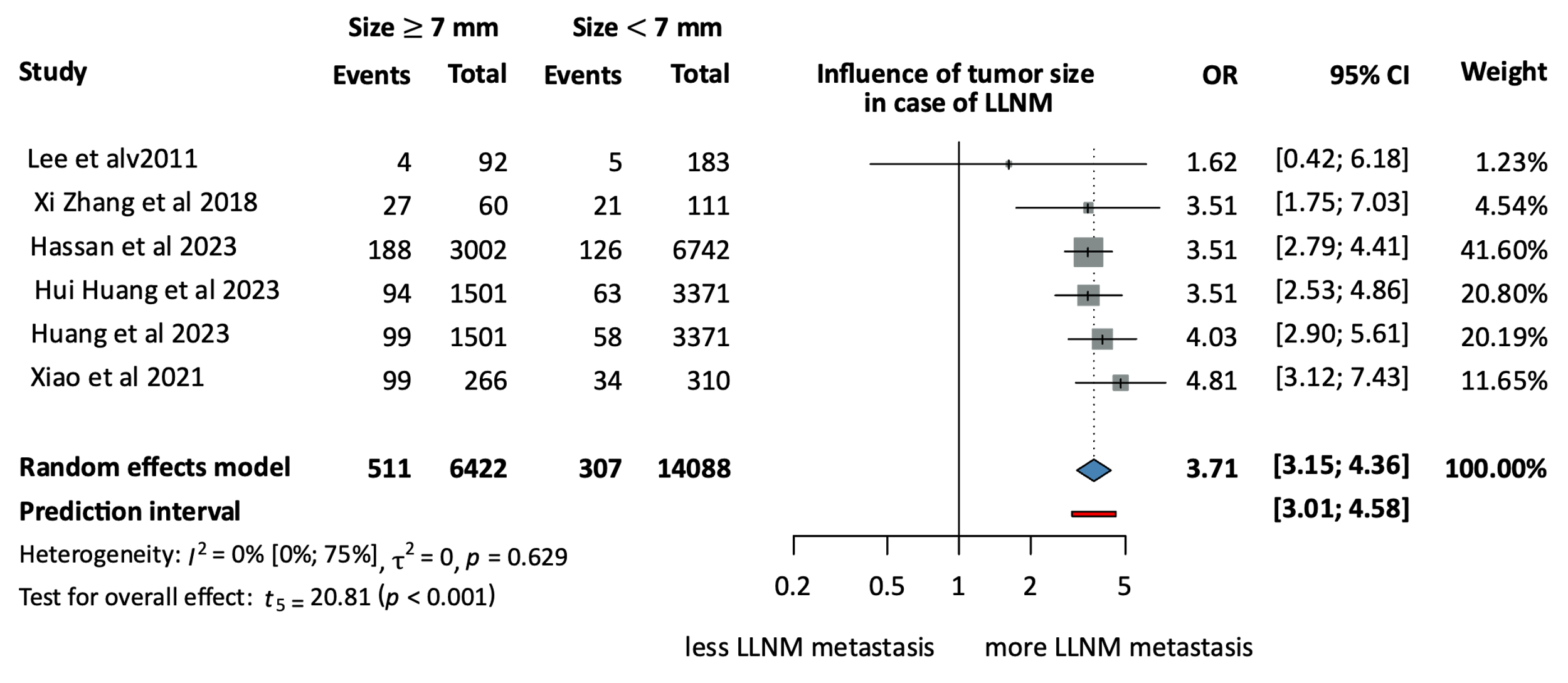


**Supplementary Figure 22 a-b** | Forest and funnel plots of multifocality and its influence in the case of lateral lymph node metastasis (LLNM)

a.)


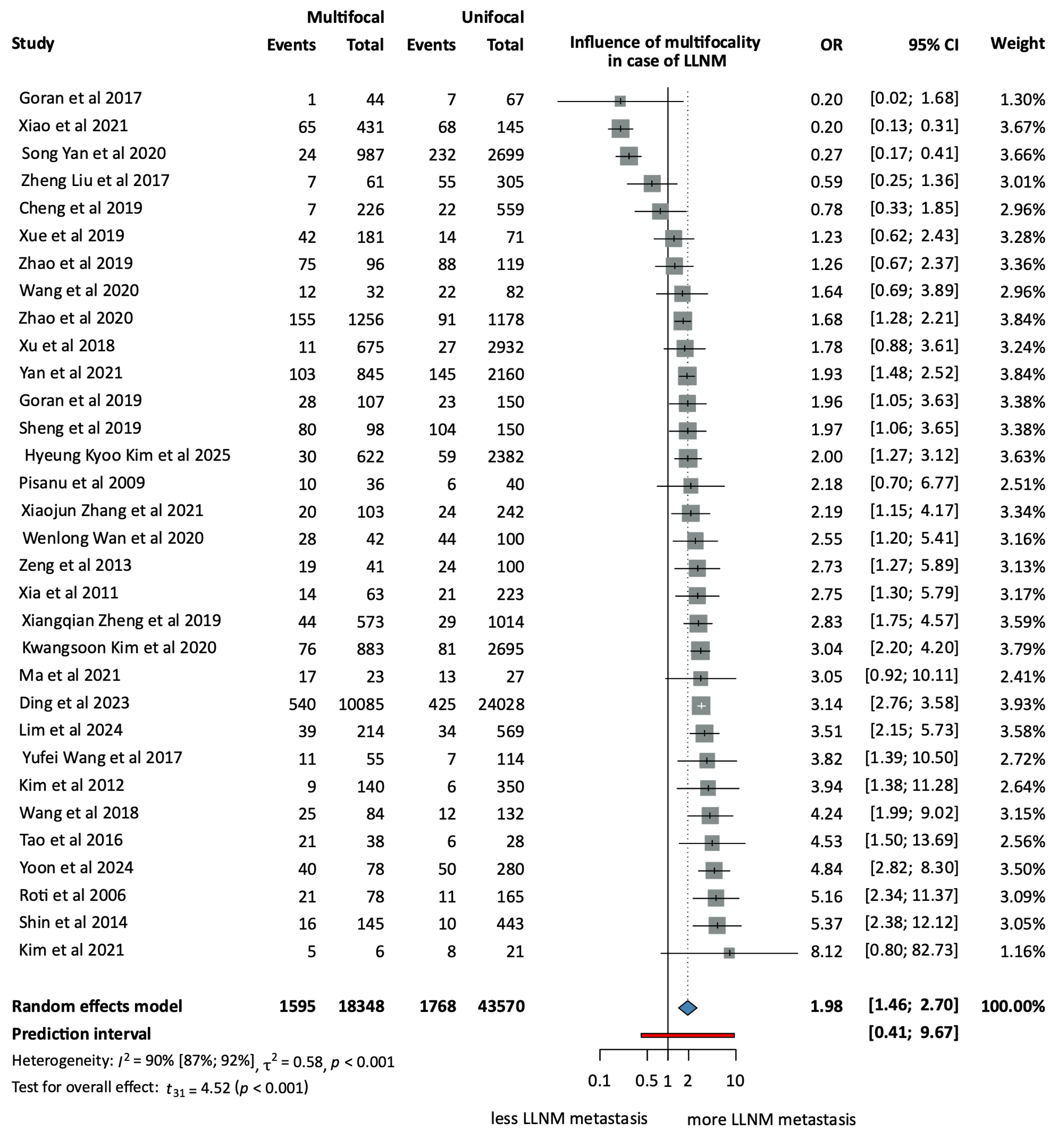


b.)


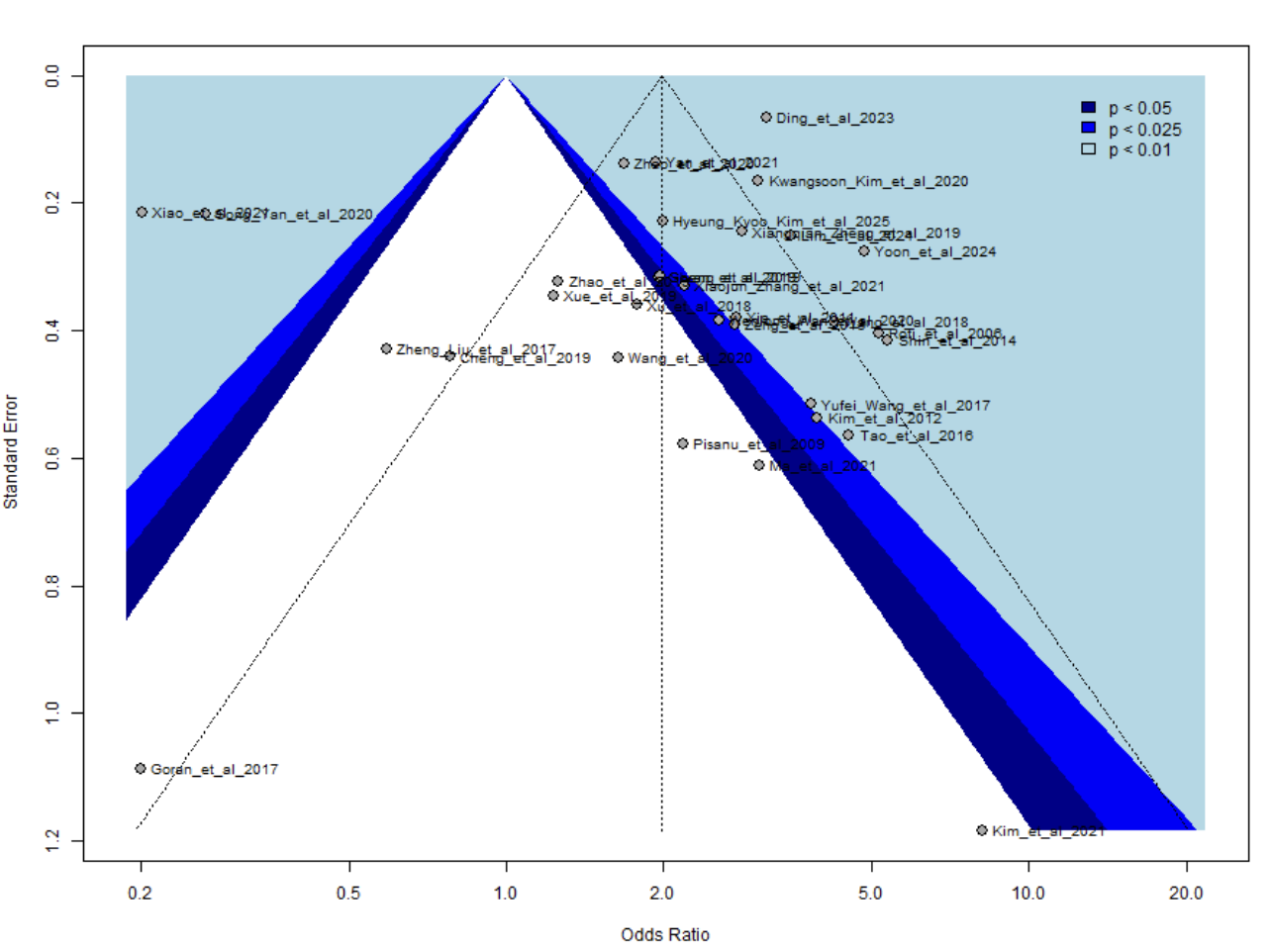


Egger’s test p= 0.7034

**Supplementary Figure 23 a-b** | Forest and funnel plots of bilaterality and its influence in the case of lateral lymph node metastasis (LLNM)

a.)


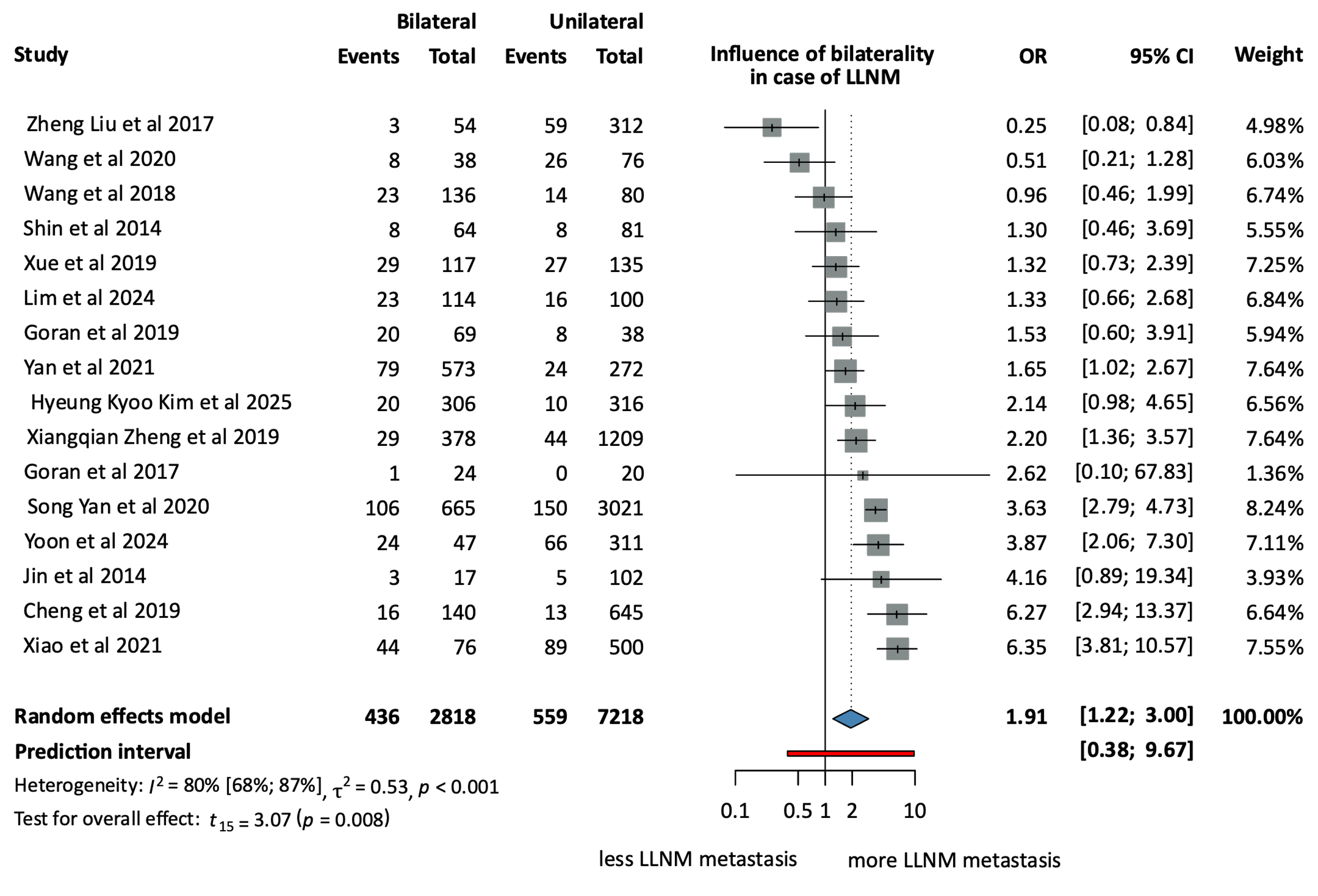


b.)


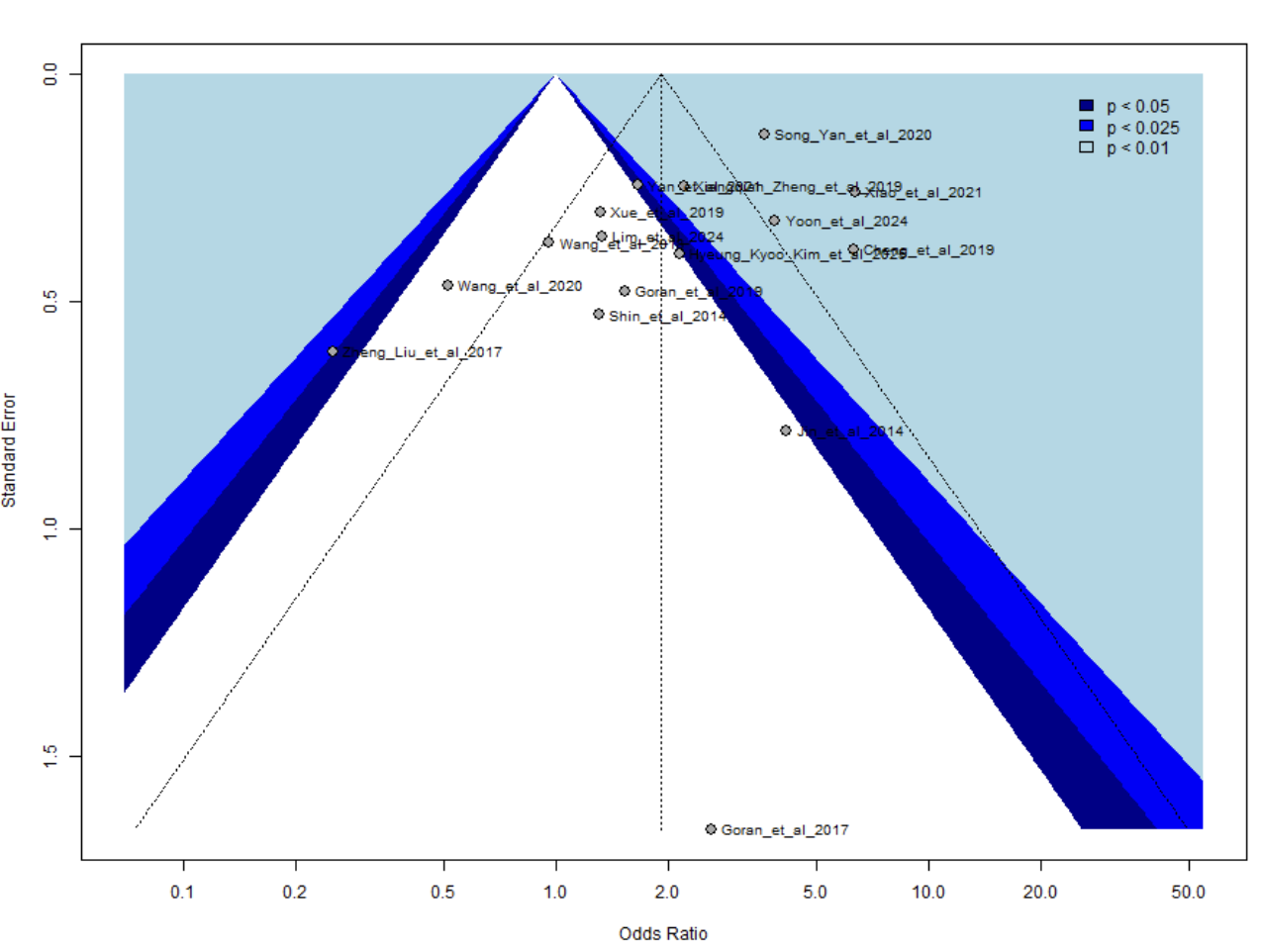


Egger’s test p= 0.0848

**Supplementary Figure 24 a-b** | Forest and funnel plots of BRAF^V600E^ mutation and its influence in case of lateral lymph node metastasis (LLNM)

a.)


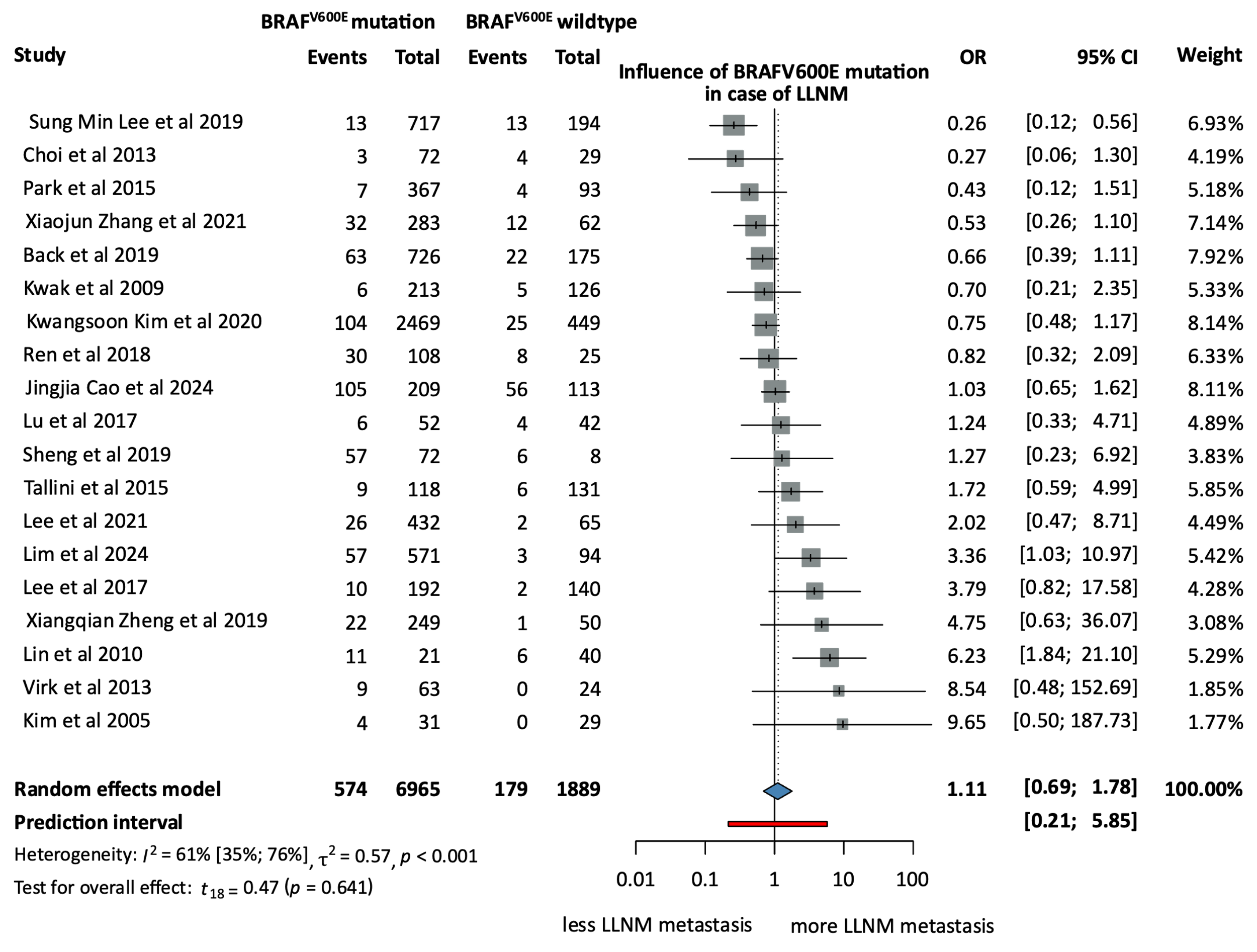


b.)


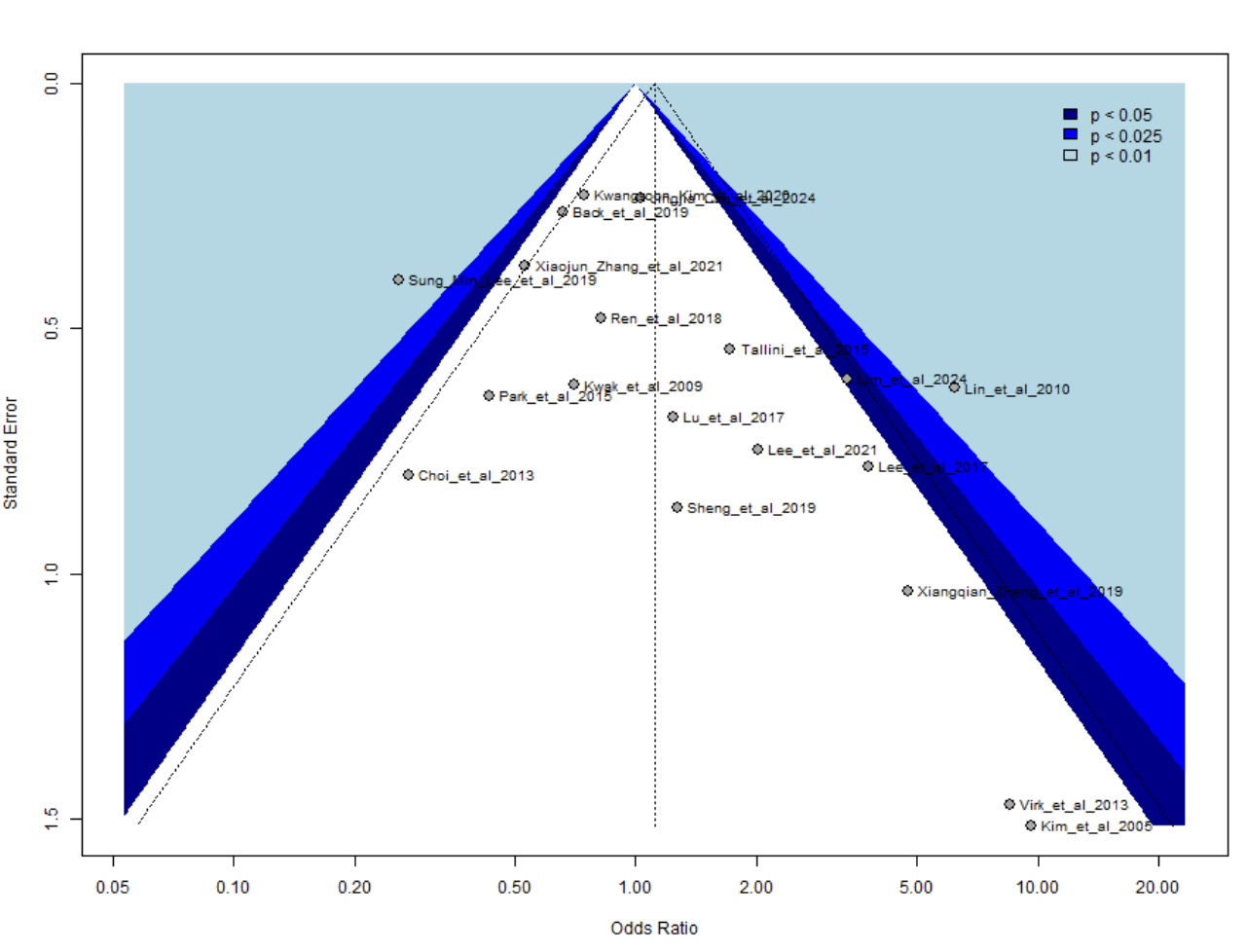


Egger’s test p= 0.0957

**Supplementary Figure 25 a-b** | Forest and funnel plots of Hashimoto’s thyroiditis and its influence in case of lateral lymph node metastasis (LLNM)

a.)


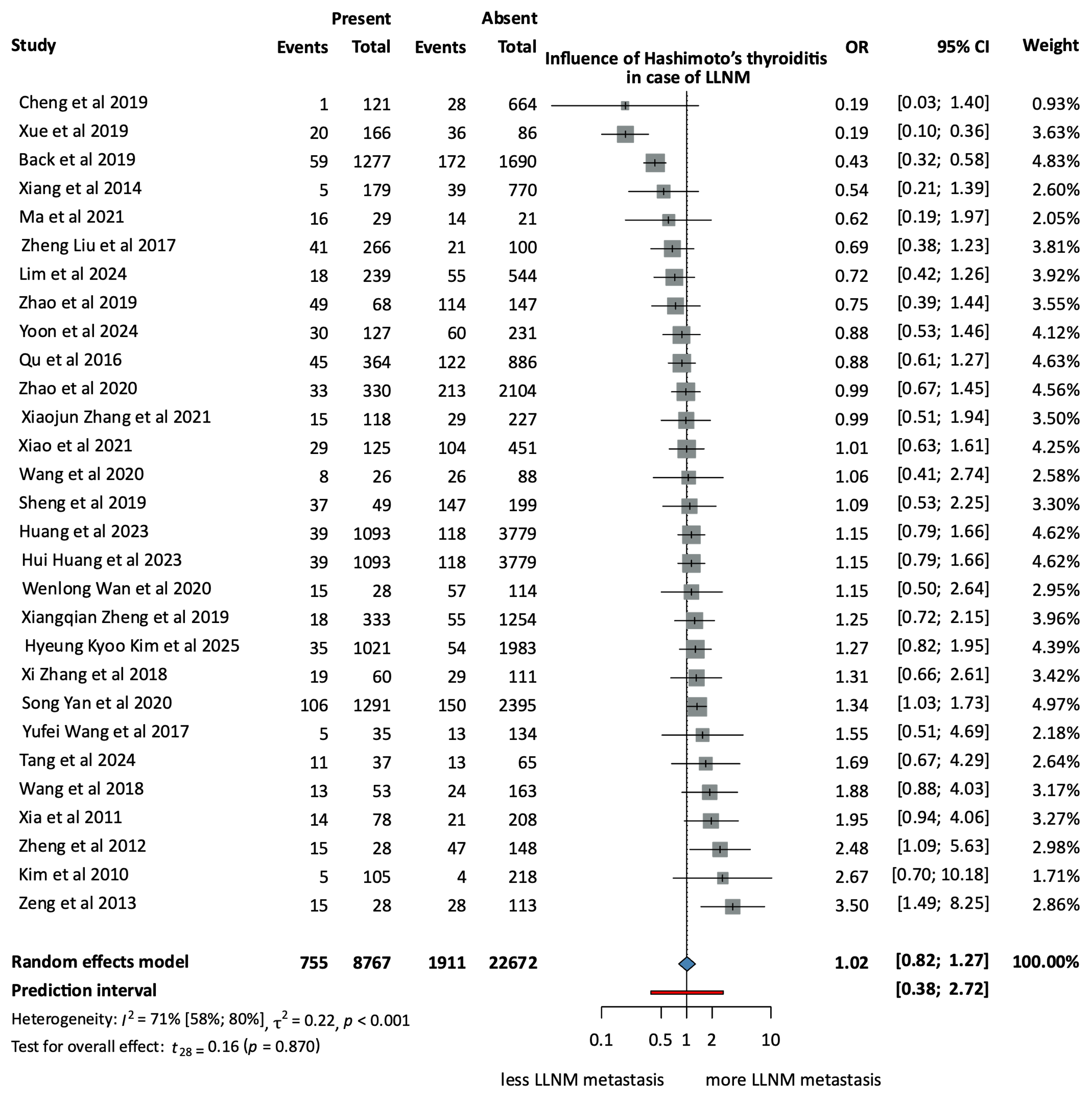


b.)


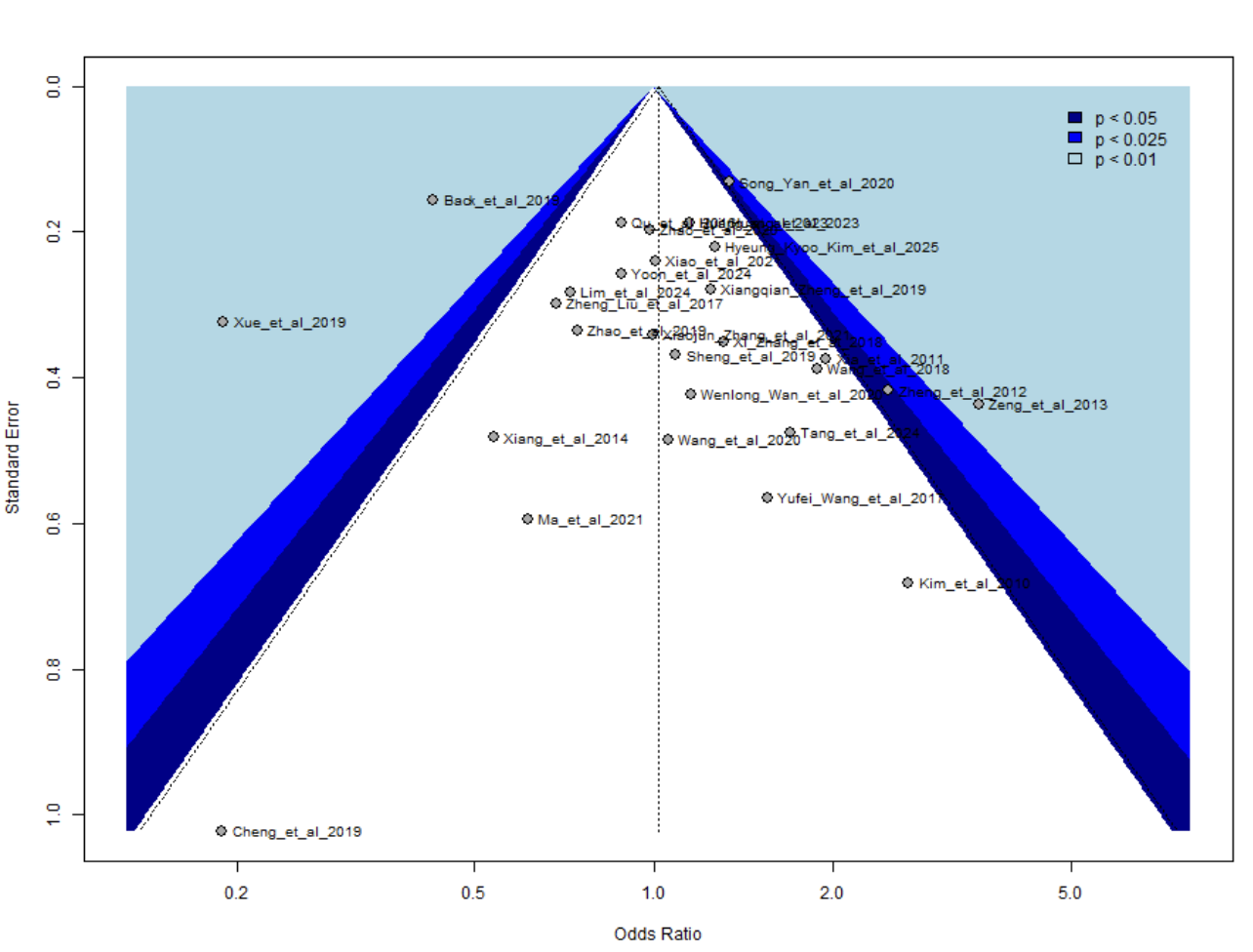


Egger’s test p= 0.4167

**Supplementary Figure 26** | Forest and funnel plot of microcalcification and its influence in case of lateral lymph node metastasis (LLNM)


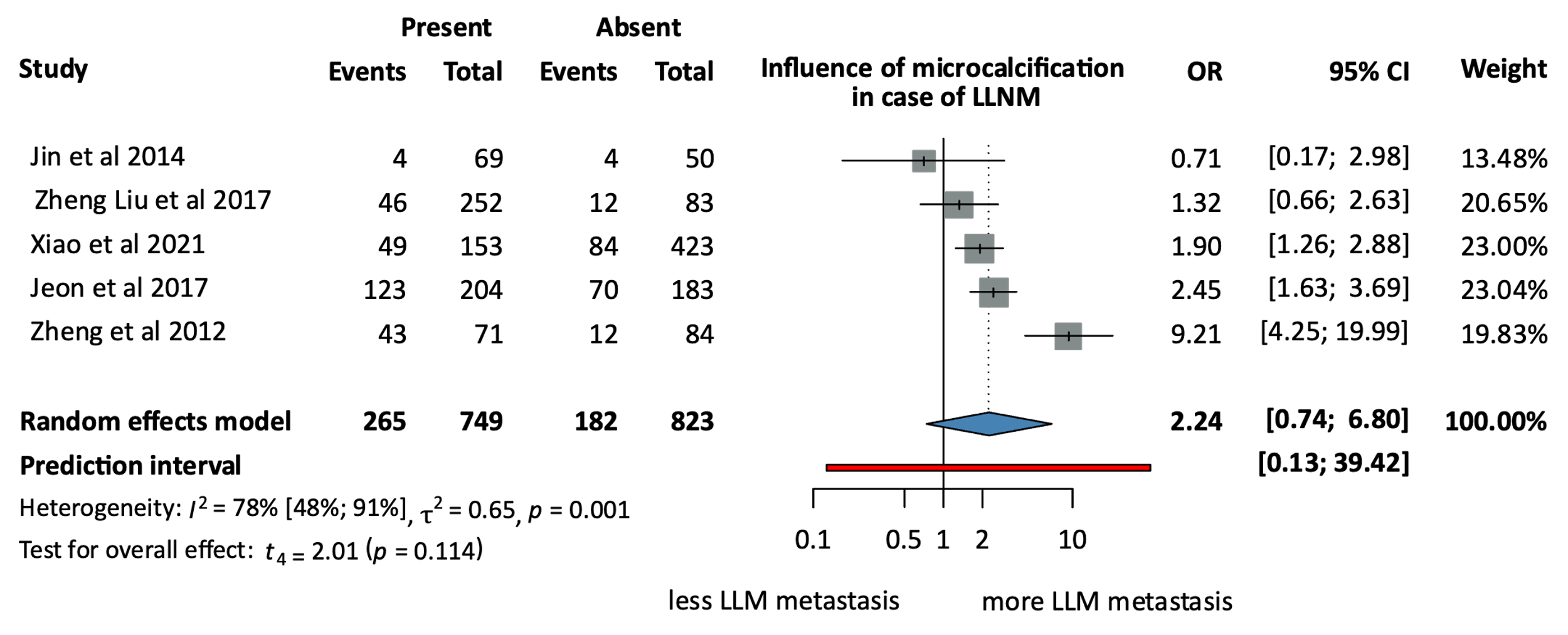


**Supplementary Figure 27** | Forest and funnel plot of goiter and its influence in case of lateral lymph node metastasis (LLNM)


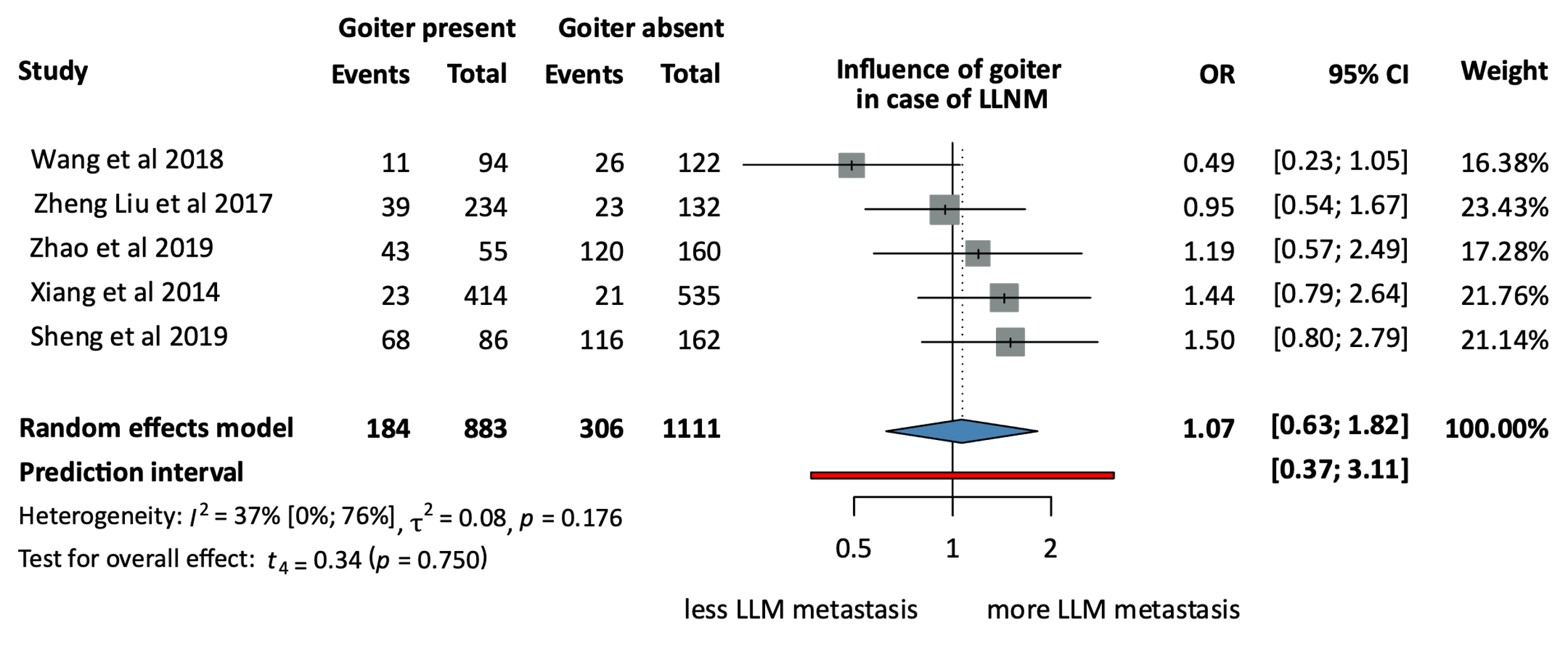


**Supplementary Figure 28** **a-b** | Forest and funnel plots of capsule invasion and its influence in case of lateral lymph node metastasis (LLNM)

a.)


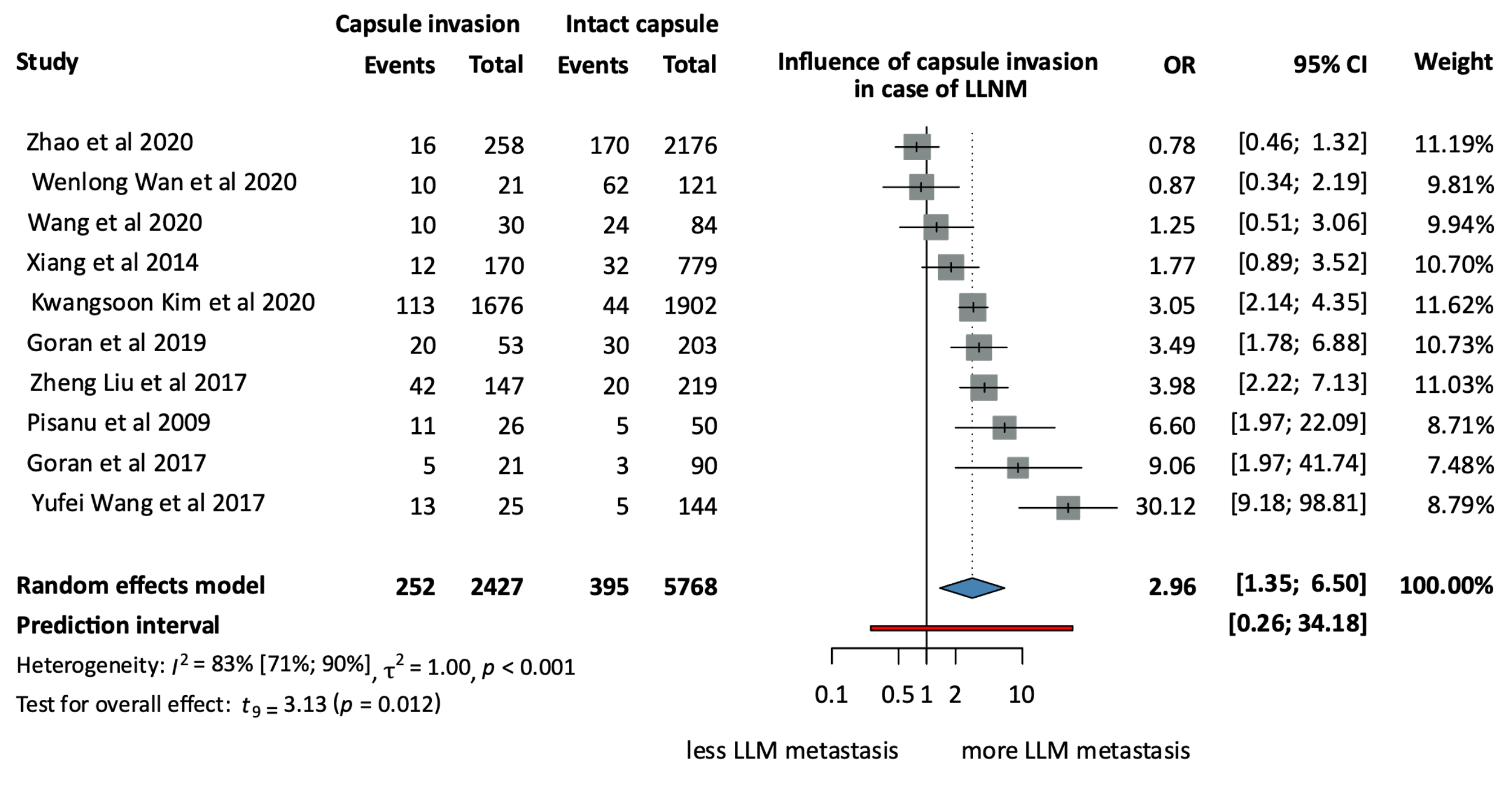


b.)


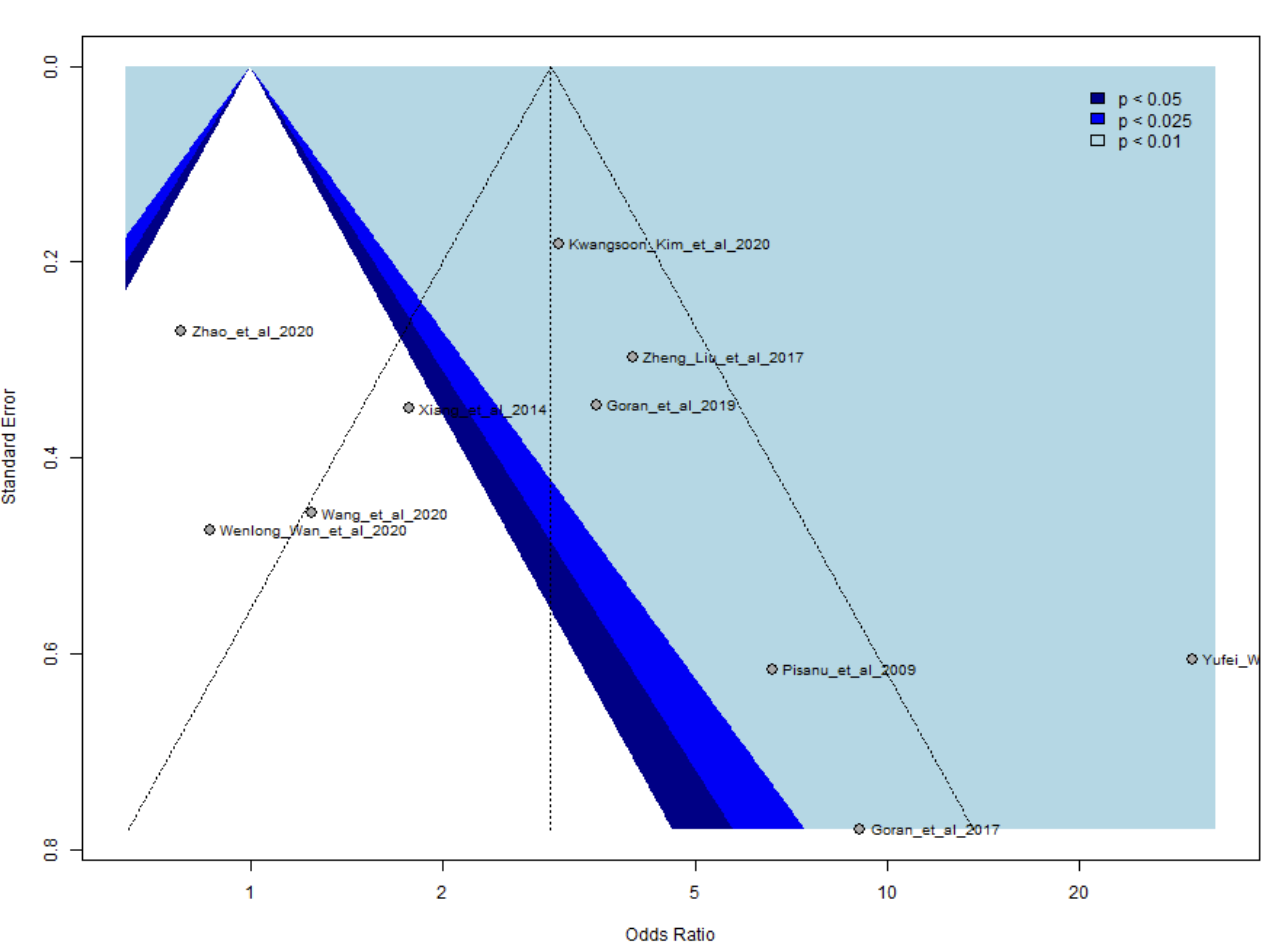


Egger’s test p= 0.4188

**Supplementary Figure 29 a-b** | Forest and funnel plots of male sex and its influence in the case of undetermined lymph node metastasis (udLNM)

a.)


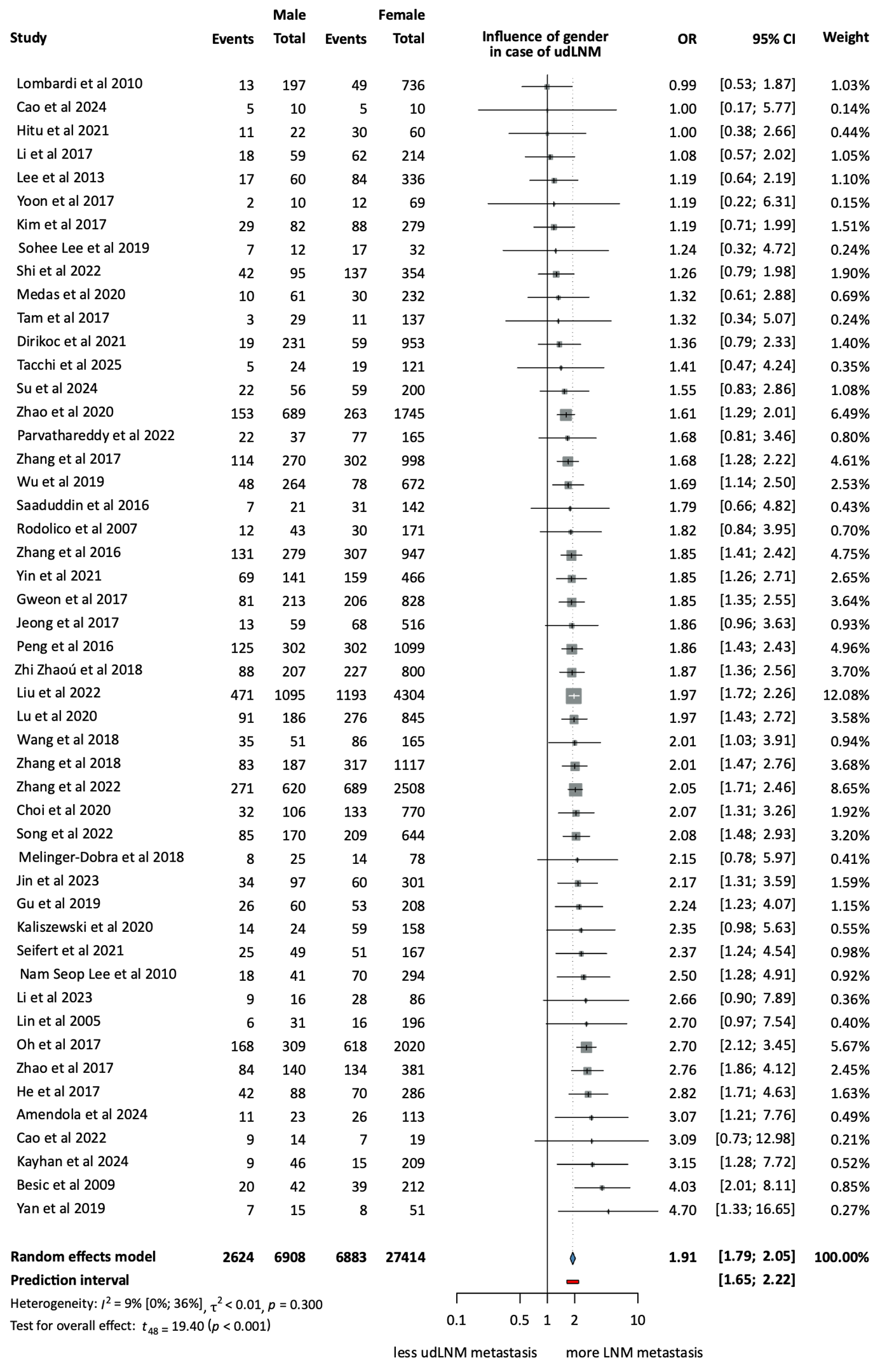


b.)


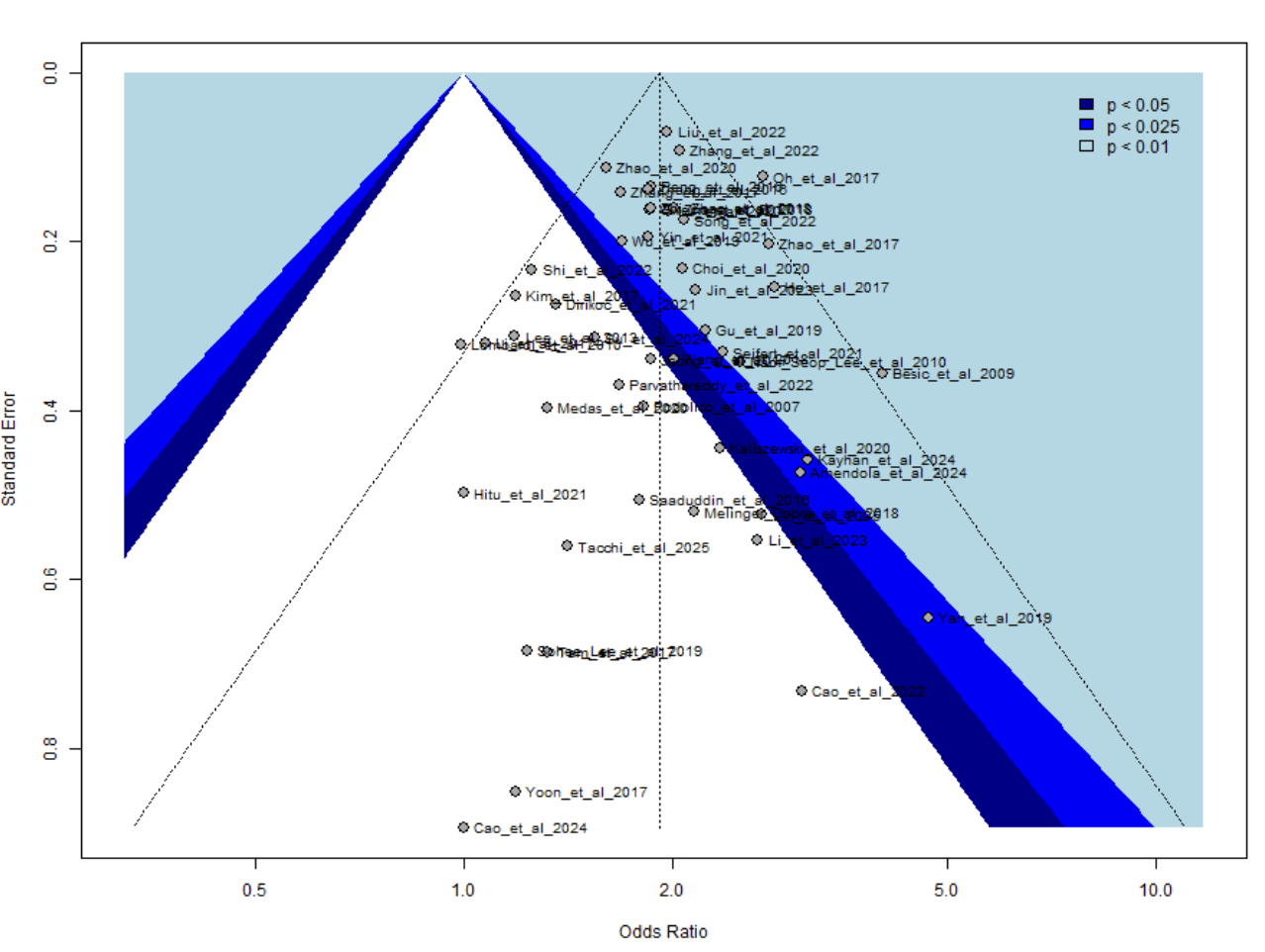


Egger’s test p= 0.6796

**Supplementary Figure 30 a-b** | Forest and funnel plots of age under 45 and its influence in the case of undetermined lymph node metastasis (udLNM)

a.)


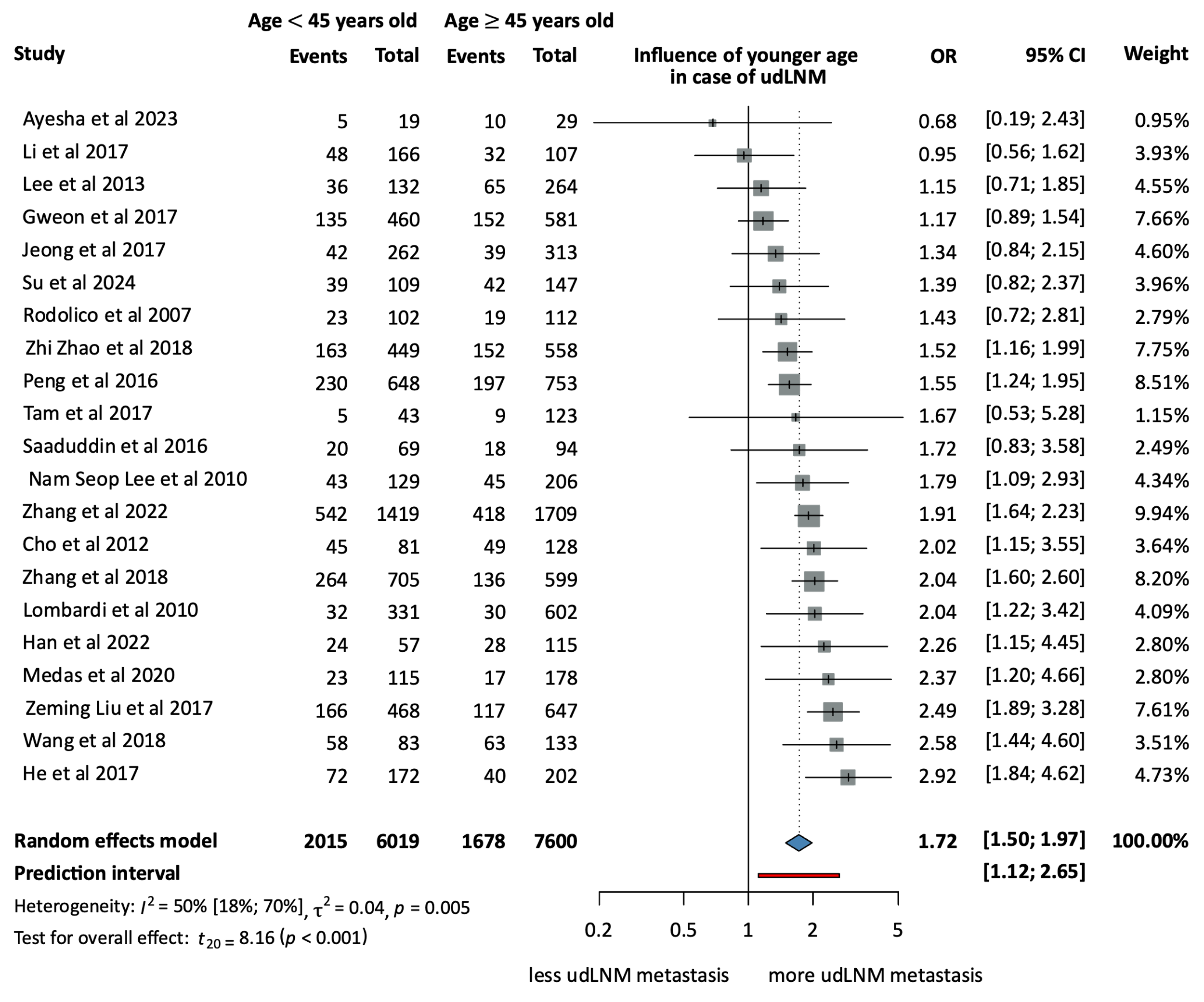


b.)


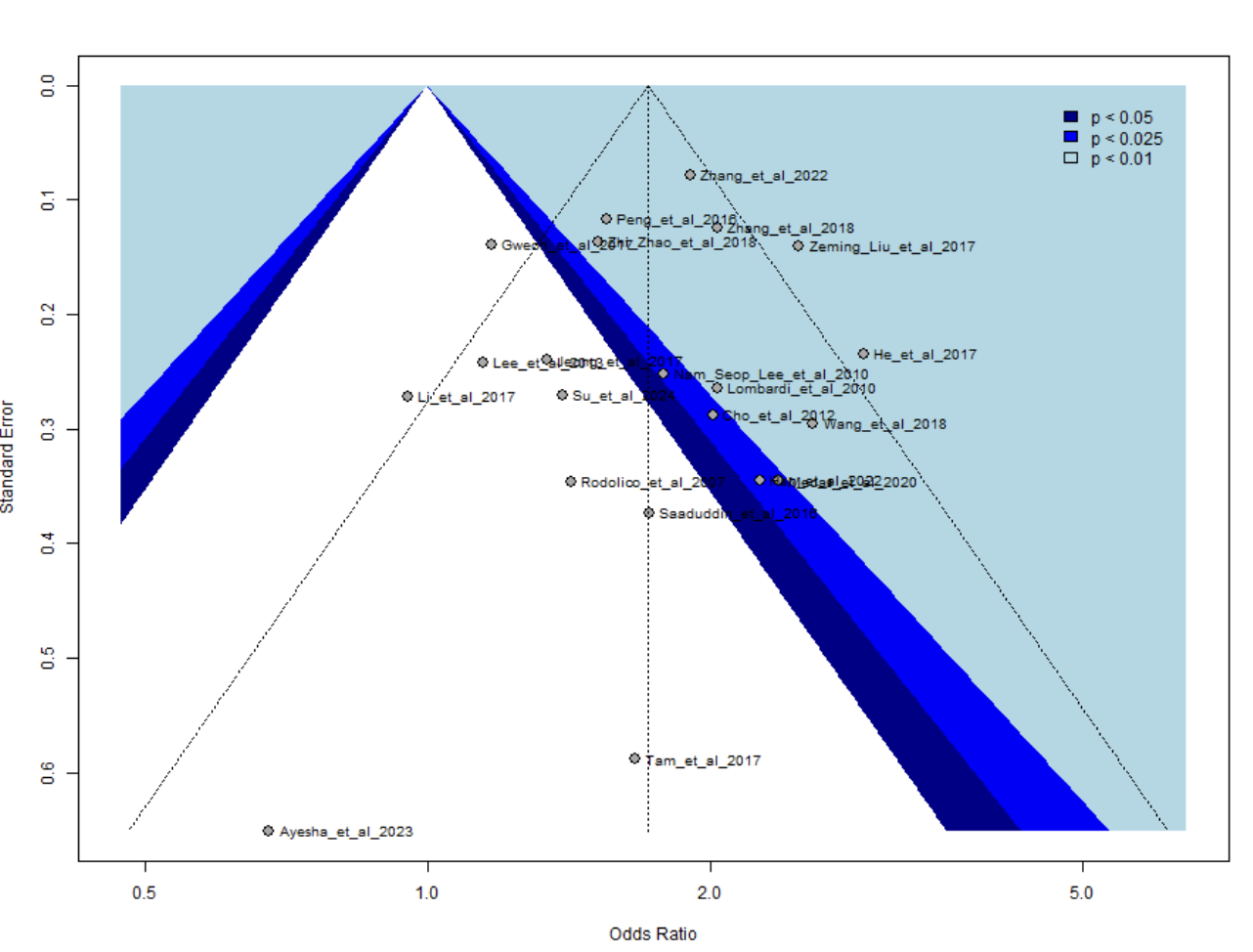


Egger’s test p= 0.4482

**Supplementary Figure 31 a-b** | Forest and funnel plots of under 55 and its influence in the case of undetermined lymph node metastasis (udLNM)

a.)


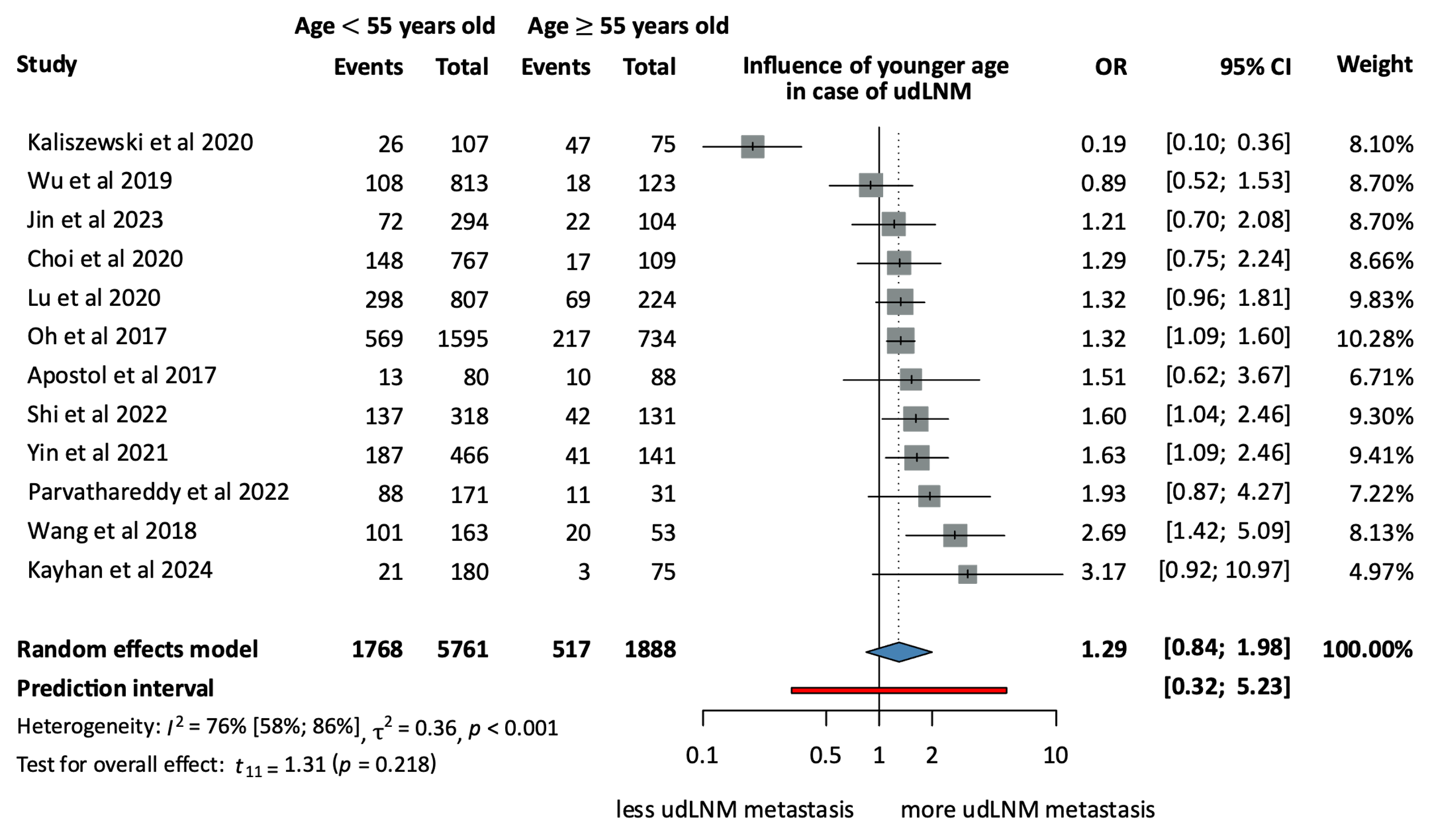


b.)


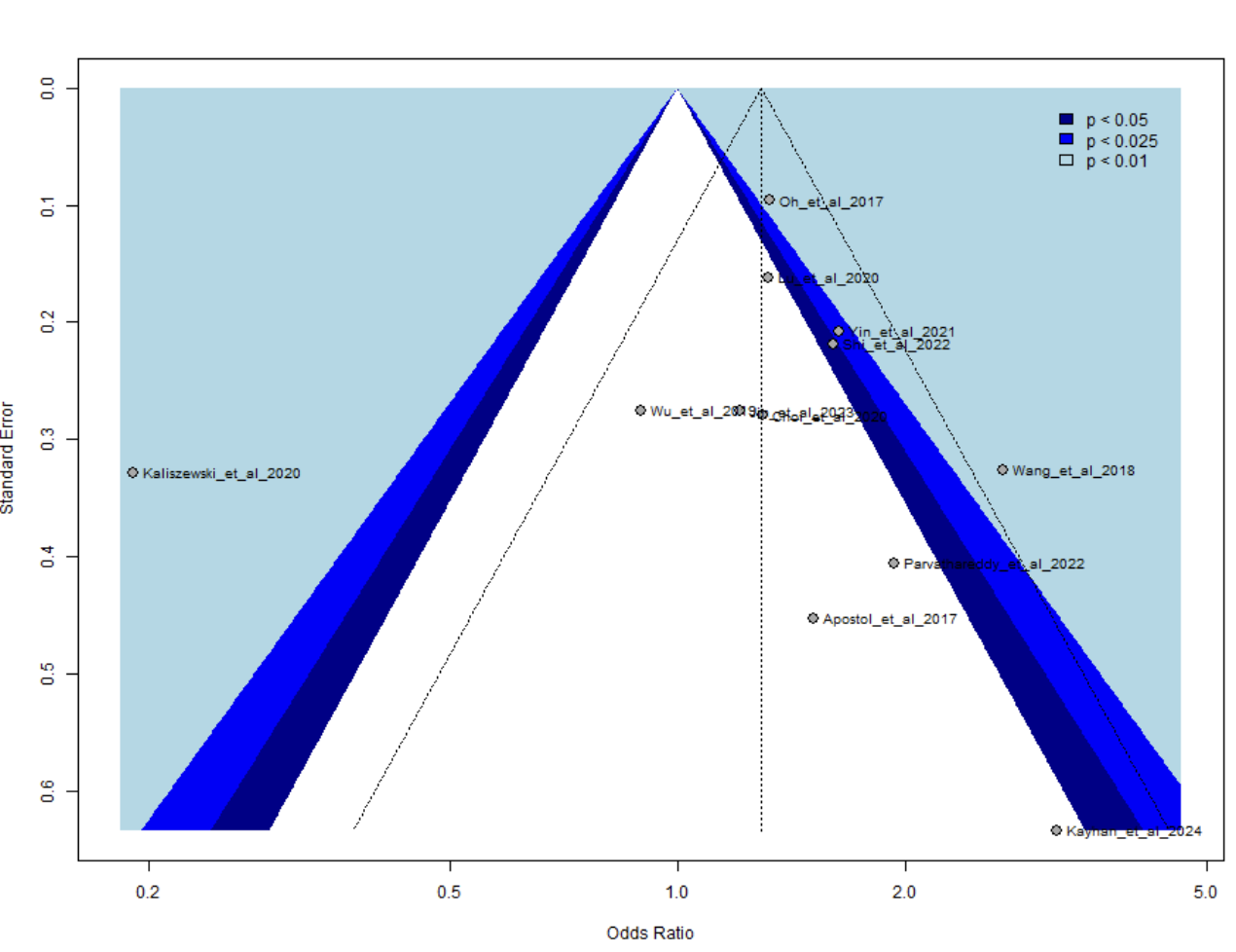


Egger’s test p= 0.8271

**Supplementary Figure 32 a-b** | Forest and funnel plots of tumor size above 5 mm and its influence in the case of undetermined lymph node metastasis (udLNM)

a.)


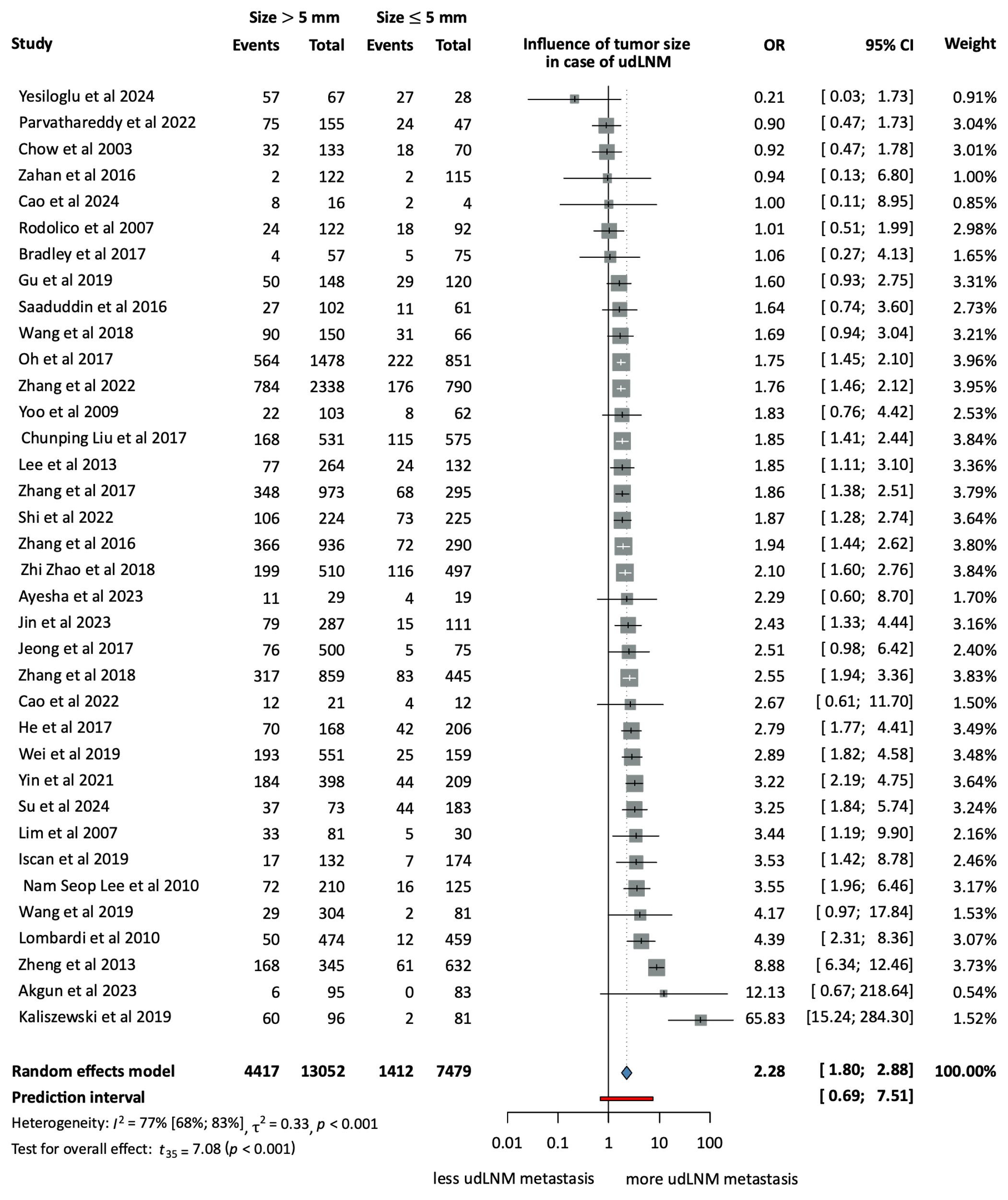


b.)


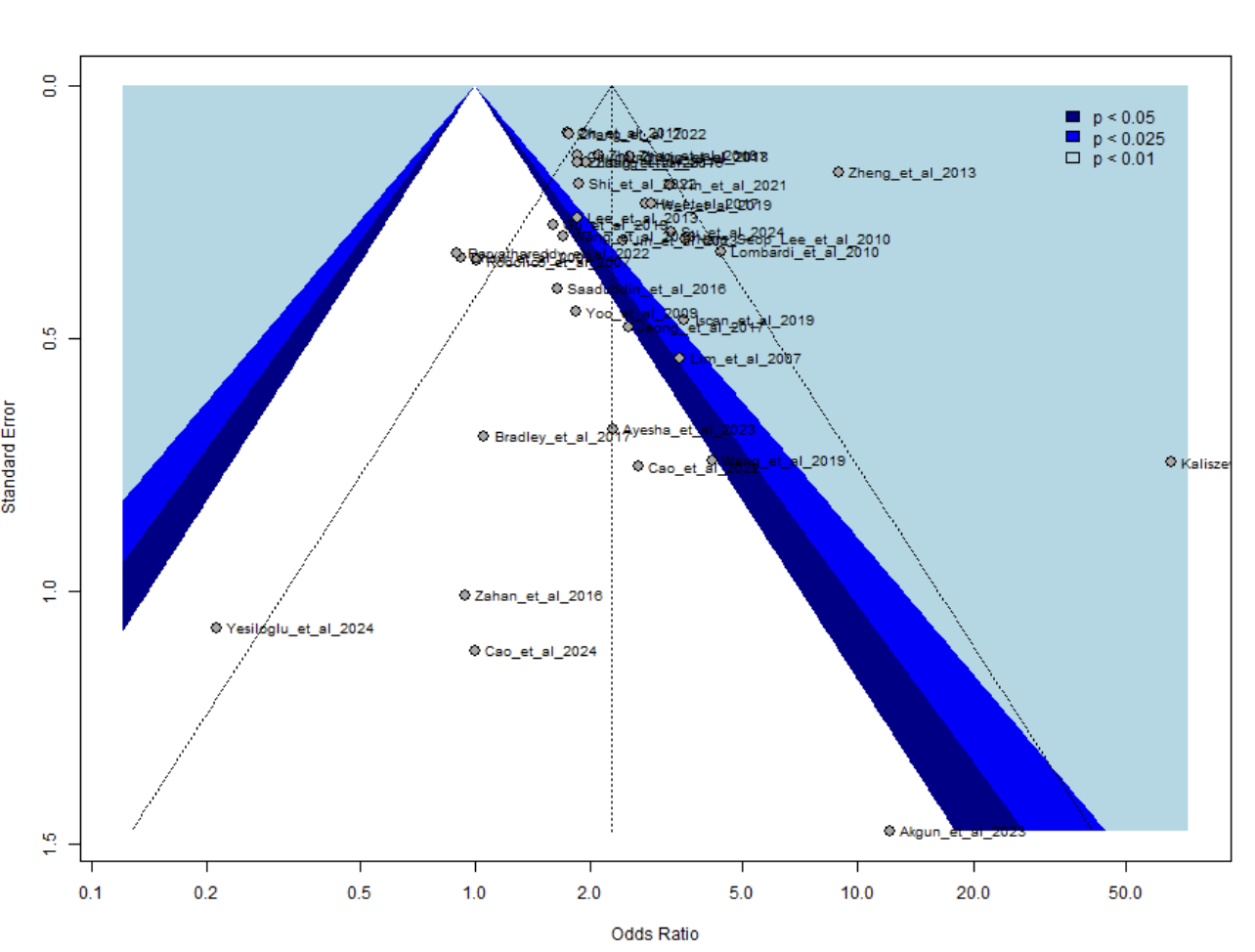


Egger’s test p= 0.8771

**Supplementary Figure 33** | Forest plot of tumor size above 6 mm and its influence in the case of undetermined lymph node metastasis (udLNM)

a.)


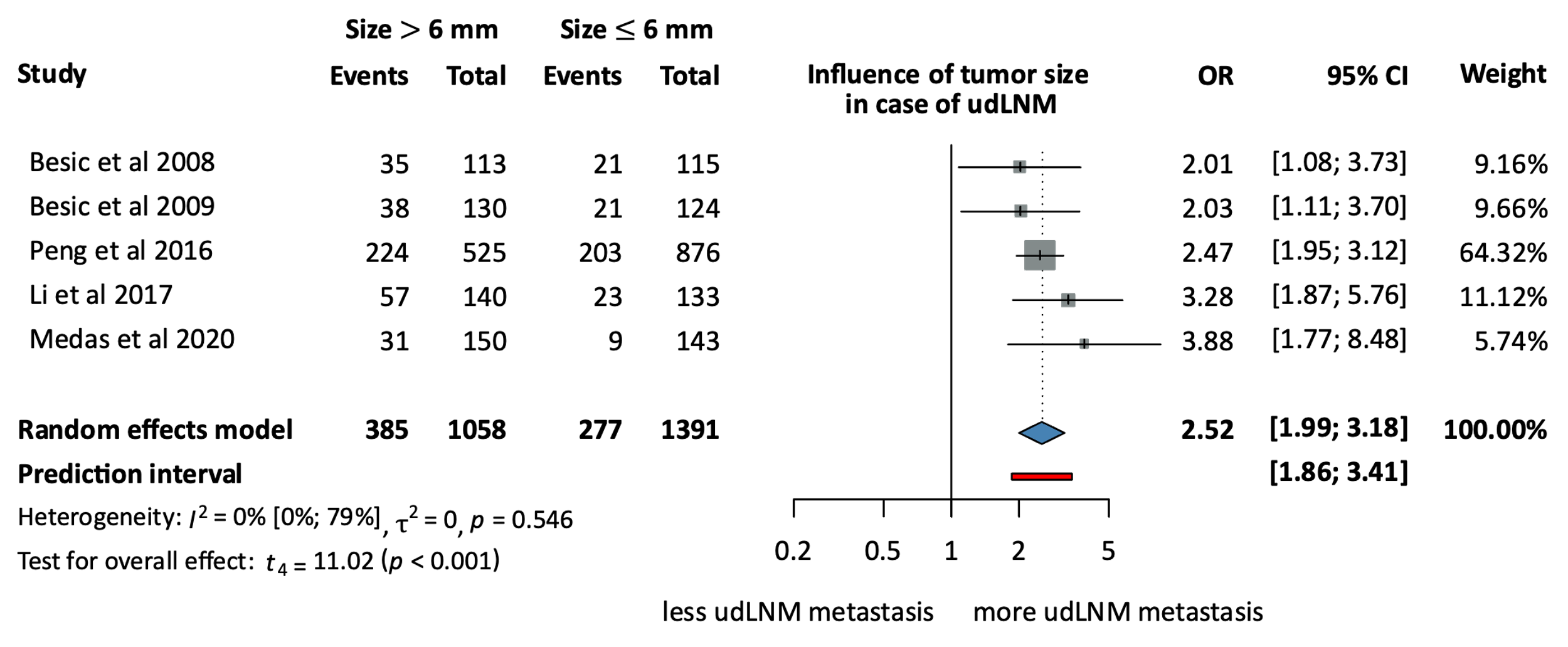


**Supplementary Figure 34 a-b** | Forest and funnel plots of multifocality and its influence in the case of undetermined lymph node metastasis (udLNM)

a.)


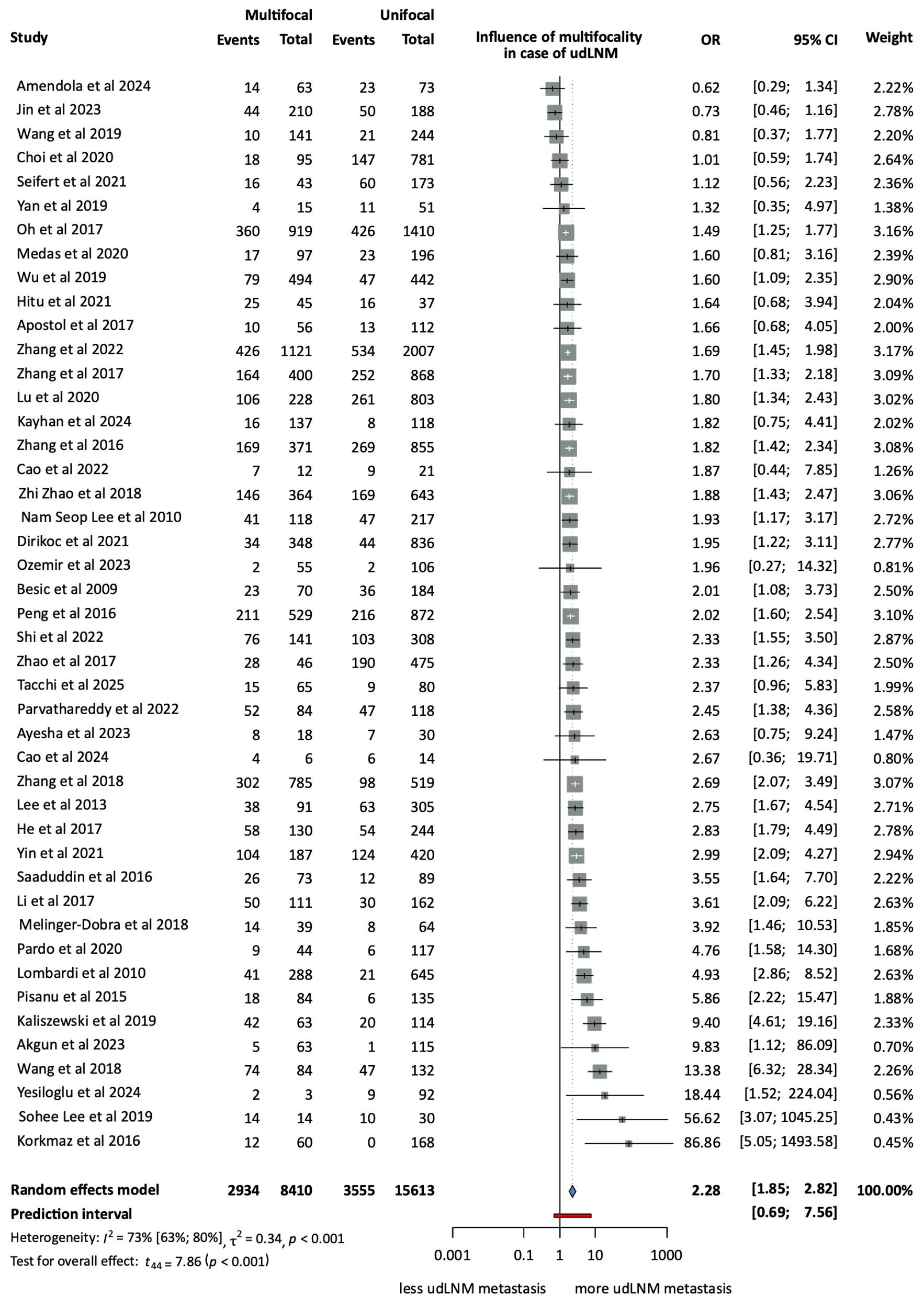


b.)


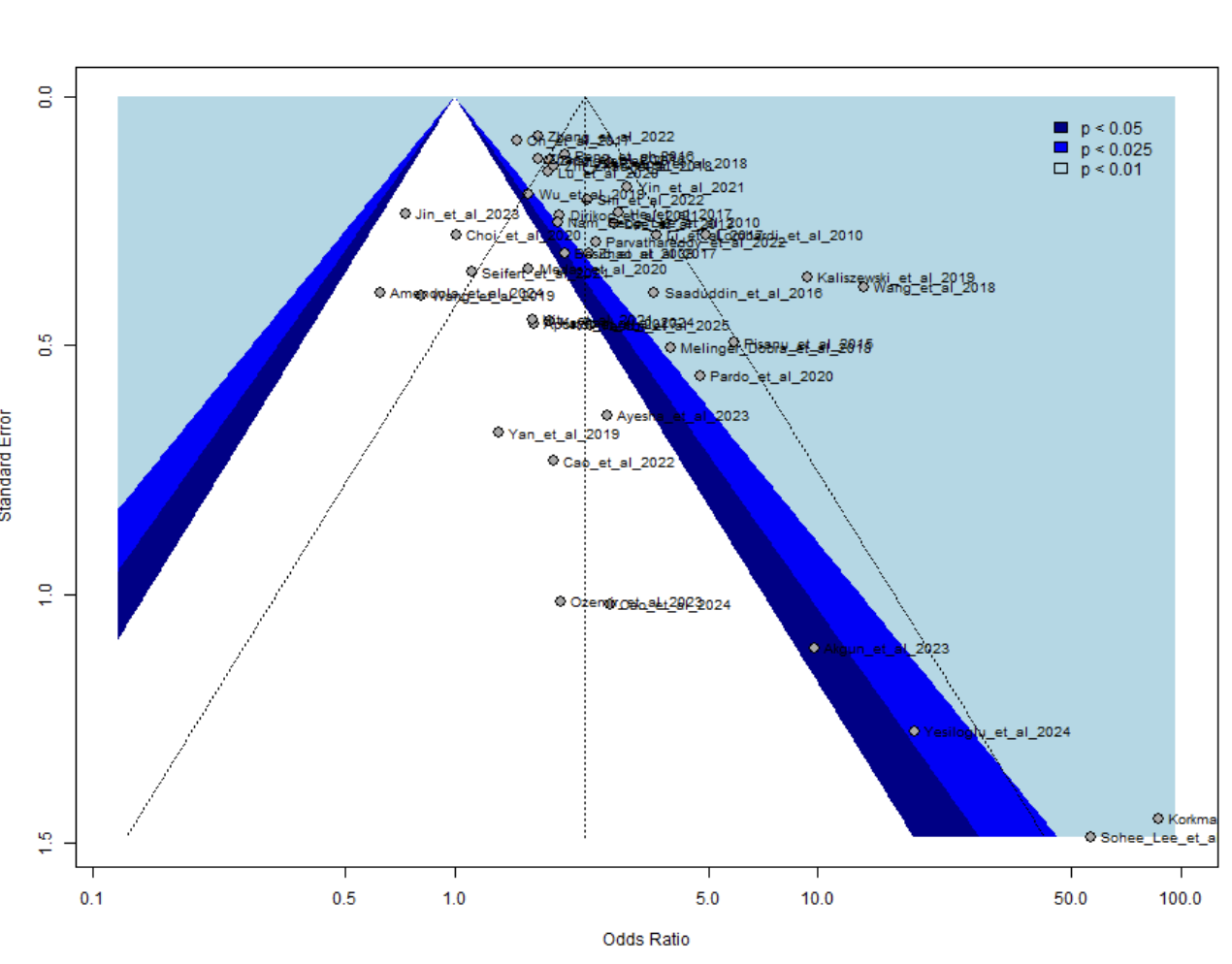


Egger’s test p= 0.0406

**Supplementary Figure 35 a-b** | Forest and funnel plots of bilaterality and its influence in the case of undetermined lymph node metastasis (udLNM)

a.)


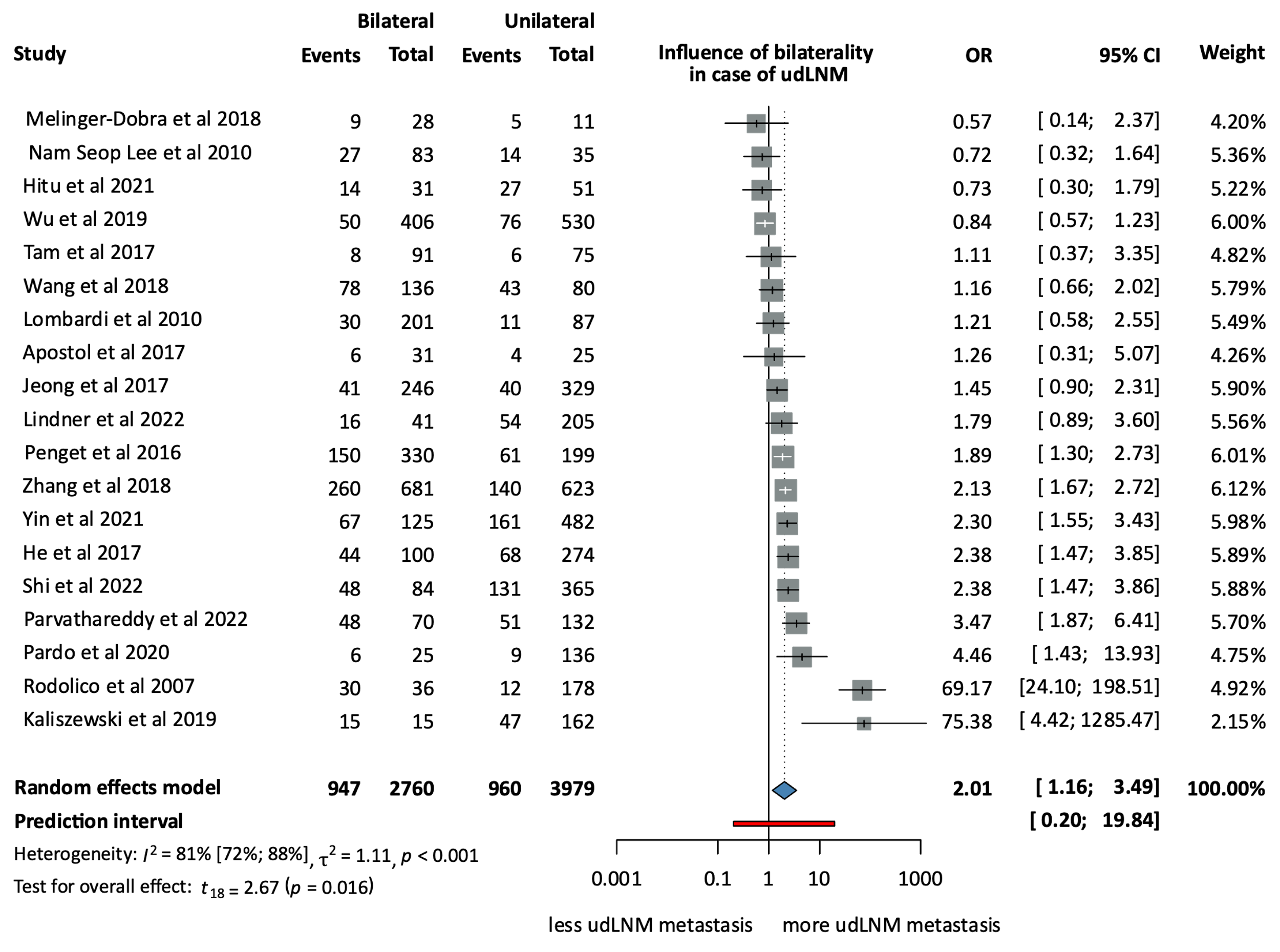


b.)


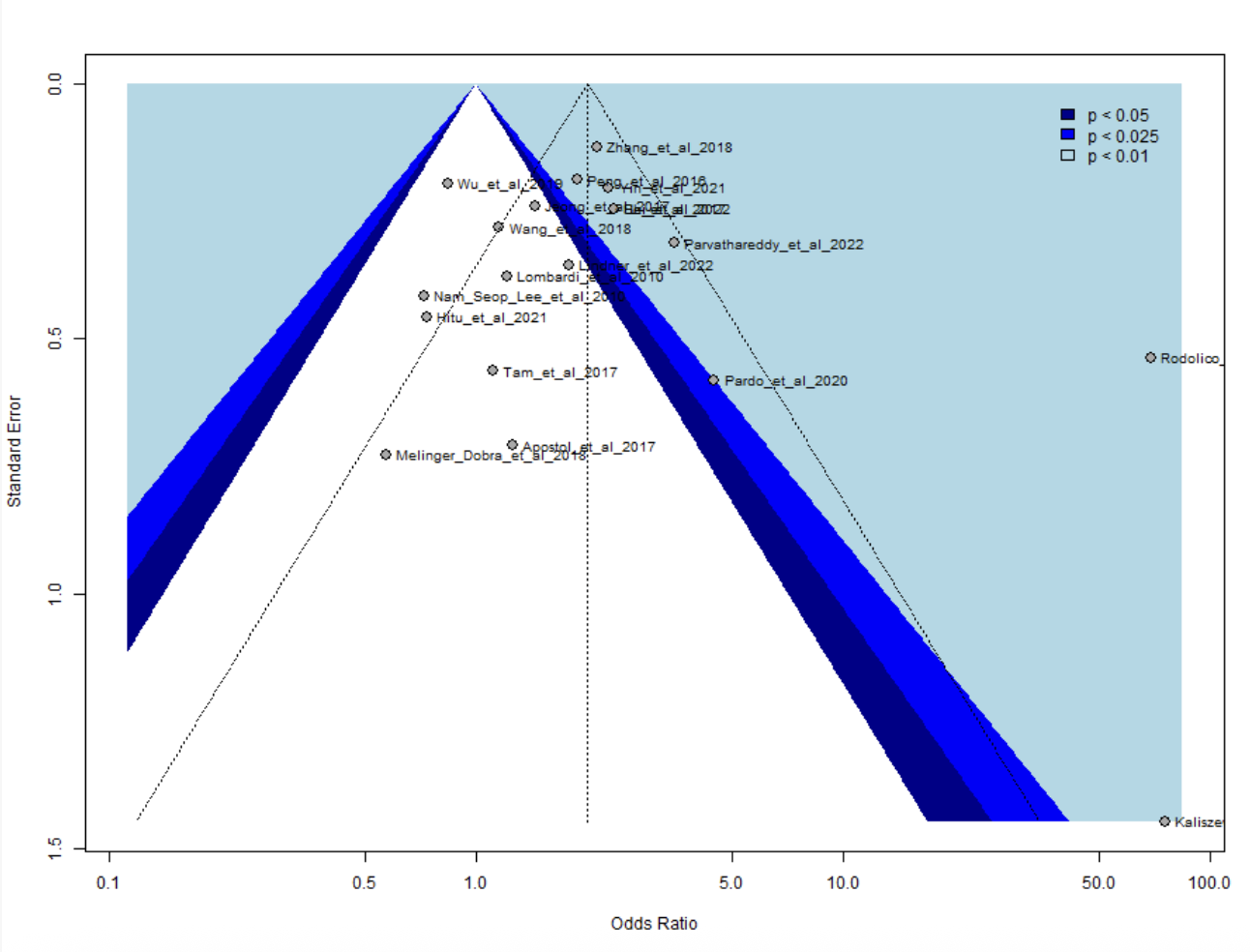


Egger’s test p= 0.9179

**Supplementary Figure 36 a-b** | Forest and funnels plot of BRAF^V600E^ mutation and its influence in the case of undetermined lymph node metastasis (udLNM)

a.)


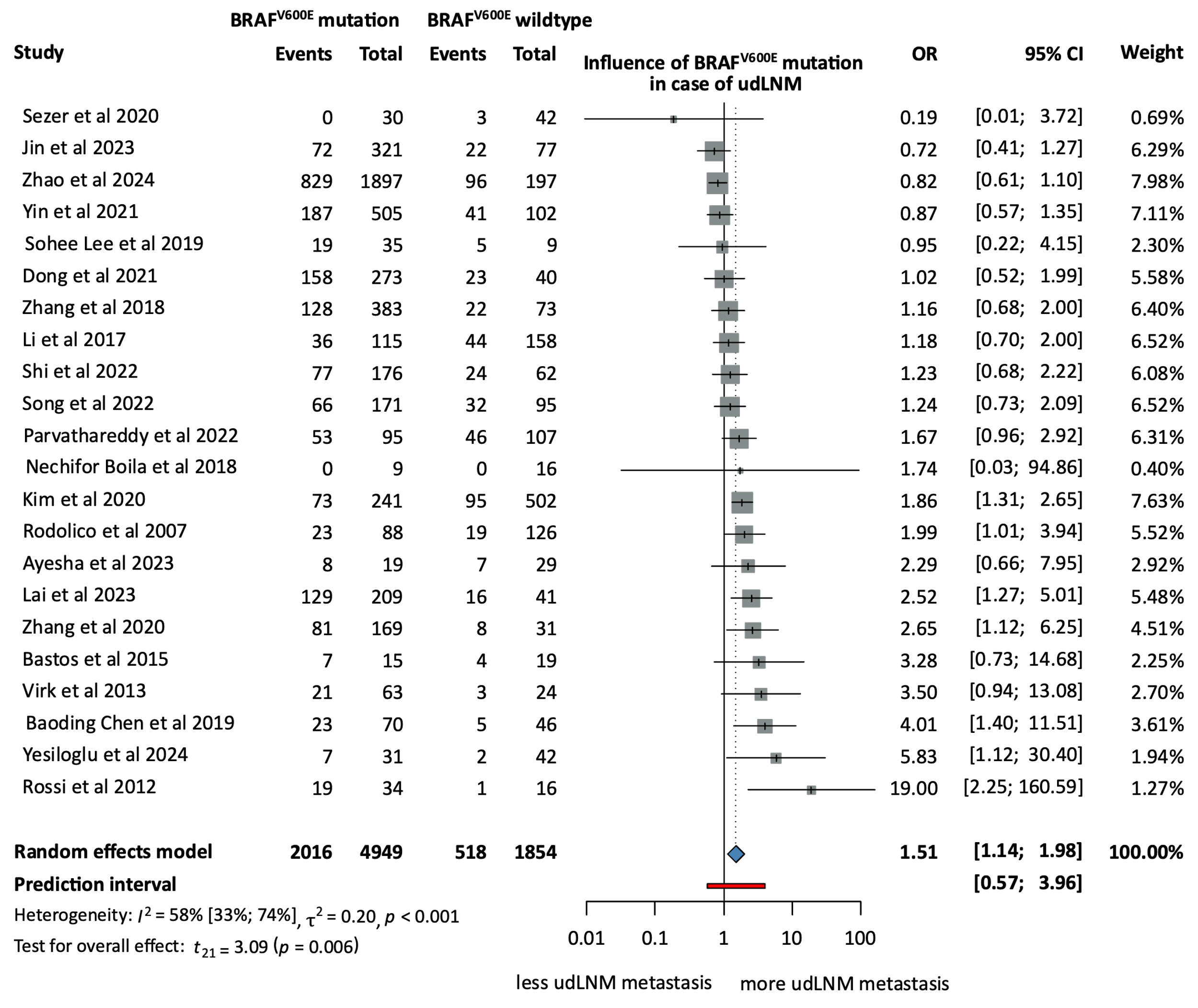


b.)


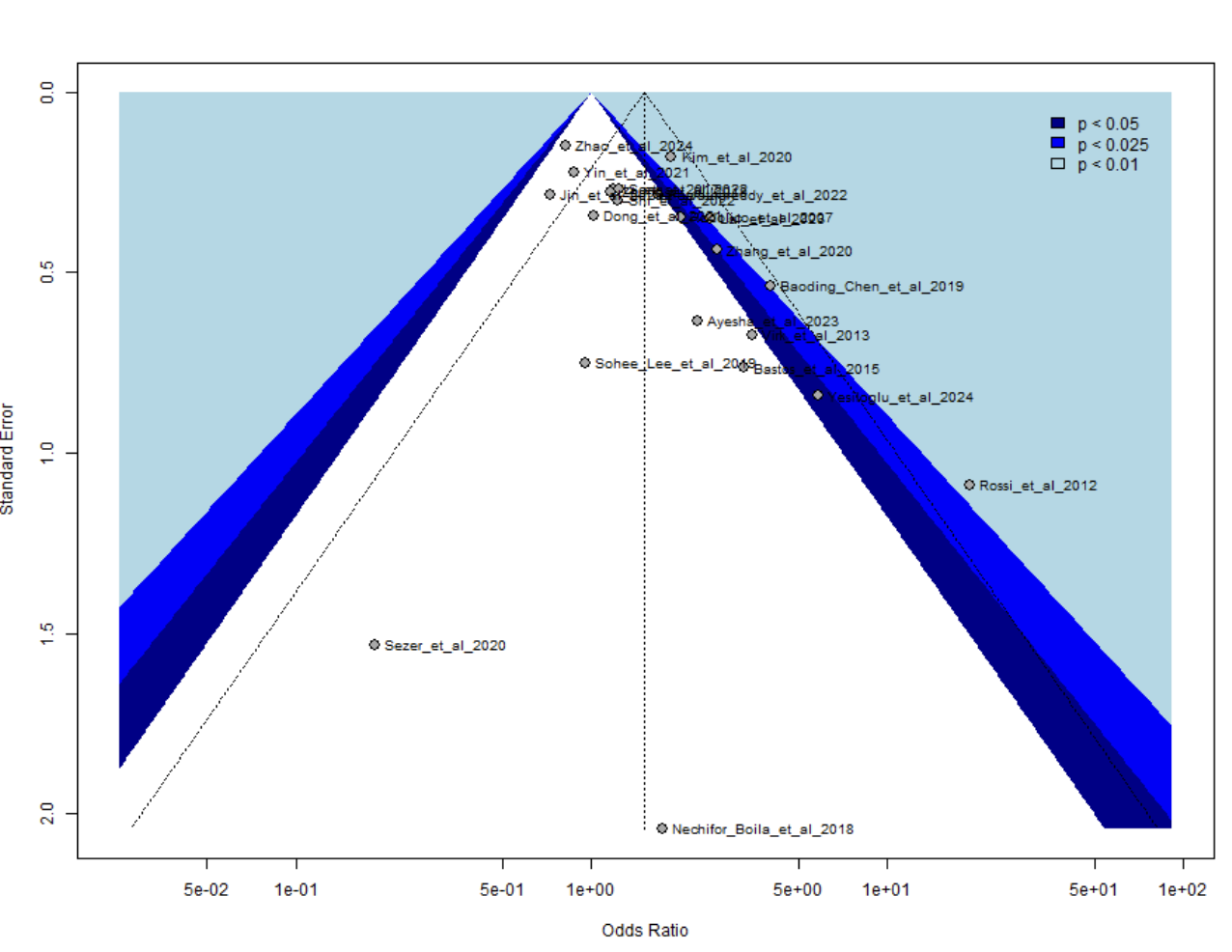


Egger’s test p= 0.0036

**Supplementary Figure 37 a-b** | Forest and funnel plots of Hashimoto’s thyroiditis and its influence in the case of undetermined lymph node metastasis (udLNM)

a.)


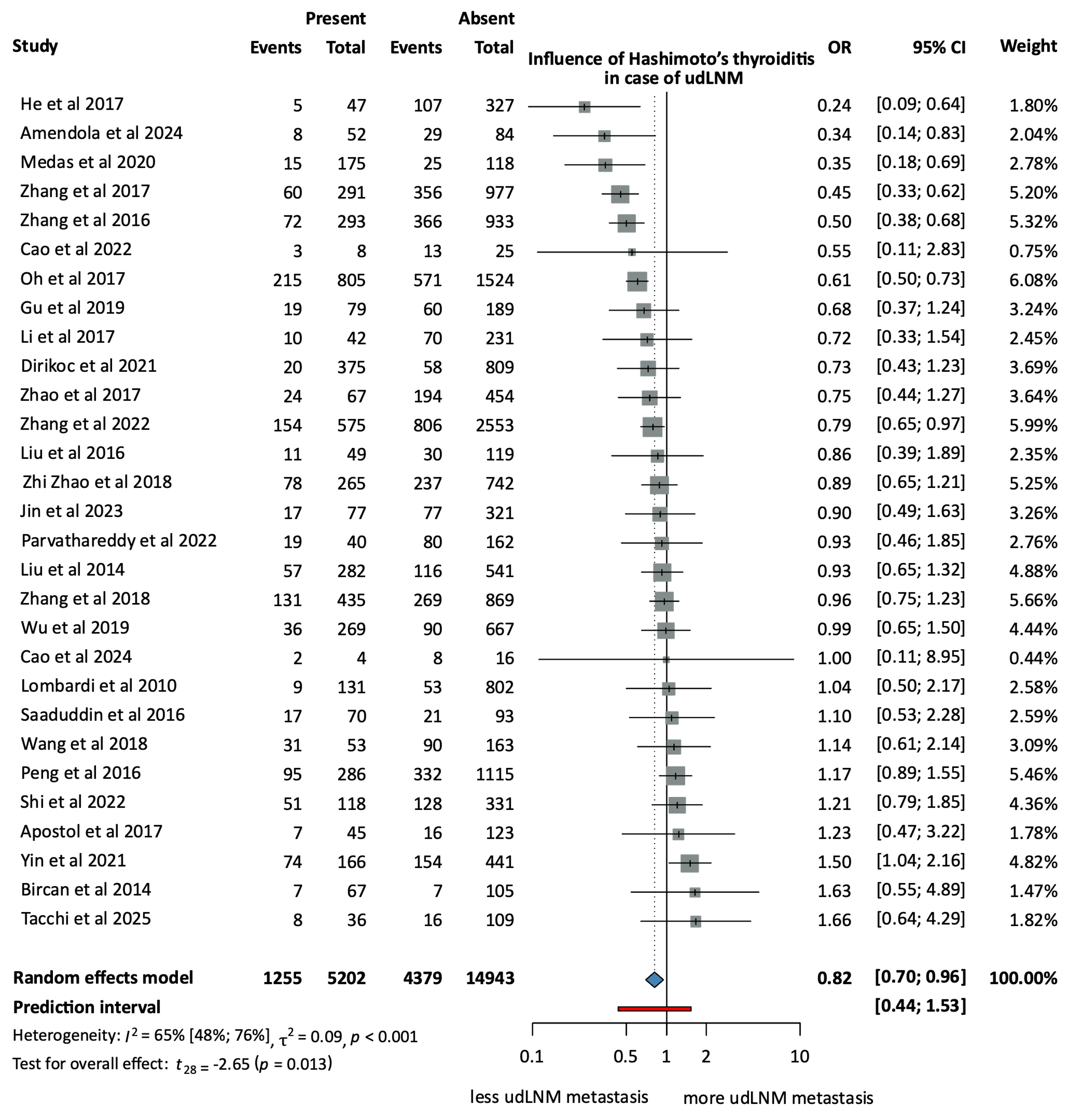


b.)


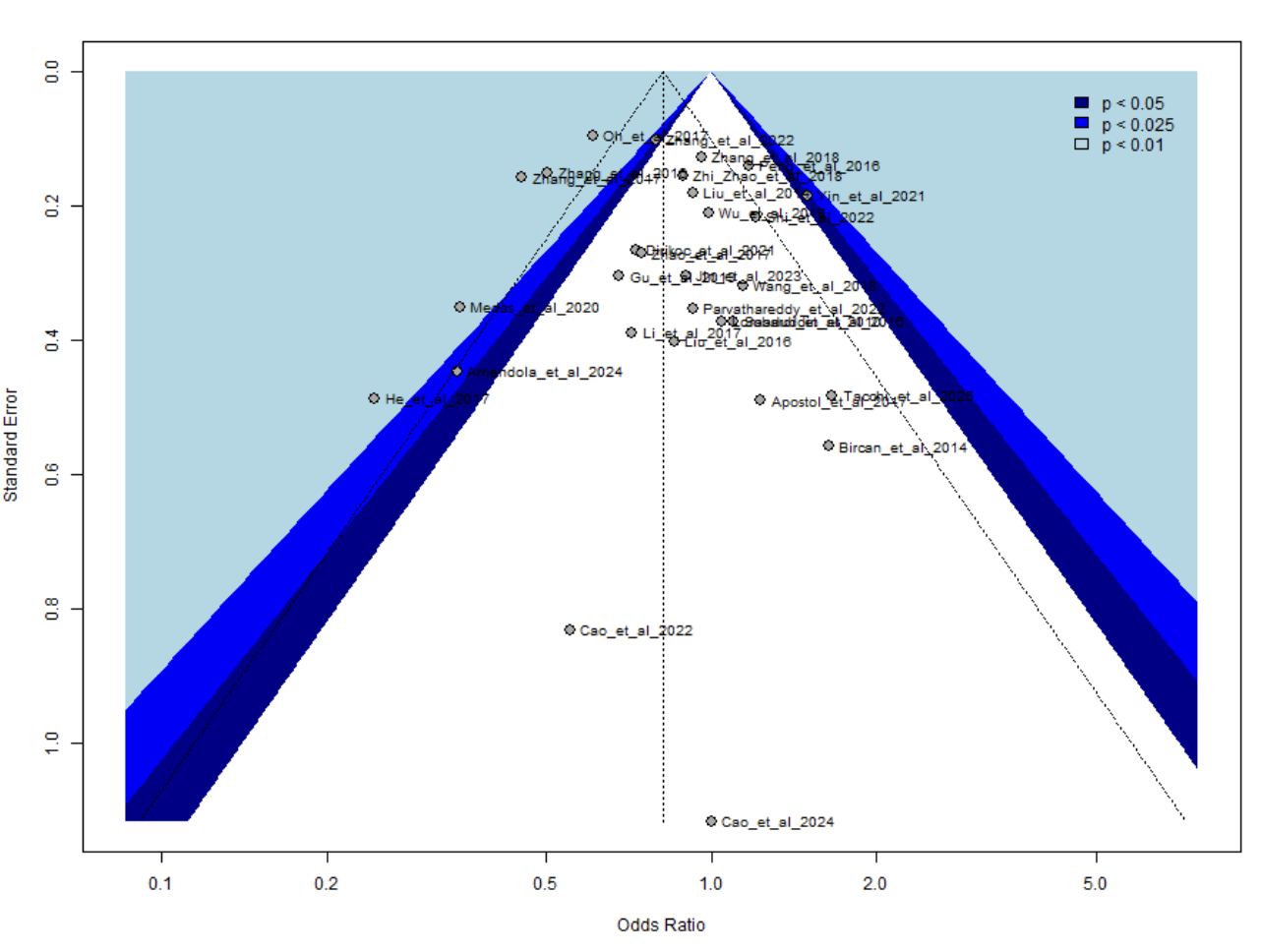


Egger’s test p= 0.8064

**Supplementary Figure 38 a-b** | Forest and funnel plots of capsule invasion and its influence in the case of undetermined lymph node metastasis (udLNM)

a.)


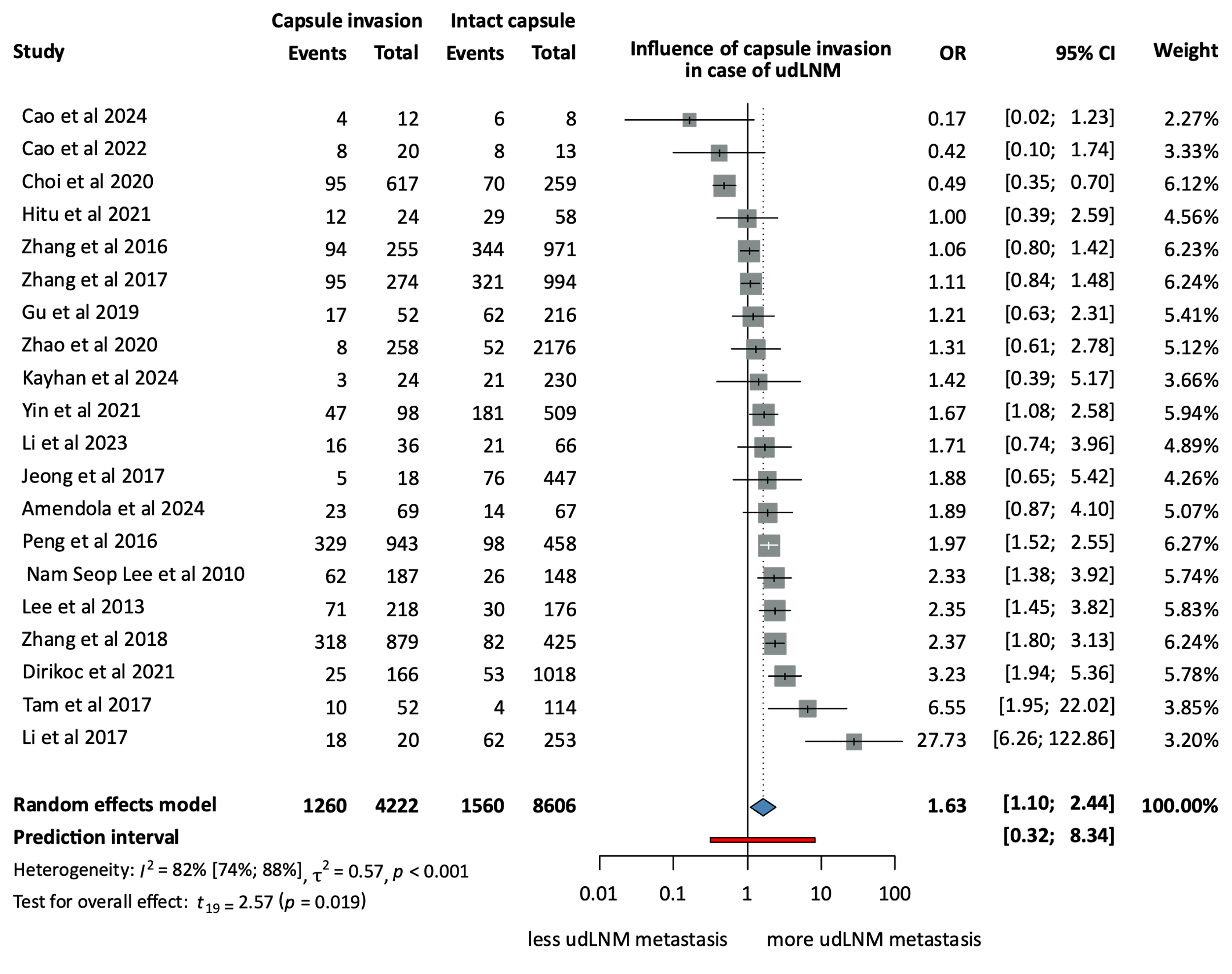


b.)


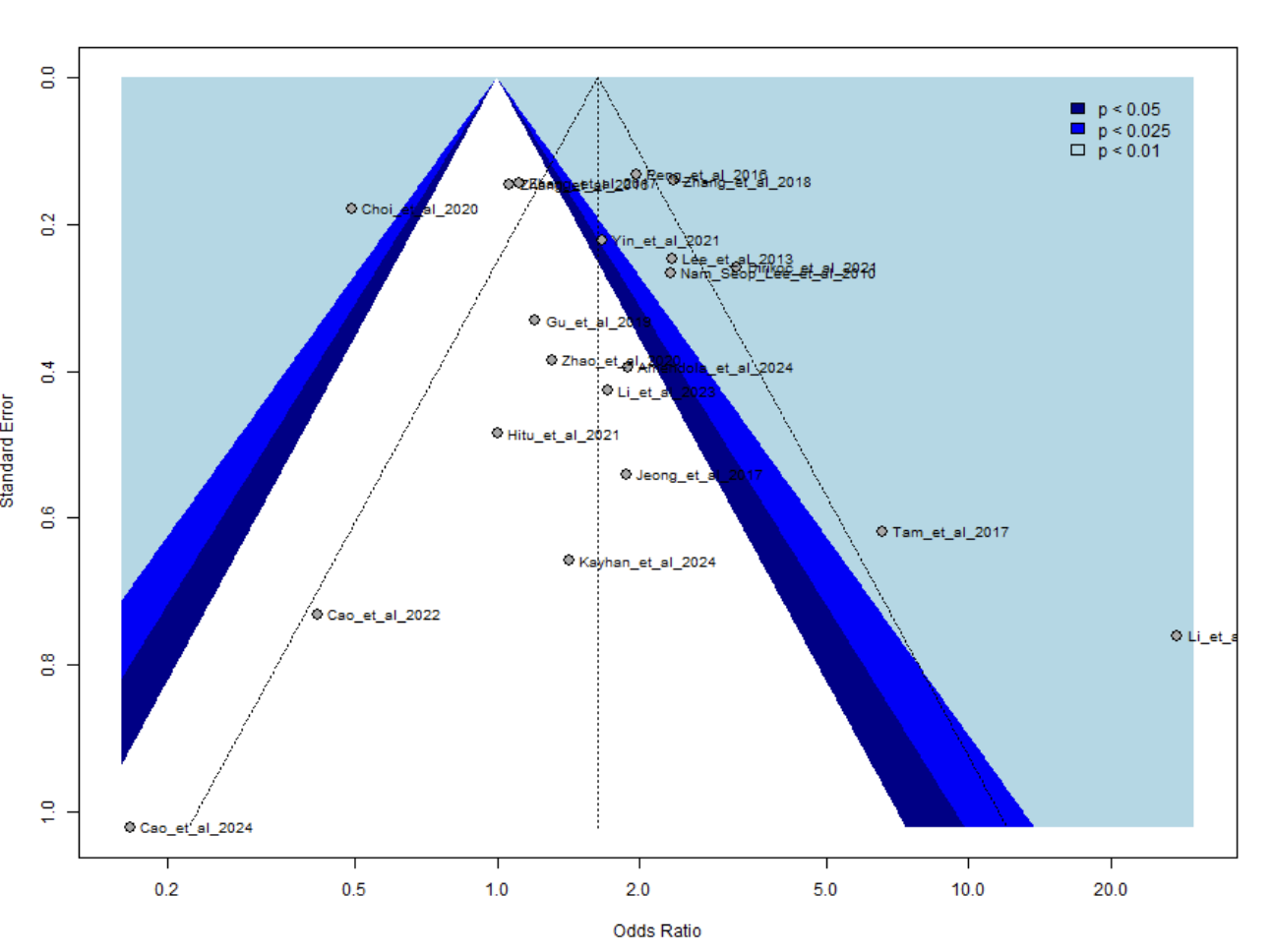


Egger’s test p= 0.7130

**Supplementary Figure 39** | Forest plot of Graves’ disease and its influence in the case of undetermined lymph node metastasis (udLNM)


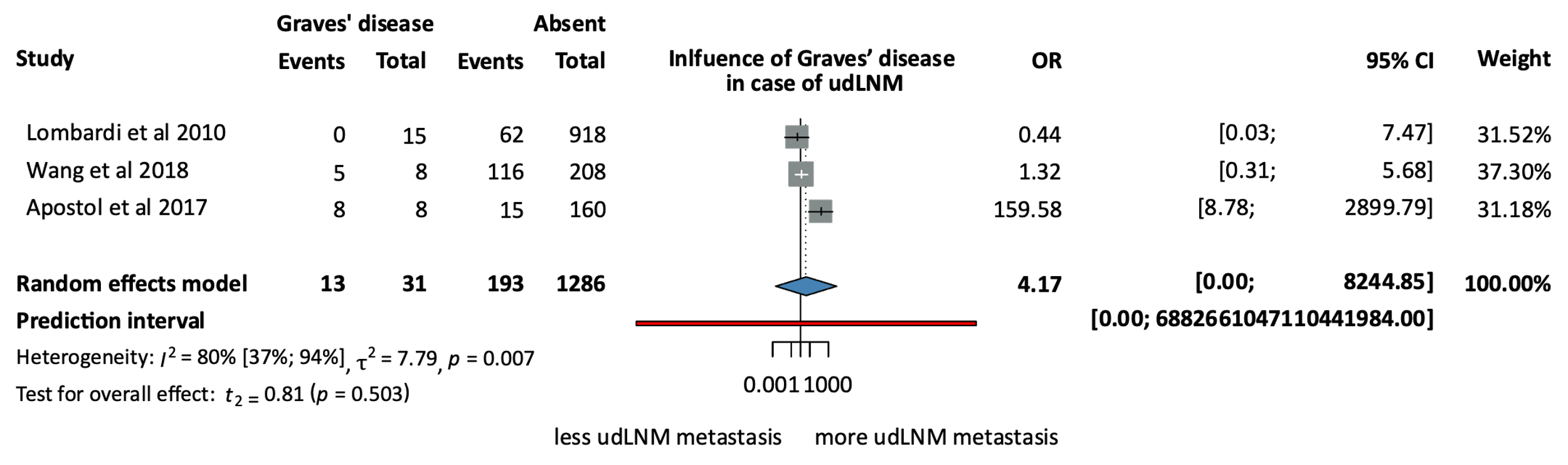


**Supplementary Figure 40** | Forest plot of thyroglobulin antibody (anti-Tg) and its influence in case of undetermined lymph node metastasis (udLNM)


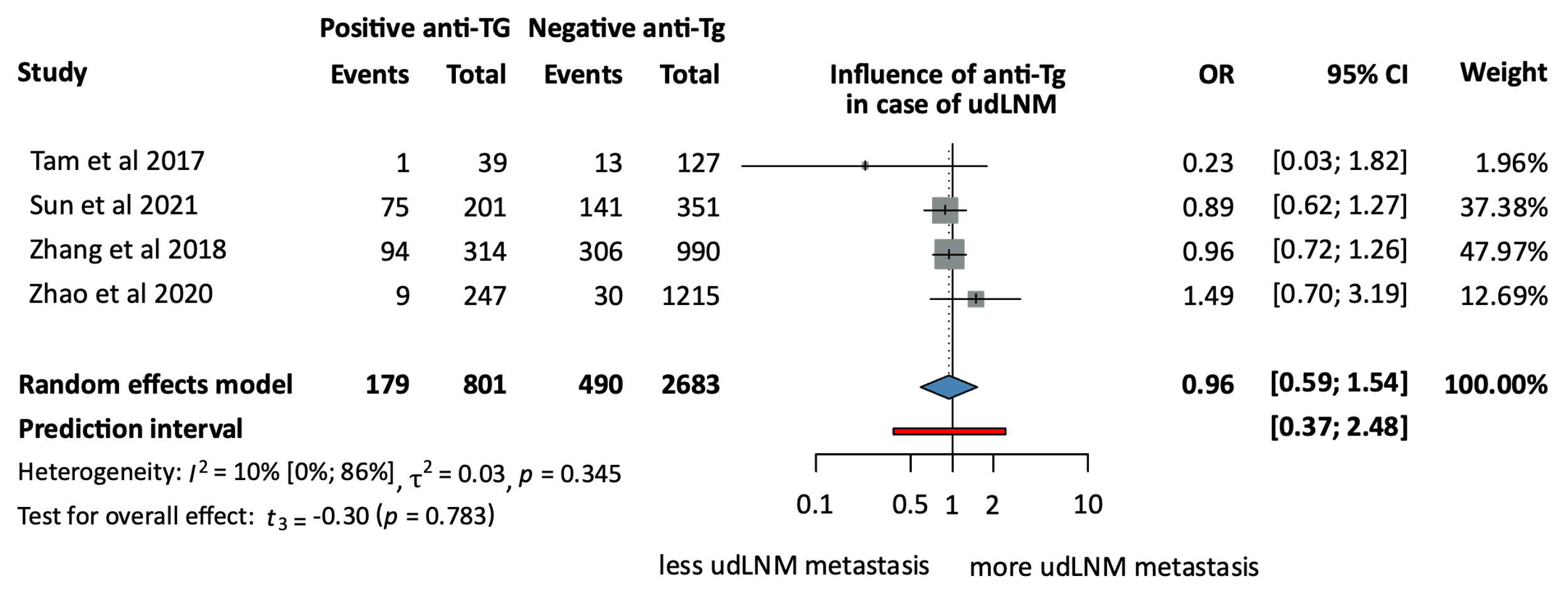


**Supplementary Figure 41** | Forest plot of microcalcification and its influence in the case of undetermined lymph node metastasis (udLNM)


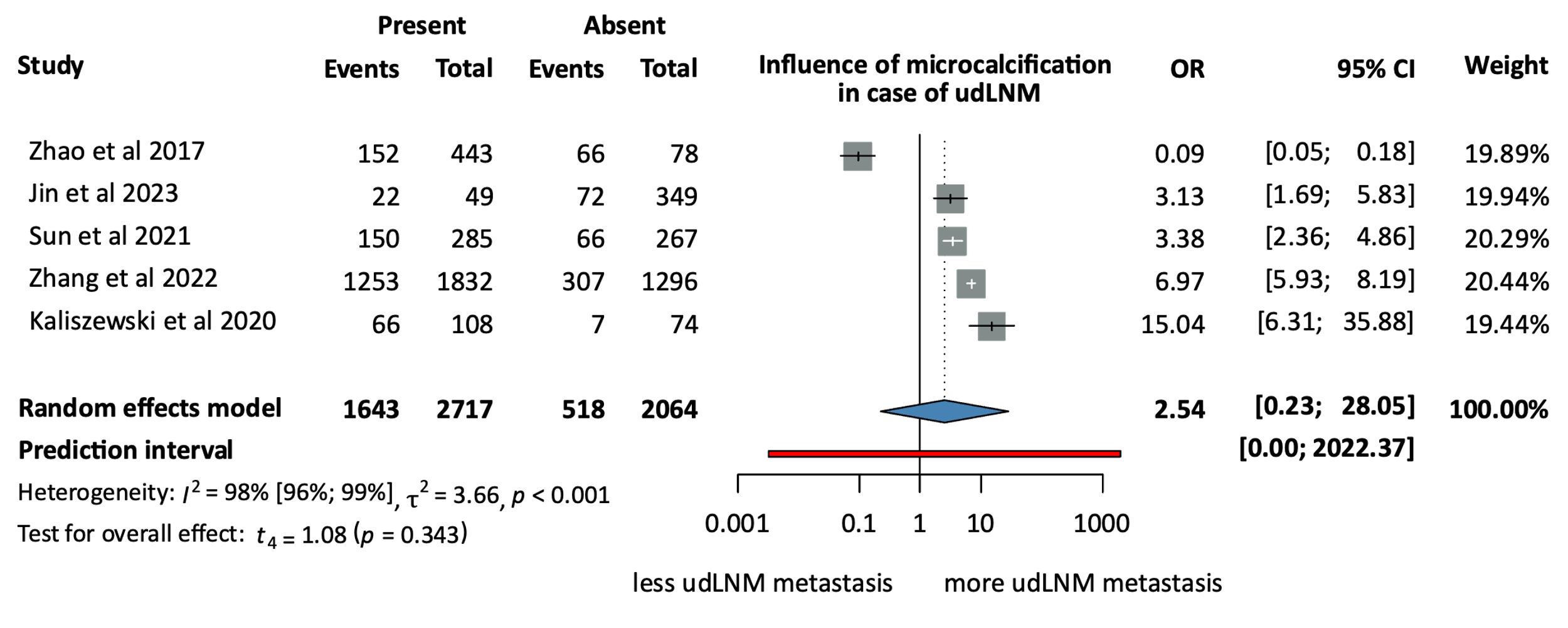


**Supplementary Figure 42** | Forest plot of goiter and its influence in the case of undetermined lymph node metastasis (udLNM)


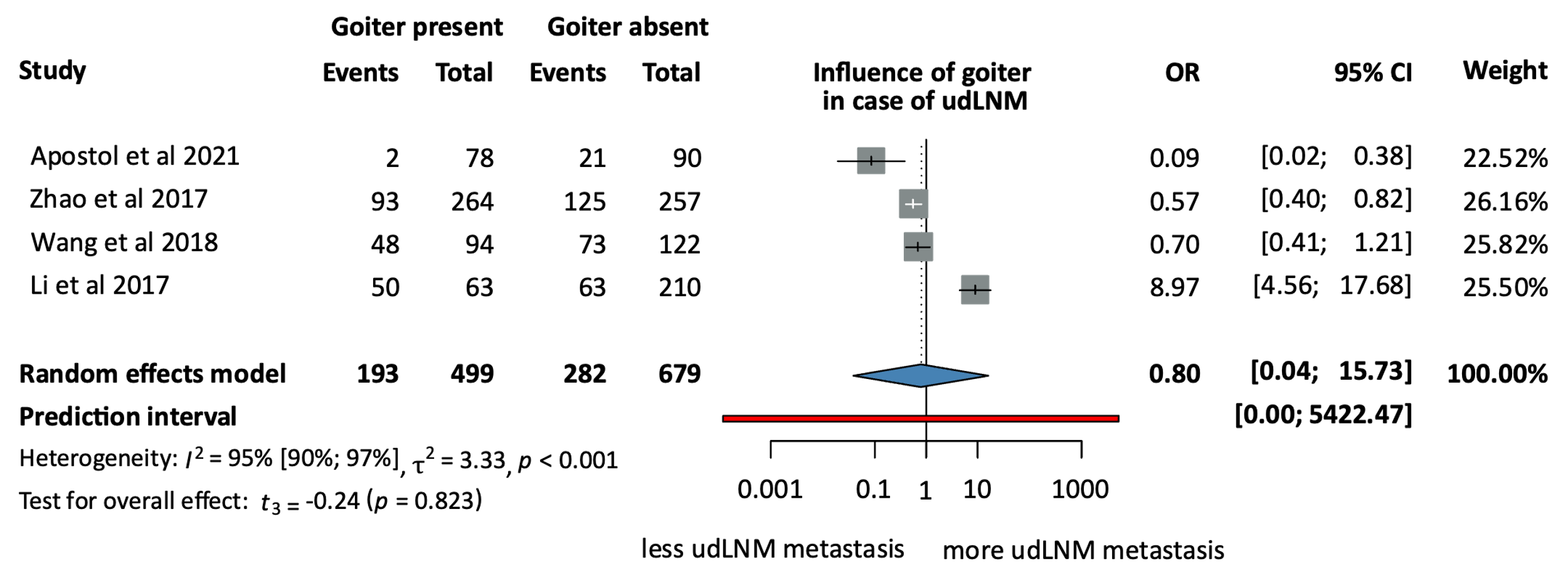


**Supplementary Figure 43** | Forest plot of TERT mutation and its influence in the case of undetermined lymph node metastasis (udLNM)


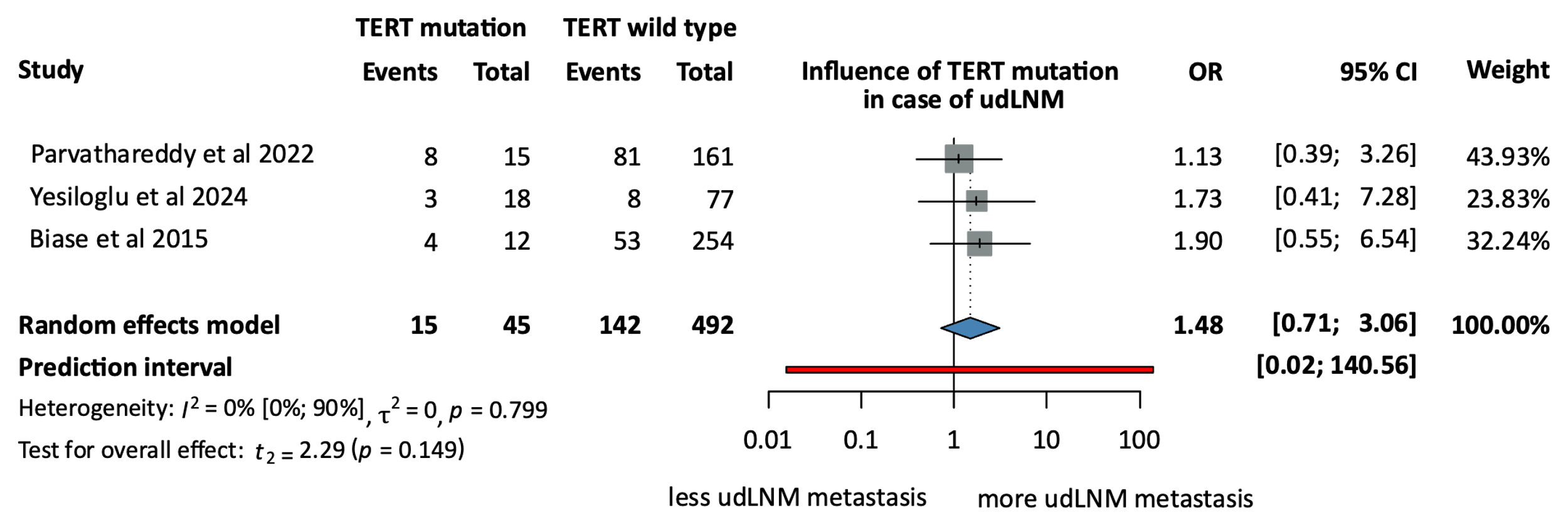


**Supplementary Figure 44** | Forest plot of increased TSH level and its influence in the case of undetermined lymph node metastasis (udLNM)


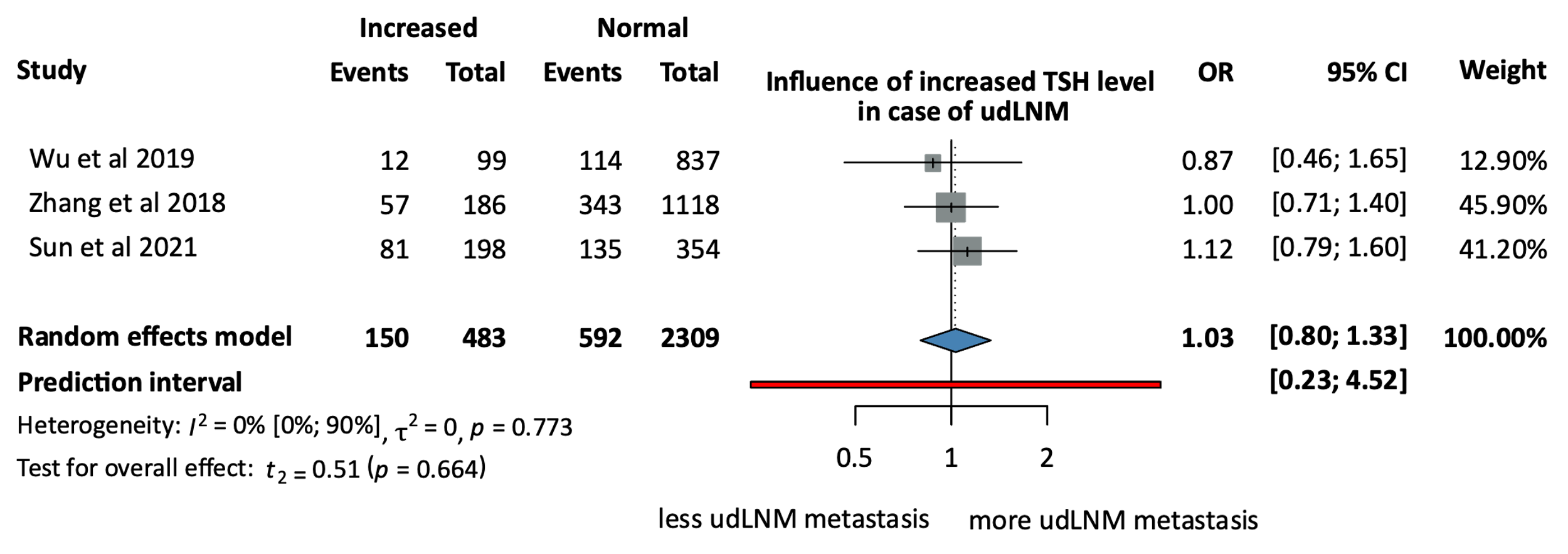


**Supplementary Figure 45 a-b** | Forest and funnel plots of male sex and its influence in the case of extrathyroidal extension (ETE)

a.)


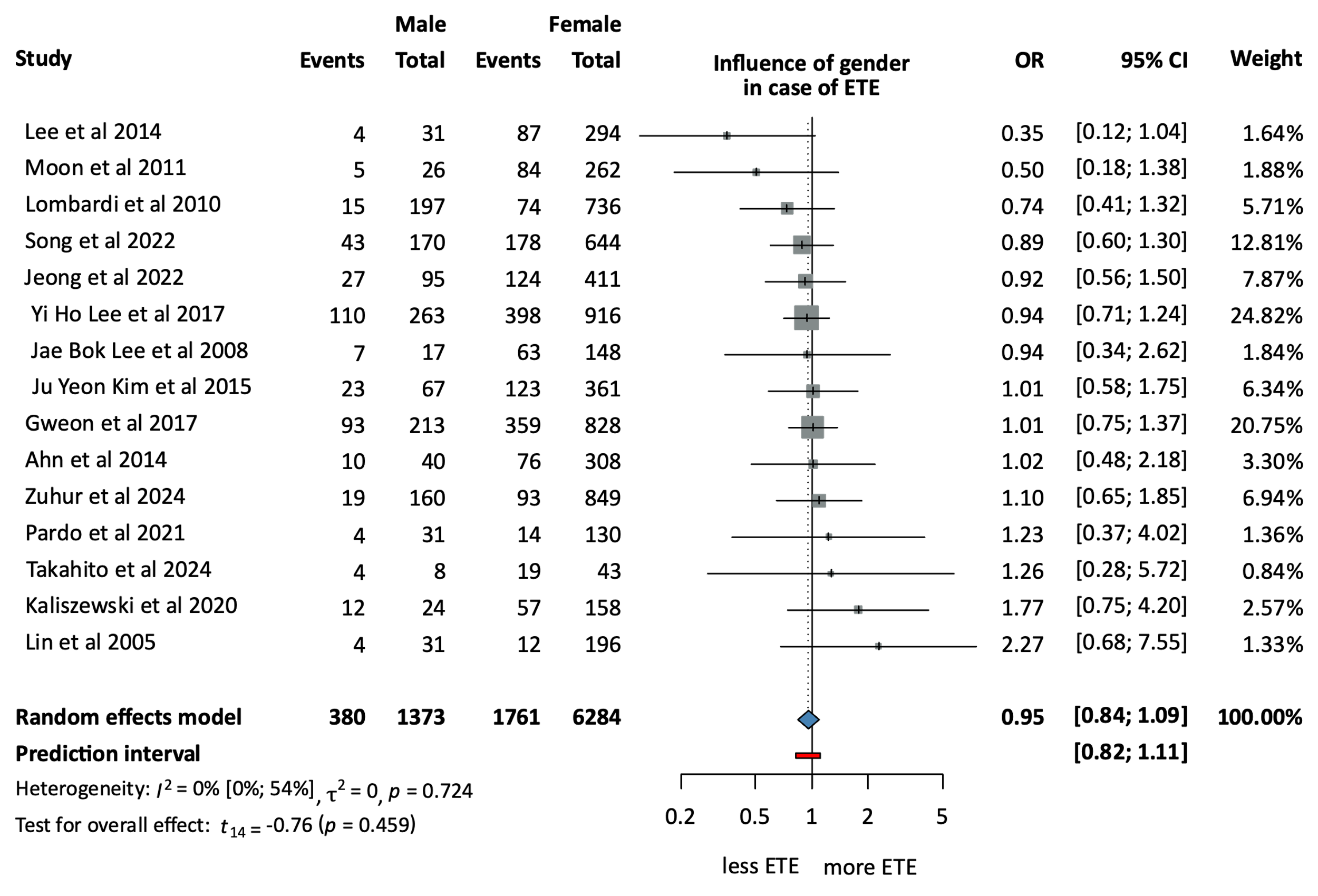


b.)


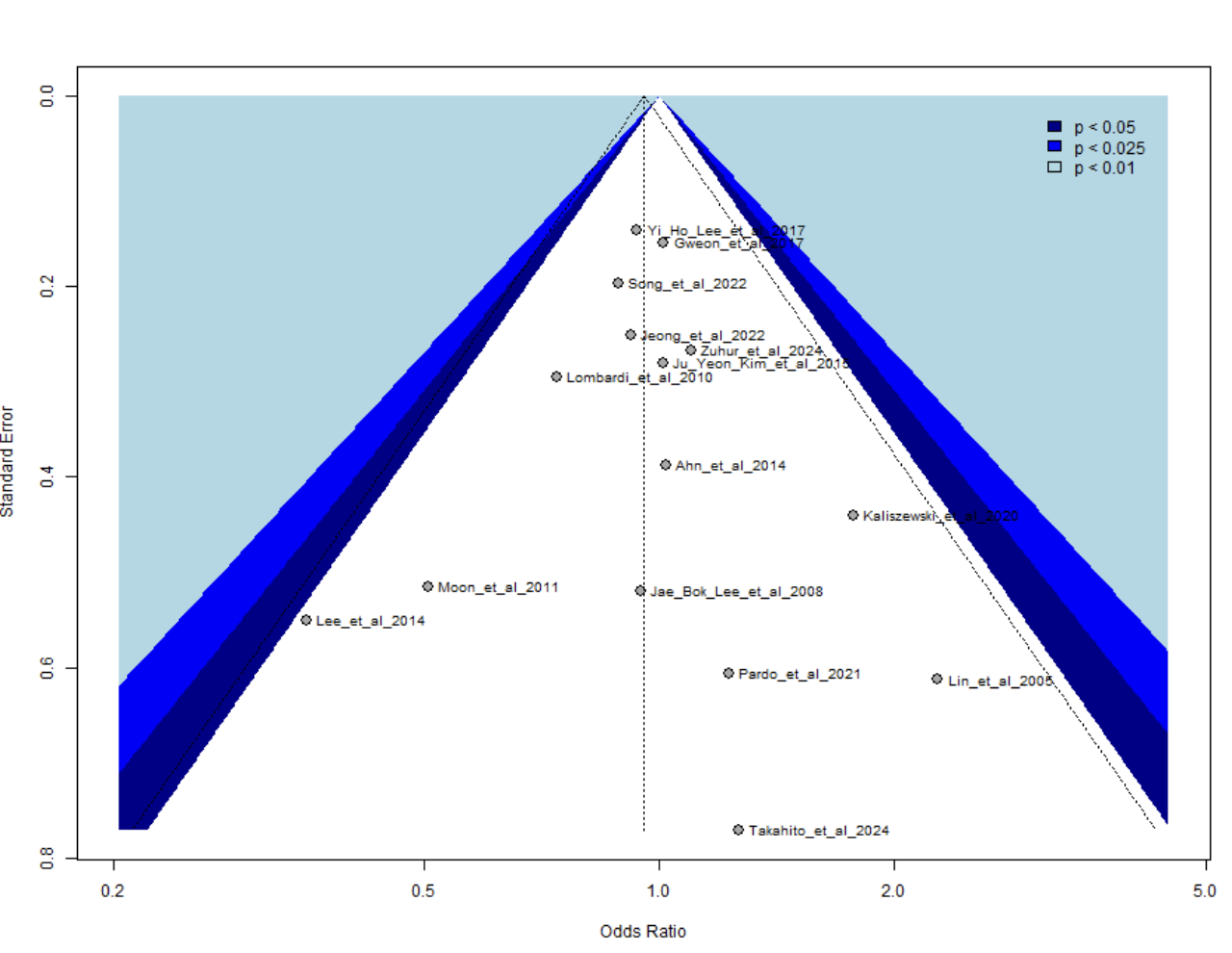


Egger’s test p= 0.7467

**Supplementary Figure 46** | Forest plot of age under 45 and its influence in the case of extrathyroidal extension (ETE)

a.)


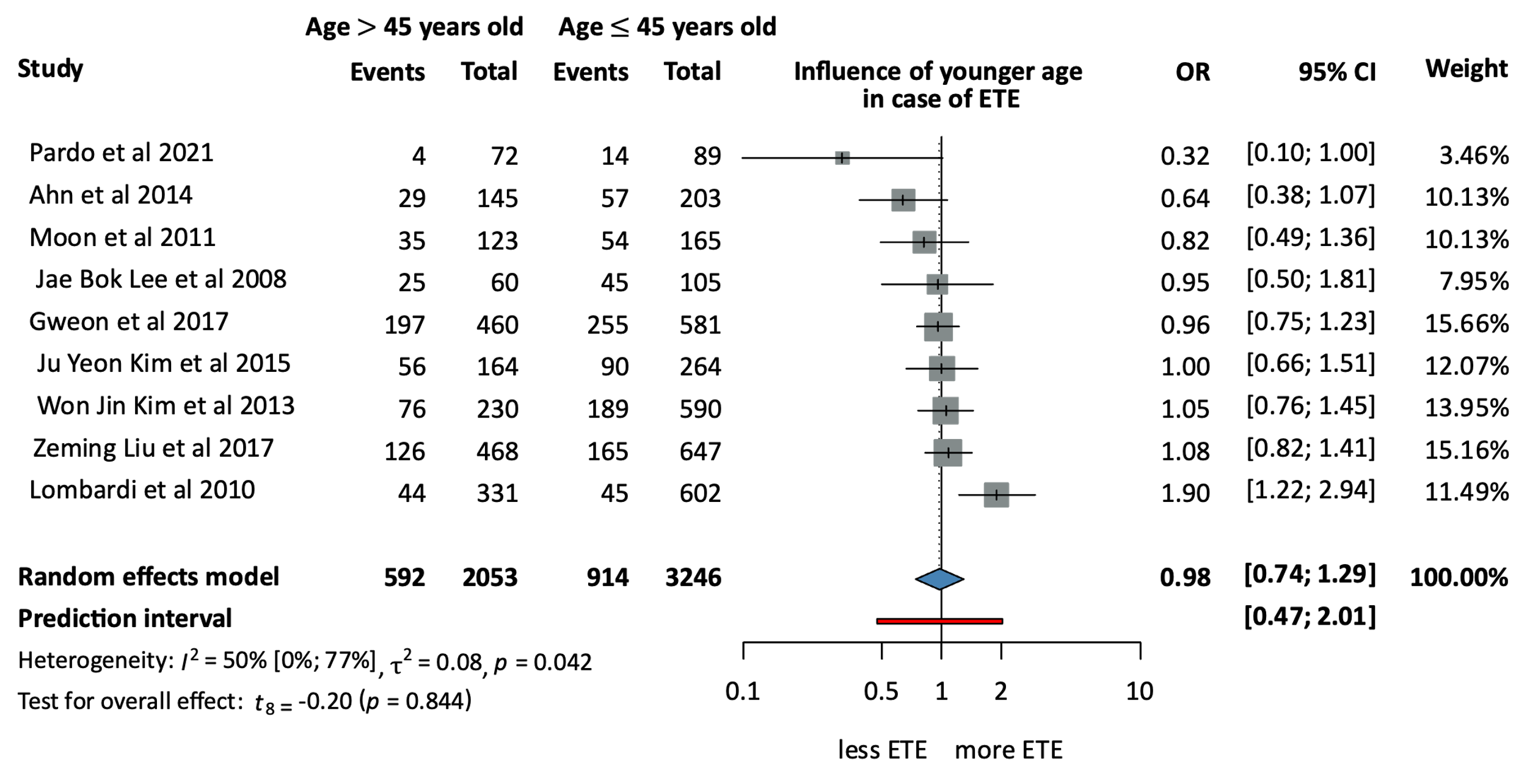


**Supplementary Figure 47** | Forest plot of age under 55 and its influence in the case of extrathyroidal extension (ETE)


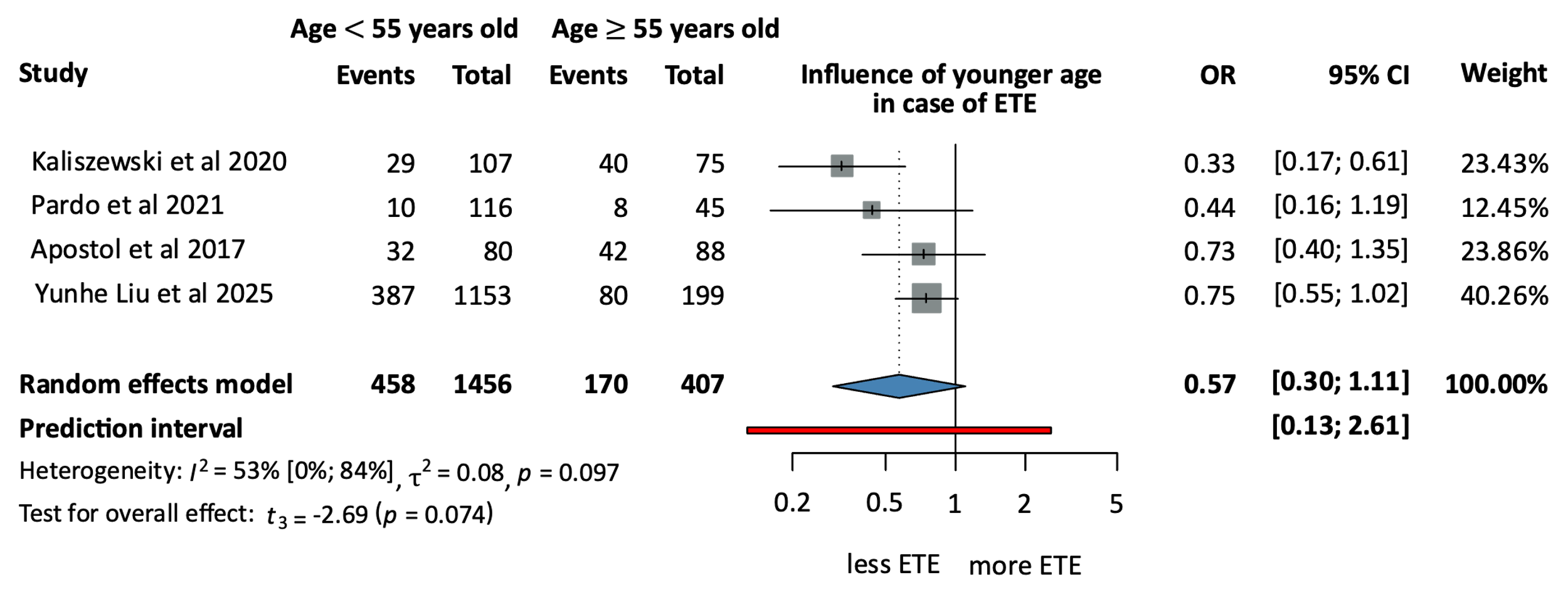


**Supplementary Figure 48 a-b** | Forest and funnel plots of tumor size above 5 mm and its influence in the case of extrathyroidal extension (ETE)

a.)


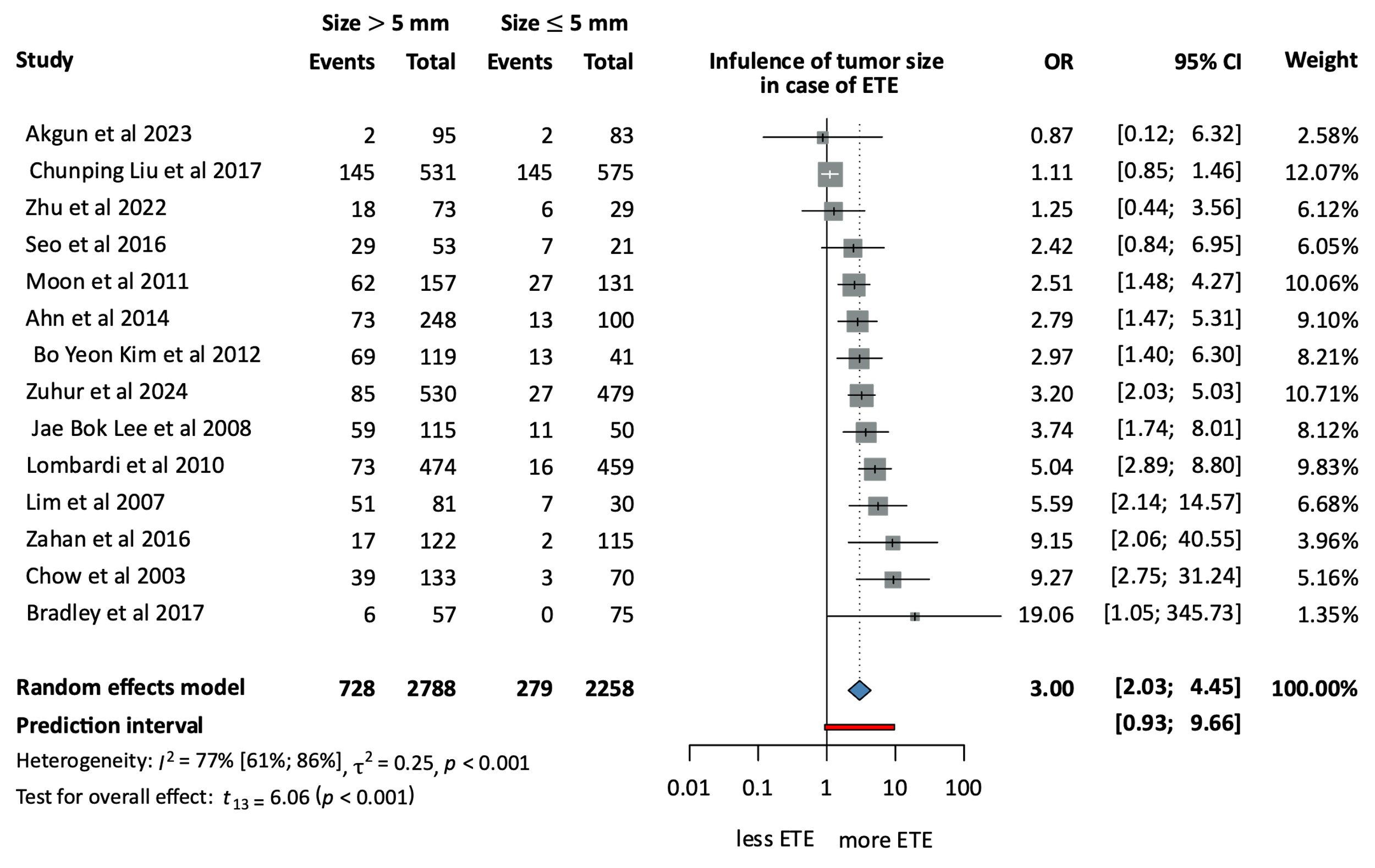


b.)


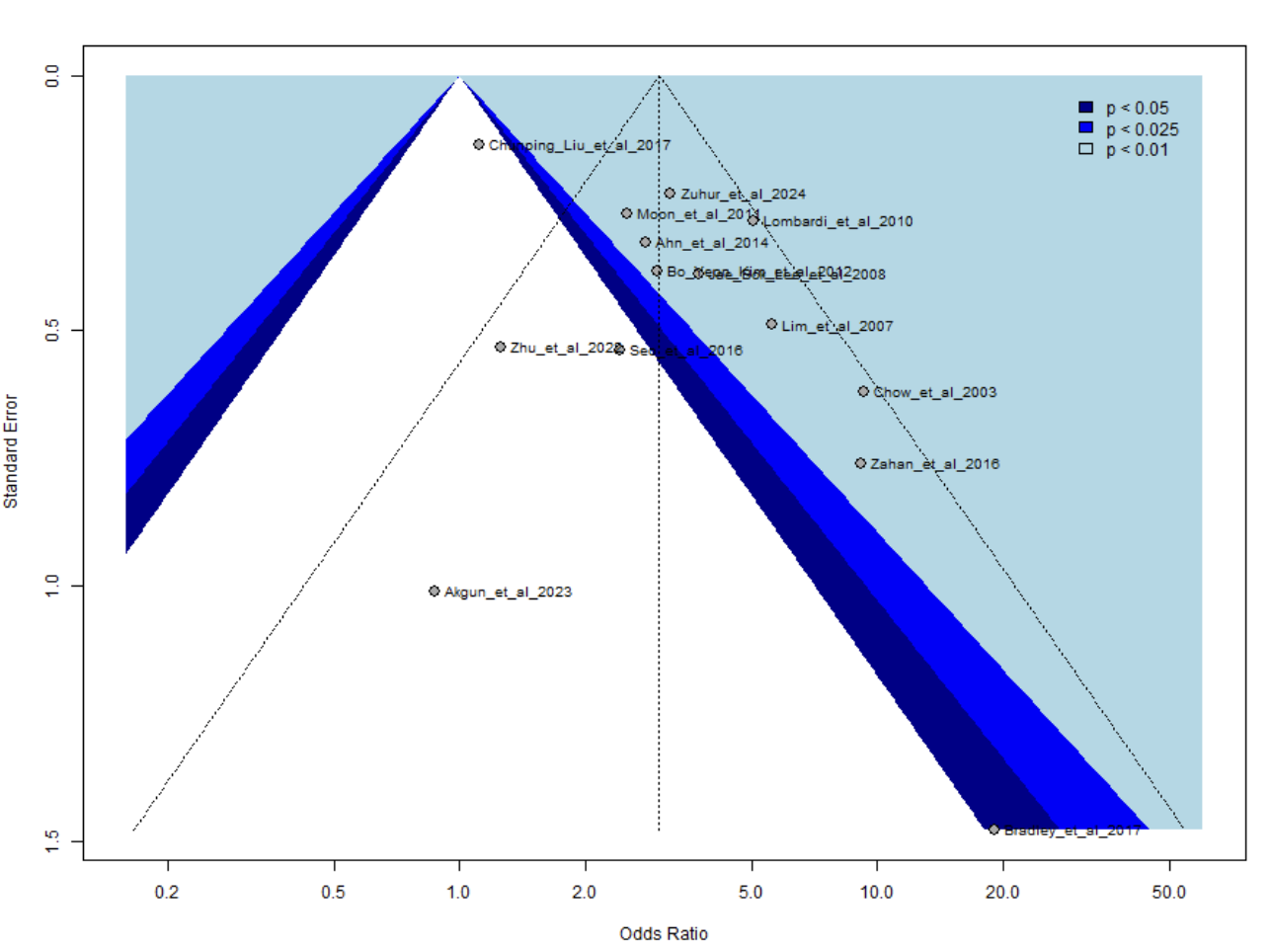


Egger’s test p= 0.2734

**Supplementary Figure 49 a-b** | Forest and funnel plots of multifocality and its influence in the case of extrathyroidal extension (ETE)

a.)


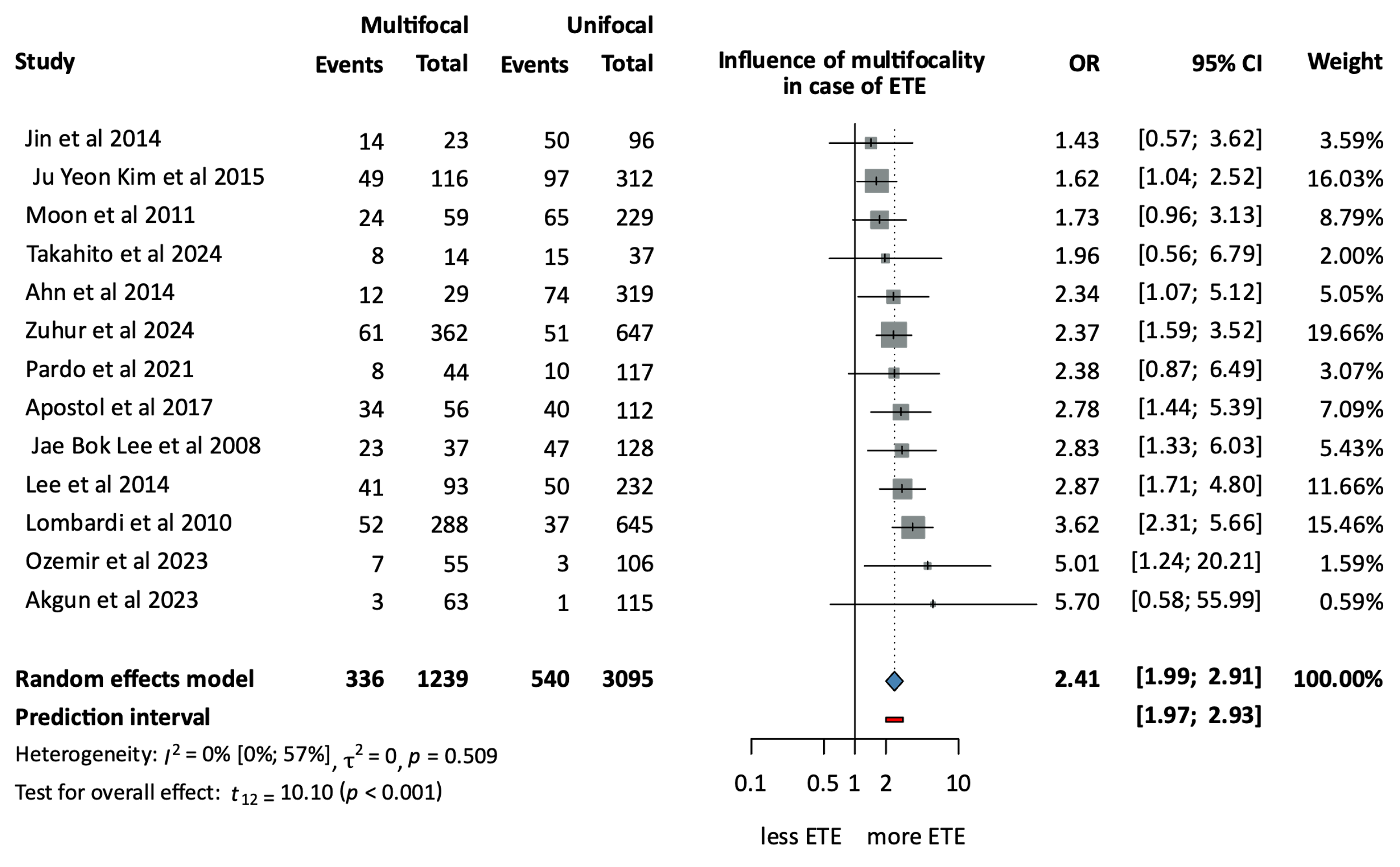


b.)


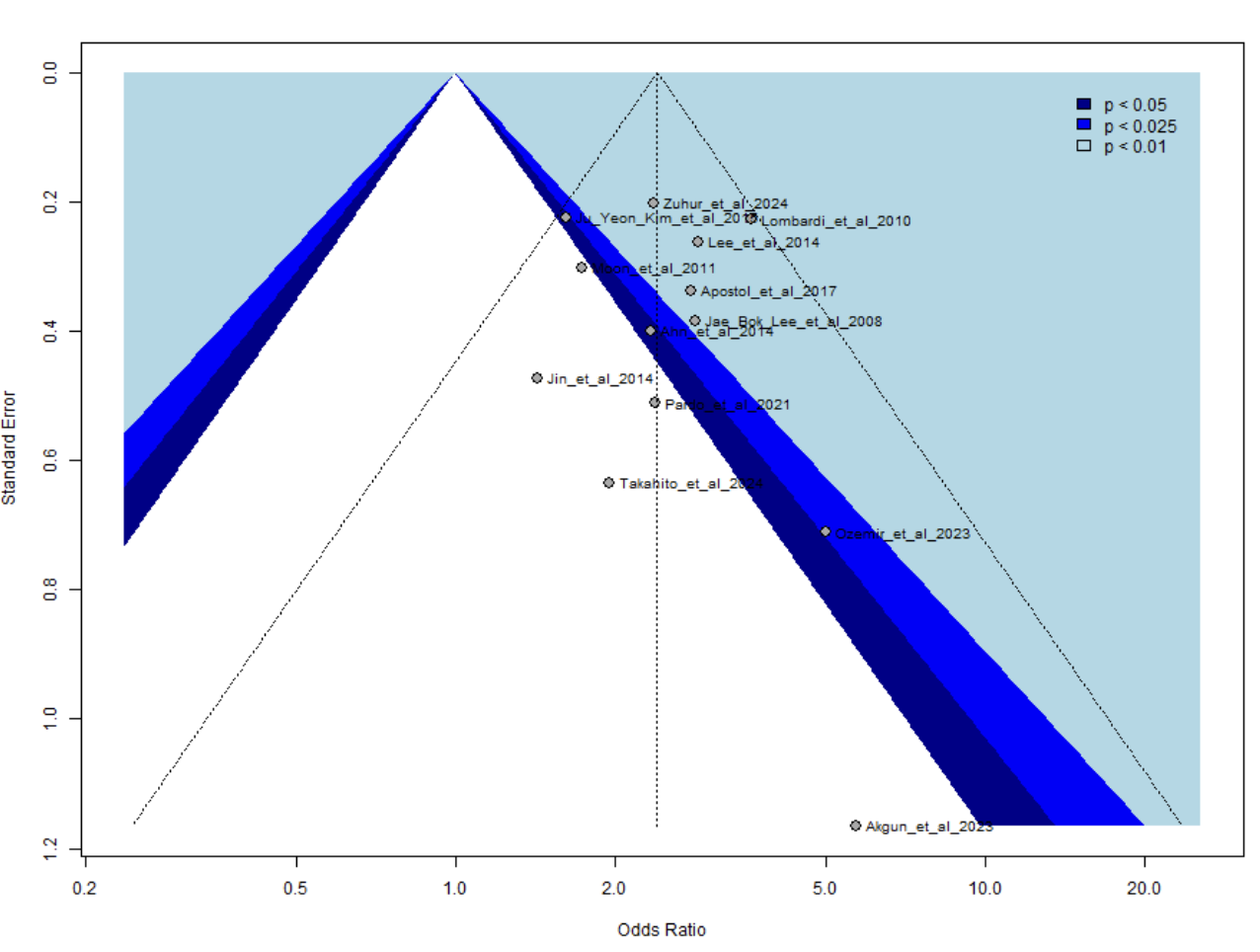


Egger’s test p= 0.5877

**Supplementary Figure 50** | Forest plot of bilaterality and its influence in the case of extrathyroidal extension (ETE)

a.)


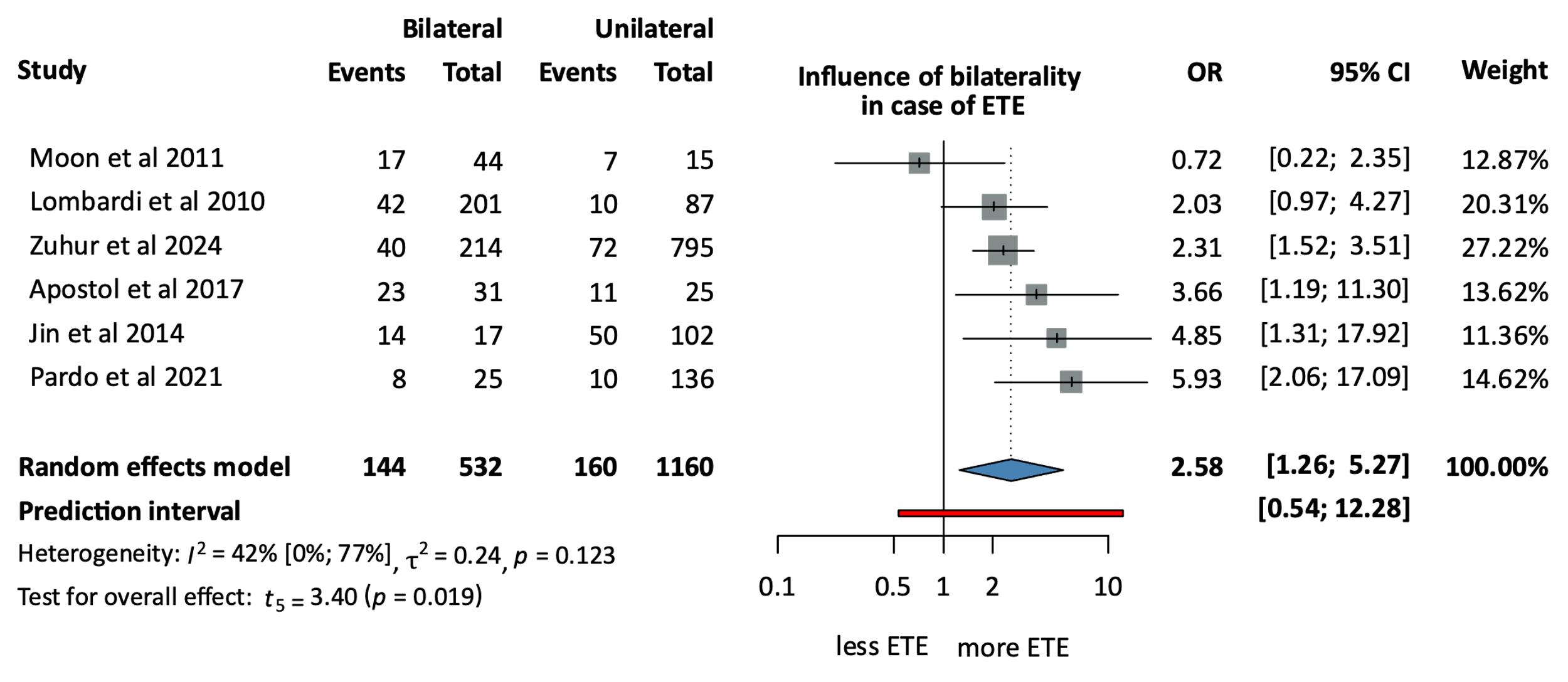


**Supplementary Figure 51 a-b** | Forest and funnel plots of BRAF^V600E^ mutation and its influence in the case of extrathyroidal extension (ETE)

a.)


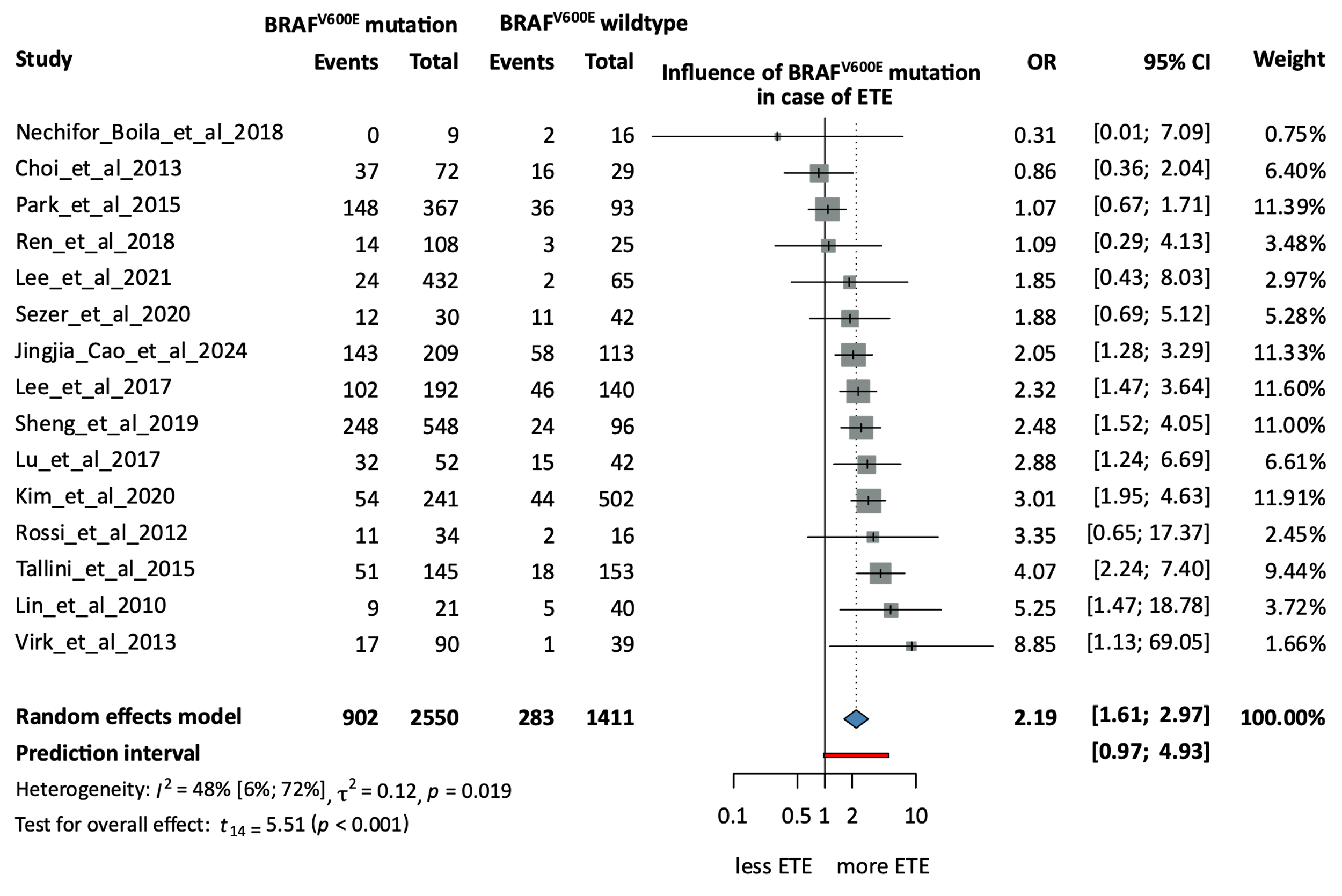


b.)


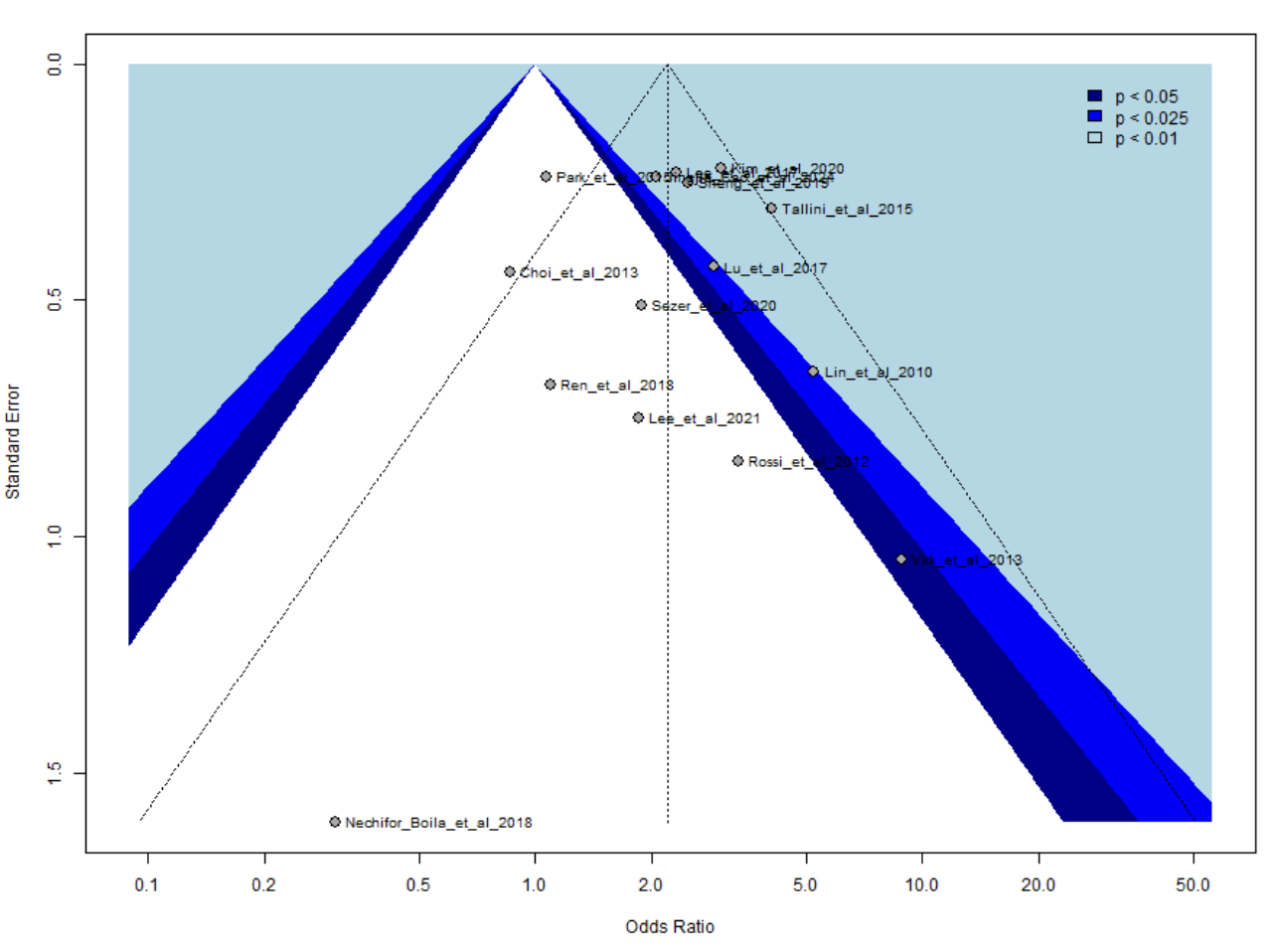


Egger’s test p= 0.9764

**Supplementary Figure 52** | Forest plot of Hashimoto’s thyroiditis and its influence in the case of extrathyroidal extension (ETE)


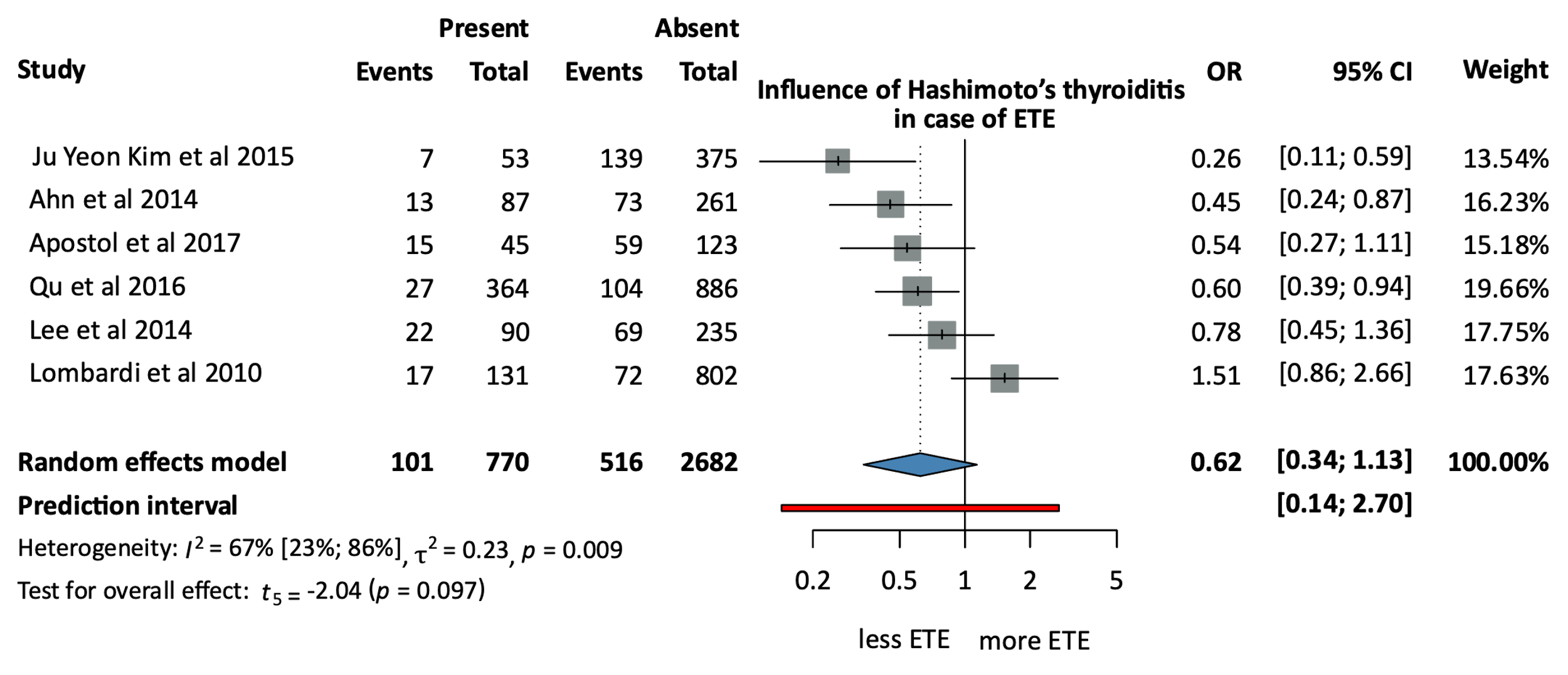


**Supplementary Figure 53** | Forest plot of male gender and its influence in case of central lymph node metastasis (CLNM) without children

**Supplementary Figure 54** | Forest plot of age under 45 and its influence in case of central lymph node metastasis (CLNM) without children


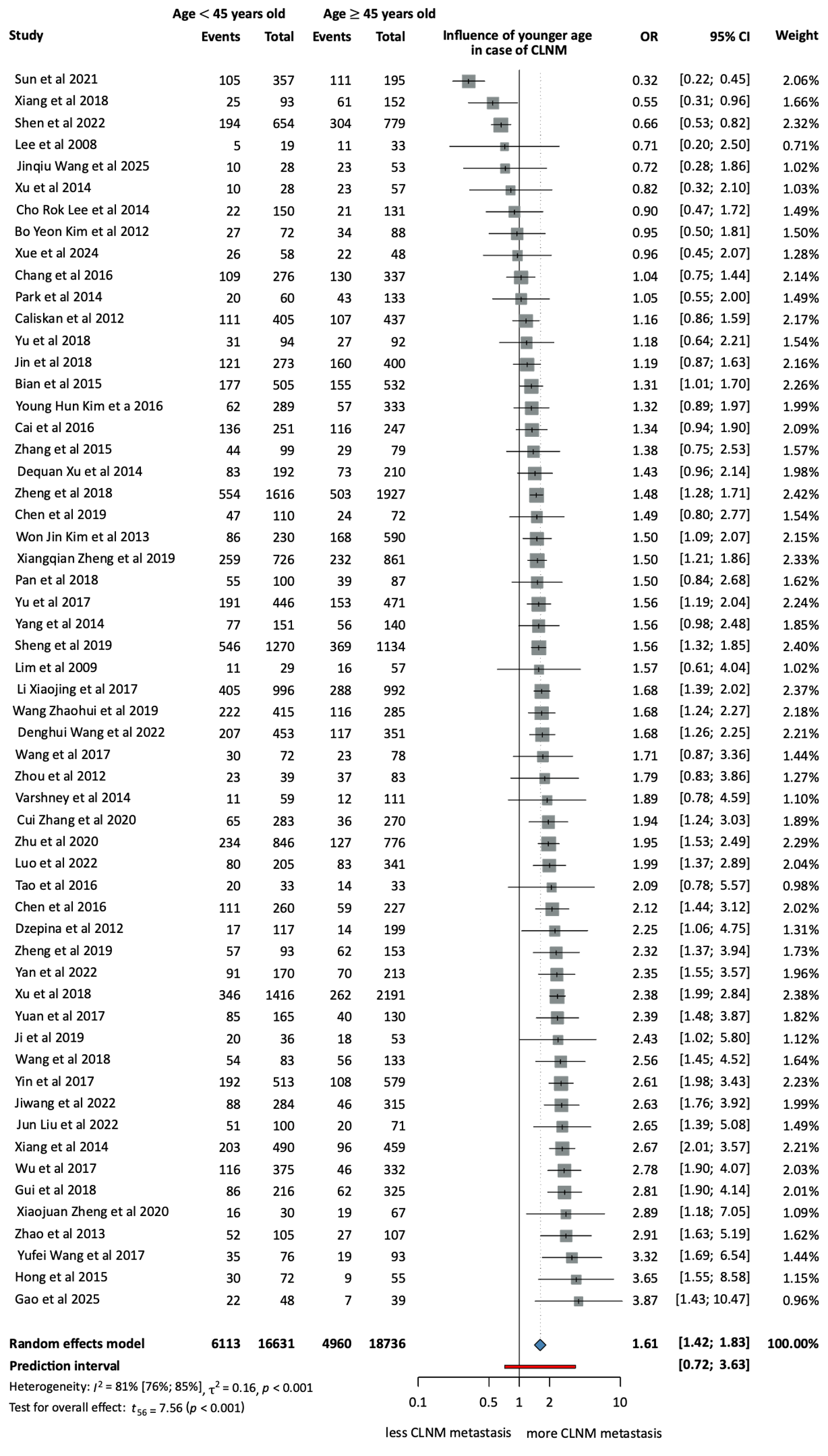


**Supplementary Figure 55** | Forest plot of age under 55 and its influence in case of central lymph node metastasis (CLNM) without children


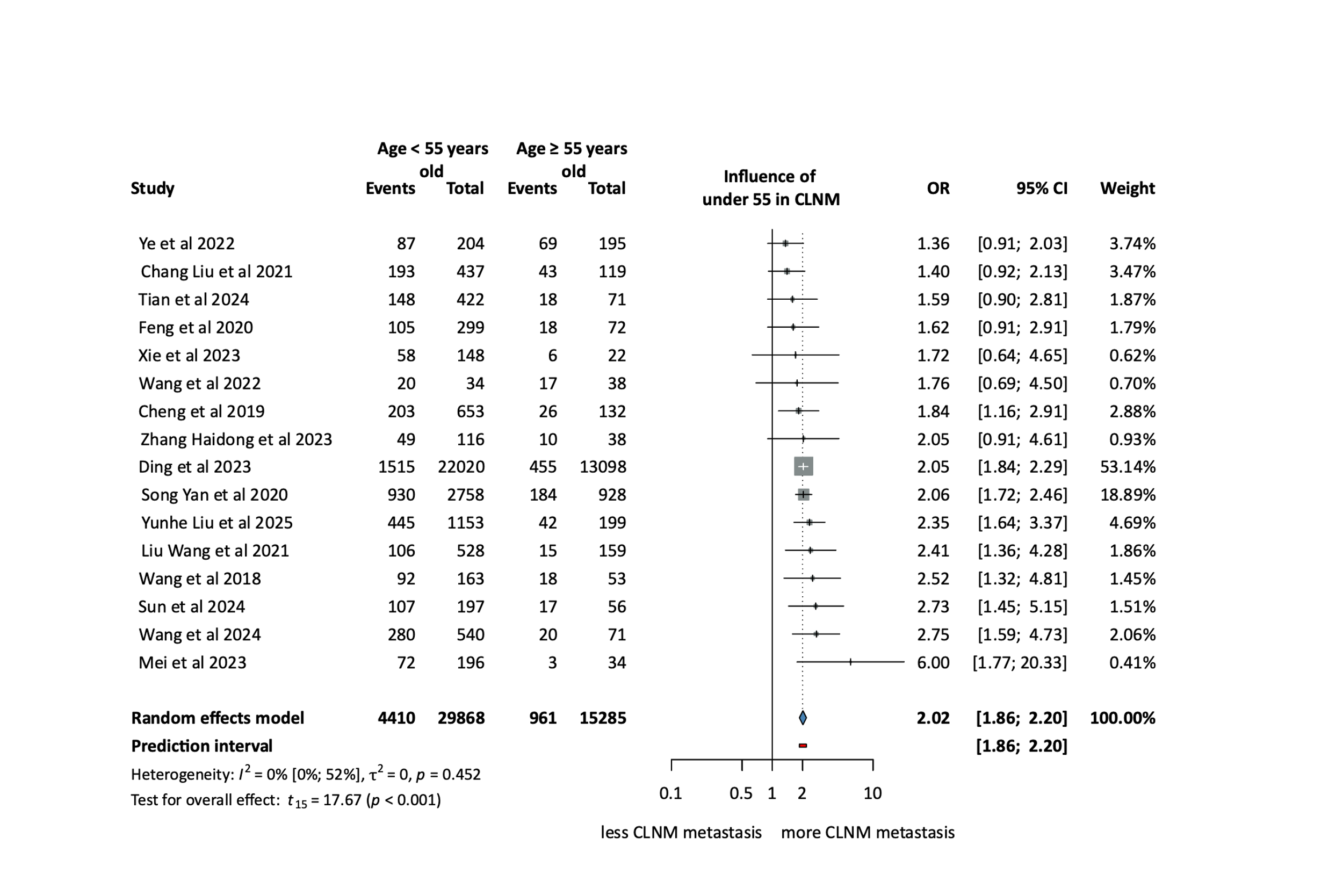


**Supplementary Figure 56** | Forest plot of tumor size above 5 mm and its influence in case of central lymph node metastasis (CLNM) without children


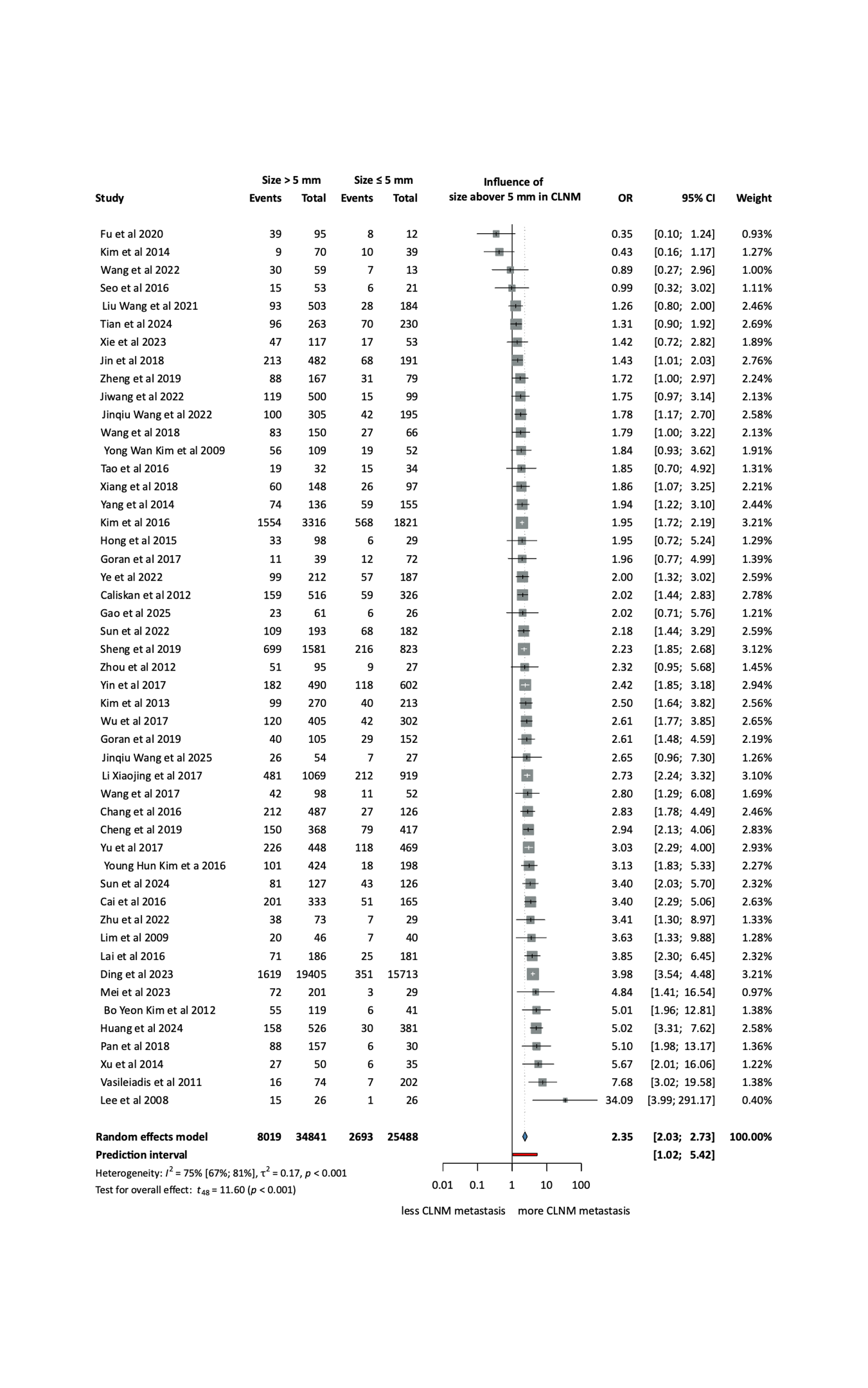


**Supplementary Figure 57** | Forest plot of tumor size above 6.5 mm and its influence in case of central lymph node metastasis (CLNM) without children


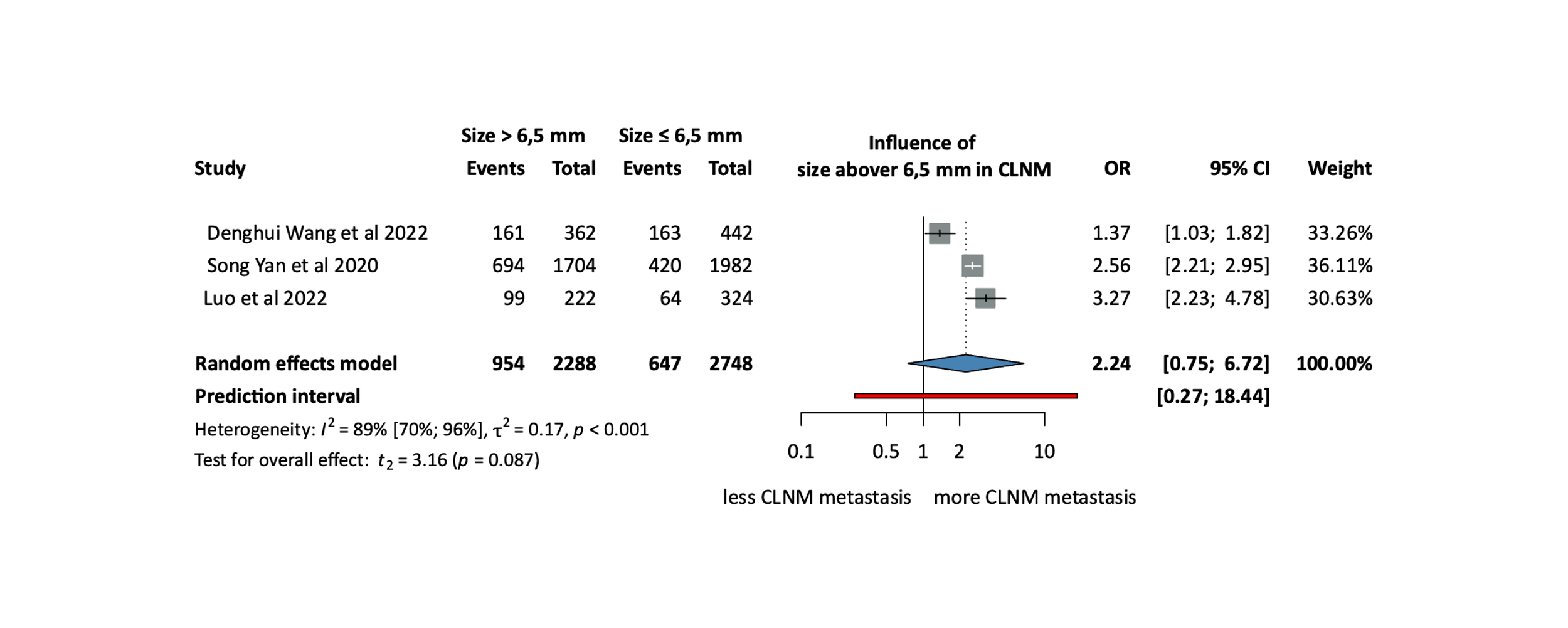


**Supplementary Figure 58** | Forest plot of tumor size above 7 mm and its influence in case of central lymph node metastasis (CLNM) without children


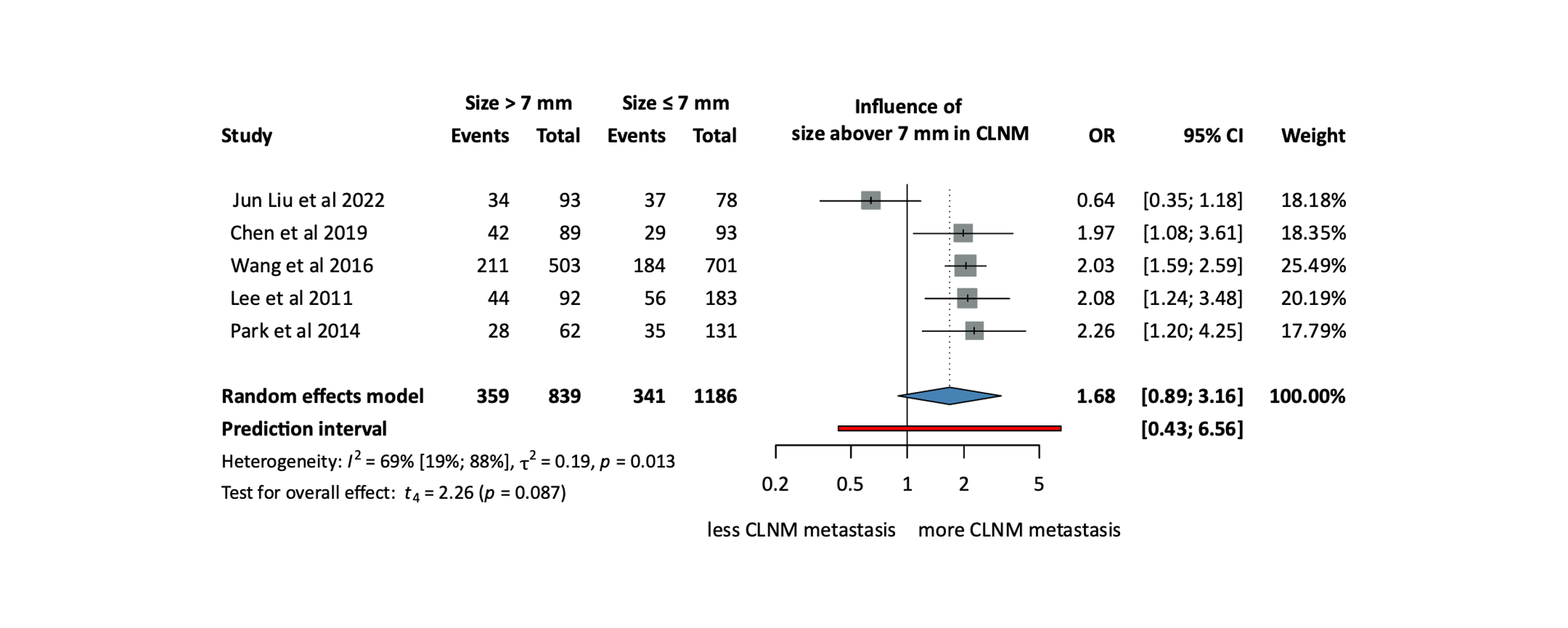


**Supplementary Figure 59** | Forest plot of multifocality and its influence in case of central lymph node metastasis (CLNM) without children


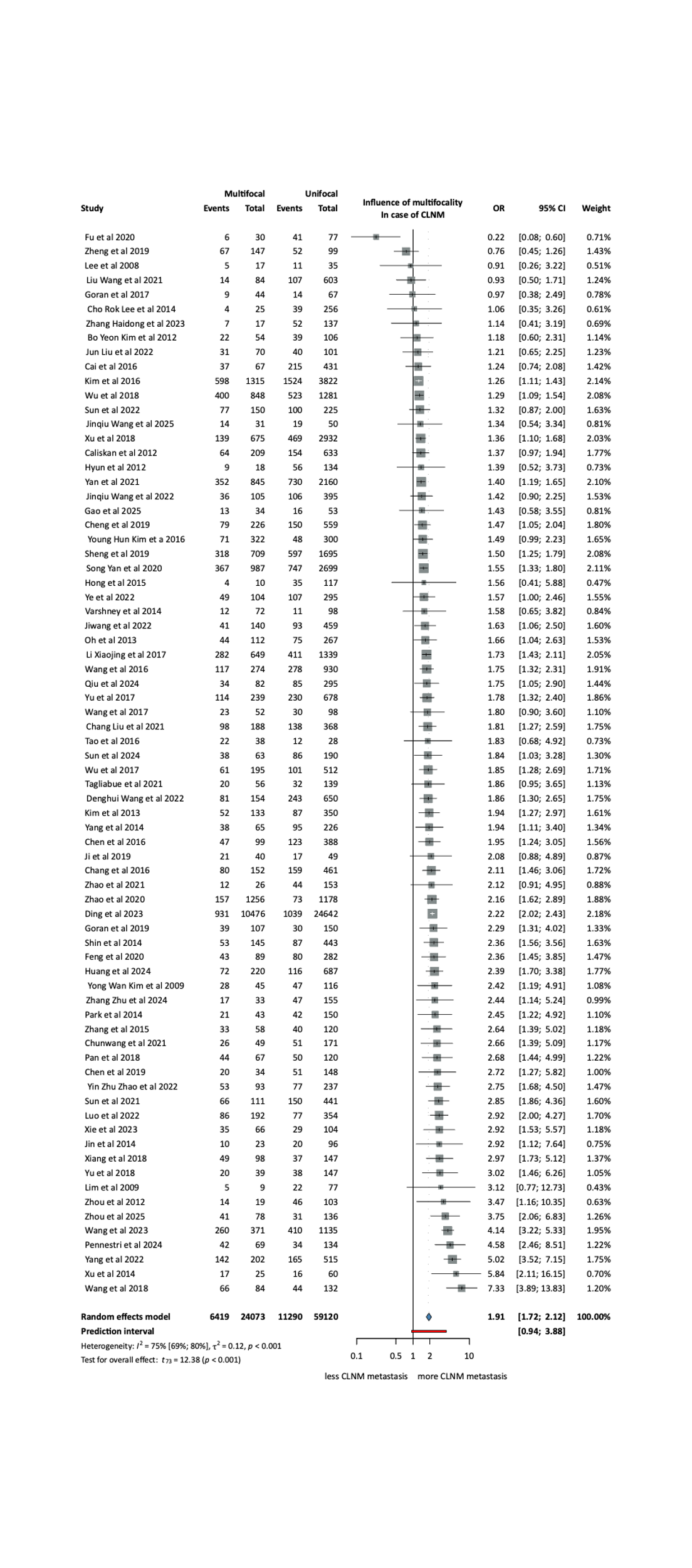


**Supplementary Figure 60** | Forest plot of bilaterality and its influence in case of central lymph node metastasis (CLNM) without children


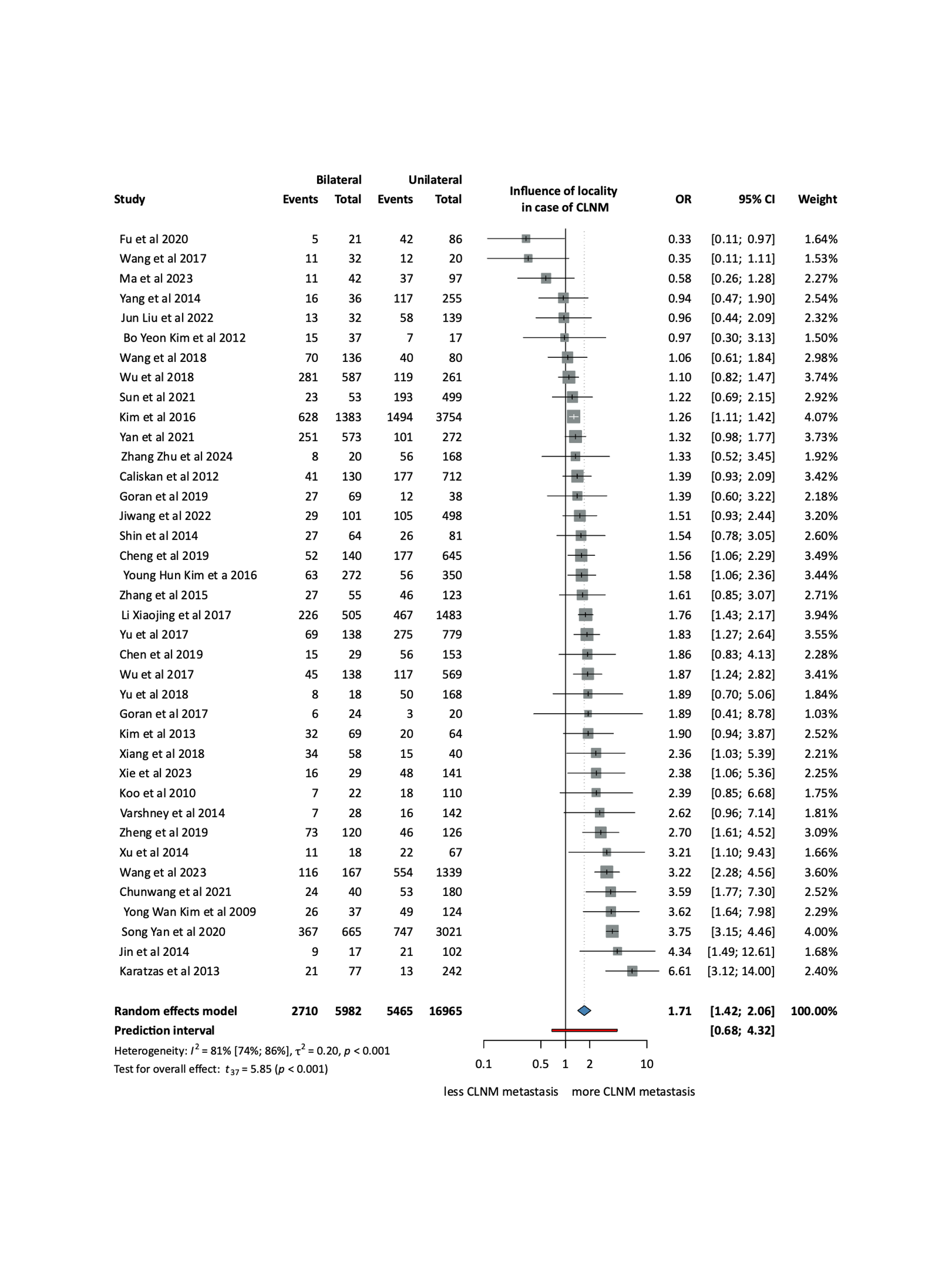


**Supplementary Figure 61** | Forest plot of BRAF^V600E^ mutation and its influence in case of central lymph node metastasis (CLNM) without children


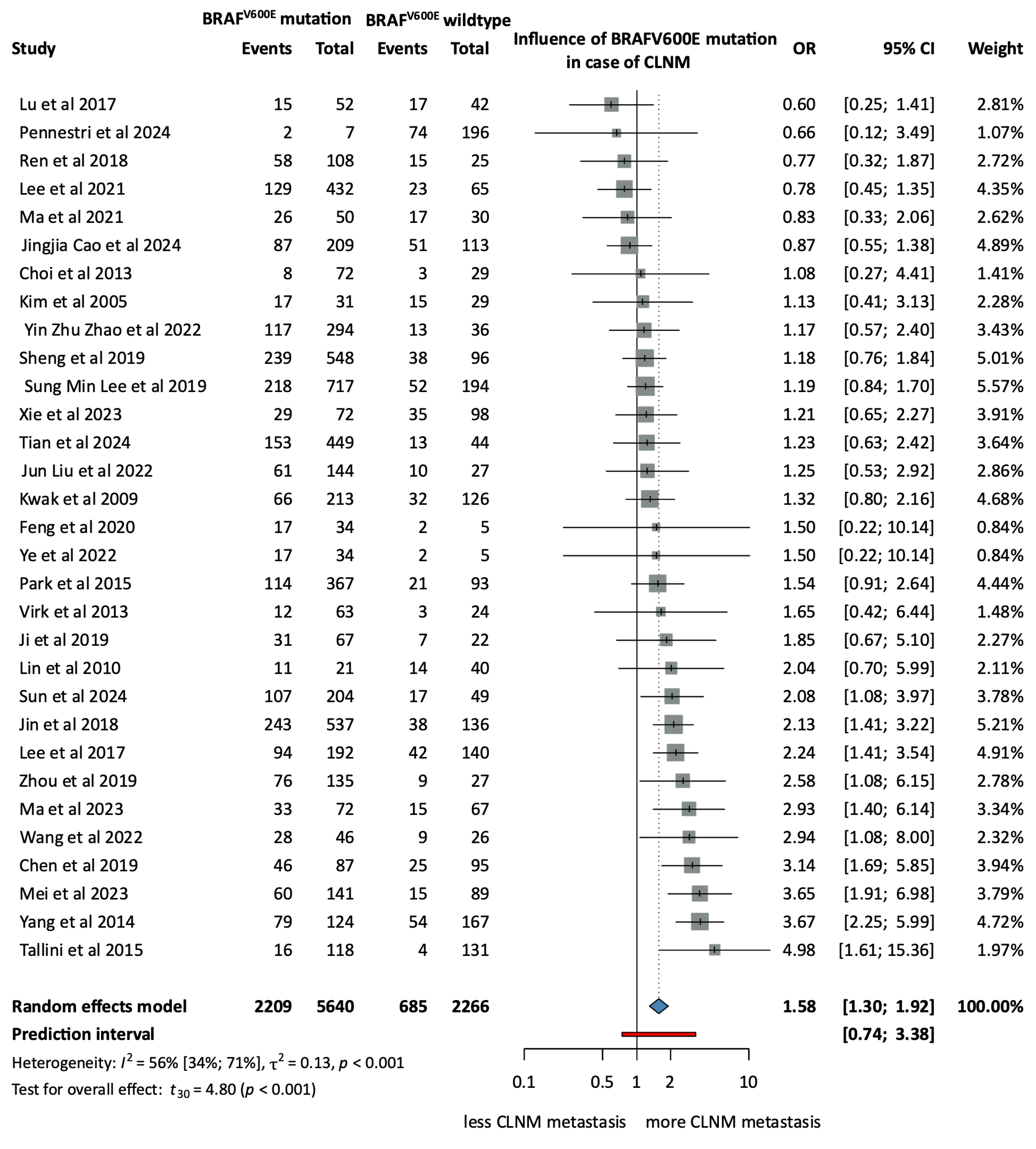


**Supplementary Figure 62** | Forest plot of Hashimoto’s thyroiditis and its influence in case of central lymph node metastasis (CLNM) without children


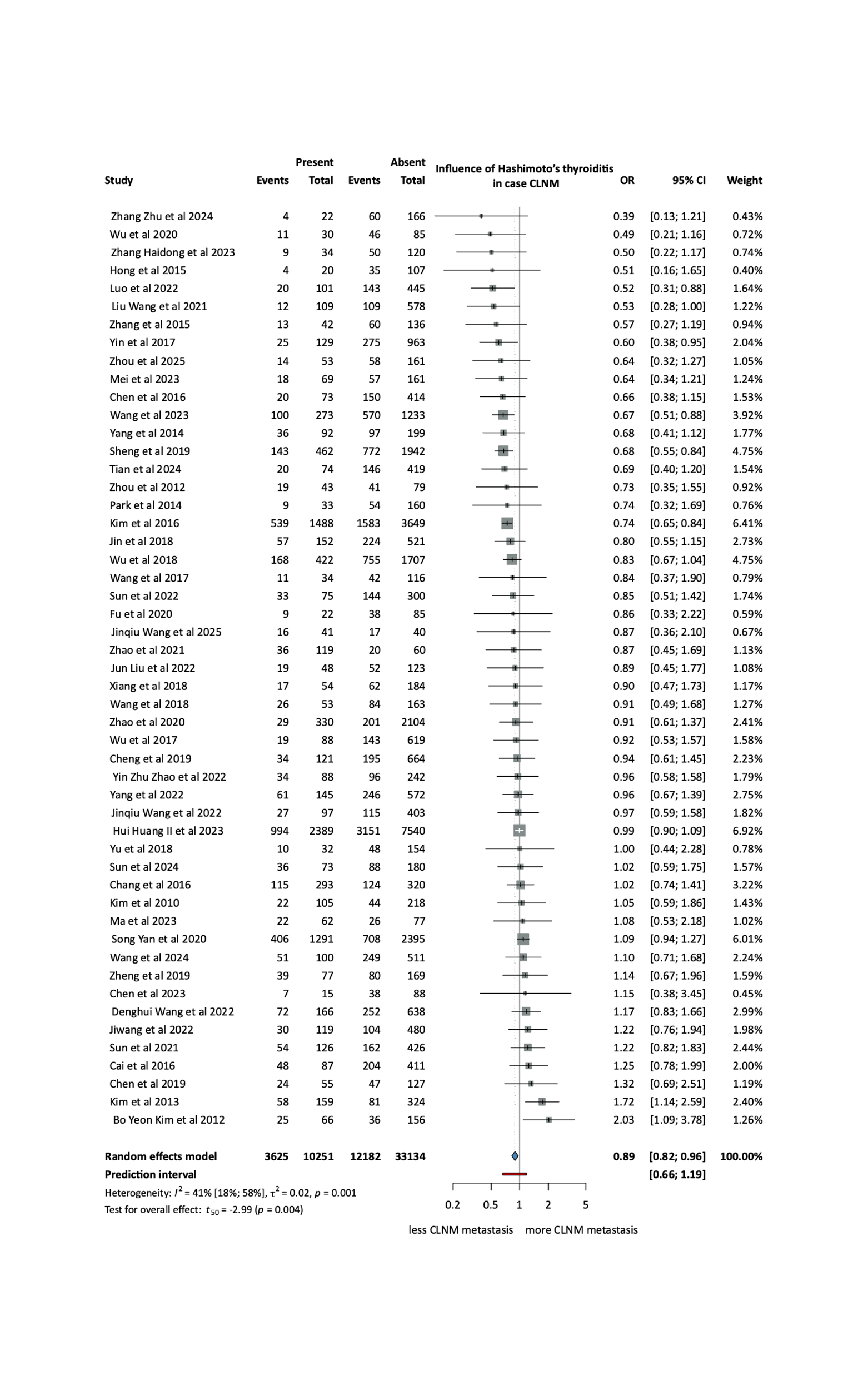


**Supplementary Figure 63** | Forest plot of obesity and its influence in case of central lymph node metastasis (CLNM) without children


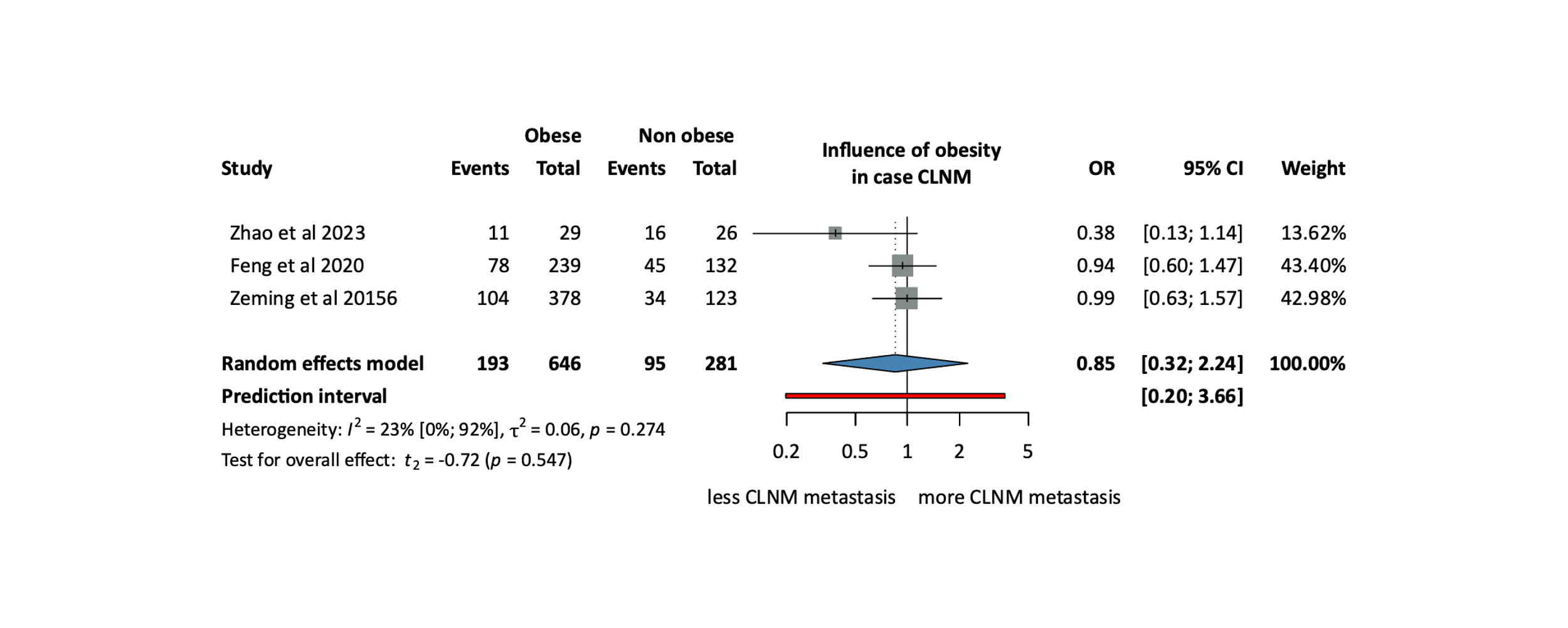


**Supplementary Figure 64** | Forest plot of male gender and its influence in case of lateral lymph node metastasis (LLNM) without children

**Supplementary Figure 65** | Forest plot of age under 45 and its influence in case of lateral lymph node metastasis (LLNM) without children

**Supplementary Figure 66** | Forest plot of age under 55 and its influence in case of lateral lymph node metastasis (LLNM) without children

**Supplementary Figure 67** | Forest plot of tumor size above 5 mm and its influence in case of lateral lymph node metastasis (LLNM) without children

**Supplementary Figure 68** | Forest plot of tumor size above 7 mm and its influence in case of lateral lymph node metastasis (LLNM) without children

**Supplementary Figure 69** | Forest plot of multifocality and its influence in case of lateral lymph node metastasis (LLNM) without children

**Supplementary Figure 70** | Forest plot of bilaterality and its influence in case of lateral lymph node metastasis (LLNM) without children

**Supplementary Figure 71** | Forest plot of BRAF^V600E^ mutation and its influence in case of lateral lymph node metastasis (LLNM) without children

**Supplementary Figure 72** | Forest plot of Hashimoto’s thyroiditis and its influence in case of lateral lymph node metastasis (LLNM) without children

**Supplementary Figure 73** | Forest plot of male gender and its influence in case of undetermined lymph node metastasis (udLNM) without children

**Supplementary Figure 74** | Forest plot of age under 45 and its influence in case of undetermined lymph node metastasis (udLNM) without children

**Supplementary Figure 75** | Forest plot of under 55 and its influence in case of undetermined lymph node metastasis (udLNM) without children

**Supplementary Figure 76** | Forest plot of tumor size above 5 mm and its influence in case of undetermined lymph node metastasis (udLNM) without children

**Supplementary Figure 77** | Forest plot of multifocality and its influence in case of undetermined lymph node metastasis (udLNM) without children

**Supplementary Figure 78** | Forest plot of bilaterality and its influence in case of undetermined lymph node metastasis (udLNM) without children

**Supplementary Figure 79** | Forest plot of BRAF^V600E^ mutation and its influence in case of undetermined lymph node metastasis (udLNM) without children

**Supplementary Figure 80** | Forest plot of Hashimoto’s thyroiditis and its influence in case of undetermined lymph node metastasis (udLNM) without children

**Supplementary Figure 81** | Forest plot of male gender and its influence in case of extrathyroidal extension (ETE) without children

**Supplementary Figure 82** | Forest plot of age under 45 and its influence in case of extrathyroidal extension (ETE) without children

**Supplementary Figure 83** | Forest plot of age under 55 and its influence in case of extrathyroidal extension (ETE) without children

**Supplementary Figure 84** | Forest plot of tumor size above 5 mm and its influence in case of extrathyroidal extension (ETE) without children

**Supplementary Figure 85** | Forest plot of multifocality and its influence in case of extrathyroidal extension (ETE) without children

**Supplementary Figure 86** | Forest plot of bilaterality and its influence in case of extrathyroidal extension (ETE) without children

**Supplementary Figure 87** | Forest plot of BRAF^V600E^ mutation and its influence in case of extrathyroidal extension (ETE) without children

**Supplementary Figure 88** | Forest plot of Hashimoto’s thyroiditis and its influence in case of extrathyroidal extension (ETE) without children

As an outlier, we identified the following articles: Zhang et al, 2024 (Hashimoto-thyroiditis – CLNM) [111], Fu et al, 2020 (multifocality – CLNM) [6], Mei et al, 2023 (age 55 y.o.> – CLNM) [262], Lee et al, 2008 (size 5 mm < – CLNM) [51], Jun et al, 2022 (size 7 mm < – CLNM) [36], Kaliszewski et al, 2020 (age 55 y.o.> – LNM) [10], Yesiloglu et al, 2024 (size 5 mm < – LNM) [38], Kaliszewski et al, 2019 (size 5 mm < – LNM) [196], Korkmaz et al, 2016 (multifocality – LNM) [183], Yesiloglu et al, 2024 (multifocality – LNM) [38], Sohee-Lee et al, 2019 (multifocality – LNM) [11], Rossi et al, 2012 (BRAF^V600E^ – LNM) [117], Rodolico et al, 2007 (locality – LNM) [206], Kaliszewski et al, 2019 (locality – LNM) [196], Moon et al, 2011 (Hashimoto-thyroiditis – ETE) [70], Pardo et al, 2021 (age 45 y.o.> – ETE) [256], Lombardi et al, 2010 (age 45 y.o.> – ETE) [60].

c. METAREGRESSION

**Supplementary Table 4** | Metaregression analysis in the case of age and tumor size

CLNM – age

|  | odds | CI lower | CI upper | p-value |
| --- | --- | --- | --- | --- |
| intrcpt | 253.127 | 83.848 | 765.157 | 0 |
| predictor | 0.871 | 0.850 | 0.893 | 0 |

LLNM – age

|  | odds | CI lower | CI upper | p-value |
| --- | --- | --- | --- | --- |
| intrcpt | 21.998 | 0.191 | 2536.949 | 0.10731 |
| predictor | 0.887 | 0.795 | 0.991 | 0.04315 |

udLNM – age

|  | odds | CI lower | CI upper | p-value |
| --- | --- | --- | --- | --- |
| intrcpt | 189.741 | 8.047 | 4473.952 | 0.01323 |
| predictor | 0.871 | 0.812 | 0.933 | 0.00786 |

CLNM – tumor size

|  | odds | CI lower | CI upper | p-value |
| --- | --- | --- | --- | --- |
| intrcpt | 0.000 | 0.000 | 0.000 | 1e-05 |
| predictor | 4.717 | 3.721 | 5.979 | 1e-05 |

LLNM – tumor size

|  | odds | CI lower | CI upper | p-value |
| --- | --- | --- | --- | --- |
| intrcpt | 0.506 | 0.128 | 1.998 | 0.16632 |
| predictor | 0.595 | 0.467 | 0.758 | 0.01154 |

We analysed with metaregression the continous variables in case of age and tumor size also. As a result, we could say that younger patients and people with larger tumor size tend to develope lymph node metastasis.

d. RISK OF BIAS

**QUIPS appendix**

Study participation

- low-risk: PTMC diagnosis based on histology
- moderate-risk: subgroup of patients with PTMC
- high-risk: PTMC diagnosis without histology

Study attrition

- dropout during follow-up in case of a prospective study: high risk
- in case of retrospective study the risk can not be applied

Prognostic factor

- sex
  - if the sex is reported than the risk will be automatically low
- age
  - low-risk: accurate birth date or mean +/- SD or median +/- IQR or range
  - moderate-risk: age categories
  - high-risk: -
- tumor size
  - low-risk: histologically confirmed and exact size (individually) or mean +/- SD or median +/- IQR or range
  - moderate-risk: measured by US and/or size categories
  - high-risk: unclear confirmation / absent information
- body mass index (BMI)
  - low-risk: accurate calculation
  - moderate-risk: -
  - high-risk: self-declaration
- multifocality
  - low-risk: histologically confirmed
  - moderate-risk: US confirmed
  - high-risk: unclear confirmation / absent information
- bilaterality
  - low-risk: histologically confirmed
  - moderate-risk: US confirmed
  - high-risk: unclear confirmation / absent information
- intraglandular localization
  - low-risk: histologically or intraoperatively confirmed
  - moderate-risk: US confirmed
  - high-risk: unclear confirmation / absent information
- Hashimoto’s thyreoiditis
  - low-risk: histologically confirmed
  - moderate-risk: US or laboratory confirmation
  - high-risk: unclear confirmation / absent information
- BRAFV600E mutation
  - low-risk: usement of standardized genetic kit/tool
  - moderate-risk: -
  - high-risk: unclear genetical examination / absent information
- pregnancy
  - low-risk: determined by medical professionals
  - moderate-risk: -
  - high-risk: self-declaration
- TERT or other mutations
  - low-risk: usement of standardized genetic kit/tool
  - moderate-risk: -
  - high-risk: unclear genetical examination / absent information
- thyreoglobulin antibody
  - low-risk: usement of standardized tool and continous variable
  - moderate-risk: dichotomous variable
  - high-risk: unclear examination / absent information
- goiter
  - low-risk: histologically confirmed
  - moderate-risk: US confirmation
  - high-risk: unclear confirmation / absent information
- Graves’ disease
  - low-risk: histologically confirmed and/or clinical symptomps and/or specific antibodies
  - moderate-risk: -
  - high-risk: unclear confirmation / absent information
- TSH
  - low-risk: use of standardized tool and continuous variable
  - moderate-risk: dichotomous variable
  - high-risk: unclear confirmation / absent information
- capsule
  - low-risk: histologically confirmed
  - moderate-risk: US confirmation
  - high-risk: unclear confirmation / absent information
- calcification
  - low-risk: histologically confirmed
  - moderate-risk: US confirmation
  - high-risk: unclear confirmation / absent information

Outcome measurement

- lymph node metastasis
  - low-risk: histologically confirmed and accurate definiton (central and/or lateral lymph node metastasis)
  - moderate-risk: histologically confirmed but inaccurate definition (lymph node metastasis)
  - high-risk: radiologically confirmed diagnosis
- extrathyroid extension
  - low-risk: histologically confirmed and accurate definition (macroscopic or microscopic extension)
  - moderate-risk: radiological confirmed
  - high-risk: unclear confirmation
- tumor size increase
  - low-risk: accurate definition (at least 3 mm size increase)
  - moderate-risk: -
  - high-risk: inaccurate definition

Study confounding

- low-risk: clear method, every detail is well known
- moderate-risk: multivariable analysis without known adjustments
- high-risk: no multivariable analysis

Statistical analysis reporting

- low-risk: clear and accurate reporting
- moderate-risk: insufficient reporting
- high-risk: missing of statistical modell

Overall risk of bias:

- low-risk: everything is low-risk +/- 1 moderate-risk
- moderate-risk: low-risk +/- 1 high-risk or low-risk + 2 moderate-risk

high-risk: if there are at least 1 moderate-risk and high-risk

**Supplementary Table 5.** | Risk of Bias Assassment

| **Study** | **Study participation** | **Study attrition** | **Prognostic factor measurement - patients and tumor characteristics** | | | | | | | | | | | | | | | | | **Outcome measurement** | | | **Study confounding** | **Statistical analysis reporting** | **Overall risk of bias** |
| --- | --- | --- | --- | --- | --- | --- | --- | --- | --- | --- | --- | --- | --- | --- | --- | --- | --- | --- | --- | --- | --- | --- | --- | --- | --- |
|  |  |  | **Sex** | **Age** | **Size** | **BMI** | **Multifocality** | **Bilaterality** | **Lobe** | **Hashimoto's thyroiditis** | **BRAFV600E mutation** | **TERT or others mutations** | **Capsule** | **Calcification** | **TSH** | **anti-TG** | **Goiter** | **Graves's disease** | **Pregnancy** | **lymph node metastasis** | **extrathyroidal**  **extension** | **size increase** |  |  |  |
| Kim et al. 2020 | + | + | n.a. | n.a. | n.a. | n.a. | n.a. | n.a. | n.a. | n.a. | + | n.a. | n.a. | n.a. | n.a. | n.a. | n.a. | n.a. | n.a. | ! | + | n.a. | ! | + | **high** |
| Choi et al. 2020 | + | + | + | ! | n.a. | n.a. | - | n.a. | n.a. | n.a. | n.a. | n.a. | + | n.a. | n.a. | n.a. | n.a. | n.a. | n.a. | ! | n.a. | n.a. | ! | + | **high** |
| Besic et al. 2009 | + | + | + | ! | ! | n.a. | + | n.a. | n.a. | n.a. | n.a. | n.a. | n.a. | n.a. | n.a. | n.a. | n.a. | n.a. | n.a. | ! | n.a. | n.a. | ! | + | **high** |
| Cai et al. 2016 | + | + | + | ! | ! | n.a. | + | n.a. | ! | - | n.a. | n.a. | + | ! | n.a. | n.a. | n.a. | n.a. | n.a. | + | n.a. | n.a. | ! | + | **high** |
| Ding et al. 2023 | + | + | + | ! | ! | n.a. | + | n.a. | n.a. | n.a. | n.a. | n.a. | n.a. | n.a. | n.a. | n.a. | n.a. | n.a. | n.a. | + | n.a. | n.a. | ! | + | **high** |
| Fu et al. 2020 | + | + | + | n.a. | ! | n.a. | n.a. | n.a. | n.a. | - | n.a. | n.a. | n.a. | n.a. | n.a. | n.a. | ! | n.a. | n.a. | + | n.a. | n.a. | ! | + | **high** |
| Ping Yang et al. 2022 | + | + | + | ! | n.a. | + | + | n.a. | + | n.a. | n.a. | n.a. | n.a. | n.a. | n.a. | n.a. | n.a. | n.a. | n.a. | + | n.a. | n.a. | - | + | **high** |
| Apostol et al. 2017 | + | + | n.a. | + | + | n.a. | + | + | n.a. | + | n.a. | n.a. | n.a. | n.a. | n.a. | n.a. | + | + | n.a. | ! | + | n.a. | ! | + | **moderate** |
| Liu et al. 2014 | + | + | n.a. | n.a. | n.a. | n.a. | n.a. | n.a. | n.a. | + | n.a. | n.a. | n.a. | n.a. | n.a. | n.a. | n.a. | n.a. | n.a. | ! | n.a. | n.a. | ! | + | **moderate** |
| Jin et al. 2023 | + | + | + | + | ! | n.a. | ! | n.a. | ! | ! | + | n.a. | n.a. | ! | n.a. | n.a. | n.a. | n.a. | n.a. | + | n.a. | n.a. | ! | + | **high** |
| Kaliszewski et al. 2020 | + | + | + | ! | n.a. | n.a. | n.a. | n.a. | n.a. | n.a. | n.a. | n.a. | n.a. | + | n.a. | n.a. | n.a. | n.a. | n.a. | ! | n.a. | n.a. | - | + | **high** |
| Sohee Lee et al. 2019 | + | + | + | + | + | n.a. | + | n.a. | n.a. | n.a. | + | n.a. | n.a. | n.a. | n.a. | n.a. | n.a. | n.a. | n.a. | + | n.a. | n.a. | ! | + | **low** |
| Lin et al. 2005 | + | + | + | n.a. | n.a. | n.a. | n.a. | n.a. | n.a. | n.a. | n.a. | n.a. | n.a. | n.a. | n.a. | n.a. | n.a. | n.a. | n.a. | ! | n.a. | n.a. | - | + | **high** |
| Amendola et al. 2024 | + | + | + | + | + | n.a. | + | n.a. | n.a. | + | n.a. | n.a. | + | n.a. | n.a. | n.a. | n.a. | n.a. | n.a. | + | n.a. | n.a. | ! | + | **low** |
| Xiang et al. 2018 | + | + | + | ! | ! | n.a. | + | + | n.a. | - | n.a. | n.a. | - | n.a. | n.a. | n.a. | n.a. | n.a. | n.a. | + | n.a. | n.a. | + | + | **high** |
| Zhang et al. 2016 | + | + | + | ! | ! | n.a. | + | n.a. | n.a. | - | n.a. | n.a. | - | n.a. | n.a. | n.a. | n.a. | n.a. | n.a. | ! | n.a. | n.a. | - | + | **high** |
| Zhu et al. 2020 | + | + | + | ! | ! | n.a. | n.a. | n.a. | n.a. | n.a. | n.a. | n.a. | ! | n.a. | n.a. | n.a. | n.a. | n.a. | n.a. | + | n.a. | n.a. | ! | + | **high** |
| Han et al. 2022 | + | + | n.a. | ! | n.a. | n.a. | n.a. | n.a. | n.a. | n.a. | n.a. | n.a. | n.a. | n.a. | n.a. | n.a. | n.a. | n.a. | n.a. | + | n.a. | n.a. | ! | + | **moderate** |
| Liu et al. 2022 | + | + | + | n.a. | n.a. | n.a. | n.a. | n.a. | n.a. | n.a. | n.a. | n.a. | n.a. | n.a. | n.a. | n.a. | n.a. | n.a. | n.a. | ! | n.a. | n.a. | - | + | **high** |
| Shi et al. 2022 | + | + | + | ! | + | n.a. | + | + | n.a. | + | + | n.a. | n.a. | n.a. | n.a. | n.a. | n.a. | n.a. | n.a. | ! | n.a. | n.a. | - | + | **high** |
| Nam Seop Lee et al. 2010 | + | + | + | ! | ! | n.a. | + | n.a. | n.a. | n.a. | n.a. | n.a. | - | n.a. | n.a. | n.a. | n.a. | n.a. | n.a. | ! | n.a. | n.a. | - | + | **high** |
| Jeong et al. 2017 | + | + | + | ! | + | n.a. | + | + | n.a. | n.a. | n.a. | n.a. | - | n.a. | n.a. | n.a. | n.a. | n.a. | n.a. | + | n.a. | n.a. | ! | + | **high** |
| Luo et al. 2022 | + | + | + | ! | ! | n.a. | + | n.a. | n.a. | ! | n.a. | n.a. | ! | n.a. | n.a. | n.a. | + | n.a. | n.a. | + | n.a. | n.a. | ! | + | **high** |
| Iscan et al. 2019 | + | + | n.a. | n.a. | ! | n.a. | n.a. | n.a. | n.a. | n.a. | n.a. | n.a. | n.a. | n.a. | n.a. | n.a. | n.a. | n.a. | n.a. | ! | n.a. | n.a. | - | + | **high** |
| Medas et al. 2020 | + | + | + | ! | ! | n.a. | + | n.a. | n.a. | ! | n.a. | n.a. | n.a. | n.a. | n.a. | n.a. | n.a. | n.a. | n.a. | ! | + | n.a. | - | + | **high** |
| Ye et al. 2022 | + | + | + | ! | ! | n.a. | + | n.a. | n.a. | n.a. | ! | n.a. | n.a. | n.a. | n.a. | n.a. | n.a. | n.a. | n.a. | + | n.a. | n.a. | ! | + | **high** |
| Wang et al. 2023 | + | + | + | + | + | n.a. | + | + | ! | + | n.a. | n.a. | ! | ! | n.a. | n.a. | n.a. | n.a. | n.a. | + | n.a. | n.a. | ! | + | **high** |
| Yin et al. 2021 | + | + | + | ! | ! | + | + | + | n.a. | + | + | n.a. | + | n.a. | n.a. | n.a. | n.a. | n.a. | n.a. | ! | n.a. | n.a. | + | + | **high** |
| Zhao et al. 2021 | + | + | + | + | n.a. | n.a. | - | n.a. | - | - | n.a. | n.a. | - | n.a. | n.a. | n.a. | n.a. | n.a. | n.a. | + | n.a. | n.a. | ! | + | **high** |
| Cheng et al. 2019 | + | + | + | ! | ! | n.a. | + | + | n.a. | - | n.a. | n.a. | n.a. | n.a. | n.a. | n.a. | n.a. | n.a. | n.a. | + | n.a. | n.a. | ! | + | **high** |
| Peng et al. 2016 | + | + | + | ! | ! | n.a. | + | + | n.a. | - | n.a. | n.a. | - | n.a. | n.a. | n.a. | n.a. | n.a. | n.a. | ! | n.a. | n.a. | - | + | **high** |
| Zhang et al. 2018 | + | + | + | ! | ! | n.a. | + | + | n.a. | - | + | n.a. | ! | n.a. | ! | ! | n.a. | n.a. | n.a. | + | n.a. | n.a. | - | + | **high** |
| Bircan et al. 2014 | + | + | n.a. | n.a. | n.a. | n.a. | n.a. | n.a. | n.a. | + | n.a. | n.a. | n.a. | n.a. | n.a. | n.a. | n.a. | n.a. | n.a. | + | n.a. | n.a. | ! | + | **low** |
| Li et al. 2017 | + | + | + | ! | ! | n.a. | + | n.a. | + | - | n.a. | n.a. | - | n.a. | n.a. | n.a. | + | n.a. | n.a. | + | n.a. | n.a. | ! | + | **high** |
| Nechifor Boila et al. 2018 | + | + | n.a. | n.a. | n.a. | n.a. | n.a. | n.a. | n.a. | n.a. | + | n.a. | n.a. | n.a. | n.a. | n.a. | n.a. | n.a. | n.a. | n.a. | + | n.a. | ! | + | **low** |
| Liu et al. 2016 | + | + | n.a. | n.a. | n.a. | n.a. | n.a. | n.a. | n.a. | + | n.a. | n.a. | n.a. | n.a. | n.a. | n.a. | n.a. | n.a. | n.a. | ! | n.a. | n.a. | + | + | **low** |
| Zeming Liu et al. 2017 | + | + | + | ! | ! | n.a. | + | n.a. | n.a. | - | n.a. | n.a. | n.a. | n.a. | n.a. | n.a. | n.a. | n.a. | n.a. | ! | + | n.a. | + | + | **high** |
| Chunping Liu et al. 2017 | + | + | + | ! | ! | n.a. | + | n.a. | n.a. | - | n.a. | n.a. | n.a. | n.a. | n.a. | n.a. | n.a. | n.a. | n.a. | ! | + | n.a. | + | + | **high** |
| Zhang et al. 2017 | + | + | + | ! | ! | n.a. | + | n.a. | n.a. | - | n.a. | n.a. | - | n.a. | n.a. | n.a. | n.a. | n.a. | n.a. | ! | n.a. | n.a. | ! | + | **high** |
| Zheng et al. 2020 | + | + | n.a. | n.a. | n.a. | n.a. | n.a. | n.a. | n.a. | n.a. | + | n.a. | n.a. | n.a. | n.a. | n.a. | n.a. | n.a. | n.a. | ! | n.a. | n.a. | - | + | **high** |
| Lee et al. 2014 | + | + | + | n.a. | n.a. | n.a. | - | n.a. | n.a. | - | n.a. | n.a. | n.a. | n.a. | n.a. | n.a. | n.a. | n.a. | n.a. | n.a. | + | n.a. | - | + | **high** |
| Dzepina et al. 2012 | + | + | + | ! | n.a. | n.a. | n.a. | n.a. | n.a. | n.a. | n.a. | n.a. | n.a. | n.a. | n.a. | n.a. | n.a. | n.a. | n.a. | + | n.a. | n.a. | ! | + | **moderate** |
| Yufei Wang et al. 2017 | + | + | + | ! | n.a. | n.a. | + | + | n.a. | + | n.a. | n.a. | + | n.a. | n.a. | n.a. | n.a. | n.a. | n.a. | + | n.a. | n.a. | + | + | **low** |
| Wang et al. 2017 | + | + | + | ! | ! | n.a. | + | n.a. | + | + | n.a. | n.a. | + | n.a. | n.a. | n.a. | n.a. | n.a. | n.a. | + | n.a. | n.a. | - | + | **high** |
| Yoon et al. 2017 | + | + | + | + | ! | n.a. | n.a. | n.a. | n.a. | n.a. | n.a. | n.a. | n.a. | n.a. | n.a. | n.a. | n.a. | n.a. | n.a. | + | + | n.a. | + | + | **low** |
| Yan et al. 2019 | + | + | + | + | + | n.a. | + | n.a. | n.a. | n.a. | n.a. | n.a. | n.a. | n.a. | n.a. | n.a. | n.a. | n.a. | n.a. | ! | n.a. | n.a. | - | + | **high** |
| Dirikoc et al. 2021 | + | + | + | + | + | n.a. | - | n.a. | n.a. | - | n.a. | n.a. | - | n.a. | n.a. | n.a. | n.a. | n.a. | n.a. | ! | n.a. | n.a. | ! | + | **high** |
| Denghui Wang et al. 2022 | + | + | + | ! | ! | n.a. | + | n.a. | + | ! | n.a. | n.a. | ! | ! | n.a. | + | n.a. | n.a. | n.a. | + | n.a. | n.a. | - | + | **high** |
| Chang et al. 2016 | + | + | + | ! | - | n.a. | - | n.a. | n.a. | - | n.a. | n.a. | n.a. | n.a. | n.a. | n.a. | n.a. | n.a. | n.a. | + | n.a. | n.a. | ! | + | **high** |
| Jun Liu et al. 2022 | + | + | + | ! | ! | n.a. | + | + | n.a. | - | + | n.a. | n.a. | ! | + | n.a. | n.a. | n.a. | n.a. | + | n.a. | n.a. | ! | + | **high** |
| Zhao et al. 2020 | + | + | + | + | + | + | + | n.a. | n.a. | + | n.a. | n.a. | + | n.a. | n.a. | ! | n.a. | n.a. | n.a. | + | n.a. | n.a. | ! | + | **moderate** |
| Yesiloglu et al. 2024 | + | + | n.a. | n.a. | ! | n.a. | + | n.a. | n.a. | n.a. | + | + | n.a. | n.a. | n.a. | n.a. | n.a. | n.a. | n.a. | ! | + | n.a. | - | + | **high** |
| Tam et al. 2017 | + | + | + | ! | + | n.a. | + | + | n.a. | ! | n.a. | n.a. | - | n.a. | n.a. | ! | n.a. | n.a. | n.a. | ! | n.a. | n.a. | - | + | **high** |
| Zhao et al. 2024 | + | + | n.a. | n.a. | n.a. | n.a. | n.a. | n.a. | n.a. | n.a. | + | n.a. | n.a. | n.a. | n.a. | n.a. | n.a. | n.a. | n.a. | ! | n.a. | n.a. | ! | + | **moderate** |
| Yunjun Wang et al. 2018 | + | + | + | ! | ! | n.a. | + | + | n.a. | ! | n.a. | n.a. | n.a. | n.a. | ! | n.a. | n.a. | n.a. | n.a. | + | n.a. | n.a. | ! | + | **high** |
| Sheng et al. 2019 | + | + | + | ! | ! | n.a. | + | n.a. | n.a. | + | + | n.a. | n.a. | n.a. | n.a. | n.a. | + | n.a. | n.a. | + | n.a. | n.a. | - | + | **high** |
| Xiao et al. 2021 | + | + | + | ! | ! | n.a. | ! | ! | ! | ! | n.a. | n.a. | n.a. | ! | n.a. | n.a. | n.a. | n.a. | n.a. | + | n.a. | n.a. | ! | + | **high** |
| Bastos et al. 2015 | + | + | n.a. | n.a. | n.a. | n.a. | n.a. | n.a. | n.a. | n.a. | + | + | n.a. | n.a. | n.a. | n.a. | n.a. | n.a. | n.a. | + | + | n.a. | ! | + | **low** |
| Baoding Chen et al. 2019 | + | + | n.a. | n.a. | n.a. | n.a. | n.a. | n.a. | n.a. | n.a. | + | n.a. | n.a. | n.a. | n.a. | n.a. | n.a. | n.a. | n.a. | ! | n.a. | n.a. | ! | + | **moderate** |
| Jeon et al. 2017 | + | + | + | ! | ! | n.a. | n.a. | n.a. | ! | n.a. | n.a. | n.a. | n.a. | ! | n.a. | n.a. | n.a. | n.a. | n.a. | + | n.a. | n.a. | ! | + | **high** |
| Kim et al. 2005 | + | + | n.a. | n.a. | n.a. | n.a. | n.a. | n.a. | n.a. | n.a. | + | n.a. | n.a. | n.a. | n.a. | n.a. | n.a. | n.a. | n.a. | + | n.a. | n.a. | ! | + | **low** |
| Kim et al. 2012 | + | + | + | + | - | n.a. | - | n.a. | n.a. | n.a. | n.a. | n.a. | n.a. | n.a. | n.a. | n.a. | n.a. | n.a. | n.a. | + | n.a. | n.a. | ! | + | **high** |
| Kim et al. 2014 | + | + | n.a. | n.a. | - | n.a. | n.a. | n.a. | n.a. | n.a. | n.a. | n.a. | n.a. | n.a. | n.a. | n.a. | n.a. | n.a. | n.a. | ! | n.a. | n.a. | ! | + | **high** |
| Kwak et al. 2009 | + | + | n.a. | n.a. | n.a. | n.a. | n.a. | n.a. | n.a. | n.a. | + | n.a. | n.a. | n.a. | n.a. | n.a. | n.a. | n.a. | n.a. | + | + | n.a. | ! | + | **low** |
| Lee et al. 2008 | + | + | + | ! | ! | n.a. | + | n.a. | n.a. | n.a. | n.a. | n.a. | n.a. | n.a. | n.a. | n.a. | n.a. | n.a. | n.a. | ! | n.a. | n.a. | ! | + | **high** |
| Lim et al. 2007 | + | + | + | n.a. | ! | n.a. | n.a. | n.a. | n.a. | n.a. | n.a. | n.a. | n.a. | n.a. | n.a. | n.a. | n.a. | n.a. | n.a. | ! | + | n.a. | ! | + | **high** |
| Lim et al. 2009 | + | + | + | ! | ! | n.a. | + | n.a. | - | n.a. | n.a. | n.a. | - | n.a. | n.a. | n.a. | n.a. | n.a. | n.a. | + | n.a. | n.a. | - | + | **high** |
| Oh et al. 2017 | + | + | + | ! | ! | n.a. | + | n.a. | n.a. | - | n.a. | n.a. | n.a. | n.a. | n.a. | n.a. | n.a. | n.a. | n.a. | ! | n.a. | n.a. | ! | + | **high** |
| Park et al. 2014 | + | + | + | ! | ! | n.a. | + | n.a. | + | + | n.a. | n.a. | n.a. | ! | n.a. | n.a. | n.a. | n.a. | n.a. | + | n.a. | n.a. | ! | + | **high** |
| Jin et al. 2014 | + | + | n.a. | n.a. | ! | n.a. | ! | ! | ! | n.a. | n.a. | n.a. | n.a. | ! | n.a. | n.a. | n.a. | n.a. | n.a. | + | + | n.a. | ! | + | **high** |
| Roti et al. 2006 | + | + | + | ! | ! | n.a. | + | n.a. | n.a. | n.a. | n.a. | n.a. | n.a. | n.a. | n.a. | n.a. | n.a. | n.a. | n.a. | + | n.a. | n.a. | ! | + | **high** |
| Pisanu et al. 2009 | + | + | + | n.a. | n.a. | n.a. | + | n.a. | n.a. | n.a. | n.a. | n.a. | - | n.a. | n.a. | n.a. | n.a. | n.a. | n.a. | ! | n.a. | n.a. | - | + | **high** |
| Lee et al. 2011 | + | + | n.a. | n.a. | - | n.a. | n.a. | n.a. | n.a. | n.a. | n.a. | n.a. | n.a. | n.a. | n.a. | n.a. | n.a. | n.a. | n.a. | + | n.a. | n.a. | ! | + | **high** |
| Lombardi et al. 2010 | + | + | + | + | + | n.a. | + | + | n.a. | + | n.a. | n.a. | n.a. | n.a. | n.a. | n.a. | n.a. | + | n.a. | ! | + | n.a. | + | + | **low** |
| Kim et al. 2013 | + | + | + | + | ! | n.a. | + | + | + | + | n.a. | n.a. | n.a. | n.a. | n.a. | n.a. | n.a. | n.a. | n.a. | + | n.a. | n.a. | ! | + | **moderate** |
| Cho Rok Lee et al. 2014 | + | + | + | ! | - | n.a. | + | n.a. | n.a. | n.a. | n.a. | n.a. | + | n.a. | n.a. | n.a. | n.a. | n.a. | n.a. | + | n.a. | n.a. | ! | + | **high** |
| Lee et al. 2013 | + | + | n.a. | n.a. | ! | n.a. | n.a. | n.a. | n.a. | n.a. | n.a. | n.a. | - | n.a. | n.a. | n.a. | n.a. | n.a. | n.a. | ! | n.a. | n.a. | ! | + | **high** |
| Jeong et al. 2022 | + | + | + | + | + | n.a. | n.a. | n.a. | n.a. | n.a. | n.a. | n.a. | n.a. | ! | n.a. | n.a. | n.a. | n.a. | n.a. | n.a. | + | n.a. | ! | + | **low** |
| Cui Zhang et al. 2020 | + | + | + | ! | ! | n.a. | + | + | + | - | n.a. | n.a. | n.a. | ! | n.a. | n.a. | + | n.a. | n.a. | + | n.a. | n.a. | ! | + | **high** |
| Hui Huang II et al. 2023 | + | + | n.a. | n.a. | n.a. | n.a. | n.a. | n.a. | n.a. | + | n.a. | n.a. | n.a. | n.a. | n.a. | n.a. | n.a. | n.a. | n.a. | + | n.a. | n.a. | ! | + | **low** |
| Lee et al. 2009 | + | + | n.a. | n.a. | n.a. | n.a. | n.a. | n.a. | n.a. | n.a. | + | n.a. | n.a. | n.a. | n.a. | n.a. | n.a. | ! | n.a. | ! | + | n.a. | ! | + | **moderate** |
| Koo et al. 2010 | + | + | + | + | - | n.a. | - | n.a. | n.a. | n.a. | n.a. | n.a. | n.a. | n.a. | n.a. | n.a. | n.a. | n.a. | n.a. | ! | n.a. | n.a. | ! | + | **high** |
| Lin et al. 2010 | + | + | + | + | + | n.a. | + | n.a. | + | n.a. | + | n.a. | n.a. | n.a. | n.a. | n.a. | n.a. | n.a. | n.a. | + | + | n.a. | ! | + | **low** |
| Moon et al. 2011 | + | + | + | + | ! | n.a. | + | + | n.a. | n.a. | n.a. | n.a. | n.a. | n.a. | n.a. | n.a. | n.a. | n.a. | n.a. | n.a. | + | n.a. | - | + | **high** |
| Hyun et al. 2012 | + | + | + | + | + | n.a. | + | n.a. | n.a. | n.a. | n.a. | n.a. | n.a. | n.a. | n.a. | n.a. | n.a. | n.a. | n.a. | + | n.a. | n.a. | ! | + | **low** |
| Zhao et al. 2013 | + | + | + | ! | ! | n.a. | + | + | n.a. | + | n.a. | n.a. | + | n.a. | n.a. | n.a. | + | n.a. | n.a. | + | n.a. | n.a. | ! | + | **high** |
| Zheng et al. 2013 | + | + | n.a. | n.a. | ! | n.a. | n.a. | n.a. | n.a. | n.a. | n.a. | n.a. | n.a. | n.a. | n.a. | n.a. | n.a. | n.a. | n.a. | ! | n.a. | n.a. | + | + | **moderate** |
| Kim et al. 2016 | + | + | + | + | + | n.a. | + | + | n.a. | + | n.a. | n.a. | n.a. | n.a. | n.a. | n.a. | n.a. | n.a. | n.a. | + | n.a. | n.a. | ! | + | **low** |
| Yi Ho Lee et al. 2017 | + | + | + | n.a. | n.a. | n.a. | n.a. | n.a. | n.a. | n.a. | n.a. | n.a. | n.a. | n.a. | n.a. | n.a. | n.a. | n.a. | n.a. | + | + | n.a. | ! | + | **low** |
| Back et al. 2019 | + | + | + | + | + | n.a. | n.a. | n.a. | ! | + | + | n.a. | n.a. | n.a. | n.a. | n.a. | n.a. | n.a. | n.a. | + | n.a. | n.a. | ! | + | **moderate** |
| Shin et al. 2014 | + | + | + | + | + | n.a. | + | + | ! | n.a. | n.a. | n.a. | n.a. | n.a. | n.a. | n.a. | n.a. | n.a. | n.a. | + | n.a. | n.a. | - | + | **high** |
| Choi et al. 2015 | + | + | n.a. | n.a. | n.a. | + | n.a. | n.a. | n.a. | n.a. | n.a. | n.a. | n.a. | n.a. | n.a. | n.a. | n.a. | n.a. | n.a. | + | + | n.a. | ! | + | **low** |
| Pisanu et al. 2015 | + | + | n.a. | n.a. | ! | n.a. | + | n.a. | n.a. | n.a. | n.a. | n.a. | n.a. | n.a. | n.a. | n.a. | n.a. | n.a. | n.a. | ! | n.a. | n.a. | - | + | **high** |
| Kwangsoon Kim et al. 2020 | + | + | + | + | - | n.a. | - | n.a. | n.a. | n.a. | + | n.a. | - | n.a. | n.a. | n.a. | n.a. | n.a. | n.a. | ! | n.a. | n.a. | ! | + | **high** |
| Zuhur et al. 2024 | + | + | + | + | + | n.a. | + | + | n.a. | n.a. | n.a. | n.a. | n.a. | n.a. | n.a. | n.a. | n.a. | n.a. | n.a. | n.a. | + | n.a. | - | + | **moderate** |
| Jingjia Cao et al. 2024 | + | + | n.a. | n.a. | n.a. | n.a. | n.a. | n.a. | n.a. | n.a. | + | n.a. | n.a. | n.a. | n.a. | n.a. | n.a. | n.a. | n.a. | + | + | n.a. | ! | + | **low** |
| Kim et al. 2010 | + | + | n.a. | n.a. | n.a. | n.a. | n.a. | n.a. | n.a. | + | n.a. | n.a. | n.a. | n.a. | n.a. | n.a. | n.a. | n.a. | n.a. | + | + | n.a. | ! | + | **low** |
| Zheng et al. 2012 | + | + | + | + | + | n.a. | n.a. | n.a. | + | ! | n.a. | n.a. | n.a. | ! | n.a. | n.a. | n.a. | n.a. | n.a. | + | n.a. | n.a. | ! | + | **moderate** |
| Qu et al. 2016 | + | + | n.a. | n.a. | n.a. | n.a. | n.a. | n.a. | n.a. | + | n.a. | n.a. | n.a. | n.a. | n.a. | n.a. | n.a. | n.a. | n.a. | + | + | n.a. | ! | + | **low** |
| Gui et al. 2018 | + | + | + | ! | ! | n.a. | + | n.a. | n.a. | + | n.a. | n.a. | n.a. | ! | n.a. | n.a. | n.a. | n.a. | n.a. | + | n.a. | n.a. | ! | + | **high** |
| Ahn et al. 2014 | + | + | + | ! | ! | n.a. | + | n.a. | n.a. | + | n.a. | n.a. | n.a. | n.a. | n.a. | n.a. | n.a. | n.a. | n.a. | n.a. | + | n.a. | ! | + | **high** |
| Feng et al. 2020 | + | + | + | ! | ! | ! | + | n.a. | + | n.a. | + | n.a. | n.a. | n.a. | n.a. | n.a. | n.a. | n.a. | n.a. | + | n.a. | n.a. | ! | + | **high** |
| Lee et al. 2021 | + | + | n.a. | n.a. | n.a. | n.a. | n.a. | n.a. | n.a. | n.a. | + | + | n.a. | n.a. | n.a. | n.a. | n.a. | n.a. | n.a. | + | + | n.a. | ! | + | **low** |
| Saaduddin et al. 2016 | + | + | + | ! | + | n.a. | + | n.a. | n.a. | + | n.a. | n.a. | n.a. | n.a. | n.a. | n.a. | n.a. | n.a. | n.a. | ! | n.a. | n.a. | - | + | **high** |
| Varshney et al. 2014 | + | + | + | ! | n.a. | n.a. | + | + | n.a. | n.a. | n.a. | n.a. | n.a. | n.a. | n.a. | n.a. | n.a. | n.a. | n.a. | ! | n.a. | n.a. | - | + | **high** |
| Vasileiadis et al. 2011 | + | + | n.a. | n.a. | ! | n.a. | n.a. | n.a. | n.a. | n.a. | n.a. | n.a. | n.a. | n.a. | n.a. | n.a. | n.a. | n.a. | n.a. | + | n.a. | n.a. | - | + | **high** |
| Wang et al. 2016 | + | + | + | + | + | n.a. | + | n.a. | n.a. | n.a. | n.a. | n.a. | n.a. | ! | n.a. | n.a. | n.a. | n.a. | n.a. | + | n.a. | n.a. | ! | + | **moderate** |
| Wu et al. 2018 | + | + | + | + | ! | + | + | + | n.a. | ! | n.a. | n.a. | - | n.a. | n.a. | ! | n.a. | n.a. | n.a. | + | n.a. | n.a. | - | + | **high** |
| Xiang et al. 2014 | + | + | + | ! | ! | n.a. | + | n.a. | + | - | n.a. | n.a. | + | n.a. | n.a. | n.a. | + | n.a. | n.a. | + | n.a. | n.a. | ! | + | **high** |
| Xu et al. 2018 | + | + | + | ! | + | n.a. | + | n.a. | n.a. | n.a. | n.a. | n.a. | n.a. | n.a. | n.a. | n.a. | n.a. | n.a. | n.a. | + | n.a. | n.a. | ! | + | **moderate** |
| Yan et al. 2021 | + | + | n.a. | n.a. | n.a. | n.a. | ! | ! | n.a. | n.a. | n.a. | n.a. | n.a. | n.a. | n.a. | n.a. | n.a. | n.a. | n.a. | + | n.a. | n.a. | ! | + | **high** |
| Yu et al. 2017 | + | + | + | ! | ! | n.a. | + | + | n.a. | n.a. | n.a. | n.a. | + | n.a. | n.a. | n.a. | n.a. | n.a. | n.a. | + | n.a. | n.a. | ! | + | **high** |
| Yuan et al. 2017 | + | + | + | ! | + | n.a. | + | + | n.a. | n.a. | n.a. | n.a. | - | n.a. | n.a. | n.a. | n.a. | n.a. | n.a. | + | n.a. | n.a. | - | + | **high** |
| Zeng et al. 2013 | + | + | + | ! | ! | n.a. | + | n.a. | n.a. | + | n.a. | n.a. | n.a. | n.a. | n.a. | n.a. | n.a. | n.a. | n.a. | + | n.a. | n.a. | ! | + | **high** |
| Zhang et al. 2013 | + | + | + | + | + | n.a. | + | + | + | n.a. | n.a. | n.a. | n.a. | n.a. | n.a. | n.a. | n.a. | n.a. | n.a. | + | n.a. | n.a. | ! | + | **low** |
| Zheng et al. 2019 | + | + | + | + | + | n.a. | + | + | n.a. | - | n.a. | n.a. | n.a. | n.a. | n.a. | n.a. | + | + | n.a. | + | n.a. | n.a. | ! | + | **high** |
| Zhao et al. 2017 | + | + | + | ! | ! | n.a. | ! | n.a. | n.a. | ! | n.a. | n.a. | n.a. | ! | n.a. | n.a. | ! | n.a. | n.a. | + | n.a. | n.a. | ! | + | **high** |
| Gao et al. 2025 | + | + | + | ! | ! | n.a. | + | n.a. | n.a. | n.a. | n.a. | n.a. | n.a. | ! | n.a. | n.a. | n.a. | n.a. | n.a. | + | n.a. | n.a. | ! | + | **high** |
| Ozemir et al. 2023 | + | + | n.a. | n.a. | n.a. | n.a. | + | n.a. | n.a. | n.a. | n.a. | n.a. | n.a. | n.a. | n.a. | n.a. | n.a. | n.a. | n.a. | ! | + | n.a. | ! | + | **moderate** |
| Goran et al. 2017 | + | + | n.a. | n.a. | ! | n.a. | + | + | n.a. | n.a. | n.a. | n.a. | - | n.a. | n.a. | n.a. | n.a. | n.a. | n.a. | + | n.a. | n.a. | ! | + | **high** |
| Goran et al. 2019 | + | + | + | n.a. | ! | n.a. | + | + | n.a. | n.a. | n.a. | n.a. | - | n.a. | n.a. | n.a. | n.a. | n.a. | n.a. | + | n.a. | n.a. | ! | + | **high** |
| Meilinger-Dobra et al. 2018 | + | + | + | n.a. | n.a. | n.a. | + | n.a. | n.a. | n.a. | n.a. | n.a. | n.a. | n.a. | n.a. | n.a. | n.a. | n.a. | n.a. | ! | n.a. | n.a. | - | + | **high** |
| Zhao et al. 2019 | + | + | + | ! | ! | n.a. | + | n.a. | ! | - | n.a. | n.a. | n.a. | n.a. | n.a. | n.a. | + | n.a. | n.a. | + | n.a. | n.a. | ! | + | **high** |
| Zhou et al. 2012 | + | + | + | + | + | n.a. | + | - | n.a. | + | n.a. | n.a. | n.a. | n.a. | n.a. | n.a. | n.a. | n.a. | n.a. | + | n.a. | n.a. | ! | + | **high** |
| Choi et al. 2013 | + | + | n.a. | n.a. | n.a. | n.a. | n.a. | n.a. | n.a. | n.a. | + | n.a. | n.a. | n.a. | n.a. | n.a. | n.a. | n.a. | n.a. | + | + | n.a. | ! | + | **low** |
| Seifert et al. 2021 | + | + | + | + | + | n.a. | + | n.a. | n.a. | n.a. | n.a. | n.a. | n.a. | n.a. | n.a. | n.a. | n.a. | n.a. | n.a. | ! | n.a. | n.a. | ! | + | **moderate** |
| Kayhan et al. 2024 | + | + | + | ! | + | n.a. | + | n.a. | n.a. | n.a. | n.a. | n.a. | + | n.a. | n.a. | n.a. | n.a. | n.a. | n.a. | ! | n.a. | n.a. | ! | + | **high** |
| Zhang Zhu et al. 2024 | + | + | + | + | + | n.a. | ! | ! | ! | ! | n.a. | n.a. | ! | ! | n.a. | n.a. | n.a. | n.a. | n.a. | + | n.a. | n.a. | ! | + | **high** |
| Zhi Zhao et al. 2018 | + | + | + | ! | ! | n.a. | + | n.a. | n.a. | + | n.a. | n.a. | n.a. | n.a. | n.a. | n.a. | n.a. | n.a. | n.a. | + | n.a. | n.a. | ! | + | **high** |
| Wada et al. 2003 | + | + | n.a. | n.a. | n.a. | n.a. | + | + | + | n.a. | n.a. | n.a. | n.a. | n.a. | n.a. | n.a. | n.a. | n.a. | n.a. | + | n.a. | n.a. | ! | + | **moderate** |
| Chen et al. 2019 | + | + | + | ! | ! | n.a. | + | + | n.a. | + | + | n.a. | n.a. | ! | n.a. | n.a. | n.a. | n.a. | n.a. | + | n.a. | n.a. | ! | + | **high** |
| Lu et al. 2020 | + | + | + | ! | + | n.a. | + | n.a. | n.a. | n.a. | n.a. | n.a. | n.a. | n.a. | n.a. | n.a. | n.a. | n.a. | n.a. | + | n.a. | n.a. | + | + | **low** |
| Oh et al. 2013 | + | + | n.a. | + | + | n.a. | + | n.a. | n.a. | n.a. | n.a. | n.a. | n.a. | ! | n.a. | n.a. | n.a. | n.a. | n.a. | + | n.a. | n.a. | - | + | **high** |
| Yu et al. 2022 | + | + | + | + | + | n.a. | + | n.a. | n.a. | + | + | n.a. | n.a. | ! | n.a. | n.a. | n.a. | n.a. | n.a. | + | n.a. | n.a. | ! | + | **moderate** |
| Xiaojuan Zheng et al. 2020 | + | + | + | ! | n.a. | n.a. | n.a. | n.a. | n.a. | n.a. | n.a. | n.a. | n.a. | n.a. | n.a. | n.a. | n.a. | n.a. | n.a. | ! | n.a. | n.a. | - | + | **high** |
| Chow et al. 2003 | + | + | n.a. | ! | n.a. | n.a. | n.a. | n.a. | n.a. | n.a. | n.a. | n.a. | n.a. | n.a. | n.a. | n.a. | n.a. | n.a. | n.a. | ! | + | n.a. | ! | + | **high** |
| Rossi et al. 2012 | + | + | n.a. | n.a. | n.a. | n.a. | n.a. | + | n.a. | n.a. | n.a. | n.a. | n.a. | n.a. | n.a. | n.a. | n.a. | n.a. | n.a. | ! | + | n.a. | ! | + | **moderate** |
| Tagliabue et al. 2021 | + | + | + | + | n.a. | n.a. | + | n.a. | n.a. | n.a. | n.a. | n.a. | n.a. | n.a. | n.a. | n.a. | n.a. | n.a. | n.a. | + | n.a. | n.a. | - | + | **moderate** |
| Xiangqian Zheng et al. 2019 | + | + | + | + | + | n.a. | + | + | + | + | + | n.a. | n.a. | n.a. | n.a. | n.a. | n.a. | n.a. | n.a. | + | + | n.a. | ! | + | **low** |
| Wei et al. 2019 | + | + | n.a. | n.a. | ! | n.a. | n.a. | n.a. | ! | n.a. | n.a. | n.a. | n.a. | n.a. | n.a. | n.a. | n.a. | n.a. | n.a. | ! | n.a. | n.a. | - | + | **high** |
| Lee et al. 2017 | + | + | n.a. | n.a. | n.a. | n.a. | n.a. | n.a. | n.a. | n.a. | + | n.a. | n.a. | n.a. | n.a. | n.a. | n.a. | n.a. | n.a. | + | + | n.a. | ! | + | **low** |
| Seo et al. 2016 | + | + | n.a. | n.a. | ! | n.a. | n.a. | n.a. | n.a. | n.a. | n.a. | n.a. | n.a. | n.a. | n.a. | n.a. | n.a. | n.a. | n.a. | + | + | n.a. | ! | + | **moderate** |
| Song Yan et al. 2020 | + | + | + | ! | ! | n.a. | + | + | + | ! | n.a. | n.a. | n.a. | n.a. | n.a. | n.a. | n.a. | n.a. | n.a. | + | n.a. | n.a. | - | + | **high** |
| Yang et al. 2014 | + | + | + | ! | ! | n.a. | + | + | n.a. | + | + | n.a. | n.a. | n.a. | n.a. | n.a. | n.a. | n.a. | n.a. | + | n.a. | n.a. | ! | + | **high** |
| Ren et al. 2018 | + | + | n.a. | n.a. | n.a. | n.a. | n.a. | n.a. | n.a. | n.a. | + | n.a. | n.a. | n.a. | n.a. | n.a. | n.a. | n.a. | n.a. | + | + | n.a. | ! | + | **low** |
| Zheng et al. 2018 | + | + | + | ! | ! | n.a. | - | - | n.a. | - | n.a. | n.a. | n.a. | n.a. | n.a. | n.a. | - | n.a. | n.a. | + | n.a. | n.a. | ! | + | **high** |
| Takahito et al. 2024 | + | + | n.a. | + | n.a. | n.a. | + | n.a. | n.a. | n.a. | n.a. | n.a. | n.a. | n.a. | n.a. | n.a. | n.a. | n.a. | n.a. | n.a. | + | n.a. | - | + | **moderate** |
| Chen et al. 2023 | + | + | + | + | + | n.a. | n.a. | n.a. | + | + | n.a. | n.a. | n.a. | ! | n.a. | n.a. | + | n.a. | n.a. | + | n.a. | n.a. | ! | + | **moderate** |
| Kim et al. 2021 | + | + | + | + | + | n.a. | + | n.a. | n.a. | n.a. | n.a. | n.a. | n.a. | n.a. | n.a. | n.a. | n.a. | n.a. | n.a. | + | n.a. | n.a. | ! | + | **low** |
| Lim et al. 2024 | + | + | + | + | + | n.a. | + | + | n.a. | ! | + | n.a. | n.a. | n.a. | n.a. | n.a. | n.a. | n.a. | n.a. | + | n.a. | n.a. | + | + | **low** |
| Xue et al. 2019 | + | + | + | + | + | + | + | + | + | + | n.a. | n.a. | n.a. | n.a. | n.a. | n.a. | n.a. | n.a. | n.a. | + | n.a. | n.a. | + | + | **low** |
| Sun et al. 2021 | + | + | + | ! | ! | n.a. | ! | ! | ! | ! | n.a. | n.a. | n.a. | ! | ! | ! | n.a. | n.a. | n.a. | + | n.a. | n.a. | ! | + | **high** |
| Chunwang et al. 2021 | + | + | + | + | + | n.a. | ! | ! | n.a. | n.a. | n.a. | n.a. | n.a. | n.a. | n.a. | n.a. | n.a. | n.a. | n.a. | + | n.a. | n.a. | ! | + | **high** |
| Ma et al. 2023 | + | + | + | + | n.a. | n.a. | + | + | n.a. | + | + | + | n.a. | n.a. | n.a. | n.a. | n.a. | n.a. | n.a. | + | n.a. | n.a. | + | + | **low** |
| Li et al. 2023 | + | + | + | ! | n.a. | n.a. | n.a. | n.a. | n.a. | n.a. | n.a. | n.a. | - | n.a. | n.a. | n.a. | n.a. | n.a. | n.a. | ! | n.a. | n.a. | ! | + | **high** |
| Zhang et al. 2023 | + | + | + | ! | n.a. | n.a. | n.a. | n.a. | ! | n.a. | n.a. | n.a. | n.a. | n.a. | n.a. | n.a. | n.a. | n.a. | n.a. | + | n.a. | n.a. | ! | + | **high** |
| Huang et al. 2024 | + | + | + | + | + | ! | + | n.a. | ! | + | n.a. | n.a. | n.a. | n.a. | n.a. | n.a. | n.a. | n.a. | n.a. | + | n.a. | n.a. | ! | + | **high** |
| Lai et al. 2016 | + | + | + | ! | ! | n.a. | ! | ! | n.a. | - | n.a. | n.a. | n.a. | n.a. | n.a. | n.a. | n.a. | n.a. | n.a. | + | n.a. | n.a. | ! | + | **high** |
| Kim et al. 2017 | + | + | + | + | ! | n.a. | n.a. | n.a. | ! | n.a. | n.a. | n.a. | n.a. | n.a. | n.a. | n.a. | n.a. | n.a. | n.a. | ! | n.a. | n.a. | ! | + | **high** |
| Wenlong Wan et al. 2020 | + | + | + | + | + | n.a. | + | + | n.a. | - | n.a. | n.a. | - | n.a. | n.a. | n.a. | n.a. | n.a. | n.a. | ! | n.a. | n.a. | - | + | **high** |
| Cao et al. 2022 | + | + | + | + | ! | + | + | + | n.a. | + | n.a. | n.a. | + | n.a. | n.a. | n.a. | n.a. | n.a. | n.a. | ! | n.a. | n.a. | ! | + | **high** |
| Zhu et al. 2022 | + | + | n.a. | n.a. | ! | n.a. | n.a. | n.a. | n.a. | n.a. | n.a. | n.a. | n.a. | n.a. | n.a. | n.a. | n.a. | n.a. | n.a. | + | + | n.a. | ! | + | **moderate** |
| Wu et al. 2020 | + | + | + | ! | + | + | + | n.a. | n.a. | - | n.a. | n.a. | - | n.a. | n.a. | n.a. | n.a. | n.a. | n.a. | + | n.a. | n.a. | - | + | **high** |
| Wang et al. 2020 | + | + | + | + | ! | n.a. | + | + | n.a. | - | n.a. | n.a. | - | n.a. | n.a. | n.a. | n.a. | n.a. | n.a. | + | n.a. | n.a. | ! | + | **high** |
| Zhao et al. 2023 | + | + | + | + | n.a. | + | n.a. | n.a. | n.a. | n.a. | n.a. | n.a. | n.a. | n.a. | ! | ! | n.a. | n.a. | n.a. | + | n.a. | n.a. | + | + | **low** |
| Karatzas et al. 2013 | + | + | n.a. | n.a. | n.a. | n.a. | n.a. | + | n.a. | n.a. | n.a. | n.a. | n.a. | n.a. | n.a. | n.a. | n.a. | n.a. | n.a. | ! | n.a. | n.a. | ! | + | **moderate** |
| Zeming et al. 2015 | + | + | n.a. | n.a. | n.a. | + | n.a. | n.a. | n.a. | n.a. | n.a. | n.a. | n.a. | n.a. | n.a. | n.a. | n.a. | n.a. | n.a. | + | + | n.a. | + | + | **low** |
| Dequan Xu et al. 2014 | + | + | + | ! | + | n.a. | + | + | + | - | n.a. | n.a. | - | n.a. | n.a. | n.a. | n.a. | n.a. | n.a. | + | n.a. | n.a. | ! | + | **high** |
| He et al. 2017 | + | + | + | ! | ! | n.a. | + | + | n.a. | + | n.a. | n.a. | n.a. | n.a. | n.a. | n.a. | n.a. | n.a. | n.a. | + | n.a. | n.a. | ! | + | **high** |
| Ji et al. 2019 | + | + | + | ! | n.a. | n.a. | n.a. | n.a. | n.a. | n.a. | + | n.a. | - | n.a. | n.a. | n.a. | n.a. | n.a. | n.a. | + | n.a. | n.a. | ! | + | **high** |
| Liu Wang et al. 2021 | + | + | + | ! | ! | n.a. | + | n.a. | n.a. | + | n.a. | n.a. | n.a. | ! | n.a. | n.a. | n.a. | n.a. | n.a. | + | n.a. | n.a. | + | + | **high** |
| Lai et al. 2023 | + | + | n.a. | n.a. | n.a. | n.a. | n.a. | n.a. | n.a. | n.a. | + | n.a. | n.a. | n.a. | n.a. | n.a. | n.a. | n.a. | n.a. | ! | n.a. | n.a. | ! | + | **high** |
| Hitu et al. 2021 | + | + | + | + | + | n.a. | + | + | n.a. | n.a. | n.a. | n.a. | + | n.a. | n.a. | n.a. | n.a. | n.a. | n.a. | ! | n.a. | n.a. | ! | + | **moderate** |
| Parvathareddy et al. 2022 | + | + | + | ! | ! | n.a. | + | + | n.a. | - | + | + | n.a. | n.a. | n.a. | n.a. | n.a. | n.a. | n.a. | ! | n.a. | n.a. | - | + | **high** |
| Yang et al. 2022 | + | + | + | n.a. | n.a. | n.a. | + | n.a. | n.a. | - | n.a. | n.a. | n.a. | n.a. | n.a. | n.a. | + | n.a. | n.a. | + | n.a. | n.a. | ! | + | **high** |
| Cho et al. 2012 | + | + | n.a. | ! | n.a. | n.a. | n.a. | n.a. | n.a. | n.a. | n.a. | n.a. | n.a. | n.a. | n.a. | n.a. | n.a. | n.a. | n.a. | + | n.a. | n.a. | ! | + | **moderate** |
| Kemin et al. 2021 | + | + | n.a. | n.a. | n.a. | n.a. | n.a. | n.a. | + | n.a. | n.a. | n.a. | ! | ! | n.a. | n.a. | n.a. | n.a. | n.a. | + | n.a. | n.a. | - | + | **high** |
| Sun et al. 2024 | + | + | + | ! | ! | n.a. | + | n.a. | n.a. | - | + | n.a. | n.a. | ! | n.a. | n.a. | n.a. | n.a. | n.a. | + | n.a. | n.a. | - | + | **high** |
| Jiwang et al. 2022 | + | + | + | ! | - | n.a. | ! | ! | ! | - | n.a. | n.a. | n.a. | n.a. | n.a. | n.a. | n.a. | n.a. | n.a. | + | n.a. | n.a. | ! | + | **high** |
| Tao et al. 2016 | + | + | + | ! | ! | n.a. | + | n.a. | n.a. | n.a. | n.a. | n.a. | n.a. | n.a. | n.a. | n.a. | n.a. | n.a. | n.a. | + | n.a. | n.a. | - | + | **high** |
| Park et al. 2015 | + | + | n.a. | n.a. | n.a. | n.a. | n.a. | + | n.a. | n.a. | n.a. | n.a. | n.a. | n.a. | n.a. | n.a. | n.a. | n.a. | n.a. | + | + | n.a. | + | + | **low** |
| Xu et al. 2016 | + | + | + | + | ! | n.a. | ! | ! | ! | n.a. | n.a. | n.a. | ! | ! | n.a. | n.a. | n.a. | n.a. | n.a. | + | n.a. | n.a. | - | + | **high** |
| Zheng Liu et al. 2017 | + | + | + | ! | ! | + | + | + | + | ! | n.a. | n.a. | ! | ! | ! | n.a. | + | + | n.a. | + | n.a. | n.a. | + | + | **high** |
| Wu et al. 2019 | + | + | + | ! | + | + | + | + | n.a. | - | n.a. | n.a. | n.a. | n.a. | ! | n.a. | n.a. | n.a. | n.a. | + | n.a. | n.a. | ! | + | **high** |
| Wang et al. 2018 | + | + | + | + | + | n.a. | + | + | n.a. | - | n.a. | n.a. | n.a. | n.a. | n.a. | n.a. | + | - | n.a. | + | n.a. | n.a. | - | + | **high** |
| Sezer et al. 2020 | + | + | n.a. | n.a. | n.a. | n.a. | n.a. | n.a. | n.a. | n.a. | + | n.a. | n.a. | n.a. | n.a. | n.a. | n.a. | n.a. | n.a. | ! | + | n.a. | - | + | **high** |
| Lu et al. 2017 | + | + | n.a. | n.a. | n.a. | n.a. | n.a. | n.a. | n.a. | n.a. | + | n.a. | n.a. | n.a. | n.a. | n.a. | n.a. | n.a. | n.a. | + | + | n.a. | + | + | **low** |
| Zhou et al. 2019 | + | + | n.a. | n.a. | n.a. | n.a. | n.a. | n.a. | n.a. | n.a. | + | n.a. | n.a. | n.a. | n.a. | n.a. | n.a. | n.a. | n.a. | + | n.a. | n.a. | - | + | **moderate** |
| Dong et al. 2021 | + | + | n.a. | n.a. | n.a. | n.a. | n.a. | n.a. | n.a. | n.a. | + | n.a. | n.a. | n.a. | n.a. | n.a. | n.a. | n.a. | n.a. | ! | n.a. | n.a. | ! | + | **moderate** |
| Wang et al. 2024 | + | + | + | + | + | n.a. | n.a. | n.a. | n.a. | - | n.a. | n.a. | - | n.a. | n.a. | n.a. | n.a. | n.a. | n.a. | + | n.a. | n.a. | ! | + | **high** |
| Xue et al. 2024 | + | + | + | ! | n.a. | n.a. | n.a. | n.a. | n.a. | n.a. | n.a. | n.a. | n.a. | n.a. | n.a. | n.a. | n.a. | n.a. | n.a. | + | n.a. | n.a. | - | + | **high** |
| Virk et al. 2013 | + | + | n.a. | n.a. | n.a. | n.a. | n.a. | n.a. | n.a. | n.a. | + | n.a. | n.a. | n.a. | n.a. | n.a. | n.a. | n.a. | n.a. | ! | + | n.a. | - | + | **high** |
| Shen et al. 2022 | + | + | + | ! | ! | n.a. | + | + | n.a. | + | n.a. | n.a. | n.a. | n.a. | n.a. | n.a. | n.a. | n.a. | n.a. | + | n.a. | n.a. | - | + | **high** |
| Jin et al. 2018 | + | + | + | ! | ! | n.a. | n.a. | n.a. | n.a. | - | + | n.a. | - | n.a. | n.a. | n.a. | n.a. | n.a. | n.a. | ! | n.a. | n.a. | ! | + | **high** |
| Zhang et al. 2015 | + | + | + | ! | ! | n.a. | + | + | n.a. | + | n.a. | n.a. | n.a. | n.a. | n.a. | n.a. | n.a. | n.a. | n.a. | + | n.a. | n.a. | ! | + | **high** |
| Ayesha et al. 2023 | + | + | n.a. | ! | ! | n.a. | + | n.a. | n.a. | + | + | n.a. | n.a. | n.a. | n.a. | n.a. | n.a. | n.a. | n.a. | ! | n.a. | n.a. | ! | + | **high** |
| Bradley et al. 2017 | + | + | n.a. | n.a. | ! | n.a. | n.a. | n.a. | n.a. | n.a. | n.a. | n.a. | n.a. | n.a. | n.a. | n.a. | n.a. | n.a. | n.a. | ! | + | n.a. | ! | + | **high** |
| Huang et al. 2023 | + | + | + | ! | ! | n.a. | + | n.a. | + | + | n.a. | n.a. | n.a. | n.a. | n.a. | n.a. | n.a. | n.a. | n.a. | + | n.a. | n.a. | ! | + | **high** |
| Qiu et al. 2024 | + | + | + | + | + | n.a. | + | n.a. | + | n.a. | n.a. | n.a. | n.a. | ! | n.a. | n.a. | n.a. | n.a. | n.a. | + | n.a. | n.a. | + | + | **low** |
| Gu et al. 2019 | + | + | + | + | + | n.a. | n.a. | n.a. | + | - | n.a. | n.a. | - | n.a. | n.a. | n.a. | n.a. | n.a. | n.a. | ! | n.a. | n.a. | ! | + | **high** |
| Xie et al. 2023 | + | + | + | ! | ! | n.a. | + | + | n.a. | n.a. | + | n.a. | - | n.a. | n.a. | n.a. | n.a. | n.a. | n.a. | + | n.a. | n.a. | + | + | **high** |
| Yin et al. 2017 | + | + | + | ! | ! | n.a. | n.a. | n.a. | n.a. | + | n.a. | n.a. | n.a. | n.a. | n.a. | n.a. | n.a. | n.a. | n.a. | + | n.a. | n.a. | ! | + | **high** |
| Song et al. 2022 | + | + | + | + | n.a. | n.a. | n.a. | + | n.a. | n.a. | n.a. | n.a. | n.a. | n.a. | n.a. | n.a. | n.a. | n.a. | n.a. | ! | + | n.a. | ! | + | **high** |
| Cao et al. 2024 | + | + | + | + | ! | n.a. | + | n.a. | n.a. | + | n.a. | n.a. | + | n.a. | n.a. | n.a. | n.a. | n.a. | n.a. | ! | n.a. | n.a. | ! | + | **high** |
| Hong et al. 2015 | + | + | + | ! | - | n.a. | - | n.a. | n.a. | - | n.a. | n.a. | n.a. | ! | n.a. | n.a. | n.a. | n.a. | n.a. | + | n.a. | n.a. | ! | + | **high** |
| Bo Yeon Kim et al. 2012 | + | + | + | ! | - | n.a. | - | - | n.a. | - | n.a. | n.a. | n.a. | n.a. | n.a. | n.a. | n.a. | n.a. | n.a. | + | + | n.a. | ! | + | **high** |
| Pan et al. 2018 | + | + | + | ! | ! | n.a. | + | n.a. | + | n.a. | n.a. | n.a. | - | n.a. | n.a. | n.a. | n.a. | n.a. | n.a. | + | n.a. | n.a. | - | + | **high** |
| Zhang et al. 2022 | + | + | + | ! | ! | n.a. | - | n.a. | n.a. | - | n.a. | n.a. | n.a. | ! | n.a. | n.a. | n.a. | n.a. | n.a. | ! | n.a. | n.a. | ! | + | **high** |
| Besic et al. 2008 | + | + | n.a. | ! | ! | n.a. | + | n.a. | n.a. | n.a. | n.a. | n.a. | n.a. | n.a. | n.a. | n.a. | n.a. | n.a. | n.a. | ! | n.a. | n.a. | ! | + | **high** |
| Li Xiaojing et al. 2017 | + | + | + | ! | - | n.a. | - | - | n.a. | n.a. | n.a. | n.a. | - | n.a. | n.a. | n.a. | n.a. | n.a. | n.a. | + | n.a. | n.a. | ! | + | **high** |
| Xu et al. 2014 | + | + | + | ! | ! | n.a. | + | + | n.a. | n.a. | n.a. | n.a. | - | n.a. | n.a. | n.a. | n.a. | n.a. | n.a. | + | n.a. | n.a. | ! | + | **high** |
| Xia et al. 2011 | + | + | + | + | + | n.a. | + | n.a. | - | - | n.a. | n.a. | n.a. | n.a. | n.a. | n.a. | n.a. | n.a. | n.a. | + | n.a. | n.a. | - | + | **high** |
| Ma et al. 2021 | + | + | n.a. | n.a. | n.a. | n.a. | n.a. | n.a. | n.a. | n.a. | + | n.a. | n.a. | n.a. | n.a. | n.a. | n.a. | n.a. | n.a. | + | n.a. | n.a. | - | + | **moderate** |
| Korkmaz et al. 2016 | + | + | n.a. | n.a. | n.a. | n.a. | - | - | n.a. | n.a. | n.a. | n.a. | n.a. | n.a. | n.a. | n.a. | n.a. | n.a. | n.a. | ! | n.a. | n.a. | ! | + | **high** |
| Chang Liu et al. 2021 | + | + | + | ! | ! | n.a. | + | n.a. | n.a. | n.a. | n.a. | n.a. | n.a. | n.a. | n.a. | n.a. | n.a. | n.a. | n.a. | + | n.a. | n.a. | + | + | **moderate** |
| Pardo et al. 2020 | + | + | n.a. | n.a. | n.a. | n.a. | + | n.a. | n.a. | n.a. | n.a. | n.a. | n.a. | n.a. | n.a. | n.a. | n.a. | n.a. | n.a. | ! | n.a. | n.a. | - | + | **high** |
| Bian et al. 2015 | + | + | + | ! | ! | n.a. | + | + | n.a. | n.a. | n.a. | n.a. | n.a. | n.a. | n.a. | n.a. | n.a. | n.a. | n.a. | + | n.a. | n.a. | ! | + | **high** |
| Chen et al. 2016 | + | + | + | ! | ! | n.a. | + | n.a. | n.a. | + | n.a. | n.a. | n.a. | n.a. | n.a. | n.a. | n.a. | n.a. | n.a. | + | n.a. | n.a. | ! | + | **high** |
| Jiang et al. 2018 | + | + | + | ! | ! | n.a. | ! | + | n.a. | n.a. | n.a. | n.a. | n.a. | n.a. | n.a. | n.a. | n.a. | n.a. | n.a. | + | n.a. | n.a. | ! | + | **high** |
| Yu et al. 2018 | + | + | + | ! | n.a. | n.a. | + | + | n.a. | + | n.a. | n.a. | n.a. | n.a. | n.a. | n.a. | n.a. | n.a. | n.a. | + | n.a. | n.a. | ! | + | **moderate** |
| Zhang Haidong et al. 2023 | + | + | + | ! | n.a. | n.a. | + | n.a. | n.a. | + | n.a. | n.a. | n.a. | n.a. | n.a. | n.a. | n.a. | n.a. | n.a. | + | n.a. | n.a. | - | + | **high** |
| Wang Zhaohui et al. 2019 | + | + | + | ! | ! | n.a. | + | n.a. | n.a. | - | n.a. | n.a. | n.a. | n.a. | n.a. | n.a. | n.a. | n.a. | n.a. | + | n.a. | n.a. | - | + | **high** |
| Wu et al. 2017 | + | + | + | ! | ! | n.a. | + | + | n.a. | - | n.a. | n.a. | n.a. | n.a. | n.a. | n.a. | n.a. | n.a. | n.a. | + | n.a. | n.a. | ! | + | **high** |
| Wang et al. 2022 | + | + | + | ! | ! | n.a. | n.a. | n.a. | n.a. | n.a. | + | n.a. | n.a. | n.a. | n.a. | n.a. | n.a. | n.a. | n.a. | + | n.a. | n.a. | - | + | **high** |
| Wang et al. 2019 | + | + | n.a. | n.a. | ! | n.a. | + | n.a. | + | n.a. | n.a. | n.a. | n.a. | n.a. | n.a. | n.a. | n.a. | n.a. | n.a. | + | n.a. | n.a. | ! | + | **moderate** |
| Tang et al. 2024 | + | + | + | ! | n.a. | n.a. | n.a. | n.a. | n.a. | - | n.a. | n.a. | n.a. | n.a. | n.a. | n.a. | n.a. | n.a. | n.a. | ! | n.a. | n.a. | - | + | **high** |
| Mei et al. 2023 | + | + | + | ! | ! | n.a. | n.a. | n.a. | + | + | + | n.a. | n.a. | n.a. | n.a. | n.a. | n.a. | n.a. | n.a. | + | n.a. | n.a. | ! | + | **high** |
| Akgun et al. 2023 | + | + | n.a. | n.a. | ! | n.a. | + | n.a. | n.a. | n.a. | n.a. | n.a. | n.a. | n.a. | n.a. | n.a. | n.a. | n.a. | n.a. | + | + | n.a. | - | + | **high** |
| Yan et al. 2022 | + | + | n.a. | ! | n.a. | n.a. | n.a. | n.a. | n.a. | n.a. | n.a. | n.a. | n.a. | n.a. | n.a. | n.a. | n.a. | n.a. | n.a. | + | n.a. | n.a. | ! | + | **moderate** |
| Caliskan et al. 2012 | + | + | + | ! | ! | n.a. | + | + | n.a. | n.a. | n.a. | n.a. | n.a. | n.a. | n.a. | n.a. | n.a. | n.a. | n.a. | + | n.a. | n.a. | ! | + | **high** |
| Gweon et al. 2017 | + | + | + | ! | n.a. | n.a. | n.a. | n.a. | n.a. | n.a. | n.a. | n.a. | n.a. | n.a. | n.a. | n.a. | n.a. | n.a. | n.a. | + | + | n.a. | ! | + | **moderate** |
| Hassan et al. 2023 | + | + | + | ! | ! | n.a. | n.a. | n.a. | n.a. | n.a. | n.a. | n.a. | n.a. | n.a. | n.a. | n.a. | n.a. | n.a. | n.a. | + | n.a. | n.a. | ! | + | **high** |
| Hui Huang et al. 2023 | + | + | + | ! | ! | n.a. | + | + | + | + | n.a. | n.a. | n.a. | n.a. | n.a. | n.a. | n.a. | n.a. | n.a. | + | n.a. | n.a. | ! | + | **high** |
| Yoon et al. 2024 | + | + | + | n.a. | n.a. | n.a. | + | + | + | - | n.a. | n.a. | n.a. | n.a. | n.a. | n.a. | n.a. | n.a. | n.a. | + | n.a. | n.a. | ! | + | **high** |
| Jinqiu Wang et al. 2022 | + | + | + | ! | ! | n.a. | + | n.a. | n.a. | - | n.a. | n.a. | n.a. | n.a. | n.a. | n.a. | n.a. | n.a. | n.a. | + | n.a. | n.a. | ! | + | **high** |
| Jinqiu Wang et al. 2025 | + | + | + | ! | ! | n.a. | + | n.a. | n.a. | + | n.a. | n.a. | n.a. | n.a. | n.a. | n.a. | n.a. | n.a. | n.a. | + | n.a. | n.a. | ! | + | **high** |
| Kaliszewski et al. 2019 | + | + | n.a. | n.a. | ! | n.a. | + | + | n.a. | n.a. | n.a. | n.a. | n.a. | n.a. | n.a. | n.a. | n.a. | n.a. | n.a. | ! | n.a. | n.a. | ! | + | **high** |
| Yong Wan Kim et al. 2009 | + | + | + | ! | ! | n.a. | + | + | n.a. | n.a. | n.a. | n.a. | n.a. | n.a. | n.a. | n.a. | n.a. | n.a. | n.a. | + | n.a. | n.a. | ! | + | **high** |
| Won Jin Kim et al. 2013 | + | + | n.a. | ! | n.a. | n.a. | n.a. | n.a. | n.a. | n.a. | n.a. | n.a. | n.a. | n.a. | n.a. | n.a. | n.a. | n.a. | n.a. | + | + | n.a. | ! | + | **moderate** |
| Ju Yeon Kim et al. 2015 | + | + | + | ! | n.a. | n.a. | + | n.a. | n.a. | - | n.a. | n.a. | n.a. | n.a. | n.a. | n.a. | n.a. | n.a. | n.a. | n.a. | + | n.a. | ! | + | **high** |
| Young Hun Kim et a 2016 | + | + | + | ! | ! | n.a. | + | + | n.a. | n.a. | n.a. | n.a. | n.a. | n.a. | n.a. | n.a. | n.a. | n.a. | n.a. | + | n.a. | n.a. | ! | + | **high** |
| Hyeung Kyoo Kim et al. 2025 | + | + | + | ! | n.a. | n.a. | + | + | + | - | n.a. | n.a. | n.a. | n.a. | n.a. | n.a. | n.a. | n.a. | n.a. | + | n.a. | n.a. | ! | + | **high** |
| Jae Bok Lee et al. 2008 | + | + | + | ! | ! | n.a. | + | n.a. | n.a. | n.a. | n.a. | n.a. | n.a. | n.a. | n.a. | n.a. | n.a. | n.a. | n.a. | n.a. | + | n.a. | ! | + | **high** |
| Sung Min Lee et al. 2019 | + | + | n.a. | n.a. | n.a. | n.a. | n.a. | n.a. | n.a. | n.a. | + | n.a. | n.a. | n.a. | n.a. | n.a. | n.a. | n.a. | n.a. | + | n.a. | n.a. | ! | + | **low** |
| Lindner et al. 2022 | + | + | n.a. | n.a. | n.a. | n.a. | n.a. | + | n.a. | n.a. | n.a. | n.a. | n.a. | n.a. | n.a. | n.a. | n.a. | n.a. | n.a. | ! | n.a. | n.a. | ! | + | **moderate** |
| Yunhe Liu et al. 2025 | + | + | n.a. | ! | n.a. | n.a. | n.a. | n.a. | n.a. | n.a. | n.a. | n.a. | n.a. | n.a. | n.a. | n.a. | n.a. | n.a. | n.a. | + | + | n.a. | + | + | **low** |
| Luo et al. 2018 | + | + | n.a. | n.a. | n.a. | n.a. | n.a. | n.a. | + | n.a. | n.a. | n.a. | n.a. | n.a. | n.a. | n.a. | n.a. | n.a. | n.a. | + | n.a. | n.a. | ! | + | **low** |
| Pennestri et al. 2024 | + | + | + | n.a. | n.a. | n.a. | + | n.a. | n.a. | n.a. | + | n.a. | n.a. | n.a. | n.a. | n.a. | n.a. | n.a. | n.a. | + | n.a. | n.a. | ! | + | **low** |
| Pardo et al. 2021 | + | + | + | ! | n.a. | n.a. | + | + | n.a. | n.a. | n.a. | n.a. | n.a. | n.a. | n.a. | n.a. | n.a. | n.a. | n.a. | + | + | n.a. | ! | + | **moderate** |
| Rodolico et al. 2007 | + | + | + | ! | ! | n.a. | + | + | n.a. | n.a. | + | n.a. | n.a. | n.a. | n.a. | n.a. | n.a. | n.a. | n.a. | ! | n.a. | n.a. | - | + | **high** |
| Song et al. 2009 | + | + | + | n.a. | n.a. | n.a. | n.a. | n.a. | n.a. | n.a. | n.a. | n.a. | n.a. | n.a. | n.a. | n.a. | n.a. | n.a. | n.a. | + | n.a. | n.a. | - | + | **moderate** |
| Su et al. 2024 | + | + | + | ! | ! | n.a. | + | n.a. | n.a. | n.a. | n.a. | n.a. | n.a. | n.a. | n.a. | n.a. | n.a. | n.a. | n.a. | ! | n.a. | n.a. | ! | + | **high** |
| Sun et al. 2022 | + | + | + | ! | ! | n.a. | + | n.a. | n.a. | - | n.a. | n.a. | n.a. | n.a. | n.a. | n.a. | n.a. | n.a. | n.a. | + | n.a. | n.a. | ! | + | **high** |
| Tacchi et al. 2025 | + | + | + | ! | n.a. | n.a. | + | n.a. | n.a. | + | n.a. | n.a. | n.a. | n.a. | n.a. | n.a. | n.a. | n.a. | n.a. | ! | n.a. | n.a. | ! | + | **high** |
| Tallini et al. 2015 | + | + | n.a. | n.a. | n.a. | n.a. | n.a. | n.a. | n.a. | n.a. | + | n.a. | n.a. | n.a. | n.a. | n.a. | n.a. | n.a. | n.a. | + | + | n.a. | ! | + | **low** |
| Tian et al. 2024 | + | + | + | ! | ! | n.a. | n.a. | n.a. | n.a. | ! | + | n.a. | n.a. | n.a. | n.a. | n.a. | n.a. | n.a. | n.a. | + | n.a. | n.a. | ! | + | **high** |
| Zhou et al. 2025 | + | + | + | n.a. | n.a. | n.a. | + | n.a. | + | - | n.a. | n.a. | n.a. | n.a. | n.a. | n.a. | n.a. | n.a. | n.a. | + | n.a. | n.a. | ! | + | **high** |
| Xiaojun Zhang et al. 2021 | + | + | + | ! | ! | n.a. | + | n.a. | + | - | + | n.a. | n.a. | n.a. | n.a. | n.a. | n.a. | n.a. | n.a. | + | n.a. | n.a. | ! | + | **high** |
| Yin Zhu Zhao et al. 2022 | + | + | + | n.a. | n.a. | n.a. | + | n.a. | n.a. | - | + | n.a. | n.a. | n.a. | n.a. | n.a. | n.a. | n.a. | n.a. | + | n.a. | n.a. | ! | + | **high** |
| Yoo et al. 2009 | + | + | n.a. | n.a. | ! | n.a. | n.a. | n.a. | n.a. | n.a. | n.a. | n.a. | n.a. | n.a. | n.a. | n.a. | n.a. | n.a. | n.a. | ! | n.a. | n.a. | - | + | **high** |
| Zahan et al. 2016 | + | + | n.a. | n.a. | ! | n.a. | n.a. | n.a. | n.a. | n.a. | n.a. | n.a. | n.a. | n.a. | n.a. | n.a. | n.a. | n.a. | n.a. | ! | + | n.a. | ! | + | **high** |
| Xi Zhang et al. 2018 | + | + | + | ! | ! | n.a. | n.a. | n.a. | n.a. | + | n.a. | n.a. | n.a. | n.a. | n.a. | n.a. | n.a. | n.a. | n.a. | + | n.a. | n.a. | ! | + | **high** |
| Ge et al. 2024 | + | + | + | ! | n.a. | n.a. | n.a. | n.a. | n.a. | ! | n.a. | n.a. | n.a. | n.a. | n.a. | n.a. | n.a. | n.a. | n.a. | ! | n.a. | + | ! | + | **high** |
| Lee et al. 2022 | + | + | + | ! | ! | n.a. | n.a. | n.a. | n.a. | n.a. | n.a. | n.a. | n.a. | n.a. | n.a. | n.a. | n.a. | n.a. | n.a. | ! | - | + | ! | + | **high** |
| Ito et al. 2014 | + | + | n.a. | ! | n.a. | n.a. | n.a. | n.a. | n.a. | n.a. | n.a. | n.a. | n.a. | n.a. | n.a. | n.a. | n.a. | n.a. | n.a. | ! | n.a. | + | + | + | **moderate** |
| Kwon et al. 2017 | + | + | + | ! | + | n.a. | n.a. | n.a. | n.a. | ! | n.a. | n.a. | n.a. | n.a. | n.a. | n.a. | n.a. | n.a. | n.a. | n.a. | n.a. | + | - | + | **high** |
| Nagaoka et al. 2021 | **+** | + | + | ! | ! | n.a. | + | n.a. | n.a. | ! | n.a. | n.a. | n.a. | n.a. | n.a. | n.a. | n.a. | n.a. | n.a. | ! | n.a. | + | ! | + | **high** |
| Rosario et al. 2021 | + | + | n.a. | n.a. | n.a. | n.a. | n.a. | n.a. | n.a. | n.a. | n.a. | n.a. | n.a. | n.a. | n.a. | n.a. | n.a. | n.a. | + | n.a. | n.a. | + | - | + | **high** |
| Shindo et al. 2014 | + | + | n.a. | n.a. | n.a. | n.a. | n.a. | n.a. | n.a. | n.a. | n.a. | n.a. | n.a. | n.a. | n.a. | n.a. | n.a. | n.a. | + | n.a. | n.a. | + | - | + | **high** |
| Ghirri et al. 2023 | + | + | n.a. | n.a. | n.a. | n.a. | n.a. | n.a. | n.a. | n.a. | n.a. | n.a. | n.a. | n.a. | n.a. | n.a. | n.a. | n.a. | + | n.a. | n.a. | + | - | + | **high** |
| Ito et al. 2016 | + | + | n.a. | n.a. | n.a. | n.a. | n.a. | n.a. | n.a. | n.a. | n.a. | n.a. | n.a. | n.a. | n.a. | n.a. | n.a. | n.a. | + | n.a. | n.a. | + | - | + | **high** |
| Wen Liu et al. 2022 | + | + | n.a. | n.a. | n.a. | n.a. | n.a. | n.a. | n.a. | n.a. | n.a. | n.a. | n.a. | n.a. | n.a. | n.a. | n.a. | n.a. | + | n.a. | n.a. | + | - | + | **high** |
| Biase et al. 2015 | + | + | n.a. | n.a. | n.a. | n.a. | n.a. | n.a. | n.a. | n.a. | + | + | n.a. | n.a. | n.a. | n.a. | n.a. | n.a. | n.a. | + | n.a. | n.a. | - | + | **high** |
| Liu et al. 2017 | + | + | n.a. | n.a. | n.a. | n.a. | n.a. | n.a. | n.a. | n.a. | n.a. | n.a. | n.a. | ! | n.a. | n.a. | n.a. | n.a. | n.a. | + | n.a. | n.a. | - | + | **high** |

| + | **low risk** |
| --- | --- |
| - | **high risk** |
| ! | **moderate risk** |
| n.a. | **not applicable** |

4. REFERENCE

1. Kim KJ, Kim SG, Tan J, et al. BRAF V600E status may facilitate decision-making on active surveillance of low-risk papillary thyroid microcarcinoma. Eur J Cancer 2020;124:161-169, doi:10.1016/j.ejca.2019.10.017

2. Choi SM, Kim JK, Lee CR, et al. Completion Total Thyroidectomy Is Not Necessary for Papillary Thyroid Microcarcinoma with Occult Central Lymph Node Metastasis: A Long-Term Serial Follow-Up. Cancers (Basel) 2020;12(10), doi:10.3390/cancers12103032

3. Besic N, Zgajnar J, Hocevar M, et al. Extent of thyroidectomy and lymphadenectomy in 254 patients with papillary thyroid microcarcinoma: a single-institution experience. Ann Surg Oncol 2009;16(4):920-8, doi:10.1245/s10434-009-0332-9

4. Cai YF, Wang QX, Ni CJ, et al. A scoring system is an effective tool for predicting central lymph node metastasis in papillary thyroid microcarcinoma: a case-control study. World J Surg Oncol 2016;14(1):45, doi:10.1186/s12957-016-0808-6

5. Ding W, Ruan G, Lin Y, et al. Survival outcomes of low-risk papillary thyroid carcinoma at different risk levels: a corollary for active surveillance. Front Endocrinol (Lausanne) 2023;14:1235006, doi:10.3389/fendo.2023.1235006

6. Fu GM, Wang ZH, Chen YB, et al. Analysis of Risk Factors for Lymph Node Metastases in Elderly Patients with Papillary Thyroid Micro-Carcinoma. Cancer Manag Res 2020;12:7143-7149, doi:10.2147/CMAR.S248374

7. Yang P, Yang L, Dong Y, et al. Impact of intraoperative frozen section pathology on the treatment outcome of unilateral papillary thyroid microcarcinoma and its influencing factors-a retrospective cohort study. Gland Surg 2022;11(10):1656-1664, doi:10.21037/gs-22-519

8. Liu X, Zhu L, Cui D, et al. Coexistence of Histologically Confirmed Hashimoto's Thyroiditis with Different Stages of Papillary Thyroid Carcinoma in a Consecutive Chinese Cohort. Int J Endocrinol 2014;2014:769294, doi:10.1155/2014/769294

9. Jin L, Zhu K, Xu C, et al. Incidence and risk factors for occult lesions in low-risk papillary thyroid microcarcinoma patients with tumor characteristics appropriate for thermal ablation: A retrospective study. Medicine (Baltimore) 2023;102(38):e34938, doi:10.1097/MD.0000000000034938

10. Kaliszewski K, Diakowska D, Rzeszutko M, et al. Risk factors of papillary thyroid microcarcinoma that predispose patients to local recurrence. PLoS One 2020;15(12):e0244930, doi:10.1371/journal.pone.0244930

11. Lee S, Bae JS, Jung CK, et al. Extensive lymphatic spread of papillary thyroid microcarcinoma is associated with an increase in expression of genes involved in epithelial-mesenchymal transition and cancer stem cell-like properties. Cancer Med 2019;8(15):6528-6537, doi:10.1002/cam4.2544

12. Amendola S, Piticchio T, Scappaticcio L, et al. Papillary thyroid carcinoma: </= 10 mm does not always mean pN0. A multicentric real-world study. Updates Surg 2024;76(3):1055-1061, doi:10.1007/s13304-024-01779-6

13. Xiang T, Yan W, Zhou L. Retrospective analysis of prognostic factors in patients of papillary thyroid microcarcinoma. Oncotarget 2018;9(85):35553-35558, doi:10.18632/oncotarget.26248

14. Zhang L, Yang J, Sun Q, et al. Risk factors for lymph node metastasis in papillary thyroid microcarcinoma: Older patients with fewer lymph node metastases. Eur J Surg Oncol 2016;42(10):1478-82, doi:10.1016/j.ejso.2016.07.002

15. Zhu M, Zheng W, Xiang Y, et al. The relationship between central lymph node metastasis and the distance from tumor to thyroid capsule in papillary thyroid microcarcinoma without capsule invasion. Gland Surg 2020;9(3):727-736, doi:10.21037/gs-20-478

16. Han B, Hao S, Wu J, et al. Predictive features of central lymph node metastasis in papillary thyroid microcarcinoma: Roles of active surveillance in over-treatment. Front Med (Lausanne) 2022;9:906648, doi:10.3389/fmed.2022.906648

17. Liu W, Yan X, Dong Z, et al. A Mathematical Model to Assess the Effect of Residual Positive Lymph Nodes on the Survival of Patients With Papillary Thyroid Microcarcinoma. Front Oncol 2022;12:855830, doi:10.3389/fonc.2022.855830

18. Shi Y, Yang Z, Heng Y, et al. Clinicopathological Findings Associated With Cervical Lymph Node Metastasis in Papillary Thyroid Microcarcinoma: A Retrospective Study in China. Cancer Control 2022;29:10732748221084926, doi:10.1177/10732748221084926

19. Lee NS, Bae JS, Jeong S-R, et al. Risk Factors of Lymph Node Metastasis in Papillary Thyroid Microcarcinoma. Journal of the Korean Surgical Society 2010;78(2), doi:10.4174/jkss.2010.78.2.82

20. Jeong SY, Kim YS, Kim KC. Predictive Factors for Central Neck Lymph Node Metastasis in Patients with Papillary Thyroid Microcarcinoma without Suspicious Metastasis by Preoperative Ultrasonography. Journal of Endocrine Surgery 2017;17(3), doi:10.16956/jes.2017.17.3.114

21. Luo QW, Gao S, Lv X, et al. A novel tool for predicting the risk of central lymph node metastasis in patients with papillary thyroid microcarcinoma: a retrospective cohort study. BMC Cancer 2022;22(1):606, doi:10.1186/s12885-022-09655-5

22. Iscan Y, Sormaz IC, Tunca F, et al. Multicentricity Is More Common in Thyroid Papillary Microcancer with a Preoperative Diagnosis Compared to Incidental Microcancer. Eur Thyroid J 2019;8(5):256-261, doi:10.1159/000501613

23. Medas F, Canu GL, Cappellacci F, et al. Predictive Factors of Lymph Node Metastasis in Patients With Papillary Microcarcinoma of the Thyroid: Retrospective Analysis on 293 Cases. Front Endocrinol (Lausanne) 2020;11:551, doi:10.3389/fendo.2020.00551

24. Ye J, Feng JW, Wu WX, et al. Papillary Thyroid Microcarcinoma: A Nomogram Based on Clinical and Ultrasound Features to Improve the Prediction of Lymph Node Metastases in the Central Compartment. Front Endocrinol (Lausanne) 2021;12(770824, doi:10.3389/fendo.2021.770824

25. Wang D, Hu J, Deng C, et al. Predictive nomogram for central lymph node metastasis in papillary thyroid microcarcinoma based on pathological and ultrasound features. Front Endocrinol (Lausanne) 2023;14(1108125, doi:10.3389/fendo.2023.1108125

26. Yin Y, Xu X, Shen L, et al. Influencing Factors and Cumulative Risk Analysis of Cervical Lymph Node Metastasis of Papillary Thyroid Microcarcinoma. Front Oncol 2021;11(644645, doi:10.3389/fonc.2021.644645

27. Zhao J, Zhao Y, Ling Y, et al. Risk Factors of Central Lymph Node Metastasis in Papillary Thyroid Microcarcinoma and the Value of Sentinel Lymph Node Biopsy. Front Surg 2021;8(680493, doi:10.3389/fsurg.2021.680493

28. Cheng F, Chen Y, Zhu L, et al. Risk Factors for Cervical Lymph Node Metastasis of Papillary Thyroid Microcarcinoma: A Single-Center Retrospective Study. Int J Endocrinol 2019;2019:8579828, doi:10.1155/2019/8579828

29. Zhang Q, Wang Z, Meng X, et al. Predictors for central lymph node metastases in CN0 papillary thyroid microcarcinoma (mPTC): A retrospective analysis of 1304 cases. Asian J Surg 2019;42(4):571-576, doi:10.1016/j.asjsur.2018.08.013

30. Kim WY, Kim HY, Son GS, et al. Clinicopathological, immunohistochemical factors and recurrence associated with extrathyroidal extension in papillary thyroid microcarcinoma. J Cancer Res Ther 2014;10(1):50-5, doi:10.4103/0973-1482.131366

31. Yoon HY, Lee JH, Kim YE, et al. Clinical Significance of Histogram Parameters on Elastography in Patients With Papillary Thyroid Microcarcinomas. Ultrasound Q 2017;33(3):219-224, doi:10.1097/RUQ.0000000000000296

32. Yan L, Blanco J, Reddy V, et al. Clinicopathological features of papillary thyroid microcarcinoma with a diameter less than or equal to 5 mm. Am J Otolaryngol 2019;40(4):560-563, doi:10.1016/j.amjoto.2019.05.003

33. Dirikoc A, Tam AA, Ince N, et al. Papillary thyroid microcarcinomas that metastasize to lymph nodes. Am J Otolaryngol 2021;42(5):103023, doi:10.1016/j.amjoto.2021.103023

34. Wang D, Zhu J, Deng C, et al. Preoperative and pathological predictive factors of central lymph node metastasis in papillary thyroid microcarcinoma. Auris Nasus Larynx 2022;49(4):690-696, doi:10.1016/j.anl.2021.12.006

35. Chang YW, Kim HS, Kim HY, et al. Should central lymph node dissection be considered for all papillary thyroid microcarcinoma? Asian J Surg 2016;39(4):197-201, doi:10.1016/j.asjsur.2015.02.006

36. Liu J, Fan XF, Yang M, et al. Analysis of the risk factors for central lymph-node metastasis of cN0 papillary thyroid microcarcinoma: A retrospective study. Asian J Surg 2022;45(8):1525-1529, doi:10.1016/j.asjsur.2021.09.014

37. Zhao L, Sun X, Luo Y, et al. Clinical and pathologic predictors of lymph node metastasis in papillary thyroid microcarcinomas. Ann Diagn Pathol 2020;49:151647, doi:10.1016/j.anndiagpath.2020.151647

38. Yesiloglu AA, Uguz AH, Erdogan KE, et al. Papillary microcarcinoma of the thyroid gland: Evaluation of TERT and BRAFV-600E expression and their relationship with clinicopathological findings. Ann Diagn Pathol 2024;73:152369, doi:10.1016/j.anndiagpath.2024.152369

39. Tam AA, Ozdemir D, Cuhaci N, et al. Can ratio of the biggest tumor diameter to total tumor diameter be a new parameter in the differential diagnosis of agressive and favorable multifocal papillary thyroid microcarcinoma? Oral Oncol 2017;65:1-7, doi:10.1016/j.oraloncology.2016.12.004

40. Zhao H, Liu CH, Lu Y, et al. BRAF(V600E) mutation does not predict lymph node metastases and recurrence in Chinese papillary thyroid microcarcinoma patients. Oral Oncol 2024;152:106755, doi:10.1016/j.oraloncology.2024.106755

41. Wang Y, Guan Q, Xiang J. Nomogram for predicting central lymph node metastasis in papillary thyroid microcarcinoma: A retrospective cohort study of 8668 patients. Int J Surg 2018;55:98-102, doi:10.1016/j.ijsu.2018.05.023

42. Sheng L, Shi J, Han B, et al. Predicting factors for central or lateral lymph node metastasis in conventional papillary thyroid microcarcinoma. Am J Surg 2020;220(2):334-340, doi:10.1016/j.amjsurg.2019.11.032

43. Xiao Y, Zhou P, Zheng Y, et al. A Nomogram for Predicting Lateral Lymph Node Metastasis in Cases of Papillary Thyroid Micro-Carcinoma with Suspected Lymph Node Metastasis. Oncologie 2021;23(2):219-228, doi:10.32604/Oncologie.2021.016480

44. Bastos AU, Oler G, Nozima BH, et al. BRAF V600E and decreased NIS and TPO expression are associated with aggressiveness of a subgroup of papillary thyroid microcarcinoma. Eur J Endocrinol 2015;173(4):525-40, doi:10.1530/EJE-15-0254

45. Chen B, Zhang Z, Wang K, et al. Association of BRAFV600E mutation with ultrasonographic features and clinicopathologic characteristics of papillary thyroid microcarcinoma: A retrospective study of 116 cases. Clin Hemorheol Microcirc 2019;73(4):545-552, doi:10.3233/CH-190568

46. Jeon MJ, Chung MS, Kwon H, et al. Features of papillary thyroid microcarcinoma associated with lateral cervical lymph node metastasis. Clin Endocrinol (Oxf) 2017;86(6):845-851, doi:10.1111/cen.13322

47. Kim TY, Kim WB, Song JY, et al. The BRAF mutation is not associated with poor prognostic factors in Korean patients with conventional papillary thyroid microcarcinoma. Clin Endocrinol (Oxf) 2005;63(5):588-93, doi:10.1111/j.1365-2265.2005.02389.x

48. Kim YS. Patterns and predictive factors of lateral lymph node metastasis in papillary thyroid microcarcinoma. Otolaryngol Head Neck Surg 2012;147(1):15-9, doi:10.1177/0194599812439277

49. Kim E, Choi JY, Koo do H, et al. Differences in the characteristics of papillary thyroid microcarcinoma </=5 mm and >5 mm in diameter. Head Neck 2015;37(5):694-7, doi:10.1002/hed.23654

50. Kwak JY, Kim EK, Chung WY, et al. Association of BRAFV600E mutation with poor clinical prognostic factors and US features in Korean patients with papillary thyroid microcarcinoma. Radiology 2009;253(3):854-60, doi:10.1148/radiol.2533090471

51. Lee SH, Lee SS, Jin SM, et al. Predictive factors for central compartment lymph node metastasis in thyroid papillary microcarcinoma. Laryngoscope 2008;118(4):659-62, doi:10.1097/MLG.0b013e318161f9d1

52. Lim DJ, Baek KH, Lee YS, et al. Clinical, histopathological, and molecular characteristics of papillary thyroid microcarcinoma. Thyroid 2007;17(9):883-8, doi:10.1089/thy.2007.0001

53. Lim YC, Choi EC, Yoon YH, et al. Central lymph node metastases in unilateral papillary thyroid microcarcinoma. Br J Surg 2009;96(3):253-7, doi:10.1002/bjs.6484

54. Oh HS, Park S, Kim M, et al. Young Age and Male Sex Are Predictors of Large-Volume Central Neck Lymph Node Metastasis in Clinical N0 Papillary Thyroid Microcarcinomas. Thyroid 2017;27(10):1285-1290, doi:10.1089/thy.2017.0250

55. Park JP, Roh JL, Lee JH, et al. Risk factors for central neck lymph node metastasis of clinically noninvasive, node-negative papillary thyroid microcarcinoma. Am J Surg 2014;208(3):412-8, doi:10.1016/j.amjsurg.2013.10.032

56. Jin ZQ, Lin MY, Hu WH, et al. Gray-scale ultrasonography combined with elastography imaging for the evaluation of papillary thyroid microcarcinoma: as a prognostic clinicopathology factor. Ultrasound Med Biol 2014;40(8):1769-77, doi:10.1016/j.ultrasmedbio.2014.02.015

57. Roti E, Rossi R, Trasforini G, et al. Clinical and histological characteristics of papillary thyroid microcarcinoma: results of a retrospective study in 243 patients. J Clin Endocrinol Metab 2006;91(6):2171-8, doi:10.1210/jc.2005-2372

58. Pisanu A, Reccia I, Nardello O, et al. Risk factors for nodal metastasis and recurrence among patients with papillary thyroid microcarcinoma: differences in clinical relevance between nonincidental and incidental tumors. World J Surg 2009;33(3):460-8, doi:10.1007/s00268-008-9870-8

59. Lee KJ, Cho YJ, Kim SJ, et al. Analysis of the clinicopathologic features of papillary thyroid microcarcinoma based on 7-mm tumor size. World J Surg 2011;35(2):318-23, doi:10.1007/s00268-010-0886-5

60. Lombardi CP, Bellantone R, De Crea C, et al. Papillary thyroid microcarcinoma: extrathyroidal extension, lymph node metastases, and risk factors for recurrence in a high prevalence of goiter area. World J Surg 2010;34(6):1214-21, doi:10.1007/s00268-009-0375-x

61. Kim KE, Kim EK, Yoon JH, et al. Preoperative prediction of central lymph node metastasis in thyroid papillary microcarcinoma using clinicopathologic and sonographic features. World J Surg 2013;37(2):385-91, doi:10.1007/s00268-012-1826-3

62. Lee CR, Son H, Lee S, et al. Lobectomy and prophylactic central neck dissection for papillary thyroid microcarcinoma: do involved lymph nodes mandate completion thyroidectomy? World J Surg 2014;38(4):872-7, doi:10.1007/s00268-013-2348-3

63. Lee HS, Park HS, Kim SW, et al. Clinical characteristics of papillary thyroid microcarcinoma less than or equal to 5 mm on ultrasonography. Eur Arch Otorhinolaryngol 2013;270(11):2969-74, doi:10.1007/s00405-013-2634-6

64. Jeong SY, Chung SR, Baek JH, et al. Sonographic assessment of minor extrathyroidal extension of papillary thyroid microcarcinoma involving the posterior thyroid capsule. Eur Radiol 2022;32(9):6090-6096, doi:10.1007/s00330-022-08765-9

65. Zhang C, Li BJ, Liu Z, et al. Predicting the factors associated with central lymph node metastasis in clinical node-negative (cN0) papillary thyroid microcarcinoma. Eur Arch Otorhinolaryngol 2020;277(4):1191-1198, doi:10.1007/s00405-020-05787-1

66. Huang H, Xu S, Ni S, et al. Hashimoto's thyroiditis is negatively associated with lymph node metastasis in PTMC. J Cancer Res Clin Oncol 2023;149(17):15525-15533, doi:10.1007/s00432-023-05332-7

67. Lee X, Gao M, Ji Y, et al. Analysis of differential BRAF(V600E) mutational status in high aggressive papillary thyroid microcarcinoma. Ann Surg Oncol 2009;16(2):240-5, doi:10.1245/s10434-008-0233-3

68. Koo BS, Lim HS, Lim YC, et al. Occult contralateral carcinoma in patients with unilateral papillary thyroid microcarcinoma. Ann Surg Oncol 2010;17(4):1101-5, doi:10.1245/s10434-009-0906-6

69. Lin KL, Wang OC, Zhang XH, et al. The BRAF mutation is predictive of aggressive clinicopathological characteristics in papillary thyroid microcarcinoma. Ann Surg Oncol 2010;17(12):3294-300, doi:10.1245/s10434-010-1129-6

70. Moon HJ, Kim EK, Chung WY, et al. Minimal extrathyroidal extension in patients with papillary thyroid microcarcinoma: is it a real prognostic factor? Ann Surg Oncol 2011;18(7):1916-23, doi:10.1245/s10434-011-1556-z

71. Hyun SM, Song HY, Kim SY, et al. Impact of combined prophylactic unilateral central neck dissection and hemithyroidectomy in patients with papillary thyroid microcarcinoma. Ann Surg Oncol 2012;19(2):591-6, doi:10.1245/s10434-011-1995-6

72. Zhao Q, Ming J, Liu C, et al. Multifocality and total tumor diameter predict central neck lymph node metastases in papillary thyroid microcarcinoma. Ann Surg Oncol 2013;20(3):746-52, doi:10.1245/s10434-012-2654-2

73. Zheng X, Wei S, Han Y, et al. Papillary microcarcinoma of the thyroid: clinical characteristics and BRAF(V600E) mutational status of 977 cases. Ann Surg Oncol 2013;20(7):2266-73, doi:10.1245/s10434-012-2851-z

74. Kim SK, Park I, Woo JW, et al. Predictive Factors for Lymph Node Metastasis in Papillary Thyroid Microcarcinoma. Ann Surg Oncol 2016;23(9):2866-73, doi:10.1245/s10434-016-5225-0

75. Lee YH, Lee YM, Sung TY, et al. Is Male Gender a Prognostic Factor for Papillary Thyroid Microcarcinoma? Ann Surg Oncol 2017;24(7):1958-1964, doi:10.1245/s10434-017-5788-4

76. Back K, Kim JS, Kim JH, et al. Superior Located Papillary Thyroid Microcarcinoma is a Risk Factor for Lateral Lymph Node Metastasis. Ann Surg Oncol 2019;26(12):3992-4001, doi:10.1245/s10434-019-07587-2

77. Shin HJ, Kim EK, Moon HJ, et al. Can increased tumoral vascularity be a quantitative predicting factor of lymph node metastasis in papillary thyroid microcarcinoma? Endocrine 2014;47(1):273-82, doi:10.1007/s12020-013-0131-8

78. Choi JS, Kim EK, Moon HJ, et al. Higher body mass index may be a predictor of extrathyroidal extension in patients with papillary thyroid microcarcinoma. Endocrine 2015;48(1):264-71, doi:10.1007/s12020-014-0293-z

79. Pisanu A, Saba A, Podda M, et al. Nodal metastasis and recurrence in papillary thyroid microcarcinoma. Endocrine 2015;48(2):575-81, doi:10.1007/s12020-014-0350-7

80. Kim K, Zheng X, Kim JK, et al. The contributing factors for lateral neck lymph node metastasis in papillary thyroid microcarcinoma (PTMC). Endocrine 2020;69(1):149-156, doi:10.1007/s12020-020-02251-2

81. Zuhur SS, Aggul H, Avci U, et al. The impact of microscopic extrathyroidal extension on the clinical outcome of classic subtype papillary thyroid microcarcinoma: a multicenter study. Endocrine 2024;83(3):700-707, doi:10.1007/s12020-023-03533-1

82. Cao J, Chen B, Zhu X, et al. BRAF (V600E) mutation in papillary thyroid microcarcinoma: is it a predictor for the prognosis of patients with intermediate to high recurrence risk? Endocrine 2024;84(1):160-170, doi:10.1007/s12020-023-03564-8

83. Kim HS, Choi YJ, Yun JS. Features of papillary thyroid microcarcinoma in the presence and absence of lymphocytic thyroiditis. Endocr Pathol 2010;21(3):149-53, doi:10.1007/s12022-010-9124-9

84. Zeng RC, Li Q, Lin KL, et al. Predicting the factors of lateral lymph node metastasis in papillary microcarcinoma of the thyroid in eastern China. Clin Transl Oncol 2012;14(11):842-7, doi:10.1007/s12094-012-0875-2

85. Qu N, Zhang L, Lin DZ, et al. The impact of coexistent Hashimoto's thyroiditis on lymph node metastasis and prognosis in papillary thyroid microcarcinoma. Tumour Biol 2016;37(6):7685-92, doi:10.1007/s13277-015-4534-4

86. Gui CY, Qiu SL, Peng ZH, et al. Clinical and pathologic predictors of central lymph node metastasis in papillary thyroid microcarcinoma: a retrospective cohort study. J Endocrinol Invest 2018;41(4):403-409, doi:10.1007/s40618-017-0759-y

87. Ahn D, Sohn JH, Jeon JH, et al. Clinical impact of microscopic extrathyroidal extension in patients with papillary thyroid microcarcinoma treated with hemithyroidectomy. J Endocrinol Invest 2014;37(2):167-73, doi:10.1007/s40618-013-0025-x

88. Feng JW, Ye J, Wu WX, et al. Management of cN0 papillary thyroid microcarcinoma patients according to risk-scoring model for central lymph node metastasis and predictors of recurrence. J Endocrinol Invest 2020;43(12):1807-1817, doi:10.1007/s40618-020-01326-1

89. Lee J, Ha EJ, Roh J, et al. Presence of TERT +/- BRAF V600E mutation is not a risk factor for the clinical management of patients with papillary thyroid microcarcinoma. Surgery 2021;170(3):743-747, doi:10.1016/j.surg.2021.03.056

90. Siddiqui S, White MG, Antic T, et al. Clinical and Pathologic Predictors of Lymph Node Metastasis and Recurrence in Papillary Thyroid Microcarcinoma. Thyroid 2016;26(6):807-15, doi:10.1089/thy.2015.0429

91. Varshney R, Pakdaman MN, Sands N, et al. Lymph node metastasis in thyroid papillary microcarcinoma: a study of 170 patients. J Laryngol Otol 2014;128(10):922-5, doi:10.1017/S0022215114001704

92. Vasileiadis I, Karakostas E, Charitoudis G, et al. Papillary thyroid microcarcinoma: clinicopathological characteristics and implications for treatment in 276 patients. Eur J Clin Invest 2012;42(6):657-64, doi:10.1111/j.1365-2362.2011.02633.x

93. Wang WH, Xu SY, Zhan WW. Clinicopathologic Factors and Thyroid Nodule Sonographic Features for Predicting Central Lymph Node Metastasis in Papillary Thyroid Microcarcinoma: A Retrospective Study of 1204 Patients. J Ultrasound Med 2016;35(11):2475-2481, doi:10.7863/ultra.15.10012

94. Wu X, Li B, Zheng C, et al. Risk Factors for Central Lymph Node Metastases in Patients with Papillary Thyroid Microcarcinoma. Endocr Pract 2018;24(12):1057-1062, doi:10.4158/EP-2018-0305

95. Xiang D, Xie L, Xu Y, et al. Papillary thyroid microcarcinomas located at the middle part of the middle third of the thyroid gland correlates with the presence of neck metastasis. Surgery 2015;157(3):526-33, doi:10.1016/j.surg.2014.10.020

96. Xu Y, Xu L, Wang J. Clinical predictors of lymph node metastasis and survival rate in papillary thyroid microcarcinoma: analysis of 3607 patients at a single institution. J Surg Res 2018;221:128-134, doi:10.1016/j.jss.2017.08.007

97. Yan T, Qiu W, Song J, et al. Bilateral multifocality, a marker for aggressive disease, is not an independent prognostic factor for papillary thyroid microcarcinoma: A propensity score matching analysis. Clin Endocrinol (Oxf) 2021;95(1):209-216, doi:10.1111/cen.14455

98. Yu X, Song X, Sun W, et al. Independent Risk Factors Predicting Central Lymph Node Metastasis in Papillary Thyroid Microcarcinoma. Horm Metab Res 2017;49(3):201-207, doi:10.1055/s-0043-101917

99. Yuan J, Li J, Chen X, et al. Identification of risk factors of central lymph node metastasis and evaluation of the effect of prophylactic central neck dissection on migration of staging and risk stratification in patients with clinically node-negative papillary thyroid microcarcinoma. Bull Cancer 2017;104(6):516-523, doi:10.1016/j.bulcan.2017.03.005

100. Zeng RC, Zhang W, Gao EL, et al. Number of central lymph node metastasis for predicting lateral lymph node metastasis in papillary thyroid microcarcinoma. Head Neck 2014;36(1):101-6, doi:10.1002/hed.23270

101. Zhang L, Wei WJ, Ji QH, et al. Risk factors for neck nodal metastasis in papillary thyroid microcarcinoma: a study of 1066 patients. J Clin Endocrinol Metab 2012;97(4):1250-7, doi:10.1210/jc.2011-1546

102. Zheng W, Wang X, Rui Z, et al. Clinical features and therapeutic outcomes of patients with papillary thyroid microcarcinomas and larger tumors. Nucl Med Commun 2019;40(5):477-483, doi:10.1097/MNM.0000000000000991

103. Zhao C, Jiang W, Gao Y, et al. Risk factors for lymph node metastasis (LNM) in patients with papillary thyroid microcarcinoma (PTMC): role of preoperative ultrasound. J Int Med Res 2017;45(3):1221-1230, doi:10.1177/0300060517708943

104. Gao L, Wen X, Yue G, et al. The Predictive Value of a Nomogram Based on Ultrasound Radiomics, Clinical Factors, and Enhanced Ultrasound Features for Central Lymph Node Metastasis in Papillary Thyroid Microcarcinoma. Ultrason Imaging 2025;47(2):93-103, doi:10.1177/01617346251313982

105. Ozemir IA, Şermet M. Association of multicentricity and prognostic factors of papillary microcarcinoma. Ann Clin Anal Med 2023;14(12), doi:10.4328/acam.21909

106. Zhao W, Chen S, Hou X, et al. Predictive Factors of Lateral Lymph Node Metastasis in Papillary Thyroid Microcarcinoma. Pathol Oncol Res 2019;25(3):1245-1251, doi:10.1007/s12253-018-0511-8

107. Zhou YL, Gao EL, Zhang W, et al. Factors predictive of papillary thyroid micro-carcinoma with bilateral involvement and central lymph node metastasis: a retrospective study. World J Surg Oncol 2012;10:67, doi:10.1186/1477-7819-10-67

108. Choi SY, Park H, Kang MK, et al. The relationship between the BRAF(V600E) mutation in papillary thyroid microcarcinoma and clinicopathologic factors. World J Surg Oncol 2013;11:291, doi:10.1186/1477-7819-11-291

109. Seifert R, Schafers MA, Heitplatz B, et al. Minimal extrathyroid extension in papillary micro carcinoma of the thyroid is an independent risk factor for relapse through lymph node and distant metastases. J Nucl Med 2021;62(12):1702-9, doi:10.2967/jnumed.121.261898

110. Kayhan Y, Azizova L, Yilmaz M, et al. Prognostic factors for aggressiveness in subcentimeter papillary thyroid carcinoma: impact of tumor size and lymph node metastases. Arch Endocrinol Metab 2024;68:e230422, doi:10.20945/2359-4292-2023-0422

111. Zhang X, Zhu J, Ai X, et al. An ultrasound-based nomogram for predicting central lymph node metastasis in papillary thyroid microcarcinoma. Med Ultrason 2024;26(4):369-375, doi:10.11152/mu-4411

112. Zhi J, Zhao J, Gao M, et al. Impact of major different variants of papillary thyroid microcarcinoma on the clinicopathological characteristics: the study of 1041 cases. Int J Clin Oncol 2018;23(1):59-65, doi:10.1007/s10147-017-1170-6

113. Oh EM, Chung YS, Song WJ, et al. The pattern and significance of the calcifications of papillary thyroid microcarcinoma presented in preoperative neck ultrasonography. Ann Surg Treat Res 2014;86(3):115-21, doi:10.4174/astr.2014.86.3.115

114. Yu Y, Yu Z, Li M, et al. Model development to predict central lymph node metastasis in cN0 papillary thyroid microcarcinoma by machine learning. Ann Transl Med 2022;10(16):892, doi:10.21037/atm-22-3594

115. Zheng X, Jiang Y, Zhao C, et al. Prognostic Value of Hyperechoic Echo Halo in cN0 Papillary Thyroid Microcarcinoma and Its Correlation with Age and Gender. Biomed Res Int 2020;2020:6479582, doi:10.1155/2020/6479582

116. Chow SM, Law SC, Chan JK, et al. Papillary microcarcinoma of the thyroid-Prognostic significance of lymph node metastasis and multifocality. Cancer 2003;98(1):31-40, doi:10.1002/cncr.11442

117. Rossi ED, Martini M, Capodimonti S, et al. BRAF (V600E) mutation analysis on liquid-based cytology-processed aspiration biopsies predicts bilaterality and lymph node involvement in papillary thyroid microcarcinoma. Cancer Cytopathol 2013;121(6):291-7, doi:10.1002/cncy.21258

118. Tagliabue M, Giugliano G, Mariani MC, et al. Prevalence of Central Compartment Lymph Node Metastases in Papillary Thyroid Micro-Carcinoma: A Retrospective Evaluation of Predictive Preoperative Features. Cancers (Basel) 2021;13(23), doi:10.3390/cancers13236028

119. Zheng X, Peng C, Gao M, et al. Risk factors for cervical lymph node metastasis in papillary thyroid microcarcinoma: a study of 1,587 patients. Cancer Biol Med 2019;16(1):121-130, doi:10.20892/j.issn.2095-3941.2018.0125

120. Wei X, Wang M, Wang X, et al. Prediction of cervical lymph node metastases in papillary thyroid microcarcinoma by sonographic features of the primary site. Cancer Biol Med 2019;16(3):587-594, doi:10.20892/j.issn.2095-3941.2018.0310

121. Lee DY, Hwang SM, An JH, et al. Predicting Extrathyroidal Extension in Patients With Papillary Thyroid Microcarcinoma According to a BRAF Mutation. Clin Exp Otorhinolaryngol 2017;10(2):174-180, doi:10.21053/ceo.2015.01655

122. Seo JW, Hwang SH, Cho A, et al. Prognostic Impact of Ultrasonography Features and (18)F-Fluorodeoxyglucose Uptake in Patients With Papillary Thyroid Microcarcinoma. Clin Exp Otorhinolaryngol 2016;9(1):62-9, doi:10.21053/ceo.2016.9.1.62

123. Song J, Yan T, Qiu W, et al. Clinical Analysis of Risk Factors for Cervical Lymph Node Metastasis in Papillary Thyroid Microcarcinoma: A Retrospective Study of 3686 Patients. Cancer Manag Res 2020;12:2523-2530, doi:10.2147/CMAR.S250163

124. Yang Y, Chen C, Chen Z, et al. Prediction of central compartment lymph node metastasis in papillary thyroid microcarcinoma. Clin Endocrinol (Oxf) 2014;81(2):282-8, doi:10.1111/cen.12417

125. Ren H, Shen Y, Hu D, et al. Co-existence of BRAF(V600E) and TERT promoter mutations in papillary thyroid carcinoma is associated with tumor aggressiveness, but not with lymph node metastasis. Cancer Manag Res 2018;10:1005-1013, doi:10.2147/CMAR.S159583

126. Zheng W, Wang K, Wu J, et al. Multifocality is associated with central neck lymph node metastases in papillary thyroid microcarcinoma. Cancer Manag Res 2018;10:1527-1533, doi:10.2147/CMAR.S163263

127. Takahito A, Fujii K, Banno H, et al. Clinicopathological Evaluation of Papillary Thyroid Microcarcinoma. Cureus 2024;16(3):e56404, doi:10.7759/cureus.56404

128. Chen SP, Jiang X, Zheng WW, et al. Correlation between Sonographic Features and Central Neck Lymph Node Metastasis in Solitary Solid Papillary Thyroid Microcarcinoma with a Taller-Than-Wide Shape. Diagnostics (Basel) 2023;13(5), doi:10.3390/diagnostics13050949

129. Kim M, Kwon CH, Jang MH, et al. Whole-Exome Sequencing in Papillary Microcarcinoma: Potential Early Biomarkers of Lateral Lymph Node Metastasis. Endocrinol Metab (Seoul) 2021;36(5):1086-1094, doi:10.3803/EnM.2021.1132

130. Lim J, Lee HS, Heo JH, et al. Clinicopathological Features and Molecular Signatures of Lateral Neck Lymph Node Metastasis in Papillary Thyroid Microcarcinoma. Endocrinol Metab (Seoul) 2024;39(2):324-333, doi:10.3803/EnM.2023.1885

131. Xue S, Wang P, Zhang Q, et al. Routine Lateral Level V Dissection May Not Be Necessary for Papillary Thyroid Microcarcinoma With Lateral Lymph Node Metastasis: A Retrospective Study of 252 Cases. Front Endocrinol (Lausanne) 2019;10:558, doi:10.3389/fendo.2019.00558

132. Sun J, Jiang Q, Wang X, et al. Nomogram for Preoperative Estimation of Cervical Lymph Node Metastasis Risk in Papillary Thyroid Microcarcinoma. Front Endocrinol (Lausanne) 2021;12:613974, doi:10.3389/fendo.2021.613974

133. Huang C, Cong S, Shang S, et al. Web-Based Ultrasonic Nomogram Predicts Preoperative Central Lymph Node Metastasis of cN0 Papillary Thyroid Microcarcinoma. Front Endocrinol (Lausanne) 2021;12:734900, doi:10.3389/fendo.2021.734900

134. Ma T, Wang L, Zhang X, et al. A clinical and molecular pathology prediction model for central lymph node metastasis in cN0 papillary thyroid microcarcinoma. Front Endocrinol (Lausanne) 2023;14:1075598, doi:10.3389/fendo.2023.1075598

135. Li WH, Yu WY, Du JR, et al. Nomogram prediction for cervical lymph node metastasis in multifocal papillary thyroid microcarcinoma. Front Endocrinol (Lausanne) 2023;14:1140360, doi:10.3389/fendo.2023.1140360

136. Zhang C, Fu S, Liu H, et al. Risk prediction for <1 cm lateral lymph node metastasis in papillary thyroid microcarcinoma. Front Endocrinol (Lausanne) 2023;14:1235354, doi:10.3389/fendo.2023.1235354

137. Huang Y, Lou P, Li H, et al. Risk nomogram for papillary thyroid microcarcinoma with central lymph node metastasis and postoperative thyroid function follow-up. Front Endocrinol (Lausanne) 2024;15:1395900, doi:10.3389/fendo.2024.1395900

138. Lai X, Zhang B, Jiang Y, et al. Sonographic and Clinical Features of Papillary Thyroid Microcarcinoma Less than or Equal to Five Millimeters: A Retrospective Study. PLoS One 2016;11(2):e0148567, doi:10.1371/journal.pone.0148567

139. Kim SY, Lee E, Nam SJ, et al. Ultrasound texture analysis: Association with lymph node metastasis of papillary thyroid microcarcinoma. PLoS One 2017;12(4):e0176103, doi:10.1371/journal.pone.0176103

140. Wang W, Zhang Z, Zhao Y, et al. Management of Lateral Multiple-Level Metastasis in N1b Papillary Thyroid Microcarcinoma. Front Oncol 2020;10:1586, doi:10.3389/fonc.2020.01586

141. Cao Z, Zhang Z, Tang X, et al. Comprehensive analysis of tissue proteomics in patients with papillary thyroid microcarcinoma uncovers the underlying mechanism of lymph node metastasis and its significant sex disparities. Front Oncol 2022;12(887977, doi:10.3389/fonc.2022.887977

142. Zhu F, Zhu L, Shen Y, et al. Differences in the clinical characteristics of papillary thyroid microcarcinoma located in the isthmus </=5 mm and >5mm in diameter. Front Oncol 2022;12(923266, doi:10.3389/fonc.2022.923266

143. Wu X, Li B, Zheng C, et al. Predicting factors of central lymph node metastases in patients with unilateral multifocal papillary thyroid microcarcinoma. Gland Surg 2020;9(3):695-701, doi:10.21037/gs.2020.03.27

144. Wang W, Bai N, Ouyang Q, et al. Prediction of level V metastases in papillary thyroid microcarcinoma: a single center analysis. Gland Surg 2020;9(4):899-906, doi:10.21037/gs-20-232

145. Zhao Y, Fu J, Liu Y, et al. Prediction of central lymph node metastasis in patients with papillary thyroid microcarcinoma by gradient-boosting decision tree model based on ultrasound radiomics and clinical features. Gland Surg 2023;12(12):1722-1734, doi:10.21037/gs-23-456

146. Liu W, Wang S, Xia X. Risk Factor Analysis for Central Lymph Node Metastasis in Papillary Thyroid Microcarcinoma. Int J Gen Med 2021;14:9923-9929, doi:10.2147/IJGM.S346143

147. Lai Y, Gu Y, Yu M, et al. Younger Than 55 Years Old and BRAF V600E Mutation are Risk Factors for Lymph Node Metastasis in Papillary Thyroid Carcinomas </=1.0 cm but Not in >1.0 cm. Int J Gen Med 2023;16:1403-1414, doi:10.2147/IJGM.S408588

148. Hitu L, Stefan PA, Piciu D. Total Tumor Diameter and Unilateral Multifocality as Independent Predictor Factors for Metastatic Papillary Thyroid Microcarcinoma. J Clin Med 2021;10(16), doi:10.3390/jcm10163707

149. Parvathareddy SK, Siraj AK, Annaiyappanaidu P, et al. Risk Factors for Cervical Lymph Node Metastasis in Middle Eastern Papillary Thyroid Microcarcinoma. J Clin Med 2022;11(15), doi:10.3390/jcm11154613

150. Yang Z, Heng Y, Qiu W, et al. Cervical Lymph Node Metastasis Differences in Patients with Unilateral or Bilateral Papillary Thyroid Microcarcinoma: A Multi-Center Analysis. J Clin Med 2022;11(16), doi:10.3390/jcm11164929

151. Cho JK, Kim JY, Jeong CY, et al. Clinical features and prognostic factors in papillary thyroid microcarcinoma depends on age. J Korean Surg Soc 2012;82(5):281-7, doi:10.4174/jkss.2012.82.5.281

152. Sun S, Zhou Q, Hu T. A model based on ultrasound and clinical factors to predict central lymph node metastasis in cN0 papillary thyroid microcarcinoma. Heliyon 2024;10(13):e33891, doi:10.1016/j.heliyon.2024.e33891

153. Jiwang L, Yahong L, Kai L, et al. Clinicopathologic factors and preoperative ultrasonographic characteristics for predicting central lymph node metastasis in papillary thyroid microcarcinoma: a single center retrospective study. Braz J Otorhinolaryngol 2022;88(1):36-45, doi:10.1016/j.bjorl.2020.05.004

154. Tao Y, Wang C, Li L, et al. Clinicopathological features for predicting central and lateral lymph node metastasis in papillary thyroid microcarcinoma: Analysis of 66 cases that underwent central and lateral lymph node dissection. Mol Clin Oncol 2017;6(1):49-55, doi:10.3892/mco.2016.1085

155. Park VY, Kim EK, Lee HS, et al. Real-Time PCR Cycle Threshold Values for the BRAFV600E Mutation in Papillary Thyroid Microcarcinoma May Be Associated With Central Lymph Node Metastasis: A Retrospective Study. Medicine (Baltimore) 2015;94(28):e1149, doi:10.1097/MD.0000000000001149

156. Xu JM, Xu HX, Li XL, et al. A Risk Model for Predicting Central Lymph Node Metastasis of Papillary Thyroid Microcarcinoma Including Conventional Ultrasound and Acoustic Radiation Force Impulse Elastography. Medicine (Baltimore) 2016;95(3):e2558, doi:10.1097/MD.0000000000002558

157. Liu Z, Lei J, Liu Y, et al. Preoperative predictors of lateral neck lymph node metastasis in papillary thyroid microcarcinoma. Medicine (Baltimore) 2017;96(10):e6240, doi:10.1097/MD.0000000000006240

158. Wu X, Li B, Zheng C, et al. Predicting factors of lateral neck lymph node metastases in patients with papillary thyroid microcarcinoma. Medicine (Baltimore) 2019;98(27):e16386, doi:10.1097/MD.0000000000016386

159. Wang X, Tan J, Zheng W, et al. A retrospective study of the clinical features in papillary thyroid microcarcinoma depending on age. Nucl Med Commun 2018;39(8):713-719, doi:10.1097/MNM.0000000000000859

160. Sezer H, Uren N, Yazici D. Association between BRAF(V600E) mutation and the clinicopathological features in incidental papillary thyroid microcarcinoma: A single-center study in Turkish patients. North Clin Istanb 2020;7(4):321-328, doi:10.14744/nci.2020.69586

161. Lu HZ, Qiu T, Ying JM, et al. Association between BRAF(V600E) mutation and the clinicopathological features of solitary papillary thyroid microcarcinoma. Oncol Lett 2017;13(3):1595-1600, doi:10.3892/ol.2017.5661

162. Zhou C, Li J, Wang Y, et al. Association of BRAF gene and TSHR with cervical lymph node metastasis of papillary thyroid microcarcinoma. Oncol Lett 2019;17(1):183-194, doi:10.3892/ol.2018.9572

163. Dong Y, Wang D, Luo Y, et al. Comprehensive evaluation of risk factors for lymph node metastasis in patients with papillary thyroid carcinoma. Oncol Lett 2021;21(3):188, doi:10.3892/ol.2021.12449

164. Wang Y, Tan HL, Duan SL, et al. Predicting central cervical lymph node metastasis in papillary thyroid microcarcinoma using deep learning. PeerJ 2024;12:e16952, doi:10.7717/peerj.16952

165. Xue J, Qu N, Liu H, et al. Value of Multimodal Ultrasound Combined with BRAF Gene in Evaluating Cervical Lymph Node Metastasis of Papillary Thyroid Microcarcinoma. Ultrasound Med Biol 2024;50(8):1183-1187, doi:10.1016/j.ultrasmedbio.2024.04.005

166. Virk RK, Van Dyke AL, Finkelstein A, et al. BRAFV600E mutation in papillary thyroid microcarcinoma: a genotype-phenotype correlation. Mod Pathol 2013;26(1):62-70, doi:10.1038/modpathol.2012.152

167. Shen Y, Pu W, Zhou H, et al. Risk factors for central lymph node metastasis in papillary thyroid microcarcinoma - a retrospective study of 1433 cases from a single center. Pol J Pathol 2022;73(3):191-197, doi:10.5114/pjp.2022.124486

168. Jin WX, Ye DR, Sun YH, et al. Prediction of central lymph node metastasis in papillary thyroid microcarcinoma according to clinicopathologic factors and thyroid nodule sonographic features: a case-control study. Cancer Manag Res 2018;10:3237-3243, doi:10.2147/CMAR.S169741

169. Zhang LY, Liu ZW, Liu YW, et al. Risk Factors for Nodal Metastasis in cN0 Papillary Thyroid Microcarcinoma. Asian Pac J Cancer Prev 2015;16(8):3361-3, doi:10.7314/apjcp.2015.16.8.3361

170. Ayesha SM, Hui M, Uppin SG, et al. Prognostic Significance of Various Clinicopathologic Parameters and BRAF V600E Mutation in Papillary Thyroid Microcarcinoma—An Observational Study. Indian Journal of Medical and Paediatric Oncology 2023;44(03):345-352, doi:10.1055/s-0043-1761412

171. Bradley NL, Wiseman SM. Papillary thyroid microcarcinoma: the significance of high risk features. BMC Cancer 2017;17(1):142, doi:10.1186/s12885-017-3120-0

172. Huang H, Xu S, Ni S, et al. A nomogram for predicting lateral lymph node metastasis in cN0 unifocal papillary thyroid microcarcinoma. BMC Cancer 2023;23(1):718, doi:10.1186/s12885-023-11219-0

173. Qiu P, Guo Q, Pan K, et al. Development of a nomogram for prediction of central lymph node metastasis of papillary thyroid microcarcinoma. BMC Cancer 2024;24(1):235, doi:10.1186/s12885-024-12004-3

174. Gu JH, Zhao YN, Xie RL, et al. Analysis of risk factors for cervical lymph node metastasis of papillary thyroid microcarcinoma: a study of 268 patients. BMC Endocr Disord 2019;19(1):124, doi:10.1186/s12902-019-0450-8

175. Xie X, Deng J, Zheng B, et al. The effect of central lymph node dissection on the prognosis of cN0 papillary thyroid microcarcinoma: a mid-term follow-up study. BMC Endocrine Disorders 2023;23(1), doi:10.1186/s12902-023-01375-6

176. Yin X, Liu C, Guo Y, et al. Influence of tumor extent on central lymph node metastasis in solitary papillary thyroid microcarcinomas: a retrospective study of 1092 patients. World J Surg Oncol 2017;15(1), doi:10.1186/s12957-017-1202-8

177. Song R-Y, Kim HS, Kang KH. Minimal extrathyroidal extension is associated with lymph node metastasis in single papillary thyroid microcarcinoma: a retrospective analysis of 814 patients. World J Surg Oncol 2022;20(1), doi:10.1186/s12957-022-02629-8

178. Cao Z, Wang Y, Wu J, et al. Serum small extracellular vesicles-derived BST2 as a biomarker for papillary thyroid microcarcinoma promotes lymph node metastasis. Cancer Gene Ther 2024;32(1):38-50, doi:10.1038/s41417-024-00854-9

179. Hong Y-R, Yan C-X, Mo G-Q, et al. Conventional US, elastography and contrast enhanced US features of papillary thyroid microcarcinoma predict central compartment lymph node metastases. Sci Rep 2015;5(1), doi:10.1038/srep07748

180. Kim B-Y, Jung C-H, Kim J-W, et al. Impact of Clinicopathologic Factors on Subclinical Central Lymph Node Metastasis in Papillary Thyroid Microcarcinoma. Yonsei Med J 2012;53(5), doi:10.3349/ymj.2012.53.5.924

181. Zhang H, Zheng X, Liu J, et al. Development of an Active Surveillance or Surgery Model to Predict Lymph Node Metastasis in cN0 Papillary Thyroid Microcarcinoma. Front Endocrinol (Lausanne) 2022;13:896121, doi:10.3389/fendo.2022.896121

182. Besic N, Pilko G, Petric R, et al. Papillary thyroid microcarcinoma: prognostic factors and treatment. J Surg Oncol 2008;97(3):221-5, doi:10.1002/jso.20935

183. Korkmaz H, Elboğa U, Akarsu E, et al. Comparison of Papillary Thyroid Carcinoma and Papillary Microcarcinoma in Terms of Clinical Features and Prognostic Factors. Endocrinol Res Pract 2016;20(3):72-77, doi:10.4274/tjem.3227

184. Liu C, Liu H, Bian C, et al. Preoperative risk factors and recommendations for surgical intervention in cN0 papillary thyroid microcarcinoma. Neoplasma 2021;68(5):1113-1117, doi:10.4149/neo_2021_210314N330

185. Ruiz Pardo J, Rios A, Rodriguez JM, et al. Risk Factors of Metastatic Lymph Nodes in Papillary Thyroid Microcarcinoma. Cir Esp (Engl Ed) 2020;98(4):219-225, doi:10.1016/j.ciresp.2019.10.003

186. Chen C, Chen HZ, Lyu CH, et al. [Clinicopathological features of thyroid papillary microcarcinoma of which the maximum diameter is less than 5 mm: a series of 487 cases]. Zhonghua Wai Ke Za Zhi 2016;54(11):870-874, doi:10.3760/cma.j.issn.0529-5815.2016.11.016

187. Wu YL, Sun JM, Zhang JJ, et al. [Clinicopathological characteristics of papillary thyroid microcarcinoma and risk factors for central lymph node metastasis]. Zhonghua Er Bi Yan Hou Tou Jing Wai Ke Za Zhi 2017;52(6):426-429, doi:10.3760/cma.j.issn.1673-0860.2017.06.006

188. Wang XQ, Wei X, Xu Y, et al. [Analysis of the relationship between ultrasonographic features and cervical lymph node skip metastasis of papillary thyroid micro-carcinoma]. Zhonghua Zhong Liu Za Zhi 2019;41(5):373-377, doi:10.3760/cma.j.issn.0253-3766.2019.05.010

189. Akkas Akgün G, Zeren S. Prognostic Factors in Thyroid Papillary Microcarcinoma. Journal of Contemporary Medicine 2023;13(2):193-197, doi:10.16899/jcm.1232902

190. Yan C, He X, Chen Z, et al. Central Compartment Lymph Nodes Have Distinct Metastatic Patterns in Different Age Groups. Front Endocrinol (Lausanne) 2022;13:807431, doi:10.3389/fendo.2022.807431

191. Gweon HM, Son EJ, Kim JA, et al. Predictive Factors for Active Surveillance of Subcentimeter Thyroid Nodules with Highly Suspicious US Features. Ann Surg Oncol 2017;24(6):1540-1545, doi:10.1245/s10434-016-5728-8

192. Huang H, Xu S, Ni S, et al. A nomogram for predicting lateral lymph node metastasis in cN0 unifocal papillary thyroid microcarcinoma. BMC Cancer 2023;23(1):718, doi:10.1186/s12885-023-11219-0

193. Yoon JH, Park JY, Hong AR, et al. Predictors of lateral lymph node metastasis and skip metastasis in patients with papillary thyroid microcarcinoma. Front Endocrinol (Lausanne) 2024;15(1392247, doi:10.3389/fendo.2024.1392247

194. Wang J, Sheng X, Dai Y, et al. The Application Value of the Central Lymph Node Metastasis Risk Assessment Model in Papillary Thyroid Microcarcinoma of Stage cN0: A Study of 828 Patients. Front Endocrinol (Lausanne) 2022;13(843573, doi:10.3389/fendo.2022.843573

195. Wang J, Fu W, Luo J, et al. Development of a clinical-molecular prediction model for central lymph node metastasis in cN0 stage papillary thyroid microcarcinoma: a retrospective study. BMC Cancer 2025;25(1):693, doi:10.1186/s12885-025-14112-0

196. Kaliszewski K, Diakowska D, Wojtczak B, et al. Which papillary thyroid microcarcinoma should be treated as "true cancer" and which as "precancer"? World J Surg Oncol 2019;17(1):91, doi:10.1186/s12957-019-1638-0

197. Kim Y-W, Wang S-G, Lee J-C, et al. Clinically Related Factors and Features of Central Compartment Neck Lymph Nodes in Thyroid Micropapillary Carcinoma. Korean Journal of Otolaryngology-Head and Neck Surgery 2009;52(3), doi:10.3342/kjorl-hns.2009.52.3.232

198. Kim WJ, Bae MJ, Yi YS, et al. Clinicopathologic Characteristics of Papillary Microcarcinoma in the Elderly. Journal of Korean Thyroid Association 2013;6(1), doi:10.11106/jkta.2013.6.1.69

199. Kim JY, Jung EJ, Park T, et al. Impact of tumor size on subclinical central lymph node metastasis in papillary thyroid microcarcinoma depends on age. World J Surg Oncol 2015;13(88, doi:10.1186/s12957-015-0478-9

200. Kim YH, Kim YS, Kim KC. Factors Influencing Central Neck Lymph Node Metastasis in Patients with Papillary Thyroid Microcarcinoma. Korean Journal of Endocrine Surgery 2016;16(3), doi:10.16956/kaes.2016.16.3.64

201. Lee SM, Lee CR, Kang S-W, et al. Association between BRAFV600E Mutations and Clinicopathological Features of Papillary Thyroid Microcarcinoma (PTMC). J Endocr Surg 2019;19(3), doi:10.16956/jes.2019.19.3.76

202. Lindner K, Iwen KA, Kussmann J, et al. Predictive Factors for Bilateral Disease in Papillary Microcarcinoma: A Retrospective Cohort Study. Curr Oncol 2022;29(9):6010-6017, doi:10.3390/curroncol29090473

203. Liu Y, Liao L, Yan D, et al. The impact of age at diagnosis on central lymph node metastasis in clinically low-risk papillary thyroid microcarcinoma patients. Thyroid Res 2025;18(1):6, doi:10.1186/s13044-025-00224-z

204. Luo Y, Zhao Y, Chen K, et al. Clinical analysis of cervical lymph node metastasis risk factors in patients with papillary thyroid microcarcinoma. J Endocrinol Invest 2019;42(2):227-236, doi:10.1007/s40618-018-0908-y

205. Pennestri F, Procopio PF, Laurino A, et al. Is conservative treatment always safe in unifocal clinically T1a/node-negative papillary thyroid carcinoma? World J Surg 2025;49(1):187-197, doi:10.1002/wjs.12440

206. Rodolico V, Cabibi D, Pizzolanti G, et al. BRAF V600E mutation and p27 kip1 expression in papillary carcinomas of the thyroid <or=1 cm and their paired lymph node metastases. Cancer 2007;110(6):1218-26, doi:10.1002/cncr.22912

207. Tacchi G, Pedicini F, Crucitti P, et al. Evaluation of predictive factors for lymph node metastasis in thyroid microcarcinoma: a two-year experience from two high-volume centers. Updates Surg 2025;77(5):1581-1591, doi:10.1007/s13304-025-02211-3

208. Tallini G, de Biase D, Durante C, et al. BRAF V600E and risk stratification of thyroid microcarcinoma: a multicenter pathological and clinical study. Mod Pathol 2015;28(10):1343-59, doi:10.1038/modpathol.2015.92

209. Tian HY, Yu ZY, Dong T, et al. Risk factors of cervical central lymph node metastasis in stage T1a unifocal papillary thyroid carcinoma. Sci Rep 2024;14(1):25577, doi:10.1038/s41598-024-77681-3

210. Zhou W, Li L, Hao X, et al. Predicting central lymph node metastasis in papillary thyroid microcarcinoma: a breakthrough with interpretable machine learning. Front Endocrinol (Lausanne) 2025;16:1537386, doi:10.3389/fendo.2025.1537386

211. Zhang X, Chen W, Fang Q, et al. Lateral Lymph Node Metastases in T1a Papillary Thyroid Carcinoma: Stratification by Tumor Location and Size. Front Endocrinol (Lausanne) 2021;12:716082, doi:10.3389/fendo.2021.716082

212. Zhao YZ, He NA, Ye XJ, et al. Analysis of Risk Factors Associated With Central Lymph Node Metastasis in Papillary Thyroid Carcinoma With cT1N0 Stage. Front Endocrinol (Lausanne) 2022;13:880911, doi:10.3389/fendo.2022.880911

213. Yoo YS, Kim SS, Mun SP, et al. Clinicopathologic Findings of Micropapillary Carcinomas, according to Tumor Size. Journal of the Korean Surgical Society 2009;76(6), doi:10.4174/jkss.2009.76.6.348

214. Zahan AE, Nechifor Boilă A, Pașcanu I, et al. Papillary Thyroid Microcarcinomas: a 25 Years Retrospective, Institutional Study of 255 Cases. Acta Medica Marisiensis 2016;62(1):41-46, doi:10.1515/amma-2015-0108

215. Zhang X, Zhang L, Xue S, et al. Predictive factors of lateral lymph node metastasis in solitary papillary thyroid microcarcinoma without gross extrathyroidal extension. Asian J Surg 2019;42(4):563-570, doi:10.1016/j.asjsur.2018.07.003

216. Lee EK, Moon JH, Hwangbo Y, et al. Progression of Low-Risk Papillary Thyroid Microcarcinoma During Active Surveillance: Interim Analysis of a Multicenter Prospective Cohort Study of Active Surveillance on Papillary Thyroid Microcarcinoma in Korea. Thyroid 2022;32(11):1328-1336, doi:10.1089/thy.2021.0614

217. Ito Y, Miyauchi A, Kihara M, et al. Patient age is significantly related to the progression of papillary microcarcinoma of the thyroid under observation. Thyroid 2014;24(1):27-34, doi:10.1089/thy.2013.0367

218. Kwon H, Oh HS, Kim M, et al. Active Surveillance for Patients With Papillary Thyroid Microcarcinoma: A Single Center's Experience in Korea. J Clin Endocrinol Metab 2017;102(6):1917-1925, doi:10.1210/jc.2016-4026

219. Nagaoka R, Ebina A, Toda K, et al. Multifocality and Progression of Papillary Thyroid Microcarcinoma During Active Surveillance. World J Surg 2021;45(9):2769-2776, doi:10.1007/s00268-021-06185-2

220. Shindo H, Amino N, Ito Y, et al. Papillary thyroid microcarcinoma might progress during pregnancy. Thyroid 2014;24(5):840-4, doi:10.1089/thy.2013.0527

221. Ghirri A, Campopiano MC, Prete A, et al. Effect of Pregnancy and Menopause on Micropapillary Thyroid Carcinomas During Active Surveillance. J Endocr Soc 2023;7(9):bvad109, doi:10.1210/jendso/bvad109

222. Ito Y, Miyauchi A, Kudo T, et al. Effects of Pregnancy on Papillary Microcarcinomas of the Thyroid Re-Evaluated in the Entire Patient Series at Kuma Hospital. Thyroid 2016;26(1):156-60, doi:10.1089/thy.2015.0393

223. Liu W, Cao W, Dong Z, et al. Can Active Surveillance Management be Developed for Patients With Low-Risk Papillary Thyroid Microcarcinoma? A Preliminary Investigation in a Chinese Population. Endocr Pract 2022;28(4):391-397, doi:10.1016/j.eprac.2022.01.013

224. de Biase D, Gandolfi G, Ragazzi M, et al. TERT Promoter Mutations in Papillary Thyroid Microcarcinomas. Thyroid 2015;25(9):1013-9, doi:10.1089/thy.2015.0101

225. Liu Z, Zeng W, Liu C, et al. Diagnostic accuracy of ultrasonographic features for lymph node metastasis in papillary thyroid microcarcinoma: a single-center retrospective study. World J Surg Oncol 2017;15(1):32, doi:10.1186/s12957-017-1099-2

226. Ciobanu Apostol D, Giuşcă SE, Căruntu ID, Lozneanu L, Andriescu EC, Moscalu M. Relationships between clinicopathological prognostic factors in papillary thyroid microcarcinoma: a refined analysis based on 428 cases. Int J Clin Exp Pathol 2017;10(8):8944-8956

227. Lin JD, Chen ST, Chao TC, Hsueh C, Weng HF. Diagnosis and Therapeutic Strategy for Papillary Thyroid Microcarcinoma. Arch Surg 2005;140:940-945, doi: 10.1001/archsurg.140.10.940

228. Peng C, Wei S, Zheng X, et al. Clinicopathological features and risk factors for central compartment nodal metastasis in papillary thyroid microcarcinoma: a study of 1 401 patients. Chin J Clin Oncol 2016;43(3), doi:10.3969/j.issn.1000-8179.2016.2016.03.187

229. Bircan HY, Koc B, Akarsu C, et al. Is Hashimoto’s thyroiditis a prognostic factor for thyroid papillary microcarcinoma? Eur Rev Med Pharmacol Sci 2014;18:1910-1915

230. Li M, Zhu XY, Lv J, et al. Risk factors for predicting central lymph node metastasis in papillary thyroid microcarcinoma (CN0): a study of 273 resections. Eur Rev Med Pharmacol Sci 2017;21:3801-3807

231. Liu H, Qian CL, Shen ZY, Ji F. The clinical evaluation of the relationship between papillary thyroid microcarcinoma and Hashimoto’s thyroiditis. Int J Clin Exp Med 2016;9(5):8348-8354

232. Liu Z, Zeng W, Liu C, et al. Diagnostic accuracy of ultrasonographic features for lymph node metastasis in papillary thyroid microcarcinoma: a single-center retrospective study. World J Surg Oncol 2017;15(1):32, doi: 10.1186/s12957-017-1099-2

233. Liu C, Wang S, Zeng W, et al. The effects of maximum tumor diameter on metastasic number of lymph node in papillary thyroid microcarcinoma. Int J Clin Exp Med 2017;10(4):6958-6965

234. Zhang L, Yang J, Sun Q, et al. Risk factors for high-volume lymph node metastases in cN0 papillary thyroid microcarcinoma. Chin J Clin Oncol 2017;44(16):805-809, doi:10.3969/j.issn.1000-8179.2017.16.352

235. Zheng HH, Gan MF, Zhang LN, et al. BRAFV600E gene mutation can predict central lymph node metastasis in thyroid papillary microcarcinoma. Kuwait Medical Journal 2020;52(1):61-65

236. Dzepina D, Bedekovic V, Cupic H, Kruslin B. Papillary Thyroid Microcarcinoma: Clinical and Pathological Study of 321 Cases. Coll Antropol 2012;36:39-45

237. Wang Y, Han J, Zhang G, Hu Y. Risk factors analysis of cervical lymph node metastasis of papillary thyroid microcarcinoma. Biomedical Research 2017;28(21):9571-9578

238. Wang Y, Han J, Lv Y, Zhang G. Risk factors analysis of lymph node metastasis in central zone of papillary thyroid microcarcinoma. Biomedical Research 2017;28(21):9567-9570

239. Nechifor-Boilă AC, Szász EA, Descotes F, et al. Morphological features predictive for BRAFV600E mutation in papillary thyroid microcarcinomas. Rom J Morphol Embryol 2018;59(3):747-753

240. Goran M, Pekmezovic T, Markovic I, et al. Lymph node metastases in clinically N0 patients with papillary thyroid microcarcinomas - a single institution experience. J BUON 2017;22(1):224-231

241. Goran M, Markovic I, Buta M, et al. The influence of papillary thyroid microcarcinomas size on the occurrence of lymph node metastases. J BUON 2019;24(5):2120-2126

242. Meilinger-Dobra M, Remenár É, Fröhlich G, et al. Pajzsmirigy-mikrokarcinómás eseteink retrospektív áttekintése a 2001–2010. közötti időszakban. Magy Onkol 2018;62:153-158

243. Wada N, Duh QY, Sugino K, et al. Lymph Node Metastasis From 259 Papillary Thyroid Microcarcinomas. Ann Surg 2003;237(3):399-407

244. Lu S, Zhao R, Ni Y, et al. Development and validation of a nomogram for preoperative prediction of cervical lymph node involvement in thyroid microcarcinoma. AGING 2020;12(6):4896-4906

245. Chen BD, Zhang Z, Wang KK, et al. A multivariable model of BRAF(V600E) and ultrasonographic features for predicting the risk of central lymph node metastasis in cN0 papillary thyroid microcarcinoma. Cancer Manag Res 2019;11:7211-7217, doi:10.2147/CMAR.S199921

246. Karatzas T, Vasileiadis I, Charitoudis G, et al. Bilateral versus unilateral papillary thyroid microcarcinoma: predictive factors and associated histopathological findings following total thyroidectomy. Hormones 2013;12(4):529-536, doi: 10.14310/horm.2002.1441

247. Liu Z, Maimaiti Y, Yu P, et al. Correlation between body mass index and clinicopathological features of papillary thyroid microcarcinoma. Int J Clin Exp Med 2015;8(9):16472-16479

248. Xu D, Lv X, Wang S, Dai W. Risk factors for predicting central lymph node metastasis in papillary thyroid microcarcinoma. Int J Clin Exp Pathol 2014;7(9):6199-6205

249. He LY, Chen ML, Wang WW, et al. The analysis of clinicopathologic predictors of lymph node metastasis and postoperative recurrence in patients with papillary thyroid microcarcinoma from Guangdong Province, China-a multicenter retrospective study. Int J Clin Exp Pathol 2017;10(9):9735-9743

250. Ji W, Xie H, Wei B, et al. Relationship between BRAF V600E gene mutation and the clinical and pathologic characteristics of papillary thyroid microcarcinoma. Int J Clin Exp Pathol 2019;12(9):3492-3499

251. Sun K, He X, Guo L. Clinical and ultrasonic features of papillary thyroid microcarcinoma and risk factors for central lymph node metastasis. J Clin Otorhinolaryngol Head Neck Surg 2021;35(3):260-263, doi:10.13201/j.issn.2096-79932.2021.03.015

252. Pan XJ, Hu DX, Su XL. Clinical value of central lymph node dissection and intraoperative frozen-section examination in the treatment of papillary thyroid microcarcinoma. J Shanghai Jiaotong Univ (Med Sci) 2018;38(2):180-183, doi:10.3969/j.issn.1674-8115.2018.02.012

253. Li XJ, Yang L, Ma BL. Risk Factors for central lymph node metastasis in 1988 papillary thyroid microcarcinoma patients. Lin Chuang Er Bi Yan Hou Tou Jing Wai Ke Za Zhi 2017;31(1):52-57, doi:10.13201/j.issn.1001-1781.2017.01.014

254. Rosario PW, Mourão GF. Pregnancy During Active Surveillance of Papillary Thyroid Microcarcinoma. J Endocrine Soc 2021;5(1):A868, doi:10.1210/jendso/bvab048

255. Ge Y, Zheng B, Zhou J, et al. Analysis of the initial results of active surveillance of the papillary thyroid microcarcinoma and related factors for its progress Chin J Endocr Surg 2024;18(4):500-504, doi:10.3760/cma.j.cn115807-20231211-00185

256. Ruiz Pardo J, Ríos Zambudio A, Rodríguez González JM, et al. Papillary thyroid microcarcinoma with minimal extrathyroidal extension. Is its course so indolent that it requires a less aggressive treatment? Revista Clinica Espanola 2021;221:131-138

257. Xu D, Lv X, Wang S, Dai W. Risk factors for predicting central lymph node metastasis in papillary thyroid microcarcinoma. Int J Clin Exp Pathol 2014;7(9):6199-6205 doi:10.13201/j.issn.1001-1781.201.2014.06.002

258. Kim HK, Lee JS, Kim SY, et al. Risk Factors for Lateral Neck Lymph Node Metastasis in Papillary Thyroid Ultra-Micro- Carcinoma: Implications for Active Surveillance. Research Square 2025, doi:10.21203/rs.3.rs-6344218/v1

259. Lee JB, Kim HY, Woo SW, et al. The clinical significance of minimal extrathyroid extension in patients with papillary thyroid microcarcinoma. Korean J Endocrine Surg 2008;8:238-249

260. Saad HA, Farid MI, Eraky ME, et al. A nomogram for lateral lymph nodes that have metastatic cN0 unifocal papillary thyroid microcarcinoma. Research Square 2023, doi:10.21203/rs.3.rs-3301085/v1

261. Caliskan M, Park JH, Jeong JS, et al. Role of prophylactic ipsilateral central compartment lymph node dissection in papillary thyroid microcarcinoma. Endocrine Journal 2012;59(4):305-311

262. Mei X, Zhang Y, Wang J, et al. Impact factors for cervical central lymph node metastasis of papillary thyroid microcarcinoma. Chin J Interv Imaging Ther 2023;20(11):680-684, doi:10.13929/j.issn.1672-8475.2023.11.009

263. Tang J, Miao R, Yang L, et al.Prognostic value of Doppler ultrasound combined with Cyfra21 ⁃ 1 and sIL ⁃ 2R in cervical lymph node metastasis of thyroid micropapillary carcinoma Chin J Endocr Surg 2024;18(5):691-695, doi:10.3760/cma.j.cn115807-20240205-00038

264. Wang CJ, Zhai ZG, Zhang L, et al. Association of LOP/C and BRAFV600E gene mutations with cervical lymph node metastasis in papillary thyroid microcarcinoma. Chin J Cancer Prev Treat 2022;29(13):990-995, doi:10.16073/j.cnki.cjcpt.2022.13.07

265. Xia T, Zhao J, Yu Y, et al. Clinical Analysis of Lateral Cervical Lymph Node Metastases from Papillary Thyroid Microcarcinoma. Chin J Clin Oncol 2011;38(24):1588-1590, doi:10.3969/j.issn.100-8179.2011.24.028

266. Ma L, Wang S, Li Y, et al. Risk factors for lateral cervical lymph node metastasis in patients with papillary thy- roid carcinoma harboring BRAFV600E mutations. Chin J Clin Oncol 2021;48(5):243-247, doi:10.3969/j.issn.10008179.2021.05.420

267. Bian X, Sun S, Guo W, et al. Risk factor analysis for cervical nodal metastasis in papillary microcarcinoma. Chin J Clin Oncol 2015;42(13):658-662, doi:10.3969/j.issn.1000-8179.20150156

268. Jiang L, Chen J, Chen C, et al. Risk predictors for central lymph node metastasis and recurrence in CN0 papillary thyroid microcarcinoma. Int J Clin Exp Med 2018;11(11):12387-12395, doi:1940-5901/IJCEM0070108

269. Yu H, Wang Q, Zha SL, et al. Patterns and risk factors of central lymph node metastasis in cN0 papillary thyroid microcarcinoma with tumor maximum diameter of 5 mm or below. Academic Journal of Second Military Medical University 2018;39:101-104, doi:10.16781/j.0258-879x.2018.01.0101

270. Zhang H, Gong S, Liu Y, et al. Analysis of risk factors for level Ⅵ lymph node metastasis in cN0 papillary thyroid microcarcinoma. Chin J Anat Clin 2023;28(6), doi:10.3760/cma.j.cn101202-20221107-00346

271. Wang Z, Zhang Y, Fu G, Chen Y. Analysis of risk factors for central lymph node metastasis in papillary thyroid microcarcinoma. Chin J Otorhinolaryngol Head Neck Surg 2019;54:12-17, doi:10.3760/cma.j.issn.1673⁃0860.2019.01.004

272. Song DK, Choi YJ, Kang YJ, et al. Clinical Characteristics and Prognostic Factors of Papillary Microcarcinoma: Results from Retrospective Analysis of 176 Patients. Korean J Endocrine Surg 2009;9(2):79-84, doi: 10.16956/kjes.2009.9.2.79

273. Su Y, Sun T, Wu Y, et al. Predictive model and clinical application for lymph node metastasis in papillary thyroid microcarcinoma. Research Square 2024, doi:10.21203/rs.3.rs-4560286/v1

274. Sun H, Liu M. Risk factors of central cervical lymph node metastasis in thyroid micropapillary carcinoma. Research Square 2022, doi:10.21203/rs.3.rs-2233486/v1
